# Mapping disparities in education across low- and middle-income countries

**DOI:** 10.1038/s41586-019-1872-1

**Published:** 2019-12-25

**Authors:** Local Burden of Disease Educational Attainment Collaborators, Nicholas Graetz, Lauren Woyczynski, Katherine F. Wilson, Jason B. Hall, Kalkidan Hassen Abate, Foad Abd-Allah, Oladimeji M. Adebayo, Victor Adekanmbi, Mahdi Afshari, Olufemi Ajumobi, Tomi Akinyemiju, Fares Alahdab, Ziyad Al-Aly, Jacqueline Elizabeth Alcalde Rabanal, Mehran Alijanzadeh, Vahid Alipour, Khalid Altirkawi, Mohammadreza Amiresmaili, Nahla Hamed Anber, Catalina Liliana Andrei, Mina Anjomshoa, Carl Abelardo T. Antonio, Jalal Arabloo, Olatunde Aremu, Krishna K. Aryal, Mehran Asadi-Aliabadi, Suleman Atique, Marcel Ausloos, Ashish Awasthi, Beatriz Paulina Ayala Quintanilla, Samad Azari, Alaa Badawi, Joseph Adel Mattar Banoub, Suzanne Lyn Barker-Collo, Anthony Barnett, Neeraj Bedi, Derrick A. Bennett, Natalia V. Bhattacharjee, Krittika Bhattacharyya, Suraj Bhattarai, Zulfiqar A. Bhutta, Ali Bijani, Boris Bikbov, Gabrielle Britton, Roy Burstein, Zahid A. Butt, Rosario Cárdenas, Félix Carvalho, Carlos A. Castañeda-Orjuela, Franz Castro, Ester Cerin, Jung-Chen Chang, Michael L. Collison, Cyrus Cooper, Michael A. Cork, Farah Daoud, Rajat Das Gupta, Nicole Davis Weaver, Jan-Walter De Neve, Kebede Deribe, Beruk Berhanu Desalegn, Aniruddha Deshpande, Melaku Desta, Meghnath Dhimal, Daniel Diaz, Mesfin Tadese Dinberu, Shirin Djalalinia, Manisha Dubey, Eleonora Dubljanin, Andre R. Durães, Laura Dwyer-Lindgren, Lucas Earl, Mohammad Ebrahimi Kalan, Ziad El-Khatib, Babak Eshrati, Mahbobeh Faramarzi, Mohammad Fareed, Andre Faro, Seyed-Mohammad Fereshtehnejad, Eduarda Fernandes, Irina Filip, Florian Fischer, Takeshi Fukumoto, Jose A. García, Paramjit Singh Gill, Tiffany K. Gill, Philimon N. Gona, Sameer Vali Gopalani, Ayman Grada, Yuming Guo, Rajeev Gupta, Vipin Gupta, Arvin Haj-Mirzaian, Arya Haj-Mirzaian, Randah R. Hamadeh, Samer Hamidi, Mehedi Hasan, Hamid Yimam Hassen, Delia Hendrie, Andualem Henok, Nathaniel J. Henry, Bernardo Hernández Prado, Claudiu Herteliu, Michael K. Hole, Naznin Hossain, Mehdi Hosseinzadeh, Guoqing Hu, Olayinka Stephen Ilesanmi, Seyed Sina Naghibi Irvani, Sheikh Mohammed Shariful Islam, Neda Izadi, Mihajlo Jakovljevic, Ravi Prakash Jha, John S. Ji, Jost B. Jonas, Zahra Jorjoran Shushtari, Jacek Jerzy Jozwiak, Tanuj Kanchan, Amir Kasaeian, Ali Kazemi Karyani, Peter Njenga Keiyoro, Chandrasekharan Nair Kesavachandran, Yousef Saleh Khader, Morteza Abdullatif Khafaie, Ejaz Ahmad Khan, Mona M. Khater, Aliasghar A. Kiadaliri, Daniel N. Kiirithio, Yun Jin Kim, Ruth W. Kimokoti, Damaris K. Kinyoki, Adnan Kisa, Soewarta Kosen, Ai Koyanagi, Kewal Krishan, Barthelemy Kuate Defo, Manasi Kumar, Pushpendra Kumar, Faris Hasan Lami, Paul H. Lee, Aubrey J. Levine, Shanshan Li, Yu Liao, Lee-Ling Lim, Stefan Listl, Jaifred Christian F. Lopez, Marek Majdan, Reza Majdzadeh, Azeem Majeed, Reza Malekzadeh, Mohammad Ali Mansournia, Francisco Rogerlândio Martins-Melo, Anthony Masaka, Benjamin Ballard Massenburg, Benjamin K. Mayala, Kala M. Mehta, Walter Mendoza, George A. Mensah, Tuomo J. Meretoja, Tomislav Mestrovic, Ted R. Miller, G. K. Mini, Erkin M. Mirrakhimov, Babak Moazen, Dara K. Mohammad, Aso Mohammad Darwesh, Shafiu Mohammed, Farnam Mohebi, Ali H. Mokdad, Lorenzo Monasta, Yoshan Moodley, Mahmood Moosazadeh, Ghobad Moradi, Maziar Moradi-Lakeh, Paula Moraga, Lidia Morawska, Shane Douglas Morrison, Jonathan F. Mosser, Seyyed Meysam Mousavi, Christopher J. L. Murray, Ghulam Mustafa, Azin Nahvijou, Farid Najafi, Vinay Nangia, Duduzile Edith Ndwandwe, Ionut Negoi, Ruxandra Irina Negoi, Josephine W. Ngunjiri, Cuong Tat Nguyen, Long Hoang Nguyen, Dina Nur Anggraini Ningrum, Jean Jacques Noubiap, Malihe Nourollahpour Shiadeh, Peter S. Nyasulu, Felix Akpojene Ogbo, Andrew T. Olagunju, Bolajoko Olubukunola Olusanya, Jacob Olusegun Olusanya, Obinna E. Onwujekwe, Doris D. V. Ortega-Altamirano, Eduardo Ortiz-Panozo, Simon Øverland, Mahesh P. A., Adrian Pana, Songhomitra Panda-Jonas, Sanghamitra Pati, George C. Patton, Norberto Perico, David M. Pigott, Meghdad Pirsaheb, Maarten J. Postma, Akram Pourshams, Swayam Prakash, Parul Puri, Mostafa Qorbani, Amir Radfar, Fakher Rahim, Vafa Rahimi-Movaghar, Mohammad Hifz Ur Rahman, Fatemeh Rajati, Chhabi Lal Ranabhat, David Laith Rawaf, Salman Rawaf, Robert C. Reiner, Giuseppe Remuzzi, Andre M. N. Renzaho, Satar Rezaei, Aziz Rezapour, Carlos Rios-González, Leonardo Roever, Luca Ronfani, Gholamreza Roshandel, Ali Rostami, Enrico Rubagotti, Nafis Sadat, Ehsan Sadeghi, Yahya Safari, Rajesh Sagar, Nasir Salam, Payman Salamati, Yahya Salimi, Hamideh Salimzadeh, Abdallah M. Samy, Juan Sanabria, Milena M. Santric Milicevic, Benn Sartorius, Brijesh Sathian, Arundhati R. Sawant, Lauren E. Schaeffer, Megan F. Schipp, David C. Schwebel, Anbissa Muleta Senbeta, Sadaf G. Sepanlou, Masood Ali Shaikh, Mehran Shams-Beyranvand, Morteza Shamsizadeh, Kiomars Sharafi, Rajesh Sharma, Jun She, Aziz Sheikh, Mika Shigematsu, Soraya Siabani, Dayane Gabriele Alves Silveira, Jasvinder A. Singh, Dhirendra Narain Sinha, Vegard Skirbekk, Amber Sligar, Badr Hasan Sobaih, Moslem Soofi, Joan B. Soriano, Ireneous N. Soyiri, Chandrashekhar T. Sreeramareddy, Agus Sudaryanto, Mu’awiyyah Babale Sufiyan, Ipsita Sutradhar, PN Sylaja, Rafael Tabarés-Seisdedos, Birkneh Tilahun Tadesse, Mohamad-Hani Temsah, Abdullah Sulieman Terkawi, Belay Tessema, Zemenu Tadesse Tessema, Kavumpurathu Raman Thankappan, Roman Topor-Madry, Marcos Roberto Tovani-Palone, Bach Xuan Tran, Lorainne Tudor Car, Irfan Ullah, Olalekan A. Uthman, Pascual R. Valdez, Yousef Veisani, Francesco S. Violante, Vasily Vlassov, Sebastian Vollmer, Giang Thu Vu, Yasir Waheed, Yuan-Pang Wang, John C. Wilkinson, Andrea Sylvia Winkler, Charles D. A. Wolfe, Tomohide Yamada, Alex Yeshaneh, Paul Yip, Engida Yisma, Naohiro Yonemoto, Mustafa Z. Younis, Mahmoud Yousefifard, Chuanhua Yu, Sojib Bin Zaman, Jianrong Zhang, Yunquan Zhang, Sanjay Zodpey, Emmanuela Gakidou, Simon I. Hay

**Affiliations:** 1 Institute for Health Metrics and Evaluation, University of Washington, Seattle, WA USA; 2 Department of Population and Family Health, Jimma University, Jimma, Ethiopia; 3 Department of Neurology, Cairo University, Cairo, Egypt; 4 Department of Medicine, University College Hospital, Ibadan, Nigeria; 5 School of Medicine, Cardiff University, Cardiff, UK; 6 Department of Community Medicine, Zabol University of Medical Sciences, Zabol, Iran; 7 School of Community Health Sciences, University of Nevada, Reno, NV USA; 8 National Malaria Elimination Program, Federal Ministry of Health, Abuja, Nigeria; 9 Duke Global Health Institute, Duke University, Durham, NC USA; 10 Department of Population Health Sciences, Duke University, Durham, NC USA; 11 Evidence Based Practice Center, Mayo Clinic Foundation for Medical Education and Research, Rochester, MN USA; 12 Internal Medicine Department, Washington University in St Louis, St Louis, MO USA; 13 Clinical Epidemiology Center, VA Saint Louis Health Care System, Department of Veterans Affairs, St Louis, MO USA; 14 Center for Health Systems Research, National Institute of Public Health, Cuernavaca, Mexico; 15 Qazvin University of Medical Sciences, Qazvin, Iran; 16 Health Management and Economics Research Center, Iran University of Medical Sciences, Tehran, Iran; 17 King Saud University, Riyadh, Saudi Arabia; 18 Department of Health Management, Policy and Economics, Kerman University of Medical Sciences, Kerman, Iran; 19 Faculty of Medicine, Mansoura University, Mansoura, Egypt; 20 Carol Davila University of Medicine and Pharmacy, Bucharest, Romania; 21 Social Determinants of Health Research Center, Rafsanjan University of Medical Sciences, Rafsanjan, Iran; 22 Department of Health Policy and Administration, University of the Philippines Manila, Manila, The Philippines; 23 Department of Applied Social Sciences, Hong Kong Polytechnic University, Hong Kong, China; 24 School of Health Sciences, Birmingham City University, Birmingham, UK; 25 Monitoring Evaluation and Operational Research Project, ABT Associates Nepal, Lalitpur, Nepal; 26 Preventive Medicine and Public Health Research Center, Iran University of Medical Sciences, Tehran, Iran; 27 Department of Health Informatics, University of Ha’il, Ha’il, Saudi Arabia; 28 Department of Statistics and Econometrics, Bucharest University of Economic Studies, Bucharest, Romania; 29 Indian Institute of Public Health, Public Health Foundation of India, Gurugram, India; 30 The Judith Lumley Centre, La Trobe University, Melbourne, Victoria Australia; 31 General Office for Research and Technological Transfer, Peruvian National Institute of Health, Lima, Peru; 32 Public Health Risk Sciences Division, Public Health Agency of Canada, Toronto, Ontario Canada; 33 Department of Nutritional Sciences, University of Toronto, Toronto, Ontario Canada; 34 Faculty of Medicine, Alexandria University, Alexandria, Egypt; 35 School of Psychology, University of Auckland, Auckland, New Zealand; 36 Mary MacKillop Institute for Health Research, Australian Catholic University, Melbourne, Victoria Australia; 37 Department of Community Medicine, Gandhi Medical College Bhopal, Bhopal, India; 38 Jazan University, Jazan, Saudi Arabia; 39 Nuffield Department of Population Health, University of Oxford, Oxford, UK; 40 Department of Statistical and Computational Genomics, National Institute of Biomedical Genomics, Kalyani, India; 41 Department of Statistics, University of Calcutta, Kolkata, India; 42 Department of Global Health, Global Institute for Interdisciplinary Studies, Kathmandu, Nepal; 43 Centre for Global Child Health, University of Toronto, Toronto, Ontario Canada; 44 Centre of Excellence in Women and Child Health, Aga Khan University, Karachi, Pakistan; 45 Social Determinants of Health Research Center, Babol University of Medical Sciences, Babol, Iran; 46 Istituto di Ricerche Farmacologiche Mario Negri IRCCS, Ranica, Italy; 47 Center for Neuroscience, Instituto de Investigaciones Científicas y Servicios de Alta Tecnología (INDICASAT AIP), Panama, Panama; 48 School of Public Health and Health Systems, University of Waterloo, Waterloo, Ontario Canada; 49 Al Shifa School of Public Health, Al Shifa Trust Eye Hospital, Rawalpindi, Pakistan; 50 Department of Population and Health, Metropolitan Autonomous University, Mexico City, Mexico; 51 Institute of Public Health, University of Porto, Porto, Portugal; 52 Applied Molecular Biosciences Unit, University of Porto, Porto, Portugal; 53 Colombian National Health Observatory, National Institute of Health, Bogota, Colombia; 54 Epidemiology and Public Health Evaluation Group, National University of Colombia, Bogota, Colombia; 55 Gorgas Memorial Institute for Health Studies, Panama, Panama; 56 School of Public Health, University of Hong Kong, Hong Kong, China; 57 College of Medicine, National Taiwan University, Taipei, Taiwan; 58 Medical Research Council Lifecourse Epidemiology Unit, University of Southampton, Southampton, UK; 59 Department of Rheumatology, University of Oxford, Oxford, UK; 60 Department of Epidemiology and Biostatistics, University of South Carolina, Columbia, SC USA; 61 James P. Grant School of Public Health, Brac University, Dhaka, Bangladesh; 62 Heidelberg Institute of Global Health, Heidelberg University, Heidelberg, Germany; 63 Department of Global Health and Infection, Brighton and Sussex Medical School, Brighton, UK; 64 School of Public Health, Addis Ababa University, Addis, Ababa Ethiopia; 65 School of Nutrition, Food Science and Technology, Hawassa University, Hawassa, Ethiopia; 66 Department of Midwifery, Debre Markos University, Debre, Markos Ethiopia; 67 Faculty of Veterinary Medicine and Zootechnics, Autonomous University of Sinaloa, Culiacan Rosales, Mexico; 68 Health Research Section, Nepal Health Research Council, Kathmandu, Nepal; 69 Center of Complexity Sciences, National Autonomous University of Mexico, Mexico City, Mexico; 70 Department of Midwifery, Debre Berhan University, Debre Berhan, Ethiopia; 71 Deputy of Research and Technology, Ministry of Health and Medical Education, Tehran, Iran; 72 United Nations World Food Programme, New Delhi, India; 73 Faculty of Medicine, University of Belgrade, Belgrade, Serbia; 74 Department of Internal Medicine, Bahia School of Medicine and Public Health, Salvador, Brazil; 75 Medical Board, Roberto Santos General Hospital, Salvador, Brazil; 76 Department of Health Metrics Sciences, School of Medicine, University of Washington, Seattle, WA USA; 77 Epidemiology Department, Florida International University, Miami, FL USA; 78 Department of Public Health Sciences, Karolinska Institutet, Stockholm, Sweden; 79 World Health Programme, Université du Québec en Abitibi-Témiscamingue, Rouyn-Noranda, Quebec, Canada; 80 Center of Communicable Disease Control, Ministry of Health and Medical Education, Tehran, Iran; 81 School of Public Health, Arak University of Medical Sciences, Arak, Iran; 82 Babol University of Medical Sciences, Babol, Iran; 83 College of Medicine, Imam Mohammad Ibn Saud Islamic University, Riyadh, Saudi Arabia; 84 Department of Psychology, Federal University of Sergipe, Sao Cristovao, Brazil; 85 Department of Neurobiology, Care Sciences and Society, Karolinska Institutet, Stockholm, Sweden; 86 Division of Neurology, University of Ottawa, Ottawa, Ontario Canada; 87 REQUIMTE/LAQV, University of Porto, Porto, Portugal; 88 Psychiatry Department, Kaiser Permanente, Fontana, CA USA; 89 Department of Health Sciences, A. T. Still University, Mesa, AZ USA; 90 Department of Population Medicine and Health Services Research, Bielefeld University, Bielefeld, Germany; 91 Department of Dermatology, Kobe University, Kobe, Japan; 92 Gene Expression & Regulation Program, The Wistar Institute, Philadelphia, PA USA; 93 Ramón de la Fuente Muñiz National Institute of Psychiatry, Mexico City, Mexico; 94 Unit of Academic Primary Care, University of Warwick, Coventry, UK; 95 Adelaide Medical School, University of Adelaide, Adelaide, South Australia Australia; 96 Nursing and Health Sciences Department, University of Massachusetts Boston, Boston, MA USA; 97 Department of Biostatistics and Epidemiology, University of Oklahoma, Oklahoma City, OK USA; 98 Department of Health and Social Affairs, Government of the Federated States of Micronesia, Palikir, Federated States of Micronesia; 99 School of Medicine, Boston University, Boston, MA USA; 100 School of Public Health and Preventive Medicine, Monash University, Melbourne, Victoria Australia; 101 Department of Epidemiology and Biostatistics, Zhengzhou University, Zhengzhou, China; 102 Academics and Research Department, Rajasthan University of Health Sciences, Jaipur, India; 103 Department of Medicine, Mahatma Gandhi University of Medical Sciences & Technology, Jaipur, India; 104 Department of Anthropology, University of Delhi, Delhi, India; 105 Department of Pharmacology, Tehran University of Medical Sciences, Tehran, Iran; 106 Obesity Research Center, Research Institute for Endocrine Sciences, Shahid Beheshti University of Medical Sciences, Tehran, Iran; 107 Department of Radiology, Johns Hopkins University, Baltimore, MD USA; 108 Department of Family and Community Medicine, Arabian Gulf University, Manama, Bahrain; 109 School of Health and Environmental Studies, Hamdan Bin Mohammed Smart University, Dubai, United Arab Emirates; 110 Department of Public Health, Mizan-Tepi University, Tepi, Ethiopia; 111 Unit of Epidemiology and Social Medicine, University Hospital Antwerp, Antwerp, Belgium; 112 School of Public Health, Curtin University, Perth, Western Australia Australia; 113 Department of Pediatrics, University of Texas Austin, Austin, TX USA; 114 Department of Pharmacology and Therapeutics, Dhaka Medical College, Dhaka, Bangladesh; 115 Department of Pharmacology, Bangladesh Industrial Gases Limited, Tangail, Bangladesh; 116 Department of Computer Engineering, Islamic Azad University, Tehran, Iran; 117 Computer Science Department, University of Human Development, Sulaimaniyah, Iraq; 118 Department of Epidemiology and Health Statistics, Central South University, Changsha, China; 119 Department of Community Medicine, University of Ibadan, Ibadan, Nigeria; 120 Research Institute for Endocrine Sciences, Shahid Beheshti University of Medical Sciences, Tehran, Iran; 121 Institute for Physical Activity and Nutrition, Deakin University, Burwood, Victoria Australia; 122 Sydney Medical School, University of Sydney, Sydney, New South Wales Australia; 123 Department of Epidemiology, Shahid Beheshti University of Medical Sciences, Tehran, Iran; 124 Department of Health Care and Public Health, Sechenov First Moscow State Medical University, Moscow, Russia; 125 Department of Community Medicine, Banaras Hindu University, Varanasi, India; 126 Environmental Research Center, Duke Kunshan University, Kunshan, China; 127 Nicholas School of the Environment, Duke University, Durham, NC USA; 128 Department of Ophthalmology, Heidelberg University, Heidelberg, Germany; 129 Beijing Institute of Ophthalmology, Beijing Tongren Hospital, Beijing, China; 130 Social Determinants of Health Research Center, University of Social Welfare and Rehabilitation Sciences, Tehran, Iran; 131 Department of Family Medicine and Public Health, University of Opole, Opole, Poland; 132 Department of Forensic Medicine and Toxicology, All India Institute of Medical Sciences, Jodhpur, India; 133 Hematology-Oncology and Stem Cell Transplantation Research Center, Tehran University of Medical Sciences, Tehran, Iran; 134 Pars Advanced and Minimally Invasive Medical Manners Research Center, Iran University of Medical Sciences, Tehran, Iran; 135 Research Center for Environmental Determinants of Health, Kermanshah University of Medical Sciences, Kermanshah, Iran; 136 ODeL Campus, University of Nairobi, Nairobi, Kenya; 137 CSIR-Indian Institute of Toxicology Research, Council of Scientific & Industrial Research, Lucknow, India; 138 Department of Public Health, Jordan University of Science and Technology, Irbid, Jordan; 139 Social Determinants of Health Research Center, Ahvaz Jundishapur University of Medical Sciences, Ahvaz, Iran; 140 Epidemiology and Biostatistics Department, Health Services Academy, Islamabad, Pakistan; 141 Department of Medical Parasitology, Cairo University, Cairo, Egypt; 142 Clinical Epidemiology Unit, Lund University, Lund, Sweden; 143 Research and Data Solutions, Synotech Consultants, Nairobi, Kenya; 144 School of Medicine, Xiamen University Malaysia, Sepang, Malaysia; 145 Department of Nutrition, Simmons University, Boston, MA USA; 146 School of Health Sciences, Kristiania University College, Oslo, Norway; 147 Independent Consultant, Jakarta, Indonesia; 148 CIBERSAM, San Juan de Dios Sanitary Park, Sant Boi De Llobregat, Spain; 149 Catalan Institution for Research and Advanced Studies (ICREA), Barcelona, Spain; 150 Department of Anthropology, Panjab University, Chandigarh, India; 151 Department of Social and Preventive Medicine, University of Montreal, Montreal, Quebec Canada; 152 Department of Demography, University of Montreal, Montreal, Quebec Canada; 153 Department of Psychiatry, University of Nairobi, Nairobi, Kenya; 154 Division of Psychology and Language Sciences, University College London, London, UK; 155 International Institute for Population Sciences, Mumbai, India; 156 Department of Community and Family Medicine, University of Baghdad, Baghdad, Iraq; 157 School of Nursing, Hong Kong Polytechnic University, Hong Kong, China; 158 Department of Medical Statistics and Epidemiology, Sun Yat-sen University, Guangzhou, China; 159 Alliance for Improving Health Outcomes Inc, Quezon City, The Philippines; 160 Department of Medicine, University of Malaya, Kuala Lumpur, Malaysia; 161 Department of Medicine and Therapeutics, The Chinese University of Hong Kong, Shatin, China; 162 Department of Dentistry, Radboud University, Nijmegen, The Netherlands; 163 Section for Translational Health Economics, Heidelberg University Hospital, Heidelberg, Germany; 164 Department of Epidemiology and Biostatistics, University of the Philippines Manila, Manila, The Philippines; 165 Department of Public Health, Trnava University, Trnava, Slovakia; 166 Community-Based Participatory-Research Center (CBPR), Tehran University of Medical Sciences, Tehran, Iran; 167 Knowledge Utilization Research Center (KURC), Tehran University of Medical Sciences, Tehran, Iran; 168 Department of Primary Care and Public Health, Imperial College London, London, UK; 169 Digestive Diseases Research Institute, Tehran University of Medical Sciences, Tehran, Iran; 170 Non-communicable Diseases Research Center, Shiraz University of Medical Sciences, Shiraz, Iran; 171 Department of Epidemiology and Biostatistics, Tehran University of Medical Sciences, Tehran, Iran; 172 Campus Caucaia, Federal Institute of Education, Science and Technology of Ceará, Caucaia, Brazil; 173 Public Health Department, Botho University-Botswana, Gaborone, Botswana; 174 Division of Plastic Surgery, University of Washington, Seattle, WA USA; 175 Department of Epidemiology and Biostatistics, University of California San Francisco, San Francisco, CA USA; 176 Peru Country Office, United Nations Population Fund (UNFPA), Lima, Peru; 177 Center for Translation Research and Implementation Science, National Institutes of Health, Bethesda, MD USA; 178 Department of Medicine, University of Cape Town, Cape Town, South Africa; 179 Breast Surgery Unit, Helsinki University Hospital, Helsinki, Finland; 180 University of Helsinki, Helsinki, Finland; 181 Clinical Microbiology and Parasitology Unit, Dr Zora Profozic Polyclinic, Zagreb, Croatia; 182 University Centre Varazdin, University North, Varazdin, Croatia; 183 Pacific Institute for Research & Evaluation, Calverton, MD USA; 184 Achutha Menon Centre for Health Science Studies, Sree Chitra Tirunal Institute for Medical Sciences and Technology, Trivandrum, India; 185 Global Institute of Public Health (GIPH), Ananthapuri Hospitals and Research Centre, Trivandrum, India; 186 Faculty of Internal Medicine, Kyrgyz State Medical Academy, Bishkek, Kyrgyzstan; 187 Department of Atherosclerosis and Coronary Heart Disease, National Center of Cardiology and Internal Disease, Bishkek, Kyrgyzstan; 188 Institute of Addiction Research (ISFF), Frankfurt University of Applied Sciences, Frankfurt, Germany; 189 Department of Food Technology, College of Agriculture, Salahaddin University-Erbil, Erbil, Iraq; 190 Department of Medicine Huddinge, Karolinska Institutet, Stockholm, Sweden; 191 Department of Information Technology, University of Human Development, Sulaimaniyah, Iraq; 192 Health Systems and Policy Research Unit, Ahmadu Bello University, Zaria, Nigeria; 193 Non-communicable Diseases Research Center, Tehran University of Medical Sciences, Tehran, Iran; 194 Iran National Institute of Health Research, Tehran University of Medical Sciences, Tehran, Iran; 195 Clinical Epidemiology and Public Health Research Unit, Burlo Garofolo Institute for Maternal and Child Health, Trieste, Italy; 196 Department of Public Health Medicine, University of Kwazulu-Natal, Durban, South Africa; 197 Health Sciences Research Center, Mazandaran University of Medical Sciences, Sari, Iran; 198 Social Determinants of Health Research Center, Kurdistan University of Medical Sciences, Sanandaj, Iran; 199 Department of Epidemiology and Biostatistics, Kurdistan University of Medical Sciences, Sanandaj, Iran; 200 Department of Mathematical Sciences, University of Bath, Bath, UK; 201 International Laboratory for Air Quality and Health, Queensland University of Technology, Brisbane, Queensland Australia; 202 Department of Surgery, University of Washington, Seattle, WA USA; 203 Department of Health Management and Economics, Tehran University of Medical Sciences, Tehran, Iran; 204 Health Management Research Center, Baqiyatallah University of Medical Sciences, Tehran, Iran; 205 Department of Pediatric Medicine, Nishtar Medical University, Multan, Pakistan; 206 Department of Pediatrics & Pediatric Pulmonology, Institute of Mother & Child Care, Multan, Pakistan; 207 Cancer Research Center, Tehran University of Medical Sciences, Tehran, Iran; 208 Department of Epidemiology & Biostatistics, Kermanshah University of Medical Sciences, Kermanshah, Iran; 209 Suraj Eye Institute, Nagpur, India; 210 Cochrane South Africa, South African Medical Research Council, Cape Town, South Africa; 211 General Surgery, Carol Davila University of Medicine and Pharmacy Bucharest, Bucharest, Romania; 212 General Surgery, Emergency Hospital of Bucharest, Bucharest, Romania; 213 Anatomy and Embryology, Carol Davila University of Medicine and Pharmacy, Bucharest, Romania; 214 Cardiology, Cardio-Aid, Bucharest, Romania; 215 Department of Biological Sciences, University of Embu, Embu, Kenya; 216 Institute for Global Health Innovations, Duy Tan University, Hanoi, Vietnam; 217 Center of Excellence in Behavioral Medicine, Nguyen Tat Thanh University, Ho Chi Minh City, Vietnam; 218 Public Health Department, Universitas Negeri Semarang, Kota Semarang, Indonesia; 219 Graduate Institute of Biomedical Informatics, Taipei Medical University, Taipei City, Taiwan; 220 Mazandaran University of Medical Sciences, Sari, Iran; 221 Faculty of Medicine & Health Sciences, Stellenbosch University, Cape Town, South Africa; 222 UCIBIO, University of Porto, Porto, Portugal; 223 Department of Psychiatry and Behavioural Neurosciences, McMaster University, Hamilton, Ontario Canada; 224 Department of Psychiatry, University of Lagos, Lagos, Nigeria; 225 Centre for Healthy Start Initiative, Lagos, Nigeria; 226 Department of Pharmacology and Therapeutics, University of Nigeria Nsukka, Enugu, Nigeria; 227 Center for Population Health Research, National Institute of Public Health, Cuernavaca, Mexico; 228 School of Health and Welfare, Jönköping University, Jönköping, Sweden; 229 Division of Mental and Physical Health, Norwegian Institute of Public Health, Bergen, Norway; 230 Department of Psychosocial Science, University of Bergen, Bergen, Norway; 231 Department of Respiratory Medicine, Jagadguru Sri Shivarathreeswara Academy of Health Education and Research, Mysore, India; 232 Health Outcomes, Center for Health Outcomes & Evaluation, Bucharest, Romania; 233 Augenpraxis Jonas, Heidelberg University, Heidelberg, Germany; 234 Regional Medical Research Centre, Indian Council of Medical Research, Bhubaneswar, India; 235 Department of Paediatrics, University of Melbourne, Melbourne, Victoria Australia; 236 Population Health, Murdoch Children’s Research Institute, Melbourne, Victoria Australia; 237 Istituto di Ricerche Farmacologiche Mario Negri IRCCS, Bergamo, Italy; 238 Department of Economics and Business, University of Groningen, Groningen, The Netherlands; 239 University Medical Center Groningen, University of Groningen, Groningen, The Netherlands; 240 Department of Nephrology, Sanjay Gandhi Postgraduate Institute of Medical Sciences, Lucknow, India; 241 Population Studies, International Institute for Population Sciences, Mumbai, India; 242 Non-communicable Diseases Research Center, Alborz University of Medical Sciences, Karaj, Iran; 243 College of Medicine, University of Central Florida, Orlando, FL USA; 244 College of Graduate Health Sciences, A. T. Still University, Mesa, AZ USA; 245 Thalassemia and Hemoglobinopathy Research Center, Ahvaz Jundishapur University of Medical Sciences, Ahvaz, Iran; 246 Metabolomics and Genomics Research Center, Tehran University of Medical Sciences, Tehran, Iran; 247 Sina Trauma and Surgery Research Center, Tehran University of Medical Sciences, Tehran, Iran; 248 Department of Public Health and Mortality Studies, International Institute for Population Sciences, Mumbai, India; 249 Policy Research Institute, Kathmandu, Nepal; 250 Institute for Poverty Alleviation and International Development, Yonsei University, Wonju, South Korea; 251 WHO Collaborating Centre for Public Health Education and Training, Imperial College London, London, UK; 252 University College London Hospitals, London, UK; 253 Academic Public Health, Public Health England, London, UK; 254 Translational Health Research Institute, Western Sydney University, Penrith, New South Wales Australia; 255 School of Social Sciences and Psychology, Western Sydney University, Penrith, New South Wales Australia; 256 Research Directorate, Nihon Gakko University, Fernando De La Mora, Paraguay; 257 Research Direction, Universidad Nacional de Caaguazú, Coronel Oviedo, Paraguay; 258 Department of Clinical Research, Federal University of Uberlândia, Uberlândia, Brazil; 259 Golestan Research Center of Gastroenterology and Hepatology, Golestan University of Medical Sciences, Gorgan, Iran; 260 Infectious Diseases and Tropical Medicine Research Center, Babol University of Medical Sciences, Babol, Iran; 261 Centro de Investigación Palmira, Agrosavia, Palmira, Colombia; 262 Department of Ocean Science and Engineering, Southern University of Science and Technology, Shenzhen, China; 263 Kermanshah University of Medical Sciences, Kermanshah, Iran; 264 Department of Psychiatry, All India Institute of Medical Sciences, New Delhi, India; 265 Department of Pathology, Imam Mohammad Ibn Saud Islamic University, Riyadh, Saudi Arabia; 266 Social Development and Health Promotion Research Center, Kermanshah University of Medical Sciences, Kermanshah, Iran; 267 Department of Entomology, Ain Shams University, Cairo, Egypt; 268 Department of Surgery, Marshall University, Huntington, WV USA; 269 Department of Nutrition and Preventive Medicine, Case Western Reserve University, Cleveland, OH USA; 270 Institute of Social Medicine, University of Belgrade, Belgrade, Serbia; 271 Centre-School of Public Health and Health Management, University of Belgrade, Belgrade, Serbia; 272 Faculty of Infectious and Tropical Diseases, London School of Hygiene & Tropical Medicine, London, UK; 273 Surgery Department, Hamad Medical Corporation, Doha, Qatar; 274 Faculty of Health & Social Sciences, Bournemouth University, Bournemouth, UK; 275 University of Alabama at Birmingham, Birmingham, AL USA; 276 Dr D. Y. Patil University, Pune, India; 277 Department of Psychology, University of Alabama at Birmingham, Birmingham, AL USA; 278 Department of Food Science and Nutrition, Jigjiga University, Jigjiga, Ethiopia; 279 Independent Consultant, Karachi, Pakistan; 280 School of Medicine, Dezful University of Medical Sciences, Dezful, Iran; 281 School of Medicine, Alborz University of Medical Sciences, Karaj, Iran; 282 Chronic Diseases (Home Care) Research Center, Hamadan University of Medical Sciences, Hamadan, Iran; 283 University School of Management and Entrepreneurship, Delhi Technological University, New Delhi, India; 284 Department of Pulmonary Medicine, Fudan University, Shanghai, China; 285 Centre for Medical Informatics, University of Edinburgh, Edinburgh, UK; 286 Division of General Internal Medicine, Harvard University, Boston, MA USA; 287 National Institute of Infectious Diseases, Tokyo, Japan; 288 Department of Health Education & Promotion, Kermanshah University of Medical Sciences, Kermanshah, Iran; 289 School of Health, University of Technology Sydney, Sydney, New South Wales Australia; 290 Brasília University, Brasília, Brazil; 291 Department of the Health Industrial Complex and Innovation in Health, Federal Ministry of Health, Brasília, Brazil; 292 Department of Epidemiology, University of Alabama at Birmingham, Birmingham, AL USA; 293 Department of Medicine, University of Alabama at Birmingham, Birmingham, AL USA; 294 Department of Epidemiology, School of Preventive Oncology, Patna, India; 295 Department of Epidemiology, Healis Sekhsaria Institute for Public Health, Mumbai, India; 296 Centre for Fertility and Health, Norwegian Institute of Public Health, Bergen, Norway; 297 Department of Pediatrics, King Saud University, Riyadh, Saudi Arabia; 298 Pediatric Department, King Khalid University Hospital, Riyadh, Saudi Arabia; 299 Hospital Universitario de la Princesa, Autonomous University of Madrid, Madrid, Spain; 300 Centro de Investigación Biomédica en Red Enfermedades Respiratorias (CIBERES), Madrid, Spain; 301 Usher Institute of Population Health Sciences and Informatics, University of Edinburgh, Edinburgh, UK; 302 Hull York Medical School, University of Hull, Hull, UK; 303 Division of Community Medicine, International Medical University, Kuala Lumpur, Malaysia; 304 Department of Nursing, Muhammadiyah University of Surakarta, Kartasura, Indonesia; 305 Department of Public Health, China Medical University, Taichung, Taiwan; 306 Department of Community Medicine, Ahmadu Bello University, Zaria, Nigeria; 307 Neurology Department, Sree Chitra Tirunal Institute for Medical Sciences and Technology, Trivandrum, India; 308 Sree Chitra Tirunal Institute for Medical Sciences and Technology, Trivandrum, India; 309 Department of Medicine, University of Valencia, Valencia, Spain; 310 Carlos III Health Institute, Biomedical Research Networking Center for Mental Health Network (CIBERSAM), Madrid, Spain; 311 Department of Pediatrics, Hawassa University, Hawassa, Ethiopia; 312 International Vaccine Institute, Seoul, South Korea; 313 College of Medicine, Alfaisal University, Riyadh, Saudi Arabia; 314 Department of Anesthesiology, Perioperative, and Pain Medicine, University of Virginia, Charlottesville, VA USA; 315 Department of Anesthesiology, King Farah Medical City, Riyadh, Saudi Arabia; 316 Department of Medical Microbiology, University of Gondar, Gondar, Ethiopia; 317 Department of Epidemiology and Biostatistics, University of Gondar, Gondar, Ethiopia; 318 Department of Public Health and Community Medicine, Central University of Kerala, Kasaragod, India; 319 Faculty of Health Sciences, Jagiellonian University Medical College, Krakow, Poland; 320 The Agency for Health Technology Assessment and Tariff System, Warsaw, Poland; 321 Department of Pathology and Legal Medicine, University of São Paulo, Ribeirão Preto, Brazil; 322 Department of Health Economics, Hanoi Medical University, Hanoi, Vietnam; 323 Lee Kong Chian School of Medicine, Nanyang Technological University, Singapore, Singapore; 324 Gomal Center of Biochemistry and Biotechnology, Gomal University, Dera Ismail Khan, Pakistan; 325 TB Culture Laboratory, Mufti Mehmood Memorial Teaching Hospital, Dera Ismail Khan, Pakistan; 326 Division of Health Sciences, University of Warwick, Coventry, UK; 327 Argentine Society of Medicine, Ciudad de Buenos Aires, Argentina; 328 Velez Sarsfield Hospital, Buenos Aires, Argentina; 329 Psychosocial Injuries Research Center, Ilam University of Medical Sciences, Ilam, Iran; 330 Department of Medical and Surgical Sciences, University of Bologna, Bologna, Italy; 331 Occupational Health Unit, Sant’Orsola Malpighi Hospital, Bologna, Italy; 332 Department of Health Care Administration and Economics, National Research University Higher School of Economics, Moscow, Russia; 333 Department of Global Health and Population, Harvard University, Boston, MA USA; 334 Department of Economics, University of Göttingen, Göttingen, Germany; 335 Foundation University Medical College, Foundation University Islamabad, Islamabad, Pakistan; 336 Department of Psychiatry, University of São Paulo, São Paulo, Brazil; 337 Institute of Health and Society, University of Oslo, Oslo, Norway; 338 Department of Neurology, Technical University of Munich, Munich, Germany; 339 School of Population Health & Environmental Sciences, King’s College London, London, UK; 340 NIHR Biomedical Research Centre, Guy’s and St Thomas’ Hospital and Kings College London, London, UK; 341 Department of Diabetes and Metabolic Diseases, University of Tokyo, Tokyo, Japan; 342 Wolkite University, Wolkite, Ethiopia; 343 Centre for Suicide Research and Prevention, University of Hong Kong, Hong Kong, China; 344 Department of Social Work and Social Administration, University of Hong Kong, Hong Kong, China; 345 School of Allied Health Sciences, Addis Ababa University, Addis Ababa, Ethiopia; 346 Department of Psychopharmacology, National Center of Neurology and Psychiatry, Tokyo, Japan; 347 Health Economics & Finance, Global Health, Jackson State University, Jackson, MS USA; 348 School of Medicine, Tsinghua University, Peking, China; 349 Prevention of Cardiovascular Disease Research Center, Shahid Beheshti University of Medical Sciences, Tehran, Iran; 350 Global Health Institute, Wuhan University, Wuhan, China; 351 Department of Epidemiology and Biostatistics, Wuhan University, Wuhan, China; 352 Department of Medicine, Monash University, Melbourne, Victoria Australia; 353 Maternal and Child Health Division, International Centre for Diarrhoeal Disease Research, Bangladesh, Dhaka, Bangladesh; 354 George Warren Brown School, Washington University in St Louis, St Louis, MO USA; 355 School of Public Health, Wuhan University of Science and Technology, Wuhan, China; 356 Hubei Province Key Laboratory of Occupational Hazard Identification and Control, Wuhan University of Science and Technology, Wuhan, China

**Keywords:** Risk factors, Developing world, Education, Society

## Abstract

Educational attainment is an important social determinant of maternal, newborn, and child health^1–3^. As a tool for promoting gender equity, it has gained increasing traction in popular media, international aid strategies, and global agenda-setting^4–6^. The global health agenda is increasingly focused on evidence of precision public health, which illustrates the subnational distribution of disease and illness^7,8^; however, an agenda focused on future equity must integrate comparable evidence on the distribution of social determinants of health^9–11^. Here we expand on the available precision SDG evidence by estimating the subnational distribution of educational attainment, including the proportions of individuals who have completed key levels of schooling, across all low- and middle-income countries from 2000 to 2017. Previous analyses have focused on geographical disparities in average attainment across Africa or for specific countries, but—to our knowledge—no analysis has examined the subnational proportions of individuals who completed specific levels of education across all low- and middle-income countries^12–14^. By geolocating subnational data for more than 184 million person-years across 528 data sources, we precisely identify inequalities across geography as well as within populations.

## Abstract (web summary)

Analyses of the proportions of individuals who have completed key levels of schooling across all low- and middle-income countries from 2000 to 2017 reveal inequalities across countries as well as within populations.

## Main

Education, as a social determinant of health, is closely linked to several facets of the Sustainable Development Goals (SDGs) of the United Nations^2^. In addition to the explicit focus of SDG 4 on educational attainment, improved gender equality (SDG 5) and maternal, newborn, and child health (SDG 3) have well-documented associations with increased schooling^15–17^. In 2016, after years of deprioritization, aid to education reached its highest level since 2002^18^. Despite this shift, only 22% of aid to basic education—defined as primary and lower-secondary—went to low-income countries in 2016 compared to 36% in 2002^19^. This reflects a persistent pattern in which the distribution of aid does not align with the greatest need, even at the national level. Beyond international aid, domestic policy is also a crucial tool for expanding access to education, especially at higher levels. However, policy-makers often do not have access to a rigorous evidence base at a subnational level. This analysis presents the subnational distribution of education to support the growing evidence base of precision public health data, which shows widespread disparity of health outcomes as well as their social determinants.

## Mapping education across gender

**Fig. 1:**
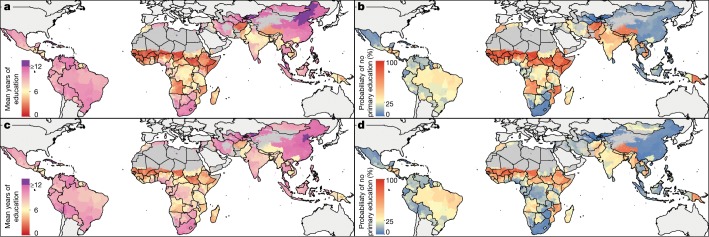
Average educational attainment and proportion of individuals with no completed primary education at the first administrative level and absolute difference between women and men aged 15–49 years. **a–d**, Mean educational attainment for women (**a**) and men (**c**) and the proportion of individuals with no primary school education for women (**b**) and men (**d**) aged 15–49 years in 2017. Maps were produced using ArcGIS Desktop 10.6.

Despite widespread improvement in educational attainment since 2000, gender disparity persists in 2017 in many regions. Figure 1 illustrates the mean number of years of education and the proportion of individuals with no primary school attainment for men and women of reproductive age (15–49 years) in 2017. The average educational attainment is very low across much of the Sahel region of sub-Saharan Africa, consistent with previously published data^14^. In 2017, there was a large gender disparity in many regions, with men attaining higher average education across central and western sub-Saharan Africa and South Asia. Considerable variation remains between the highest- and lowest-performing administrative units within countries in 2017. For Uganda in 2017, this indicator ranged from 1.9 years of education (95% uncertainty interval, 0.8–3.0 years) in rural Kotido to 11.1 years (10.1–12 years) in Kampala, the capital city. Figure 1b, d displays the proportion of men and women aged 15–49 years who have not completed primary school. By considering the variation within populations in different locations, these maps help to identify areas with large populations in the vulnerable lower end of the attainment distribution. We estimated large improvements in the proportions of individuals who have completed primary school in Mexico and China. However, across much of the world women in this age group failed to complete primary school at a much higher rate than their male counterparts.

Despite continued lack of gender parity in education among the reproductive age group, vast progress towards parity has been made among the 20–24 age group. Extended Data Fig. 2 further examines gender parity in 2000 and 2017. This figure highlights two additional advantages of our analytic framework. First, we examined a younger group aged 20–24 years. Although education in this group is less directly relevant to maternal, newborn, and child health than education in the full window of reproductive age, these estimates allowed us to capture how the landscape of education has shifted over time (that is, across successive cohorts) and is therefore more likely to pick up improvements to access and retention in education systems that have been made since 2000. Second, we illustrate the probability that this estimated ratio is credibly different from 1 (parity between sexes) given the full uncertainty in our data and model. In 2000, we estimated that men completed schooling at a higher rate than women across much of the world, particularly for primary school education (that is, the probability that the parity ratio is greater than 1 was over 95%). This was true in most countries for both primary and secondary completion rates, but especially so in Burundi, Angola, Uganda, and Afghanistan (Extended Data Fig. 2a, c). By 2017, many countries moved significantly towards parity in both secondary and primary completion rates with the exception of large regions within central and western sub-Saharan Africa (Extended Data Fig. 2b, d).

## Inequalities within and between countries

**Fig. 2:**
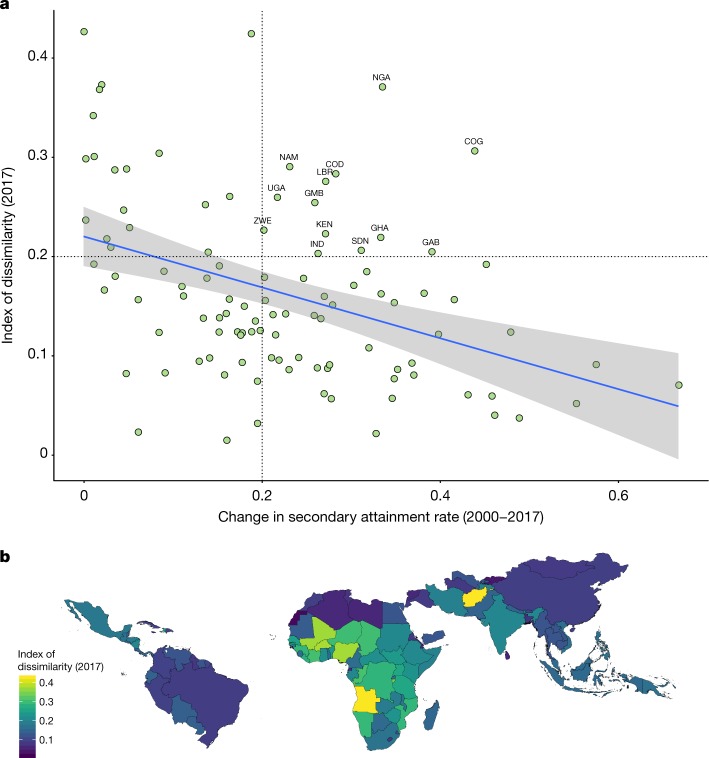
National progress in secondary attainment rates for women aged 20–24 years compared with the national index of dissimilarity in 2017. **a**, Change in secondary attainment rates for women age 20–24 years between 2000 and 2017 compared with the national index of dissimilarity in 2017 (simple linear regression lines are included). **b**, Map of the national index of dissimilarity in 2017. Maps were produced using ArcGIS Desktop 10.6.

The subnational estimates of attainment presented here enable a closer examination of within-country inequality and associated trends over time. Figure 2 plots the national change in secondary attainment rates for women aged 20–24 years with the index of dissimilarity across second administrative-level units in 2017. The index of dissimilarity is an intuitive measure of geographical inequality that can be interpreted as the percentage of women with secondary attainment that would have to move in order to equalize secondary rates across all subnational districts. We estimated that countries that experienced more national progress over the period tended to be more spatially equal in 2017. However, the top-right quadrant of the graph highlights several countries that experienced substantial national progress yet remain some of the most geographically unequal countries today.

**Fig. 3:**
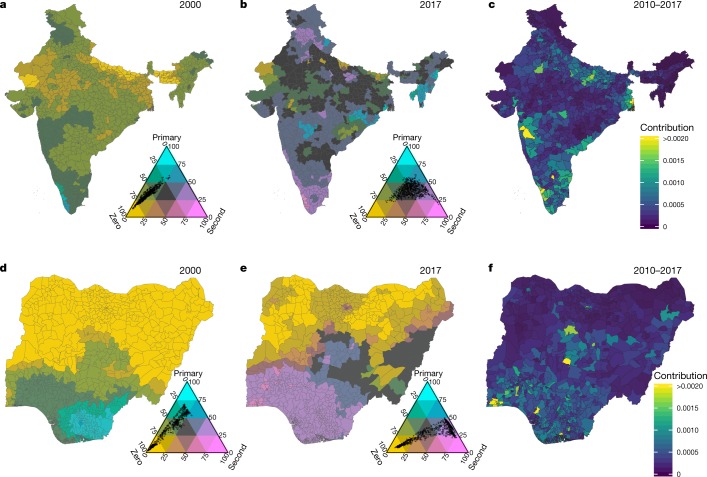
Attainment rates and contributions to national change in secondary rates for women aged 20–24 years in India and Nigeria, 2000–2017. **a**, **b**, Attainment rates for women aged 20–24 years in 2000 (**a**) and 2017 (**b**) at the second administrative level in India. **c**, Additive contributions of changes in the attainment rates at the second administrative level to change in the rate at the national level between 2000 and 2017 in India. **d**, **e**, Attainment rates for women aged 20–24 years in 2000 and 2017 at the second administrative level in Nigeria. **f**, Additive contributions of changes in the attainment rates at the second administrative level to change in the rate at the national level between 2000 and 2017 in Nigeria. On all ternary maps, the ‘Zero’ category includes all individuals with either no schooling or some primary schooling without completion. Maps were produced using ArcGIS Desktop 10.6.

We further examined national progress between 2000 and 2017 in two such countries, India and Nigeria, where rates of secondary attainment increased from 10.9% (8.5–12.5%) to 37.2% (33.6–41.1%) and from 11.5% (6.2–18.3%) to 45.0% (37.0–52.5%), respectively (Fig. 3). The geographical distribution between two cohorts—women aged 20–24 years in 2000 and 2017—was analysed by examining all proportions simultaneously (Fig. 3a, b). We estimate that there has been a massive shift towards primary and secondary completion coupled with greater geographical variability in completion rates (that is, spread of the dots that represent subnational units in the legend). The majority of the 2017 cohort living in the northwest and northeast of India never completed secondary school. Urban centres in the south, such as Bangalore and Mumbai, have seen considerable progress compared with more rural regions. In Nigeria, we estimate substantial national improvement; however, the country remained one of the most spatially unequal in 2017 (Fig. 3d, e). The more-urban south, particularly around Lagos, experienced much faster progress than the more-rural north. The implications of the population distribution were explored by decomposing the improvement in the national rate of secondary completion since 2000 for each country into the additive contributions of rate changes at the second administrative level (Fig. 3c, f). This demonstrates that national progress was largely driven by improvements in populous urban regions (particularly Maharashtra, India, and Lagos, Nigeria), underscoring the importance of how subnational progress (or lack thereof) contributes differentially to narratives surrounding national change.

## Discussion and limitations

We have built on previous modelling efforts that focused on the geographical distribution of average education^14^ by extending our estimation to the distribution of attainment, highlighting not only average attainment but also the proportions of individuals who completed key levels of schooling that are central to policy efforts. As we demonstrate, throughout much of the world women lag behind their male counterparts, and there is significant heterogeneity across subnational regions. Countries such as South Africa, Peru, and Colombia have seen tremendous improvement since 2000 in the proportion of the young adult population who have completed secondary school. As this trend continues, it will be important to focus not only on attainment but also on quality of education. However, many young women across the world still faced obstacles to attaining even a basic level of education in 2017 (Extended Data Fig. 3). This represents a missed opportunity for the global health community to focus on a well-studied determinant of maternal, newborn, and child health. Even with only marginal returns to health in the short term, studies suggest that, on average, communities will also see increased human capital, social mobility, and less engagement in child marriage or early childbearing^20,21^.

Children and adolescents do not complete formal schooling for many reasons. Many factors differentially affect girls, such as cost, late or no school enrolment, forced withdrawal of married adolescents, and the social influence of family members concerning the traditional roles of girls and women^4,20,22,23^. A critical step is acknowledging that commercialization in the area of education typically leads to higher inequity^24^. Treating public education as a societal good by increasing access, particularly in underserved rural communities, reduces inequality. Identifying areas that are stagnating or worsening, particularly in the realm of basic education for young women across the world, is an important first step to targeted, long-term reform efforts that will ultimately have widespread benefits for equity in health and development.

Many recent international calls to improve the social determinants of health have stated that measurement of inequity within countries is critical to understanding and tracking the problem, noting that geography is an increasingly important dimension of inequity^24–26^. Where people are born greatly determines their life chances, and continuing to consider development and human capital formation on a national level is insufficient^24^. The goal of this analysis is to identify local areas that may have experienced negligible improvements, but further rigorous research is required to contextualize these patterns within the unique mix of structural obstacles that each community faces. There are many indirect costs for attending school and each disadvantaged area that we identify in our analysis may experience them in different ways. These include the demand for children to work, the opportunity or monetary costs of attending school, distance to school, lack of compulsory education requirements, high fees for attendance, political instability, and many other forces. Overcoming these obstacles to improve educational attainment alone will not necessarily result in a more-educated and healthy population for each country as highly educated individuals may be more likely to emigrate, resulting in ‘brain drain’. This is especially true for countries that have been economically crippled over the past two decades and may lack the economic capacity to absorb a more highly educated labour force. Opening access to education will need to be coupled with economic reforms, both internationally and domestically, if countries are to fully experience dividends in human capital and health.

Over the next decade of the SDG agenda, it will be important to maintain the progress that has been made to reprioritise investment in education systems. There remains an alarming lack of distributional accountability in aid, especially to basic education, for which most funding is not going to the countries that need it most^19^. Connections between educational attainment and health offer promising opportunities for co-financing initiatives. For example, USAID recently invested US$90 million in HIV funding to the construction of secondary schools in sub-Saharan Africa. Global health leaders have noted the need to invest in precise data systems and eliminate data gaps to effectively target resources, develop equitable policy, and track accountability^7^. Our analysis provides a robust evidence base for such decision-making and advocacy. Decades of research on the effect of basic education on maternal, newborn, and child health positions this issue squarely in the purview of the global health agenda. It is crucial for the global health community to invest in long-term, sustainable improvement in the underlying distribution of human capital, as this is the only way to truly influence health equity across generations.

## Methods

### Overview

Using a Bayesian model-based geostatistical framework and synthesizing geolocated data from 528 household and census datasets, this analysis provides subnational estimates of mean numbers years of education and the proportion of the population who attained key levels of education for women of reproductive age (15–49 years), women aged 20–24 years, and equivalent male age bins between 2000 and 2017 in 105 countries across all low- and middle-income countries (LMICs). Countries were selected for inclusion in this analysis using the socio-demographic index (SDI) published in the Global Burden of Disease (GBD) study^27^. The SDI is a measure of development that combines education, fertility, and poverty. Countries in the middle, lower-middle, or low SDI quintiles were included, with several exceptions. Albania, Bosnia, and Moldova were excluded despite middle SDI status due to geographical discontinuity with other included countries and lack of available survey data. Libya, Malaysia, Panama, and Turkmenistan were included despite higher-middle SDI status to create better geographical continuity. We did not analyse American Samoa, Federated States of Micronesia, Fiji, Kiribati, Marshall Islands, Samoa, Solomon Islands, or Tonga, where no available survey data could be sourced. Analytical steps are described below, and additional details can be found in the Supplementary Information.

### Data

We compiled a database of survey and census datasets that contained geocoding of subnational administrative boundaries or GPS coordinates for sampled clusters. These included datasets from 528 sources (see Supplementary Table 2). These sources comprised at least one data source for all but two countries on our list of LMICs: Western Sahara and French Guiana. We chose to exclude these two countries from our analysis; 42 of 105 included countries have only subnational administrative level data. We extracted demographic, education, and sample design variables. The coding of educational attainment varies across survey families. In some surveys, the precise number of years of attainment is not provided, with attainment instead aggregated into categories such as ‘primary completion’ or ‘secondary completion’. In such cases, individuals who report ‘primary completion’ may have gone on to complete some portion of secondary education, but these additional years of education are not captured in the underlying dataset. Previous efforts to examine trends in mean years of education have either assumed that no additional years of education were completed (that is, primary education only) or have used the midpoint between primary and secondary education as a proxy^28^. Trends in the single-year data, however, demonstrate that such assumptions introduce bias in the estimation of attainment trends over time and space, as differences in actual drop-out patterns or binning schema can lead to biased mean estimates^29^.

For this analysis, we used a recently developed method that selects a training subset of similar surveys across time and space to estimate the unobserved single-year distribution of binned datasets^29^. In comprehensive tests of cross-validation that leveraged data for which the single-year distributions are observed, this algorithmic approach significantly reduces bias in summary statistics estimated from datasets with binned coding schemes compared to alternatives such as the standard-duration method^28^. The years in all coding schemes were mapped to the country- and year-specific references in the UNESCO International Standard Classification of Education (ISCED) for comparability^30^. We used a top coding of 18 years on all data; this is a common threshold in many surveys that have a cap and it is reasonable to assume that the importance of education for health outcomes (and other related SDGs) greatly diminishes after what is the equivalent of 2 to 3 years of graduate education in most systems.

Data were aggregated to mean years of education attained and the proportions achieving key levels of education. The levels chosen were proportion with zero years, proportion with less than primary school (1–5 years of education), proportion with at least primary school (6–11 years of education), and proportion achieving secondary school or higher (12 or more years of education). A subset of the data for a smaller age bin (20–24 years) was also examined to more closely track temporal shifts. Equivalent age bins were aggregated for both women and men to examine disparities in mean years of attainment by sex. Where GPS coordinates were available, data were aggregated to a specific latitude and longitude assuming a simple-random sample, as the cluster is the primary sampling unit for the stratified design survey families, such as the Demographic and Health Survey (DHS) and Multiple Indicator Cluster Survey (MICS). Where only geographical information was available at the level of administrative units, data were aggregated with appropriate weighting according to their sample design. Design effects were estimated using a package for analysing complex survey data in R^31^.

### Spatial covariates

To leverage strength from locations with observations to the entire spatiotemporal domain, we compiled several 5 × 5-km^2^ raster layers of possible socioeconomic and environmental correlates of education (Supplementary Table 5 and Supplementary Fig. 6). Acquisition of temporally dynamic datasets, where possible, was prioritized to best match our observations and thus predict the changing dynamics of educational attainment. We included nine covariates indexed at the 5 × 5-km^2^ level: access to roads, nighttime lights^tv^, population^tv^, growing season, aridity^tv^, elevation, urbanicity^tv^, irrigation, and year^tv^ (tv, time-varying covariates). More details, including plots of all covariates, can be found in the Supplementary Information.

Our primary goal is to provide educational attainment predictions across LMICs at a high (local) resolution, and our methods provide the best out-of-sample predictive performance at the expense of inferential understanding. To select covariates and capture possible nonlinear effects and complex interactions between them, an ensemble covariate modelling method was implemented^32^. For each region, three submodels were fitted to our outcomes using all of our covariate data: generalized additive models, boosted regression trees, and lasso regression. Each submodel was fit using fivefold cross-validation to avoid overfitting and the out-of-sample predictions from across the five folds were compiled into a single comprehensive set of predictions from that model. Additionally, the same submodels were also run using 100% of the data and a full set of in-sample predictions were created. The five sets of out-of-sample submodel predictions were fed into the full geostatistical model as predictors when performing the model fit. The in-sample predictions from the submodels were used as the covariates when generating predictions using the fitted full geostatistical model. This methodology maximizes out-of-sample predictive performance at the expense of the ability to provide statistical inference on the relationships between the predictors and the outcome. A recent study has shown that this ensemble approach can improve predictive validity by up to 25% over an individual model^32^. More details on this approach can be found in the Supplementary Information.

The primary goal of using the stacking procedure in our analyses was to maximize the predictive power of the raster covariates by capturing the nonlinear effects and complex interactions between covariates to optimize the model performance. It has previously been suggested^32^ that the primary purpose of the submodel predictions is to improve the mean function of the Gaussian process. Although we have determined a way to include the uncertainty from two of our submodels (lasso regression and generalized additive models (GAM)), we have not determined a way to include uncertainty from the boosted regression tree (BRT) submodel into our final estimates. Whereas GAM and lasso regression seek to fit a single model that best describes the relationship between response variable and some set of predictors, BRT method fits a large number of relatively simple models for which the predictions are then combined to give robust estimates of the response. Although this feature of the BRT model makes it a powerful tool for analysing complex data, quantifying the relative uncertainty contributed by each simple model as well as uncertainty from the complex interactions of the predictor variables is challenging^33,34^. It is worth noting, however, that our out-of-sample validation indicates that the 95% coverage is fairly accurate (for example, closely ranges around 95%) as shown in the figures and table of Supplementary Information section 4.3.2. This indicates that we are not misrepresenting the uncertainty in our final estimates.

### Analysis

#### Geostatistical model

**Extended Data Fig. 1:**
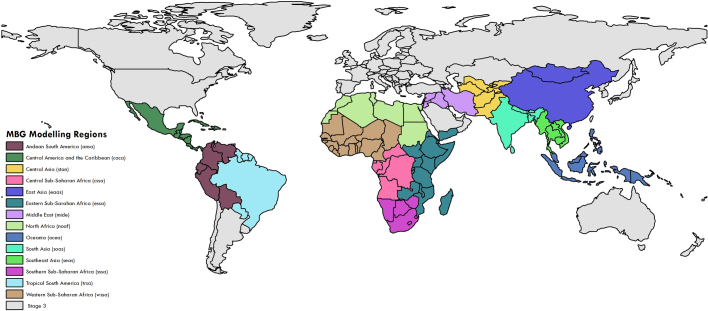
Modelling regions based on geographical and SDI regions from the GBD. Modelling regions were defined as follows. Andean South America, Central America and the Caribbean, central sub-Saharan Africa, East Asia, eastern sub-Saharan Africa, Middle East, North Africa, Oceania, Southeast Asia, South Asia, southern sub-Saharan Africa, Central Asia, Tropical South America, and western sub-Saharan Africa. Regions in grey were not included in our models due to high-middle and high SDIs^27^. The map was produced using ArcGIS Desktop 10.6.

**Extended Data Fig. 2:**
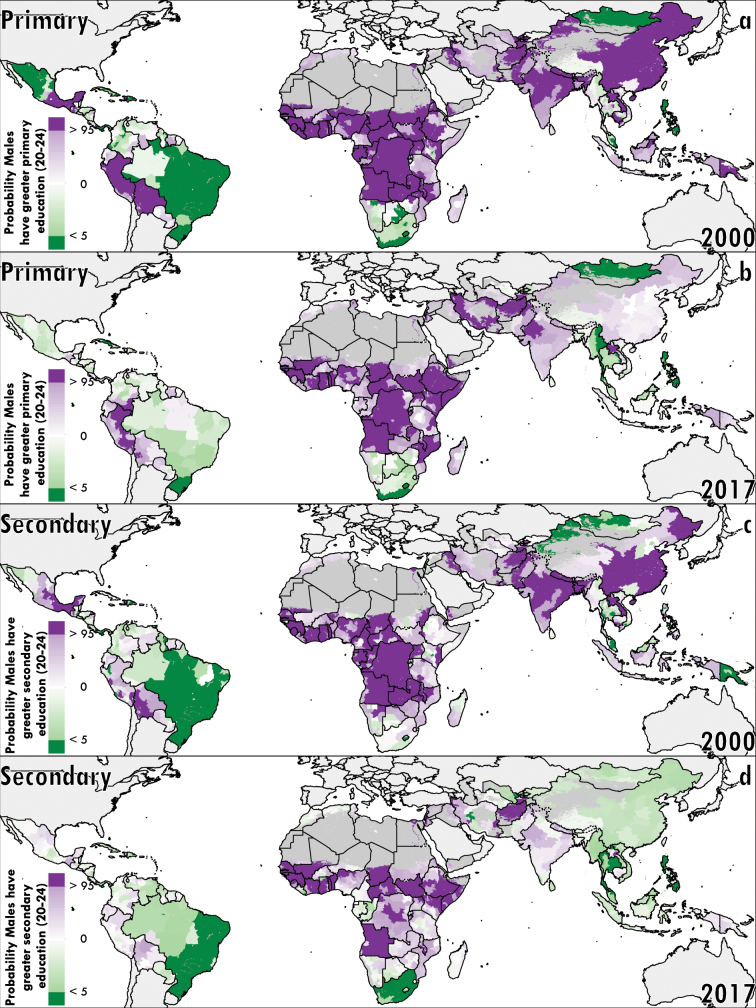
Probability that the ratio of men to women aged 20–24 years who attained primary and secondary education is >1 in 2000 and 2017. **a**–**d**, Probability that ratio is >1 (for example, men complete at a higher rate than women) for attaining primary education (**a**, **b**) and secondary education (**c**, **d**), aggregated to first administrative-level units in 2000 (**a**, **c**) and 2017 (**b**, **d**). Maps were produced using ArcGIS Desktop 10.6.

**Extended Data Fig. 3:**
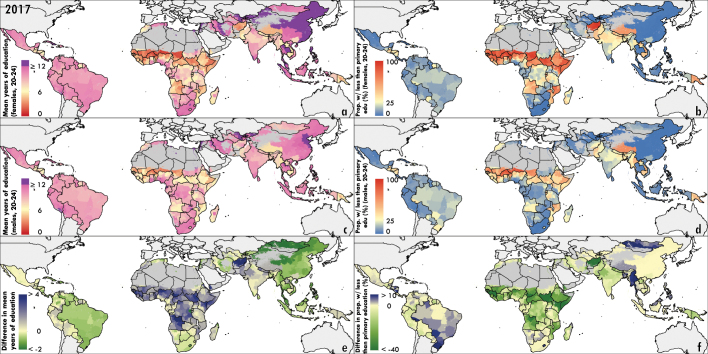
Average educational attainment and proportion with no primary school at the first administrative level and absolute difference between women and men aged 20–24 years. **a**–**d**, Average educational attainment for women (**a**) and men (**c**) and proportion with no primary school for women (**b**) and men (**d**) aged 20–24 years in 2017. **e**, **f**, The absolute difference in average educational attainment between men and women aged 20–24 years in 2017 (**e**) and proportion of individuals with no primary school education (**f**). Maps reflect administrative boundaries, land cover, lakes and population; grey-coloured grid cells were classified as ‘barren or sparsely vegetated’ and had fewer than ten people per 1 × 1-km^2^ grid cell^49,58–60,62^, or were not included in these analyses. Interactive visualization tools are available at https://vizhub.healthdata.org/lbd/education. Maps were produced using ArcGIS Desktop 10.6.

Gaussian and binomial data are modelled within a Bayesian hierarchical modelling framework using a spatially and temporally explicit hierarchical generalized linear regression model to fit the mean number years of education attainment and the proportion of the population who achieved key bins of school in 14 regions across all LMICs as defined in the GBD study (Extended Data Fig. 1). This means we fit 14 independent models for each indicator (for example, the proportion of women with zero years of schooling). GBD study design sought to create regions on the basis of three primary criteria: epidemiological homogeneity, sociodemographic similarity, and geographical contiguity^27^. Fitting our models by these regions has the advantage of allowing for some non-stationarity and non-isotropy in the spatial error term, compared to if we modelled one spatiotemporal random-effect structure over the entire modelling region of all LMICs.

For each Gaussian indicator, we modelled the mean number of years of attainment in each survey cluster, *d*. Survey clusters are precisely located by their GPS coordinates and year of observation, which we map to a spatial raster location *i* at time *t*. We model the mean number of years of attainment as Gaussian data given fixed precision *τ* and a scaling parameter *s*_*d*_ (defined by the sample size in the observed cluster). As we may have observed multiple data clusters within a given location *i* at time *t*, we refer to the mean attainment, *μ*, within a given cluster *d* by its indexed location *i*, and time *t* as *μ*_*i*(*d*),*t*(*d*)_.

$${{\rm{edu}}}_{d}|{\mu }_{i(d),t(d)},{s}_{d},\tau \, \sim \,{\rm{Normal}}({\mu }_{i(d),t(d)},\tau {s}_{d})\,\forall \,{\rm{observed}}\,{\rm{clusters}}\,d$$

$${\mu }_{i,t}=\,{\beta }_{0}+{{\bf{X}}}_{i,t}{\boldsymbol{\beta }}+{Z}_{i,t}+{{\epsilon }}_{{\rm{ctr}}(i)}+{{\epsilon }}_{i,t}\,\forall \,i\in {\rm{spatial}}\,{\rm{domain}}\,\forall \,t\in {\rm{time}}\,{\rm{domain}}$$

For each binomial indicator, we modelled the number of individuals at a given attainment level in each survey cluster, *d*. We observed the number of individuals reporting a given attainment level as binomial count data *C*_*d*_ among an observed sample size *N*_*d*_. As we may have observed multiple data clusters within a given location *i* at time *t*, we refer to the probability of attaining that level, *p*, within a given cluster *d* by its indexed location *i* and time *t* as *p*_*i*(*d*),*t*(*d*)_.

$${C}_{d}|{p}_{i(d),t(d)},\,{N}_{d}\sim {\rm{Binomial}}({p}_{i(d),t(d)},\,{N}_{d})\,\forall \,{\rm{observed}}\,{\rm{clusters}}\,d$$

$${\rm{logit}}({p}_{i,t})=\,{\beta }_{0}+{{\bf{X}}}_{i,t}{\boldsymbol{\beta }}+{Z}_{i,t}+{{\epsilon }}_{{\rm{ctr}}(i)}+{{\epsilon }}_{i,t}\,\forall \,i\in {\rm{spatial}}\,{\rm{domain}}\,\forall \,t\in {\rm{time}}\,{\rm{domain}}$$

We used a continuation-ratio modelling approach to account for the ordinal data structure of the binomial indicators^35^. To do this, the proportion of the population with zero years of education was modelled using a binomial model. The proportion with less than primary education was modelled as those with less than primary education of those that have more than zero years of education. The same method followed for the proportion of population completing primary education. The proportion achieving secondary school or higher was estimated as the complement of the sum of the three binomial models.

The remaining parameter specification was consistent between all indicators in both binomial and Gaussian models:

$$\mathop{\sum }\limits_{h=1}^{3}{\beta }_{h}\,=1$$

$${{\epsilon }}_{{\rm{ctr}}}\sim {\rm{iid}}\,{\rm{Normal}}(0,\,{\gamma }^{2})$$

$${{\epsilon }}_{i,t}\sim {\rm{iid}}\,{\rm{Normal}}(0,\,{\sigma }^{2})$$

$${\bf{Z}}\sim {\rm{GP}}(0,{\Sigma }^{{\rm{space}}}\otimes {\Sigma }^{{\rm{time}}})$$

$${\Sigma }^{{\rm{space}}}=\,\frac{{\omega }^{2}}{\varGamma (\nu ){2}^{v-1}}\times {(\kappa D)}^{\nu }\times {{\rm K}}_{\nu }(\kappa D)$$

$${\Sigma }_{j,\,k}^{{\rm{time}}\,}={\rho }^{|k-j|}$$

For indices *d*, *i*, and *t*, *(index) is the value of * at that index. The probabilities *p*_*i*,*t*_ represent both the annual proportions at the space–time location and the probability that an individual had that level of attainment given that they lived at that particular location. The annual probability *p*_*i*,*t*_ of each indicator (or *μ*_*i*,*t*_ for the mean indicators) was modelled as a linear combination of the three submodels (GAM, BRT, and lasso regression), rasterized covariate values *X*_*i*,*t*_, a correlated spatiotemporal error term *Z*_*i*,*t*_, country random effects ${{\epsilon }}_{{\rm{ctr}}(i)}$ with one unstructured country random effect fit for each country in the modelling region and all sharing a common variance parameter, *γ*^2^, and an independent nugget effect $\,{{\epsilon }}_{i,t}$ with variance parameter *σ*^2^. Coefficients *β*_*h*_ in the three submodels *h* = 1, 2, 3 represent their respective predictive weighting in the mean logit link, while the joint error term *Z*_*i*,*t*_ accounts for residual spatiotemporal autocorrelation between individual data points that remains after accounting for the predictive effect of the submodel covariates, the country-level random effect ${{\epsilon }}_{{\rm{ctr}}(i)}$, and the nugget independent error term, $\,{{\epsilon }}_{i,t}$. The purpose of the country-level random effect is to capture spatially unstructured, unobserved country-specific variables, as there are often sharp discontinuities in educational attainment between adjacent countries due to systematic differences in governance, infrastructure, and social policies.

The residuals *Z*_*i*,*t*_ are modelled as a three-dimensional Gaussian process (GP) in space–time centred at zero and with a covariance matrix constructed from a Kronecker product of spatial and temporal covariance kernels. The spatial covariance Σ^space^ is modelled using an isotropic and stationary Matérn function^36^, and temporal covariance Σ^time^ as an annual autoregressive (AR1) function over the 18 years represented in the model. In the stationary Matérn function, Γ is the Gamma function, *K*_*v*_ is the modified Bessel function of order *v* > 0, *κ* > 0 is a scaling parameter, *D* denotes the Euclidean distance, and *ω*^2^ is the marginal variance. The scaling parameter, *κ*, is defined to be $\kappa =\sqrt{8v}/\delta$ where *δ* is a range parameter (which is about the distance for which the covariance function approaches 0.1) and *v* is a scaling constant, which is set to 2 rather than fit from the data^37,38^. This parameter is difficult to reliably fit, as documented by many other analyses^37,39,40^ that set this parameter to 2. The number of rows and the number of columns of the spatial Matérn covariance matrix are equal to the number of spatial mesh points for a given modelling region. In the AR1 function, *ρ* is the autocorrelation function (ACF), and *k* and *j* are points in the time series where |*k* − *j*| defines the lag. The number of rows and the number of columns of the AR1 covariance matrix are equal to the number of temporal mesh points (18). The number of rows and the number of columns of the space–time covariance matrix, Σ^space^ ⊗ Σ^time^, for a given modelling region are equal to: the number of spatial mesh points × the number of temporal mesh points.

This approach leveraged the residual correlation structure of the data to more accurately predict estimates for locations with no data, while also propagating the dependence in the data through to uncertainty estimates^41^. The posterior distributions were fit using computationally efficient and accurate approximations in R-integrated nested Laplace approximation (INLA) with the stochastic partial differential equations (SPDE) approximation to the Gaussian process residuals using R project version 3.5.1^42–45^. The SPDE approach using INLA has been demonstrated elsewhere, including the estimation of health indicators, particulate air matter, and population age structure^10,11,46,47^. Uncertainty intervals were generated from 1,000 draws (that is, statistically plausible candidate maps)^48^ created from the posterior-estimated distributions of modelled parameters. Additional details regarding model and estimation processes can be found in the Supplementary Information.

To transform grid cell-level estimates into a range of information that is useful to a wide constituency of potential users, these estimates were aggregated from the 1,000 candidate maps up to district, provincial, and national levels using 5 × 5-km^2^ population data^49^. This aggregation also enabled the calibration of estimates to national GBD estimates for 2000–2017. This was achieved by calculating the ratio of the posterior mean national-level estimate from each candidate map draw in the analysis to the posterior mean national estimates from GBD, and then multiplying each cell in the posterior sample by this ratio. National-level estimates from this analysis with GBD estimates can be found in Supplementary Table 44.

To illustrate how subnational progress has contributed differentially to national progress (Fig. 3), we decomposed the improvement in the national rate of secondary completion since 2000 for each country into the additive contributions of rate changes at the second administrative level, where *C* is the national secondary rate change, *N* is the total number of second-level administrative units, *c*_*i*_ is the population proportion in administrative unit *i*, and *r*_*i*_ is the rate of secondary attainment in administrative unit *i*.

$$C=\,\mathop{\sum }\limits_{i=1}^{N}({c}_{i,2017}{r}_{i,2017})-({c}_{i,2000}{r}_{i,2000})$$

Although the model can predict at all locations covered by available raster covariates, all final model outputs for which land cover was classified as ‘barren or sparsely vegetated’ were masked, on the basis of the most recently available Moderate Resolution Imaging Spectroradiometer (MODIS) satellite data (2013), as well as areas in which the total population density was less than 10 individuals per 1 × 1-km^2^ pixel in 2015^50^. This step has led to improved understanding when communicating with data specialists and policy-makers.

#### Model validation

Models were validated using source-stratified fivefold cross-validation. To offer a more stringent analysis by respecting some of the source and spatial correlation in the data, holdout sets were created by combining sets of data sources (for example, entire survey- or census-years). Model performance was summarized by the bias (mean error), total variance (root-mean-square error) and 95% data coverage within prediction intervals, and the correlation between observed data and predictions. All validation metrics were calculated on the predictions from the fivefold cross-validation. Where possible, estimates from these models were compared against other existing estimates. Furthermore, measures of spatial and temporal autocorrelation pre- and post-modelling were examined to verify correct recognition, fitting, and accounting for the complex spatiotemporal correlation structure in the data. All validation procedures and corresponding results are provided in the Supplementary Information.

#### Limitations

Our analysis is not without several important limitations. First, almost all data collection tools conflate gender and sex and we therefore do not capture the full distribution of sex or gender separately in our data. We refer throughout to the measurement of ‘gender (in)equality’, following the usage in SDG 5. Second, it is extremely difficult to quantify quality of education on this scale in a comparable way. Quality is ultimately a large part of the SDG agenda and of utmost importance to achieving equity in opportunity for social mobility. However, many studies across diverse low- and middle-income settings have linked attainment, even very low levels, to measurable improvement in maternal and child health^17^. As our analysis highlights with the proportional indicators, there are still many subnational regions across the world where large proportions do not complete primary school. A third limitation is that we are unable to measure or account for migration. A concept note released from the forthcoming Global Education Monitoring Report 2019 focuses on how migration and displacement affects schooling^51^. Our estimates of the modelled outcome, educational attainment for a particular space–time–age–sex, are demonstrated to be statistically unbiased (Supplementary Information section 4.3); however, interpretation of any change in attainment as a change in the underlying education system could potentially be biased by the effects of migration. It is possible that geographical disparities reflect changes in population composition rather than changes in the underlying infrastructure or education system. Pathways for this change are complex and may be voluntary. Those who manage to receive an education in a low-attainment area may have an increased ability to migrate and choose to do so. This change may also be involuntary, particularly in politically unstable areas where displacement may make geographical changes over time difficult to estimate. A shifting population composition is a general limitation of many longitudinal ecological analyses, but the spatially granular nature of the analyses used here may be more sensitive to the effects of mobile populations.

Our analysis is purely predictive but draws heavily in its motivation from a rich history of literature on the role of education in reducing maternal mortality, improving child health, and increasing human capital. Studies have also demonstrated complex relationships between increased education and a myriad of positive health outcomes, such as HIV risk reductions and spillover effects to other household members^52,53^. The vast majority of these studies are associational and recent attempts at causal analyses have provided more-mixed evidence^54–56^. Although causal analyses of education are very difficult and often rely on situational quasi-experiments, associational analyses using the most comprehensive datasets demonstrate consistent support for the connection between education and health^17,57^. Looking towards future analyses, it will be important to study patterns of change in these data and how they overlap with distributions of health. Lastly, our estimates cannot be seen as a replacement for proper data collection systems, especially for tracking contemporaneous change. Our analysis of uncertainty at a high-resolution may be used to inform investment in more robust data systems and collection efforts, especially if the ultimate goal is to measure and track progress in the quality of schooling.

### Reporting summary

Further information on research design is available in the Nature Research Reporting Summary linked to this paper.

## Online content

Any methods, additional references, Nature Research reporting summaries, source data, extended data, supplementary information, acknowledgements, peer review information; details of author contributions and competing interests; and statements of data and code availability are available at 10.1038/s41586-019-1872-1.

## Supplementary information

Supplementary Information .Guidelines for Accurate and Transparent Health Estimates Reporting Compliance Checklist, Supplementary Discussion, Supplementary Text on data, methods, and covariates, Model descriptions, Supplementary References, Supplementary Sections 4.3 and 4.3.2, and Supplementary Tables 2, 3, and 44.

Reporting Summary

## Data Availability

The findings of this study are supported by data that are available in public online repositories, data that are publicly available upon request from the data provider, and data that are not publicly available owing to restrictions by the data provider, which were used under license for the current study, but may be available from the authors upon reasonable request and permission of the data provider. A detailed table of data sources and availability can be found in Supplementary Table 2. Interactive visualization tools are available at https://vizhub.healthdata.org/lbd/education. All maps presented in this study are generated by the authors; no permissions are required for publication. Administrative boundaries were retrieved from the Global Administrative Unit Layers (GAUL) dataset, implemented by FAO within the CountrySTAT and Agricultural Market Information System (AMIS) projects^58^. Land cover was retrieved from the online Data Pool, courtesy of the NASA EOSDIS Land Processes Distributed Active Archive Center (LP DAAC), USGS/Earth Resources Observation and Science (EROS) Center, Sioux Falls, South Dakota^50^. Lakes were retrieved from the Global Lakes and Wetlands Database (GLWD), courtesy of the World Wildlife Fund and the Center for Environmental Systems Research, University of Kassel^59,60^. Populations were retrieved from WorldPop^49,61^. All maps were produced using ArcGIS Desktop 10.6.

## Code availability

Our study follows the Guidelines for Accurate and Transparent Health Estimates Reporting (GATHER). All code used for these analyses is available online at http://ghdx.healthdata.org/record/ihme-data/lmic-education-geospatial-estimates-2000-2017, and at http://github.com/ihmeuw/lbd/tree/edu-lmic-2019.
