# Supplementary material for: Mapping disparities in education across low- and middle-income countries
Source: Nature. 2019 Dec 25;577(7789):235–8. doi: 10.1038/s41586-019-1872-1 (PMC7015853; doi:10.1038/s41586-019-1872-1)
Supplement: Supplementary file 1 — .Guidelines for Accurate and Transparent Health Estimates Reporting Compliance Checklist, Supplementary Discussion, Supplementary Text on data, methods, and covariates, Model descriptions, Supplementary References, Supplementary Sections 4.3 and 4.3.2, and Supplementary Tables 2, 3, and 44. [file 41586_2019_1872_MOESM1_ESM.pdf]

nature

https://doi.org/10.1038/s41586-019-1872-1

Supplementary information

Mapping disparities in education across low- and middle-income countries

In the format provided by the authors and unedited

Local Burden of Disease Educational Attainment Collaborators

Supplementary Information

Table of Contents

Supplementary Figures 2
Supplementary Tables 4
1.0 GATHER compliance 6
2.0 Supplementary discussion 9
2.1 International aid and domestic policy 9
2.2 Relationship between education and health 9
3.0 Supplementary data 10
3.1 Data excluded from model 27
4.0 Supplementary covariates 36
5.0 Supplementary methods 39
5.1 Cluster combination and spatial integration over polygon records 39
5.2 Geostatistical model 39
5.2.1 Model geographies 39
5.2.2 Ensemble covariate modelling 40
5.2.3 Model description 41
5.2.4 Priors 43
5.2.5 Mesh construction 45
5.2.6 Model fitting and estimate generation 45
5.2.7 Model Results 46
5.3 Model validation 90
5.3.1 In-sample metrics 90
5.3.2 Metrics of predictive validity 90
5.3.3 Post estimation calibration to national estimates 149
5.3.4 Aggregation to first- and second-level administrative subdivisions 165
5.3.5 Calculating people with zero years of education 165
6.0 Supplementary references 166
7.0 Authors' contributions 169

Supplementary Figures

Supplementary Figure 1. Education data availability in Africa by type and country for women ages 15–49, 1998–2017 31
Supplementary Figure 2. Education data availability in Latin America and the Caribbean by type and country for women ages 15–49, 1998–2017 32
Supplementary Figure 3. Education data availability in the Middle East and Central Asia by type and country for women ages 15–49, 1998–2017 33
Supplementary Figure 4. Education data availability in East and Southeast Asia by type and country for women ages 15–49, 1998–2017 34
Supplementary Figure 5. Education data availability in South Asia by type and country for women ages 15–49, 1998–2017 35
Supplementary Figure 6. Covariates 38
Supplementary Figure 7. Finite elements mesh 46
Supplementary Figure 8. Zero years, women ages 15–49 posterior means and 95% uncertainty interval, 2017 70
Supplementary Figure 13. 1–5 years, men ages 15–49 posterior means and 95% uncertainty interval, 2017 75
Supplementary Figure 16. 6–11 years, women ages 15–49 posterior means and 95% uncertainty interval, 2017 78
Supplementary Figure 17. 6–11 years, men ages 15–49 posterior means and 95% uncertainty interval, 2017 79
Supplementary Figure 20. 12+ years, women ages 15–49 posterior means and 95% uncertainty interval, 2017 82
Supplementary Figure 21. 12+ years, men ages 15–49 posterior means and 95% uncertainty interval, 2017 83
Supplementary Figure 23. 12+ years, men ages 20–24 posterior means and 95% uncertainty interval, 2017 85
Supplementary Figure 24. Mean years, women ages 15–49 posterior means and 95% uncertainty interval 86
Supplementary Figure 25. Mean years, men ages 15–49 posterior means and 95% uncertainty interval 87
Supplementary Figure 27. Mean years, men ages 20–24 posterior means and 95% uncertainty interval 89
Supplementary Figure 28. Zero years education (women, ages 15–49) admin 0 aggregation 92
Supplementary Figure 29. Zero years education (women, ages 15–49) admin 1 aggregation 93
Supplementary Figure 30. Zero years education (women, ages 15–49) admin 2 aggregation 94
Supplementary Figure 31. Zero years education (men, ages 15–49) admin 0 aggregation 95
Supplementary Figure 32. Zero years education (men, ages 15–49) admin 1 aggregation 96
Supplementary Figure 33. Zero years education (men, ages 15–49) admin 2 aggregation 97
Supplementary Figure 34. Zero years education (women, ages 20–24) admin 0 aggregation 99
Supplementary Figure 35. Zero years education (women, ages 20–24) admin 1 aggregation 100
Supplementary Figure 36. Zero years education (women, ages 20–24) admin 2 aggregation 101
Supplementary Figure 37. Zero years education (men, ages 20–24) admin 0 aggregation 102
Supplementary Figure 38. Zero years education (men, ages 20–24) admin 1 aggregation 103
Supplementary Figure 39. Zero years education (men, ages 20–24) admin 2 aggregation 104
Supplementary Figure 40. 1–5 years education (women, ages 15–49) admin 0 aggregation 106
Supplementary Figure 41. 1–5 years education (women, ages 15–49) admin 1 aggregation 107
Supplementary Figure 42. 1–5 years education (women, ages 15–49) admin 2 aggregation 108
Supplementary Figure 43. 1–5 years education (men, ages 15–49) admin 0 aggregation 109
Supplementary Figure 44. 1–5 years education (men, ages 15–49) admin 1 aggregation 110
Supplementary Figure 45. 1–5 years education (men, ages 15–49) admin 2 aggregation 111
Supplementary Figure 46. 1–5 years education (women, ages 20–24) admin 0 aggregation 113
Supplementary Figure 47. 1–5 years education (women, ages 20–24) admin 1 aggregation 114
Supplementary Figure 48. 1–5 years education (women, ages 20–24) admin 2 aggregation 115

Supplementary Figure 49. 1–5 years education (men, ages 20–24) admin 0 aggregation....116
Supplementary Figure 50. 1–5 years education (men, ages 20–24) admin 1 aggregation....117
Supplementary Figure 51. 1–5 years education (men, ages 20–24) admin 2 aggregation....118
Supplementary Figure 52. 6–11 years education (women, ages 15–49) admin 0 aggregation....120
Supplementary Figure 53. 6–11 years education (women, ages 15–49) admin 1 aggregation....121
Supplementary Figure 54. 6–11 years education (women, ages 15–49) admin 2 aggregation....122
Supplementary Figure 55. 6–11 years education (men, ages 15–49) admin 2 aggregation....123
Supplementary Figure 56. 6–11 years education (men, ages 15–49) admin 2 aggregation....124
Supplementary Figure 57. 6–11 years education (men, ages 15–49) admin 2 aggregation....125
Supplementary Figure 58. 6–11 years education (women, ages 20–24) admin 0 aggregation....127
Supplementary Figure 59. 6–11 years education (women, ages 20–24) admin 1 aggregation....128
Supplementary Figure 60. 6–11 years education (women, ages 20–24) admin 2 aggregation....129
Supplementary Figure 61. 6–11 years education (men, ages 20–24) admin 0 aggregation....130
Supplementary Figure 62. 6–11 years education (men, ages 20–24) admin 1 aggregation....131
Supplementary Figure 63. 6–11 years education (men, ages 20–24) admin 2 aggregation....132
Supplementary Figure 64. Mean years education (women, ages 15–49) admin 0 aggregation....134
Supplementary Figure 65. Mean years education (women, ages 15–49) admin 1 aggregation....135
Supplementary Figure 66. Mean years education (women, ages 15–49) admin 2 aggregation....136
Supplementary Figure 67. Mean years education (men, ages 15–49) admin 0 aggregation....137
Supplementary Figure 68. Mean years education (men, ages 15–49) admin 1 aggregation....138
Supplementary Figure 69. Mean years education (men, ages 15–49) admin 2 aggregation....139
Supplementary Figure 70. Mean years education (women, ages 20–24) admin 0 aggregation....141
Supplementary Figure 71. Mean years education (women, ages 20–24) admin 1 aggregation....142
Supplementary Figure 72. Mean years education (women, ages 20–24) admin 2 aggregation....143
Supplementary Figure 73. Mean years education (men, ages 20–24) admin 0 aggregation....144
Supplementary Figure 74. Mean years education (men, ages 20–24) admin 1 aggregation....145
Supplementary Figure 75. Mean years education (men, ages 20–24) admin 2 aggregation....146
Supplementary Figure 76. Mean years education (women, ages 15–49) comparison to GBD national estimates. 149
Supplementary Figure 77. Mean years education (men, ages 15–49) comparison to GBD national estimates. 150
Supplementary Figure 78. Mean years education (women, ages 20–24) comparison to GBD national estimates. 151
Supplementary Figure 79. Mean years education (men, ages 20–24) comparison to GBD national estimates. 152
Supplementary Figure 80. Zero years of education (women, ages 15–49) comparison to GBD national estimates. 153
Supplementary Figure 81. Zero years of education (men, ages 15–49) comparison to GBD national estimates. 154
Supplementary Figure 82. Zero years of education (women, ages 20–24) comparison to GBD national estimates. 155
Supplementary Figure 83. Zero years of education (men, ages 20–24) comparison to GBD national estimates. 156
Supplementary Figure 84. 1–6 years of education (women, ages 15–49) comparison to GBD national estimates. 157
Supplementary Figure 85. 1–6 years of education (men, ages 15–49) comparison to GBD national estimates. 158
Supplementary Figure 86. 1–6 years of education (women, ages 20–24) comparison to GBD national estimates. 159

Supplementary Figure 87. 1–6 years of education (men, ages 20–24) comparison to GBD national estimates. 160
Supplementary Figure 88. 6–11 years of education (women, ages 15–49) comparison to GBD national estimates. 161
Supplementary Figure 89. 6–11 years of education (men, ages 15–49) comparison to GBD national estimates. 162
Supplementary Figure 90. 6–11 years of education (women, ages 20–24) comparison to GBD national estimates. 163
Supplementary Figure 91. 6–11 years of education (men, ages 20–24) comparison to GBD national estimates. 164

Supplementary Tables

Supplementary Table 1. Guidelines for Accurate and Transparent Health Estimates Reporting (GATHER) checklist 6
Supplementary Table 2. List of countries included in analysis, stratified by Socio-Demographic Index, as published in the 2017 Global Burden of Disease Study 11
Supplementary Table 3. Sources included in analysis 13
Supplementary Table 4. Excluded so
Supplementary Table 1. Guidelines for Accurate and Transparent Health Estimates Reporting (GATHER) checklist....6
Supplementary Table 2. List of countries included in analysis, stratified by Socio-Demographic Index, as published in the 2017 Global Burden of Disease Study....11
Supplementary Table 3. Sources included in analysis....13
Supplementary Table 4. Excluded sources....28
35
Supplementary Table 5. Covariates used in mapping....36
Supplementary Table 6. Spatial hyperparameter priors by region....44
Supplementary Table 7. List of all modeled outcomes and the populations for which they are modelled....47
Supplementary Table 8. Proportion with zero years, women ages 15–49, fitted parameters....48
Supplementary Table 9. Proportion with 1–5 years, women ages 15–49 fitted parameters....49
Supplementary Table 10. Proportion with 6–11 years, women ages 15–49 fitted parameters....51
Supplementary Table 11. Proportion with zero years, men ages 15–49 fitted parameters....52
Supplementary Table 12. Proportion with 1–5 years, men ages 15–49 fitted parameters....53
Supplementary Table 13. Proportion with 6–11 years, men ages 15–49 fitted parameters....55
Supplementary Table 14. Proportion with zero years, women ages 20–24 fitted parameters....56
Supplementary Table 15. Proportion with 1–5 years, women ages 20–24 fitted parameters....58
Supplementary Table 16. Proportion with 6–11 years, women ages 20–24 fitted parameters....59
Supplementary Table 17. Proportion with zero years, men ages 20–24 fitted parameters....60
Supplementary Table 18. Proportion with 1–5 years, men ages 20–24 fitted parameters....62
Supplementary Table 19. Proportion with 6–11 years, men ages 20–24 fitted parameters....63
Supplementary Table 20. Mean years, women ages 15–49 fitted parameters....64
Supplementary Table 21. Mean years, men ages 15–49 fitted parameters....66
Supplementary Table 22. Mean years, women ages 20–24 fitted parameters....67

Supplementary Table 23. Mean years, men ages 20–24 fitted parameters 68
Supplementary Table 24. In sample predictive metrics for zero years of education (women, ages 15–49), aggregated to different administrative levels (admin 0, admin 1, and admin 2)......................................91
Supplementary Table 25. In sample predictive metrics for zero years of education (men, ages 15–49), aggregated to different administrative levels (admin 0, admin 1, and admin2).......................................91
Supplementary Table 26. In sample predictive metrics for zero years of education (women, ages 20-24), aggregated to admin 0, admin 1, and admin 2 98
Supplementary Table 27. In sample predictive metrics for zero years of education (men, ages 20–24), aggregated to admin 0, admin 1, and admin 2 98
Supplementary Table 28. In sample predictive metrics for 1–5 years of education (women, ages 15–49), aggregated to admin 0, admin 1, and admin 2 105
Supplementary Table 29. In sample predictive metrics for 1–5 years of education (men, ages 15–49), aggregated to admin 0, admin 1, and admin 2 105
Supplementary Table 30. In sample predictive metrics for 1–5 years of education (women, ages 20–24), aggregated to admin 0, admin 1, and admin 2 112
Supplementary Table 31. In sample predictive metrics for 1–5 years of education (men, ages 20–24), aggregated to admin 0, admin 1, and admin 2 112
Supplementary Table 32. In sample predictive metrics for 6–11 years of education (women, ages 15–49), aggregated to admin 0, admin 1, and admin 2 119
Supplementary Table 33. In sample predictive metrics for 6–11 years of education (men, ages 15–49), aggregated to admin 0, admin 1, and admin 2 119
Supplementary Table 34. In sample predictive metrics for 6–11 years of education (women, ages 20–24), aggregated to admin 0, admin 1, and admin 2 126
Supplementary Table 35. In sample predictive metrics for 6–11 years of education (men, ages 20–24), aggregated to admin 0, admin 1, and admin 2 126
Supplementary Table 36. In sample predictive metrics for mean years of education (women, ages 15–49), aggregated to admin 0, admin 1, and admin 2 133
Supplementary Table 37. In sample predictive metrics for mean years of education (men, ages 15–49), aggregated to admin 0, admin 1, and admin 2 133
Supplementary Table 38. In sample predictive metrics for mean years of education (women, ages 20–24), aggregated to admin 0, admin 1, and admin 2 140
Supplementary Table 39. In sample predictive metrics for mean years of education (men, ages 20–24), aggregated to admin 0, admin 1, and admin 2 140
Supplementary Table 40. Out of sample predictive metrics for zero years of education (women, ages 15–49), aggregated by fold to admin 0, admin 1, and admin 2 147
Supplementary Table 42. Out of sample predictive metrics for mean years of education (women, ages 15–49), aggregated by fold to admin 0, admin 1, and admin 2 148
Supplementary Table 43. Out of sample predictive metrics for mean years of education (women, ages 20–24), aggregated by fold to admin 0, admin 1, and admin 2 148
Supplementary Table 44. Quartiles for raking factors (RF) from MBG to GBD estimates for all indicators 165

1.0 GATHER compliance

Supplementary Table 1. Guidelines for Accurate and Transparent Health Estimates Reporting (GATHER) checklist

Item #	Checklist item	Reported in
**Objectives and funding**		
**1**	Define the indicator(s), populations (including age, sex, and geographic entities), and time period(s) for which estimates were made.	Main text: Precision public health and education, Main text: Methods
**2**	List the funding sources for the work.	Main text: Acknowledgements
**Data Inputs**		
For all data inputs from multiple sources that are synthesized as part of the study:		
**3**	Describe how the data were identified and how the data were accessed.	Main text: Methods (Analysis)
**4**	Specify the inclusion and exclusion criteria. Identify all ad-hoc exclusions.	Main text: Methods, Supplementary Information: 3.1 Data excluded from model
**5**	Provide information on all included data sources and their main characteristics. For each data source used, report reference information or contact name/institution, population represented, data collection method, year(s) of data collection, sex and age range, diagnostic criteria or measurement method, and sample size, as relevant.	Supplementary Information: Extended Data Table 1
**6**	Identify and describe any categories of input data that have potentially important biases (e.g., based on characteristics listed in item 5).	Main text: Methods
For data inputs that contribute to the analysis but were not synthesized as part of the study:		
**7**	Describe and give sources for any other data inputs.	Supplementary Information: 4.0 Supplementary covariates
For all data inputs:		
**8**	Provide all data inputs in a file format from which data can be efficiently extracted (e.g., a spreadsheet rather than a PDF), including all relevant meta-data listed in item 5. For any data inputs that cannot be shared because of ethical or legal reasons, such as third-party ownership, provide a contact name or the name of the institution that retains the right to the data.	Supplementary Information: Extended Data Table 1
**Data analysis**		

Item #	Checklist item	Reported in
**9**	Provide a conceptual overview of the data analysis method. A diagram may be helpful.	Main text: Methods (Analysis)
**10**	Provide a detailed description of all steps of the analysis, including mathematical formulae. This description should cover, as relevant, data cleaning, data pre-processing, data adjustments and weighting of data sources, and mathematical or statistical model(s).	Main text: Methods, Supplementary Information: 5.2 Geostatistical model
**11**	Describe how candidate models were evaluated and how the final model(s) were selected.	Main text: Methods (Model validation), Supplementary Information: 5.2 Geostatistical model, 5.3 Model validation
**12**	Provide the results of an evaluation of model performance, if done, as well as the results of any relevant sensitivity analysis.	Main text: Methods (Model validation), Supplementary Information: 5.2 Geostatistical model, 5.3 Model validation
**13**	Describe methods for calculating uncertainty of the estimates. State which sources of uncertainty were, and were not, accounted for in the uncertainty analysis.	Main text: Methods (Model validation), Supplementary Information: 5.2 Geostatistical model, 5.3 Model validation
**14**	State how analytic or statistical source code used to generate estimates can be accessed.	Available at http://ghdx.healthdata.org/lbd-data
**Results and Discussion**		
**15**	Provide published estimates in a file format from which data can be efficiently extracted.	Raster files for spatial data and CSVs of estimates available at
**16**	Report a quantitative measure of the uncertainty of the estimates (e.g., uncertainty intervals).	Supplementary Information: 5.2 Geostatistical model, 5.3 Model validation
**17**	Interpret results in light of existing evidence. If updating a previous set of estimates, describe the reasons for changes in estimates.	Main text: Persistent differences in educational attainment, Implications for international goals

Item #	Checklist item	Reported in
**18**	Discuss limitations of the estimates. Include a discussion of any modelling assumptions or data limitations that affect interpretation of the estimates.	Main text: Discussion, limitations and future work, Supplementary Information: 2.4 Limitations of a geographic perspective

2.0 Supplementary discussion

2.1 International aid and domestic policy

After years of deprioritization in the global aid portfolio, in 2016 aid to education reached its highest level since 2002[1]. Despite this shift, only 22% of aid to basic education went to low-income countries in 2016 compared to 36% in 2002 (basic education defined as primary and lower-secondary)[2]. This reflects a concerning and persistent pattern of the distribution of aid not aligning with the distribution of greatest need, even at the national level. Beyond international aid, domestic policy is also a critical tool for expanding access to education, especially at higher levels. In sub-Saharan countries such as Ghana, Burundi, Kenya, Malawi, the United Republic of Tanzania, or Uganda, primary enrollment rates increased dramatically after primary education fees were removed [3]. Most low- and middle-income countries (LMICs) are now providing free primary education, but significant monetary obstacles remain in place for accessing secondary and tertiary education. Enrollment rates drop significantly in secondary school and tertiary due to tuition fees and other education costs (e.g., uniforms, school supplies, travel time) [1,4].

2.2 Relationship between education and health

Policy and intervention research has called for an increased focus on mapping the relationships between the different Sustainable Development Goals (SDGs), such as the effect of educational levels and equity on the goals related to child growth failure and mortality [2,5]. In determining risks to development during childhood, the educational attainment of mothers has been repeatedly identified as a critical factor associated with the quality of child care, survival, and healthy growth trajectories[6–11]. This effect operates on the quality of care sought and attained during pregnancy and post-partum, as well as the quality of care for children through the duration of breastfeeding and healthcare-seeking when children are ill[12,13]. Beyond an individual's education, a comprehensive multi-level study demonstrated that increases in the average educational attainment within communities are associated with improved nutrition and survival for all children in that community[14]. Gender inequality is also recognized for its impact on child undernutrition through women's control of their time, household income, and mental health. Around the world, girls are less likely than boys to attend and finish primary school and gender inequality in education inhibits national economic growth[1,15–17]. This robust body of evidence, along with a pattern of inequitable distribution of aid, suggests that precision estimates of educational attainment and inequality therein will provide a powerful tool for advocates and policymakers to achieve cross-cutting progress towards the SDGs.

The SDGs related to education, child mortality, and child nutrition are all framed around the importance of equity in progress towards targets across dimensions such as geography and gender [18,19]. Indicators such as under-5 mortality and stunting have seen dramatic improvement over the past few decades, but recent geospatial analyses find persistent disadvantage remains subnationally[20,21]. The global health agenda is increasingly focused on precision public health

evidence illustrating the subnational distribution of disease and illness[22,23], but an agenda focused on equity for the future must integrate comparable evidence on the distribution of social determinants of health[20,21,24].

3.0 Supplementary data

We compiled a database of survey and census datasets that contained geocoding of subnational administrative boundaries or GPS coordinates for sampled clusters. These included datasets from 528 sources (see Supplementary Table 2). These sources comprised at least one data source for all but two countries on our list of LMICs: Western Sahara and French Guiana. We chose to exclude these two countries from our analysis. Of the included countries, 42 of 105 have only subnational administrative level data. We extracted demographic, education, and sample design variables. The coding of educational attainment varies across survey families. In some surveys, the precise number of years of attainment is not provided, with attainment instead aggregated into categories such as "primary completion" or "secondary completion". In such cases, individuals who report "primary completion" may have gone on to complete some portion of secondary education, but these additional years of education are not captured in the underlying data set. Previous efforts to examine trends in mean years of education have either assumed that no additional years of education were completed (i.e., primary education only) or have used the midpoint between primary and secondary education as a proxy[25]. Trends in the single-year data, however, demonstrate that such assumptions introduce bias in the estimation of attainment trends over time and space, as differences in actual drop-out patterns or binning schema can lead to biased mean estimates[26].

For this analysis, we employed a novel method that selects a training subset of similar surveys across time and space to estimate the unobserved single-year distribution of binned datasets[26]. In comprehensive tests of cross-validation leveraging data where the single-year distribution is observed, this algorithmic approach significantly reduces bias in summary statistics estimated from datasets with binned coding schemes compared to alternatives such as the standard-duration method. The years in all coding schemes were mapped to the country- and year-specific references in the UNESCO International Standard Classification of Education (ISCED) for comparability[27]. We used a top coding of 18 years on all data; this is a common threshold in many surveys that have a cap and it is reasonable to assume that the importance of education for health outcomes (and other related SDGs) greatly diminishes after what is the equivalent of 2 to 3 years of graduate education in most systems.

Data were aggregated to mean years of education attained and the proportions achieving key levels of education. The levels chosen were proportion with zero years, proportion with less than primary school (1–5 years), proportion with at least primary school (6–11 years), and proportion achieving secondary school or higher (12 or more years). A subset of the data for a smaller age-bin (20–24 years) were also examined to more closely track temporal shifts. Equivalent age-bins were aggregated for both women and men in order to examine disparities in mean years of attainment by sex. Where GPS coordinates were available, data were aggregated to a specific

latitude/longitude assuming a simple-random-sample, as the cluster is the primary sampling unit for the stratified design survey families such as the Demographic and Health Survey (DHS) and Multiple Indicator Cluster Survey (MICS). Where only geography information was available at the level of administrative units, data were aggregated with appropriate weighting according to their sample design. Design effects were estimated using a package for analyzing complex survey data in R[28].

The data sources used to model educational attainment indicators are described in Supplementary Table 3. Supplementary Figures 1–5 display data availability for mean years of education in women 15–49 for 5 broad regions. Data sources are the same between all indictors.

Supplementary Table 2. List of countries included in analysis, stratified by Socio-Demographic Index, as published in the 2017 Global Burden of Disease Study

Low SDI	Low Middle SDI	Middle SDI	High Middle SDI
Afghanistan	Angola	Algeria	China
Bangladesh	Belize	Botswana	Iran
Benin	Bhutan	Brazil	Libya
Burkina Faso	Bolivia	Colombia	Malaysia
Burundi	Cambodia	Costa Rica	
Central African Republic	Cameroon	Cuba	
Chad	Cape Verde	Ecuador	
Comoros	Djibouti	Equatorial Guinea	
Côte d'Ivoire	Dominican Republic	Gabon	
Democratic Republic of the Congo	Egypt	Indonesia	
Eritrea	El Salvador	Jamaica	
Ethiopia	Ghana	Jordan	
Guinea	Guatemala	Mexico	
Guinea-Bissau	Guyana	Mongolia	
Haiti	Honduras	Namibia	
Liberia	India	Panama	
Madagascar	Iraq	Paraguay	
Malawi	Kenya	Peru	
Mali	Kyrgyzstan	Philippines	
Mozambique	Laos	South Africa	
Nepal	Lesotho	Sri Lanka	
Niger	Mauritania	Suriname	
Papua New Guinea	Morocco	Syria	
Rwanda	Myanmar	Thailand	
Senegal	Nicaragua	Trinidad and Tobago	
Sierra Leone	Nigeria	Tunisia	
Somalia	Pakistan	Turkmenistan	
South Sudan	Palestine	Uzbekistan	
Tanzania	Republic of the Congo	Venezuela	
The Gambia	São Tomé and Príncipe	Vietnam	
Togo	Sudan		
Uganda	Swaziland		
Yemen	Tajikistan		
	Timor-Leste		

Zambia
Zimbabwe

Supplementary Table 3. Sources included in analysis

Country	Source Year	Source	Sample Size	Number of geo-positioned clusters	Number of polygons	GHDx ID*	In Last Paper
**Afghanistan**	2006	Country-specific	24,561	708	29	18468†	No
**Afghanistan**	2010	UNICEF MICS	45,191	914	34	56830	No
**Algeria**	2012	UNICEF MICS	84,574	1,909	7	210614	Yes
**Angola**	2001	UNICEF MICS	13,002	1,503	18	687	Yes
**Angola**	2007	DHS MIS	2,953	115	0	672	No
**Angola**	2015	DHS	27,806	625	0	218555	Yes
**Bangladesh**	2001	Country-specific	260,832	208	64	18920	No
**Bangladesh**	2001	Country-specific	5,517,658	466	457	105319	No
**Bangladesh**	2004	DHS	27,972	359	0	18902	No
**Bangladesh**	2006	UNICEF MICS	152,860	207	64	951	No
**Bangladesh**	2007	DHS	27,433	361	0	18913	No
**Bangladesh**	2011	DHS	42,205	600	0	55956	No
**Bangladesh**	2012	UNICEF MICS	118,561	208	64	151086	No
**Bangladesh**	2014	DHS	41,543	178	7	157021	No
**Belize**	2006	UNICEF MICS	3,730	30	6	1089	No
**Belize**	2011	UNICEF MICS	7,468	30	6	76699	No
**Belize**	2013	Country-specific	8,959	30	6	314646	No
**Belize**	2015	UNICEF MICS	9,457	30	6	264910	No
**Benin**	2002	Census	293,300	183	76	367347	No
**Benin**	2013	Census	447,851	183	76	367419	No
**Benin**	2001	DHS	30,417	247	0	18950	Yes
**Benin**	2006	DHS	90,650	0	12	18959	Yes
**Benin**	2011	DHS	88,174	750	0	79839	Yes
**Bhutan**	2005	Country-specific	43,911	47	0	1175†	No
**Bhutan**	2010	UNICEF MICS	33,300	59	20	40028	No
**Bhutan**	2017	Country-specific	6,748	61	20	394652†	No
**Bolivia**	2000	UNICEF MICS	8,718	1,317	3	1289	No
**Bolivia**	2001	Census	396,370	1,355	83	1362	No
**Bolivia**	2012	Country-specific	5,899	7	4	298261	No
**Bolivia**	2016	Country-specific	23,968	1,320	9	323944	No
**Botswana**	2000	UNICEF MICS	13,731	705	14	1404	Yes
**Botswana**	2001	Country-specific	2,946	645	21	22112	No
**Botswana**	2001	Census	86,820	707	22	294205	No
**Botswana**	2004	Country-specific	7,917	396	0	22114	No
**Botswana**	2008	Country-specific	8,932	395	0	22116	No
**Botswana**	2011	Census	109,473	707	22	294235	No
**Botswana**	2013	Country-specific	6,182	292	0	134753	No
**Brazil**	2000	Country-specific	10,749,064	12,841	5,217	1590†	No

Country	Source Year	Source	Sample Size	Number of geo-positioned clusters	Number of polygons	GHDx ID*	In Last Paper
**Brazil**	2000	Census	4,411,453	8,279	1,986	38230	No
**Brazil**	2001	Country-specific	205,512	10,108	27	1477	No
**Brazil**	2002	Country-specific	209,641	10,108	27	1488	No
**Brazil**	2002	Country-specific	92,617	10,108	27	33019	No
**Brazil**	2003	Country-specific	210,168	10,108	27	1489	No
**Brazil**	2004	Country-specific	217,623	10,108	27	1490	No
**Brazil**	2005	Country-specific	222,787	10,108	27	80311	No
**Brazil**	2006	Country-specific	223,835	10,108	27	93528	No
**Brazil**	2006	Country-specific	31,030	10,094	5	141948	No
**Brazil**	2007	Country-specific	217,841	10,108	27	93490	No
**Brazil**	2008	Country-specific	213,243	10,108	27	93487	No
**Brazil**	2009	Country-specific	217,307	10,108	27	93522	No
**Brazil**	2010	Census	5,271,618	11,345	2,424	105322	No
**Brazil**	2011	Country-specific	194,871	10,108	27	106724	No
**Brazil**	2012	Country-specific	195,575	10,108	27	156581	No
**Brazil**	2013	Country-specific	194,816	10,108	27	156583	No
**Brazil**	2014	Country-specific	193,934	10,108	27	238441	No
**Brazil**	2015	Country-specific	189,690	10,108	27	281548	No
**Burkina Faso**	1998	DHS	12,756	208	0	19076	Yes
**Burkina Faso**	2003	DHS	24,633	397	0	19088	Yes
**Burkina Faso**	2006	UNICEF MICS	15,521	195	0	1927	Yes
**Burkina Faso**	2006	Census	369,435	378	236	105403	Yes
**Burkina Faso**	2010	DHS	30,851	541	0	19133	Yes
**Burkina Faso**	2014	DHS MIS	7,968	248	0	188785	No
**Burundi**	2000	UNICEF MICS	9,127	39	17	1994	Yes
**Burundi**	2005	UNICEF MICS	17,948	39	17	1981	Yes
**Burundi**	2010	DHS	18,853	376	0	30431	Yes
**Burundi**	2012	DHS MIS	10,217	200	0	108080	No
**Burundi**	2016	DHS	33,115	552	0	286766	No
**Cambodia**	2000	DHS	30,583	470	0	19156	No
**Cambodia**	2000	Country-specific	14,003	226	24	282112	No
**Cambodia**	2001	Country-specific	13,979	224	23	2003	No
**Cambodia**	2005	DHS	34,841	548	0	19167	No
**Cambodia**	2008	Census	1,235,351	301	164	35329	No
**Cambodia**	2010	DHS	39,768	607	0	30379	No
**Cambodia**	2013	Country-specific	68,211	226	24	164729†	No
**Cambodia**	2014	DHS	37,278	611	0	157024	No
**Cameroon**	1998	DHS	11,735	553	10	19198	Yes
**Cameroon**	2000	UNICEF MICS	11,105	553	10	2053	Yes

Country	Source Year	Source	Sample Size	Number of geo-positioned clusters	Number of polygons	GHDx ID*	In Last Paper
**Cameroon**	2005	Census	777,045	538	209	105800	Yes
**Cameroon**	2006	UNICEF MICS	19,018	753	191	2063	Yes
**Cameroon**	2014	UNICEF MICS	20,174	531	206	244455	No
**Cameroon**	2004	DHS	51,976	465	0	19211	Yes
**Cameroon**	2011	DHS	72,622	578	0	19274	Yes
**Cape Verde**	1998	CDC RHS	6,250	12	12	27511	No
**Central African Republic**	2000	UNICEF MICS	37,252	702	17	2209	Yes
**Central African Republic**	2006	UNICEF MICS	26,753	646	16	2223	Yes
**Central African Republic**	2010	UNICEF MICS	25,324	702	17	82832	Yes
**Chad**	2000	UNICEF MICS	11,572	1,462	15	2244	Yes
**Chad**	2004	DHS	12,476	1,466	9	19315	Yes
**Chad**	2010	UNICEF MICS	36,464	1,453	60	76701	Yes
**Chad**	2014	DHS	36,100	624	0	157025	Yes
**China**	2000	Census	6,651,846	13,159	339	294255	No
**China**	2004	Country-specific	25,747	13,014	31	120994†	No
**China**	2007	WHO	9,109	1,874	8	60405†	No
**China**	2007	Country-specific	38,195	13,014	31	111916†	No
**China**	2010	Country-specific	67,468	13,014	31	103981†	No
**China**	2010	Census	718,432,187	5,611	21	124067†	No
**China**	2010	Country-specific	20,110	6,579	25	283812	No
**China**	2011	Country-specific	14,064	333	98	397821†	No
**China**	2012	Country-specific	12,201	9,683	27	283815	No
**China**	2012	Country-specific	3,724	27	1	298372	No
**China**	2013	Country-specific	21,317	2,129	96	397820†	No
**China**	2014	Country-specific	8,650	8,789	26	283816	No
**China**	2015	Country-specific	21,931	404	132	394660†	No
**Colombia**	2000	DHS	24,498	673	23	19359	No
**Colombia**	2004	DHS	79,166	1,318	33	19324	No
**Colombia**	2005	Census	1,947,305	1,582	487	3029	No
**Colombia**	2007	Country-specific	29,141	1,321	33	3100†	No
**Colombia**	2009	DHS	56,658	2,695	0	21281	No
**Colombia**	2016	DHS	80,291	1,321	33	218566	No
**Comoros**	2000	UNICEF MICS	10,690	4	3	3114	No
**Comoros**	2012	DHS	10,567	242	0	76850	Yes
**Costa Rica**	2000	Census	204,185	104	63	3243	No
**Costa Rica**	2011	UNICEF MICS	11,092	63	7	125596	No
**Costa Rica**	2011	Census	236,795	104	64	227111	No

Country	Source Year	Source	Sample Size	Number of geo-positioned clusters	Number of polygons	GHDx ID*	In Last Paper
**Costa Rica**	2015	Country-specific	19,812	64	6	238431	No
**Côte d'Ivoire**	1998	DHS	6,435	140	0	18531	Yes
**Côte d'Ivoire**	2000	UNICEF MICS	22,889	384	11	26444	Yes
**Côte d'Ivoire**	2005	DHS AIS	11,774	384	11	56148	No
**Côte d'Ivoire**	2006	UNICEF MICS	24,540	282	52	26433	Yes
**Côte d'Ivoire**	2011	DHS	21,963	341	0	18533	Yes
**Côte d'Ivoire**	2016	UNICEF MICS	24,602	384	11	218611	No
**Cuba**	2002	Census	598,939	142	15	3317	No
**Cuba**	2006	UNICEF MICS	16,311	137	4	3310	No
**Cuba**	2010	UNICEF MICS	17,589	143	15	60935	No
**Cuba**	2014	UNICEF MICS	16,451	137	4	169975	No
**Democratic Republic of the Congo**	2001	UNICEF MICS	24,791	2,717	11	3161	Yes
**Democratic Republic of the Congo**	2007	DHS	20,454	293	0	19381	Yes
**Democratic Republic of the Congo**	2010	UNICEF MICS	24,599	357	0	26998	Yes
**Democratic Republic of the Congo**	2013	DHS	34,666	492	0	76878	Yes
**Djibouti**	2006	UNICEF MICS	14,641	36	1	3404	Yes
**Dominican Republic**	1999	DHS	2,817	62	8	19431	No
**Dominican Republic**	2000	UNICEF MICS	8,399	75	30	27069	No
**Dominican Republic**	2002	DHS	51,429	76	32	19444	No
**Dominican Republic**	2002	Census	348,280	91	78	151296	No
**Dominican Republic**	2007	DHS	58,180	1,425	0	19456	No
**Dominican Republic**	2007	Country-specific	3,351	23	9	21198	No
**Dominican Republic**	2010	Census	483,543	97	83	151304	No
**Dominican Republic**	2013	DHS	20,581	524	0	77819	No
**Dominican Republic**	2013	Country-specific	3,881	114	0	165645	No
**Dominican Republic**	2014	UNICEF MICS	58,195	63	10	200697	No
**Ecuador**	1999	CDC RHS	14,095	279	21	27621	No
**Ecuador**	2001	Census	617,412	358	133	3549	No

Country	Source Year	Source	Sample Size	Number of geo-positioned clusters	Number of polygons	GHDx ID*	In Last Paper
**Ecuador**	2004	CDC RHS	10,268	690	0	27630	No
**Ecuador**	2010	Census	725,472	360	140	105801	No
**Egypt**	2000	DHS	46,487	997	0	19511	Yes
**Egypt**	2003	DHS	24,597	924	0	19529	No
**Egypt**	2005	DHS	56,711	1,298	0	19521	Yes
**Egypt**	2006	Census	3,993,131	1,136	316	35578	Yes
**Egypt**	2008	DHS	47,652	1,244	0	26842	Yes
**Egypt**	2013	UNICEF MICS	16,026	27	6	159617	No
**Egypt**	2014	DHS	60,038	1,817	0	154897	Yes
**El Salvador**	1998	CDC RHS	12,634	22	9	27590	No
**El Salvador**	2007	Census	285,613	106	103	56476	No
**El Salvador**	2008	CDC RHS	12,008	32	14	27606	No
**El Salvador**	2014	UNICEF MICS	27,268	32	14	200636	No
**Equatorial Guinea**	2000	UNICEF MICS	8,312	35	7	3655	No
**Eritrea**	2002	DHS	18,464	148	6	19539	Yes
**Ethiopia**	2000	DHS	31,427	533	0	19571	Yes
**Ethiopia**	2004	Country-specific	79,637	1,302	10	34085†	No
**Ethiopia**	2005	DHS	29,557	528	0	19557	Yes
**Ethiopia**	2010	DHS	32,611	571	0	21301	Yes
**Ethiopia**	2014	Country-specific	6,537	1,340	11	153503	No
**Ethiopia**	2014	DHS	18,182	1,339	11	153507†	No
**Ethiopia**	2014	Country-specific	6,537	1,340	11	256175	No
**Ethiopia**	2015	Country-specific	6,753	1,340	11	256176	No
**Ethiopia**	2016	DHS	30,798	622	0	218568	Yes
**Ethiopia**	2007	Census	7,434,086	0	631	227133	Yes
**Gabon**	2000	DHS	13,727	298	40	19579	Yes
**Gabon**	2012	DHS	16,781	331	0	76706	Yes
**Ghana**	1998	DHS	9,909	400	0	19614	Yes
**Ghana**	2000	Census	883,037	329	110	38508	Yes
**Ghana**	2003	DHS	11,308	410	0	19627	Yes
**Ghana**	2006	UNICEF MICS	11,958	286	10	4694	Yes
**Ghana**	2007	Country-specific	9,651	338	338	21173	No
**Ghana**	2007	UNICEF MICS	23,387	129	4	160576†	Yes
**Ghana**	2008	DHS	20,747	404	0	21188	Yes
**Ghana**	2010	UNICEF MICS	2,689	5	0	56241	No
**Ghana**	2010	Census	1,217,176	366	169	151306	Yes
**Ghana**	2011	UNICEF MICS	19,387	741	0	63993	Yes
**Ghana**	2012	Country-specific	25,661	286	10	165101	No
**Ghana**	2014	DHS	18,917	423	0	157027	Yes

Country	Source Year	Source	Sample Size	Number of geo-positioned clusters	Number of polygons	GHDx ID*	In Last Paper
**Ghana**	2016	DHS MIS	4,934	192	0	286788	No
**Ghana**	2017	DHS	49,231	897	0	218572	No
**Guatemala**	1998	DHS	6,021	136	8	19656	No
**Guatemala**	2008	CDC RHS	19,934	142	22	4779	No
**Guatemala**	2015	DHS	49,309	853	0	157031	No
**Guinea**	1999	DHS	13,663	293	0	19670	Yes
**Guinea**	2005	DHS	14,602	291	0	19683	Yes
**Guinea**	2012	DHS	17,351	300	0	69761	Yes
**Guinea**	2016	UNICEF MICS	19,426	294	8	303458	No
**Guinea-Bissau**	2006	UNICEF MICS	18,308	43	9	4818	Yes
**Guinea-Bissau**	2010	UNICEF MICS	8,642	44	9	27215	No
**Guinea-Bissau**	2000	UNICEF MICS	35,060	0	9	4808	Yes
**Guyana**	2000	UNICEF MICS	9,695	164	10	4916	No
**Guyana**	2005	DHS AIS	5,366	123	9	4837	No
**Guyana**	2006	UNICEF MICS	10,194	164	10	4926	No
**Guyana**	2009	DHS	9,819	312	0	21348	No
**Guyana**	2014	UNICEF MICS	10,795	250	0	200598	No
**Haiti**	2000	DHS	20,447	317	0	19708	No
**Haiti**	2003	Census	423,707	54	42	106473	No
**Haiti**	2005	DHS	20,550	332	0	19720	No
**Haiti**	2012	DHS	27,806	437	0	65118	No
**Haiti**	2016	Country-specific	27,683	450	0	218574	No
**Honduras**	2001	CDC RHS	8,362	122	16	27551	No
**Honduras**	2001	Census	284,269	184	111	367563	No
**Honduras**	2005	DHS	42,752	123	16	19728	No
**Honduras**	2011	DHS	48,459	1,127	0	95440	No
**India**	1998	DHS	262,816	3,212	440	19950	No
**India**	1999	Census	310,057	4,096	505	5294	No
**India**	2004	Country-specific	111,951	3,009	363	26919	No
**India**	2004	Census	317,120	4,157	582	152602	No
**India**	2005	DHS	275,927	3,982	29	19963	No
**India**	2007	Country-specific	240,192	3,629	34	80778†	No
**India**	2009	Country-specific	248,714	3,581	574	74309†	No
**India**	2009	Census	252,381	4,177	608	294537	No
**India**	2011	Country-specific	249,185	3,718	585	129718†	No
**India**	2014	Country-specific	177,150	4,226	624	225626†	No
**India**	2015	DHS	1,474,786	28,392	0	157050	No
**Indonesia**	2002	DHS	77,331	1,318	0	20011	No
**Indonesia**	2002	Country-specific	77,149	1,315	0	20040	No

Country	Source Year	Source	Sample Size	Number of geo-positioned clusters	Number of polygons	GHDx ID*	In Last Paper
**Indonesia**	2005	Census	602,727	2,338	413	56573	No
**Indonesia**	2007	DHS	94,683	2,191	33	20021	No
**Indonesia**	2010	Census	12,982,214	2,450	492	56558	No
**Indonesia**	2012	DHS	97,326	2,191	33	76705	No
**Indonesia**	2014	Country-specific	518,893	2,283	444	165186†	No
**Indonesia**	2015	Country-specific	519,674	2,357	448	238332†	No
**Indonesia**	2016	Country-specific	531,330	2,366	449	282087†	No
**Indonesia**	2011	UNICEF MICS	5,324	51	3	104042	No
**Indonesia**	2011	UNICEF MICS	5,653	206	3	104043	No
**Iran**	2006	Census	758,931	2,404	332	39396	No
**Iran**	2011	Census	871,433	2,433	391	294570	No
**Iran**	2016	Country-specific	901,570	2,452	429	299134†	No
**Iraq**	2000	UNICEF MICS	45,695	599	18	7054	No
**Iraq**	2006	UNICEF MICS	55,760	609	18	7028	No
**Iraq**	2006	Country-specific	61,818	536	19	34524	No
**Iraq**	2011	UNICEF MICS	114,043	612	48	76707	No
**Jamaica**	2001	Census	100,631	19	14	39450	No
**Jamaica**	2002	CDC RHS	7,080	19	14	7161†	No
**Jamaica**	2005	UNICEF MICS	7,708	19	14	7149	No
**Jamaica**	2011	UNICEF MICS	9,769	19	14	141336	No
**Jordan**	2002	DHS	23,195	495	0	20073	No
**Jordan**	2007	DHS	42,395	926	0	20083	No
**Jordan**	2009	DHS	38,154	127	12	21206	No
**Jordan**	2012	DHS	41,999	806	0	77517	No
**Kenya**	1998	DHS	16,146	526	0	20132	Yes
**Kenya**	1999	Country-specific	1,343,800	6,590	0	153943	No
**Kenya**	2000	UNICEF MICS	19,071	870	0	7387	Yes
**Kenya**	2003	DHS	17,357	399	0	20145	Yes
**Kenya**	2005	Country-specific	31,005	1,339	0	7375†	No
**Kenya**	2007	Country-specific	14,698	385	0	133219	No
**Kenya**	2008	UNICEF MICS	30,220	590	0	7401	No
**Kenya**	2008	DHS	16,853	397	0	21365	Yes
**Kenya**	2009	Census	1,811,545	725	158	106512	No
**Kenya**	2010	Country-specific	6,442	119	0	157397	No
**Kenya**	2012	Country-specific	10,703	365	0	133304	No
**Kenya**	2014	DHS	66,501	1,584	0	157057	Yes
**Kenya**	2011	UNICEF MICS	11,479	264	0	135416	No
**Kyrgyzstan**	2005	UNICEF MICS	13,082	306	8	7540	No
**Kyrgyzstan**	2009	Census	316,765	334	56	106520	No

Country	Source Year	Source	Sample Size	Number of geo-positioned clusters	Number of polygons	GHDx ID*	In Last Paper
**Kyrgyzstan**	2012	DHS	16,227	314	0	77518	No
**Kyrgyzstan**	2014	UNICEF MICS	13,897	307	9	162283	No
**Laos**	2000	UNICEF MICS	17,403	265	0	7618	No
**Laos**	2006	UNICEF MICS	15,400	300	0	7629	No
**Laos**	2011	UNICEF MICS	47,017	294	17	103973	No
**Laos**	2017	UNICEF MICS	51,897	294	18	375362	No
**Lesotho**	2000	UNICEF MICS	15,029	44	10	7721	No
**Lesotho**	2004	DHS	18,459	381	0	20167	Yes
**Lesotho**	2006	Census	94,211	81	63	367585	No
**Lesotho**	2009	DHS	21,326	395	0	21382	Yes
**Lesotho**	2014	DHS	19,132	399	0	157058	Yes
**Liberia**	2006	DHS	14,274	291	0	20191	Yes
**Liberia**	2008	Census	170,930	121	15	151310	Yes
**Liberia**	2011	DHS MIS	3,938	150	0	56828	No
**Liberia**	2013	DHS	19,878	322	0	77385	Yes
**Liberia**	2016	DHS MIS	4,113	150	0	286768	No
**Libya**	2007	Country-specific	64,609	1,034	22	107340†	No
**Madagascar**	2003	DHS	17,232	732	6	20223	Yes
**Madagascar**	2008	DHS	35,938	585	0	21409	Yes
**Madagascar**	2011	DHS MIS	8,154	266	0	69806	No
**Madagascar**	2016	DHS MIS	10,655	743	22	218580	No
**Madagascar**	2012	UNICEF MICS	15,556	127	0	125594	Yes
**Malawi**	1998	Census	459,872	246	211	40179	Yes
**Malawi**	2000	DHS	27,603	559	0	20252	Yes
**Malawi**	2004	DHS	24,770	520	0	20263	Yes
**Malawi**	2008	Census	626,890	260	225	40186	Yes
**Malawi**	2010	DHS	46,806	827	0	21393	Yes
**Malawi**	2012	DHS MIS	2,906	140	0	77387	No
**Malawi**	2013	UNICEF MICS	50,323	127	31	161662	Yes
**Malawi**	2013	LSMS	9,382	908	0	224223	No
**Malawi**	2015	DHS	50,488	850	0	218581	No
**Malawi**	2017	DHS MIS	3,809	148	0	286769	No
**Malaysia**	2000	Census	222,947	455	133	40785	No
**Mali**	1998	Census	397,809	1,140	243	40235	Yes
**Mali**	2001	DHS	586	1,023	9	20315	Yes
**Mali**	2006	DHS	29,011	405	0	20274	Yes
**Mali**	2009	Census	606,397	1,143	244	151311	Yes
**Mali**	2012	DHS	21,571	413	0	77388	Yes
**Mali**	2015	DHS MIS	7,758	177	0	218587	No

Country	Source Year	Source	Sample Size	Number of geo-positioned clusters	Number of polygons	GHDx ID*	In Last Paper
**Mauritania**	2000	DHS	15,569	771	13	20322	Yes
**Mauritania**	2007	UNICEF MICS	23,792	709	197	8115	No
**Mauritania**	2011	UNICEF MICS	24,852	769	194	152783	Yes
**Mauritania**	2015	UNICEF MICS	26,627	771	13	267343	No
**Mexico**	2000	Country-specific	4,928,871	4,220	2,437	8634	No
**Mexico**	2002	Country-specific	1,078	442	152	8672	No
**Mexico**	2005	Country-specific	103,651	1,508	579	8618	No
**Mexico**	2005	Census	5,361,887	4,178	2,454	43776	No
**Mexico**	2006	Country-specific	71,412	2,492	32	23982	No
**Mexico**	2008	Country-specific	13,010	2,496	32	105286	No
**Mexico**	2009	Country-specific	180,372	2,493	32	24006	No
**Mexico**	2011	Country-specific	96,386	1,725	710	81748	No
**Mexico**	2011	Country-specific	9,655	2,162	26	105291	No
**Mexico**	2015	Census	5,788,035	4,139	2,447	294574	No
**Mexico**	2015	Country-specific	235,574	2,692	1,291	317752	No
**Mexico**	2016	Country-specific	10,664	375	0	316736	No
**Mongolia**	2000	Census	132,808	2,137	63	40796	No
**Mongolia**	2005	UNICEF MICS	15,003	2,113	22	8777	No
**Mongolia**	2008	Country-specific	3,575	3	1	125230	No
**Mongolia**	2010	UNICEF MICS	19,028	1,507	217	76704	No
**Mongolia**	2012	UNICEF MICS	3,639	190	23	189045	No
**Mongolia**	2013	UNICEF MICS	26,401	798	527	150866	No
**Mongolia**	2016	UNICEF MICS	4,221	190	23	335994	No
**Morocco**	2003	DHS	33,874	480	0	20361	Yes
**Morocco**	2004	Census	800,887	942	60	56492	Yes
**Mozambique**	2003	DHS	27,239	959	11	20394	Yes
**Mozambique**	2007	Census	878,780	1,109	337	227143	Yes
**Mozambique**	2011	DHS	25,659	609	0	55975	Yes
**Myanmar**	2000	UNICEF MICS	70,574	841	16	8932	No
**Myanmar**	2009	UNICEF MICS	70,639	841	17	90696†	No
**Myanmar**	2015	DHS	26,487	441	0	157061	No
**Namibia**	2000	DHS	14,459	259	0	20417	Yes
**Namibia**	2006	DHS	19,879	491	0	20428	Yes
**Namibia**	2013	DHS	19,929	549	0	150382	Yes
**Nepal**	2001	DHS	21,415	251	0	20450	No
**Nepal**	2006	DHS	20,203	260	0	20462	No
**Nepal**	2010	UNICEF MICS	14,449	93	24	39999	No
**Nepal**	2011	DHS	23,631	289	0	21240	No
**Nepal**	2014	UNICEF MICS	27,236	510	0	162317	No

Country	Source Year	Source	Sample Size	Number of geo-positioned clusters	Number of polygons	GHDx ID*	In Last Paper
**Nepal**	2016	DHS	23,145	383	0	286782	No
**Nicaragua**	2001	DHS	28,775	224	132	20487	No
**Nicaragua**	2005	Census	262,017	193	93	56520	No
**Nicaragua**	2006	CDC RHS	14,116	226	140	9270	No
**Nicaragua**	2011	Country-specific	46,952	211	133	126952	No
**Niger**	1998	DHS	14,837	268	0	20537	Yes
**Niger**	2000	UNICEF MICS	11,040	1,123	8	9439	Yes
**Niger**	2006	DHS	17,754	1,123	8	20499	Yes
**Niger**	2012	DHS	21,903	1,123	8	74393	Yes
**Nigeria**	1999	UNICEF MICS	36,017	1,094	31	9506	No
**Nigeria**	2003	DHS	15,909	360	0	20567	Yes
**Nigeria**	2007	UNICEF MICS	54,390	1,097	37	9516	Yes
**Nigeria**	2007	Census	37,777	1,097	37	151312	Yes
**Nigeria**	2008	DHS	66,679	886	0	21433	Yes
**Nigeria**	2008	Census	37,202	1,097	37	151313	Yes
**Nigeria**	2009	Census	47,618	1,097	37	151314	Yes
**Nigeria**	2010	DHS MIS	12,546	239	0	30991	No
**Nigeria**	2010	Census	35,260	1,259	626	151315	No
**Nigeria**	2010	Census	34,232	1,097	37	151317	Yes
**Nigeria**	2011	UNICEF MICS	63,833	1,097	37	76703	No
**Nigeria**	2013	DHS	75,764	889	0	77390	Yes
**Nigeria**	2015	DHS MIS	15,673	322	0	218590	No
**Nigeria**	2016	UNICEF MICS	67,553	2,150	0	218613	No
**Pakistan**	1998	Country-specific	2,481	1,162	7	9658†	No
**Pakistan**	1998	Census	5,975,006	1,036	27	41099	No
**Pakistan**	2001	Country-specific	14,368	1,124	6	9720†	No
**Pakistan**	2006	DHS	33,089	956	0	20595	No
**Pakistan**	2011	UNICEF MICS	298,466	337	145	104236	No
**Pakistan**	2012	DHS	46,512	1,109	6	77521	No
**Pakistan**	2014	UNICEF MICS	121,646	297	36	236266	No
**Pakistan**	2017	DHS	40,066	995	123	286783	No
**Palestine**	2004	Country-specific	15,337	9	2	20596†	No
**Palestine**	2010	UNICEF MICS	39,814	18	16	125591	No
**Palestine**	2014	UNICEF MICS	28,366	18	16	161590	No
**Panama**	2000	Census	149,425	105	38	40907	No
**Panama**	2010	Census	179,003	106	39	106529	No
**Panama**	2013	UNICEF MICS	18,960	90	12	161587	No
**Papua New Guinea**	2000	Country-specific	2,569,236	549	20	58191†	No

Country	Source Year	Source	Sample Size	Number of geo-positioned clusters	Number of polygons	GHDx ID*	In Last Paper
**Papua New Guinea**	2000	Census	257,478	589	87	367640	No
**Papua New Guinea**	2006	DHS	22,344	654	0	44870†	No
**Paraguay**	2002	Census	250,883	524	96	227167	No
**Paraguay**	2016	UNICEF MICS	15,317	476	9	324470	No
**Peru**	2000	DHS	62,235	1,408	0	20649	No
**Peru**	2003	DHS	69,409	1,409	0	275090	No
**Peru**	2007	Census	1,456,206	1,609	175	41267	No
**Peru**	2009	DHS	51,622	1,131	0	270404	No
**Peru**	2010	DHS	49,849	1,536	24	270469	No
**Peru**	2011	DHS	48,474	1,536	24	270470	No
**Peru**	2012	DHS	51,269	1,536	24	270471	No
**Peru**	2013	DHS	48,677	1,536	24	146860	No
**Peru**	2013	DHS	48,731	1,535	25	210231	No
**Peru**	2014	DHS	52,859	1,536	24	209930	No
**Peru**	2014	DHS	52,867	1,241	0	210182	No
**Philippines**	1998	DHS	31,562	386	82	20683	No
**Philippines**	2000	Census	3,613,086	1,291	1,235	41296	No
**Philippines**	2003	DHS	30,571	815	0	20699	No
**Philippines**	2008	DHS	30,285	789	0	21421	No
**Philippines**	2010	Census	4,900,544	1,355	1,308	367607	No
**Philippines**	2013	DHS	35,860	350	17	142943	No
**Philippines**	2017	DHS	56,473	1,212	0	337877	No
**Republic of the Congo**	2005	DHS	15,007	406	12	19391	Yes
**Republic of the Congo**	2009	DHS AIS	13,363	406	12	3133	No
**Republic of the Congo**	2011	DHS	22,063	406	12	56151	Yes
**Republic of the Congo**	2014	UNICEF MICS	22,785	369	11	234733	No
**Rwanda**	2000	DHS	20,023	36	12	20722	Yes
**Rwanda**	2000	UNICEF MICS	9,450	36	12	26930	Yes
**Rwanda**	2002	Census	364,889	107	104	42432	Yes
**Rwanda**	2005	DHS	21,280	456	0	20740	Yes
**Rwanda**	2010	DHS	25,859	492	0	56040	Yes
**Rwanda**	2011	Country-specific	3,448	31	5	56426	No
**Rwanda**	2012	DHS MIS	5,118	31	5	77391	No
**Rwanda**	2012	Census	477,969	41	29	367645	No
**Rwanda**	2014	DHS	25,563	492	0	157063	Yes
**Rwanda**	2017	DHS MIS	5,008	43	30	350836	No

Country	Source Year	Source	Sample Size	Number of geo-positioned clusters	Number of polygons	GHDx ID*	In Last Paper
**São Tomé and Príncipe**	2000	UNICEF MICS	6,352	4	4	27055	Yes
**São Tomé and Príncipe**	2008	DHS	5,807	7	7	26866	Yes
**São Tomé and Príncipe**	2014	UNICEF MICS	5,811	7	7	214640	Yes
**Senegal**	1999	DHS	17,188	165	10	20786	No
**Senegal**	2000	UNICEF MICS	25,780	241	10	27044	Yes
**Senegal**	2002	Census	471,859	254	34	43142	Yes
**Senegal**	2005	DHS	28,417	366	0	26855	Yes
**Senegal**	2006	DHS MIS	6,639	243	11	11516	No
**Senegal**	2009	DHS MIS	19,297	319	0	11540	No
**Senegal**	2010	DHS	30,888	385	0	56063	Yes
**Senegal**	2012	DHS	17,231	200	0	111432	Yes
**Senegal**	2014	DHS	15,876	197	0	191270	Yes
**Senegal**	2015	DHS	16,637	214	0	218592	Yes
**Senegal**	2016	DHS	16,567	214	0	286772	Yes
**Sierra Leone**	2000	UNICEF MICS	10,764	86	4	11639	Yes
**Sierra Leone**	2004	Census	234,719	136	100	11661	Yes
**Sierra Leone**	2005	UNICEF MICS	18,478	93	14	11649	No
**Sierra Leone**	2008	DHS	15,720	350	0	21258	Yes
**Sierra Leone**	2010	UNICEF MICS	29,820	93	14	76700	Yes
**Sierra Leone**	2013	DHS	31,760	435	0	131467	Yes
**Sierra Leone**	2016	DHS MIS	8,463	93	14	286773	No
**Somalia**	2006	UNICEF MICS	13,529	751	18	11774	Yes
**Somalia**	2011	UNICEF MICS	11,969	245	0	91507	Yes
**Somalia**	2011	UNICEF MICS	10,782	276	0	91508	No
**South Africa**	1998	Country-specific	41,551	1,609	9	12105	No
**South Africa**	1998	DHS	23,502	1,609	9	20796	Yes
**South Africa**	2001	Census	24,330,578	1,785	354	12144†	No
**South Africa**	2001	Census	1,994,695	1,635	53	43152	Yes
**South Africa**	2002	Country-specific	53,175	1,609	9	115481	No
**South Africa**	2003	Country-specific	68,433	1,609	9	11787	No
**South Africa**	2004	Country-specific	51,149	1,609	9	11788	No
**South Africa**	2005	Country-specific	55,627	1,609	9	11789	No
**South Africa**	2006	Country-specific	55,213	1,609	9	115486	No
**South Africa**	2007	Country-specific	56,909	1,609	9	11790	No
**South Africa**	2007	Census	500,790	1,632	52	43158	Yes
**South Africa**	2008	Country-specific	49,139	1,609	9	115488	No
**South Africa**	2009	Country-specific	48,561	1,609	9	115489	No
**South Africa**	2010	Country-specific	49,421	1,609	9	115490	No

Country	Source Year	Source	Sample Size	Number of geo-positioned clusters	Number of polygons	GHDx ID*	In Last Paper
**South Africa**	2011	Census	2,332,162	1,631	52	227194	Yes
**South Africa**	2013	Country-specific	40,494	1,609	9	162652	No
**South Africa**	2014	Country-specific	45,845	1,609	9	238485	No
**South Africa**	2016	DHS	19,156	741	0	157064	No
**South Africa**	2016	Country-specific	1,699,197	1,718	212	280803	No
**South Africa**	2016	Country-specific	36,984	1,609	9	317089	No
**South Sudan**	2010	UNICEF MICS	20,531	748	10	32189	No
**Sudan**	2008	Census	542,765	0	72	106548	Yes
**Sudan**	2000	UNICEF MICS	65,449	2,257	16	12243	No
**Sudan**	2008	Census	2,253,065	2,291	126	43167	No
**Sudan**	2010	UNICEF MICS	35,181	2,244	15	153643	No
**Sudan**	2014	UNICEF MICS	40,931	2,272	18	200617	No
**Suriname**	1999	UNICEF MICS	8,736	118	10	12280	No
**Suriname**	2010	UNICEF MICS	13,479	118	10	81203	No
**Swaziland**	2000	UNICEF MICS	10,177	24	4	12320	Yes
**Swaziland**	2006	DHS	9,941	270	0	20829	Yes
**Swaziland**	2010	UNICEF MICS	9,150	24	4	30325	Yes
**Swaziland**	2014	UNICEF MICS	9,597	24	4	200707	No
**Tajikistan**	1999	Country-specific	4,961	213	5	12455	No
**Tajikistan**	2000	UNICEF MICS	12,421	213	5	12595	No
**Tajikistan**	2005	UNICEF MICS	20,685	213	5	12608	No
**Tajikistan**	2007	Country-specific	14,866	261	0	12584	No
**Tajikistan**	2012	DHS	18,876	343	0	74460	No
**Tajikistan**	2017	DHS	21,576	365	0	341838	No
**Tanzania**	1999	DHS	8,217	173	0	20865	Yes
**Tanzania**	2002	Census	1,719,577	1,112	129	43212	Yes
**Tanzania**	2003	DHS AIS	14,167	345	0	12630	No
**Tanzania**	2004	DHS	21,513	1,061	26	20875	Yes
**Tanzania**	2008	DHS AIS	18,901	466	0	12644	No
**Tanzania**	2009	DHS	20,629	458	0	21331	Yes
**Tanzania**	2011	DHS AIS	21,950	573	0	77395	No
**Tanzania**	2012	Census	2,054,046	1,129	169	294725	No
**Tanzania**	2015	DHS	27,670	608	0	218593	Yes
**Thailand**	2000	Census	347,465	659	76	43231	No
**Thailand**	2005	UNICEF MICS	70,730	623	4	12732	No
**Thailand**	2012	UNICEF MICS	42,441	623	5	148649	No
**Thailand**	2015	UNICEF MICS	49,442	623	5	296646	No
**Thailand**	2016	UNICEF MICS	5,804	2	1	331377	No
**The Gambia**	2000	UNICEF MICS	10,047	17	7	3922	Yes

Country	Source Year	Source	Sample Size	Number of geo-positioned clusters	Number of polygons	GHDx ID*	In Last Paper
**The Gambia**	2010	UNICEF MICS	22,549	13	5	91506	No
**The Gambia**	2013	DHS	19,751	36	36	77384	Yes
**Timor-Leste**	2001	Country-specific	1,411	3	2	12863	No
**Timor-Leste**	2003	DHS	10,749	379	92	20888†	No
**Timor-Leste**	2009	DHS	27,914	23	13	21274	No
**Timor-Leste**	2016	DHS	26,425	455	0	286785	No
**Togo**	2000	UNICEF MICS	24,485	0	30	12886	Yes
**Togo**	1998	DHS	17,777	287	0	20909	Yes
**Togo**	2006	UNICEF MICS	13,862	70	6	12896	Yes
**Togo**	2010	UNICEF MICS	13,214	70	6	40021	Yes
**Togo**	2013	DHS	19,238	330	0	77515	Yes
**Trinidad and Tobago**	2000	Census	59,322	16	15	294806	No
**Trinidad and Tobago**	2006	UNICEF MICS	10,000	15	15	12950	No
**Trinidad and Tobago**	2011	Census	60,902	16	15	294807	No
**Trinidad and Tobago**	2011	UNICEF MICS	9,016	8	5	332558	No
**Tunisia**	2011	UNICEF MICS	20,596	223	9	76709	Yes
**Turkmenistan**	2006	UNICEF MICS	13,859	709	6	13064	No
**Turkmenistan**	2015	UNICEF MICS	15,019	709	6	264583	No
**Uganda**	2000	DHS	14,120	270	0	20993	Yes
**Uganda**	2002	Country-specific	1,087,339	318	161	43328	Yes
**Uganda**	2004	DHS AIS	20,039	256	55	13084†	No
**Uganda**	2006	DHS	16,206	336	0	21014	Yes
**Uganda**	2011	DHS AIS	20,707	470	0	55973	No
**Uganda**	2011	DHS	18,266	400	0	56021	Yes
**Uganda**	2014	DHS MIS	5,227	208	0	157065	No
**Uganda**	2016	DHS	36,805	685	0	286780	No
**Uzbekistan**	2000	UNICEF MICS	15,464	678	5	13436	No
**Uzbekistan**	2002	Country-specific	11,457	218	0	21039	No
**Uzbekistan**	2006	UNICEF MICS	28,484	678	6	13445	No
**Venezuela**	2001	Census	1,216,987	1,130	237	43412	No
**Vietnam**	1999	Census	1,277,625	429	61	43718	No
**Vietnam**	2000	UNICEF MICS	18,652	403	8	13708	No
**Vietnam**	2002	DHS	17,216	273	41	21058	No
**Vietnam**	2004	Country-specific	18,657	431	64	13693†	No
**Vietnam**	2005	DHS AIS	14,631	431	64	13544	No
**Vietnam**	2006	UNICEF MICS	20,230	405	8	13719	No
**Vietnam**	2009	Census	8,192,555	827	673	43726	No

Country	Source Year	Source	Sample Size	Number of geo-positioned clusters	Number of polygons	GHDx ID*	In Last Paper
**Vietnam**	2010	UNICEF MICS	24,102	226	6	57999	No
**Vietnam**	2013	UNICEF MICS	20,342	508	508	152735	No
**Yemen**	2006	UNICEF MICS	11,917	175	21	13816	Yes
**Yemen**	2012	Country-specific	62,650	165	19	249499	No
**Yemen**	2013	DHS	55,227	175	21	112500	No
**Zambia**	1999	UNICEF MICS	18,627	892	70	14122	No
**Zambia**	2000	Country-specific	459,953	968	150	151325	Yes
**Zambia**	2001	DHS	519	896	9	21102	Yes
**Zambia**	2002	DHS	11,048	929	72	27898†	No
**Zambia**	2007	DHS	14,922	319	0	21117	No
**Zambia**	2010	Country-specific	579,018	968	150	151326	Yes
**Zambia**	2013	DHS	34,371	719	0	77516	Yes
**Zimbabwe**	1999	DHS	12,204	221	0	21151	Yes
**Zimbabwe**	2005	DHS	18,798	396	0	21163	Yes
**Zimbabwe**	2009	UNICEF MICS	23,995	486	10	35493	Yes
**Zimbabwe**	2010	DHS	17,967	393	0	55992	Yes
**Zimbabwe**	2012	Census	312,911	537	88	367747	No
**Zimbabwe**	2014	UNICEF MICS	29,221	486	10	152720	Yes
**Zimbabwe**	2015	DHS	19,947	400	0	157066	Yes

* GHDx ID = Data source unique identifier in the Global Health Data Exchange (http://ghdx.healthdata.org/). Additional information about each data source is available via the GHDx, including information about the data provider and links to where the data can be accessed or requested (where available). NIDs can be entered in the search bar to retrieve the record for a particular source.

† Data source is not publicly available due to restrictions by the data provider and was used under license for the current study.

3.1 Data excluded from model

Select data sources that were identified to contain years of education within the geographic area of interest were excluded for the following reasons: missing survey weights for areal data, missing gender variable, incomplete sampling (e.g., only a specific age range), high rates of missingness in education variables, implausible education data values, or untrustworthy data (as determined by the survey administrator or by inspection). This source was removed due to implausibly high educational attainment values around the capital city and expert opinion regarding the quality of the data. Within each source, administrative units with a sample size of one were excluded. We define high missingness as 50% of respondents missing a response for the relevant education variable. A survey was determined to have an implausible value if its spread of data for any indicator modelled was highly disparate from other surveys in the same location and similar years. A source identified for exclusion was excluded for all indicator and all age-sex combinations. All exclusions are listed in Supplementary Table 4.

Supplementary Table 4. Excluded sources

Country	Source	Year	Reason for Exclusion	GHDx ID*
**All**	Gallup World Poll	1998–2017	Non-representative below national level	367574
**All**	ISSP	1998–2017	Survey dates unclear, not usable	142405
**All**	Afrobarometer	1998–2017	Very small sample sizes, non-representative	340004
**All**	Eurobarometer	1998–2017	No stage 1 or 2 country data	96291
**All**	WHS	1998–2017	Geographic data unusable	542
**Bhutan**	Bhutan Census	2005	Dropped for missingness in indicator responses	1175
**Brazil**	National Health Survey	2013	Dropped for missing weights	195010
**Cambodia**	IPUMS Census	1998	Small sample size, indicator measures not consistent with rest of data	35322
**Cambodia**	IPUMS Census	2008	Small sample size, indicator measures not consistent with rest of data	35329
**Cambodia**	Intercensal Pop Survey	2004	Small sample size, indicator measures not consistent with rest of data	2002
**Cambodia**	Labor Force Survey	2000	Mean educational attainment estimates unrealistically high	282112
**Cambodia**	IPUMS Census	1998	Zero proportion estimate unrealistically high	35322
**China**	WB STEP Household Survey	2012	Dropped for missing weights	298372
**China**	IPUMS Census	2000	Mean educational attainment estimates unrealistically low	294255
**Ethiopia**	IPUMS Census	2007	Dropped for missingness in indicator responses	227133
**Ethiopia**	PMA	2014	Extremely small sample size, indicator estimates non-representative/ missingness in indicator responses	153503
**Ethiopia**	PMA	2014	Extremely small sample size, indicator estimates non-representative	256175
**Ethiopia**	PMA	2015	Extremely small sample size, indicator estimates non-representative	256176
**India**	Longitudinal Aging Study	2010	Dropped for missing weights	174154

Country	Source	Year	Reason for Exclusion	GHDx ID*
**India**	District Level Household Survey	2007	Dropped for missingness in indicator responses	23258
**India**	Global Adult Tobacco Survey	2009	Mean educational attainment estimates unrealistically high	21988
**India**	District Level Household Survey	2012	Mean educational attainment estimates unrealistically low	165390
**India**	District Level Household Survey	2012	Dropped for incomplete age ranges	165390
**Indonesia**	IPUMS Census	2000	Zero proportion/NA estimate unrealistically high	56554
**Indonesia**	UNICEF MICS	2000	Missingness in education variable	27009
**Iraq**	Socioeconomic Survey	2012	Dropped for missingness in indicator responses	235348
**Kenya**	UNICEF MICS	2007	Small sample size, indicator measures not consistent with rest of data	155335
**Kenya**	Kenya AIDS Indicator Survey	2012	Dropped for incomplete age ranges	133304
**Kenya**	Kenya AIDS Indicator Survey	2007	Dropped for incomplete age ranges	133219
**Kyrgyzstan**	WB LSMS	1998	Dropped for missingness in indicator responses	45989
**Kyrgyzstan**	IPUMS Census	1999	Dropped for missingness in indicator responses	39466
**Kyrgyzstan**	UNICEF MICS	2014	Dropped for missing weights	162283
**Laos**	UNICEF MICS	2006	Mean educational attainment estimates unrealistically high	7629
**Madagascar**	UNICEF MICS	2012	Dropped for missingness in indicator responses	125594
**Malawi**	WB LSMS	2013	Inconsistent w/ Country's time trend	224223
**Mexico**	Mexico National Addiction Survey	2002	Dropped for incomplete age ranges	8672
**Mexico**	IPUMS Census	2005	Dropped for incomplete age ranges	43776
**Mexico**	Mexico National Addiction Survey	2008	Dropped for incomplete age ranges	105286
**Mexico**	Income and Household Expenditure Survey	2010	Mean educational attainment estimates unrealistically low	93321
**Nigeria**	Household School and Health Facility Survey	2005	Mean educational attainment estimates unrealistically low	50393

Country	Source	Year	Reason for Exclusion	GHDx ID*
**Palestine**	IPUMS Census	2007	Dropped for missing weights	41088
**South Sudan**	IPUMS Census	2008	Dropped for missingness in indicator responses	106548
**Swaziland**	UNICEF MICS	2000	Mean educational attainment estimates unrealistically high	12320
**Tanzania**	WB LSMS	2010	Inconsistent w/ Country's time trend	93807
**Turkmenistan**	UNICEF MICS	2015	Dropped for missing weights	264583
**Vietnam**	WB LSMS	2004	Mean educational attainment estimates unrealistically low	13693

* GHDx ID = Data source unique identifier in the Global Health Data Exchange (http://ghdx.healthdata.org/). Additional information about each data source is available via the GHDx, including information about the data provider and links to where the data can be accessed or requested (where available). NIDs can be entered in the search bar to retrieve the record for a particular source.

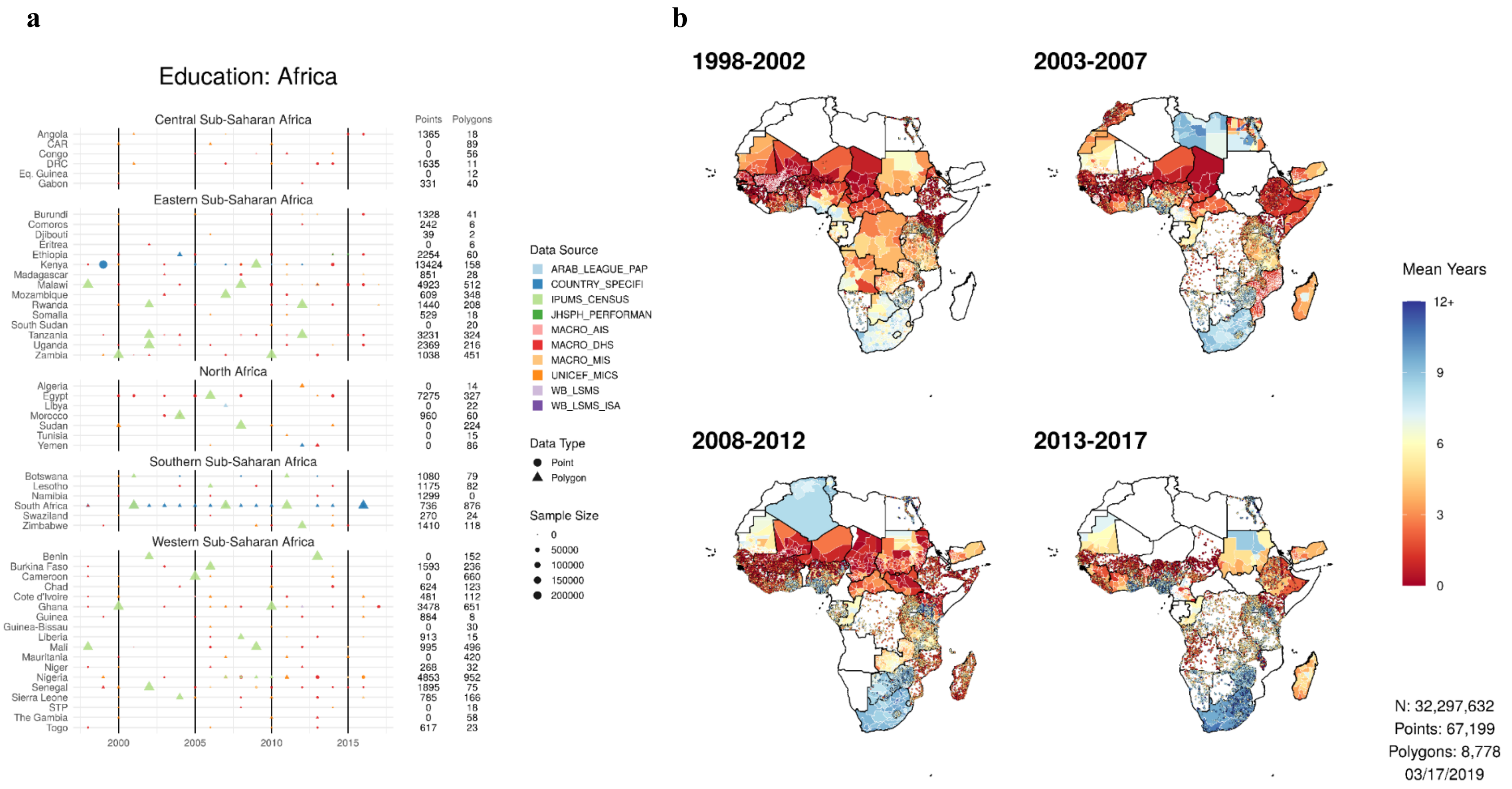

Supplementary Figure 1. Education data availability in Africa by type and country for women ages 15–49, 1998–2017

Data are shown by country and year of survey and mapped at their corresponding geopositioned coordinate or area. All points and polygons (areal) for each country are plotted by data source, type, and sample size (a). Sample size represents the number of individual microdata records for each survey. Mean years of attainment for the input coordinate or area are mapped (b). This database consists of 67,199 clusters and 8,778 polygons.

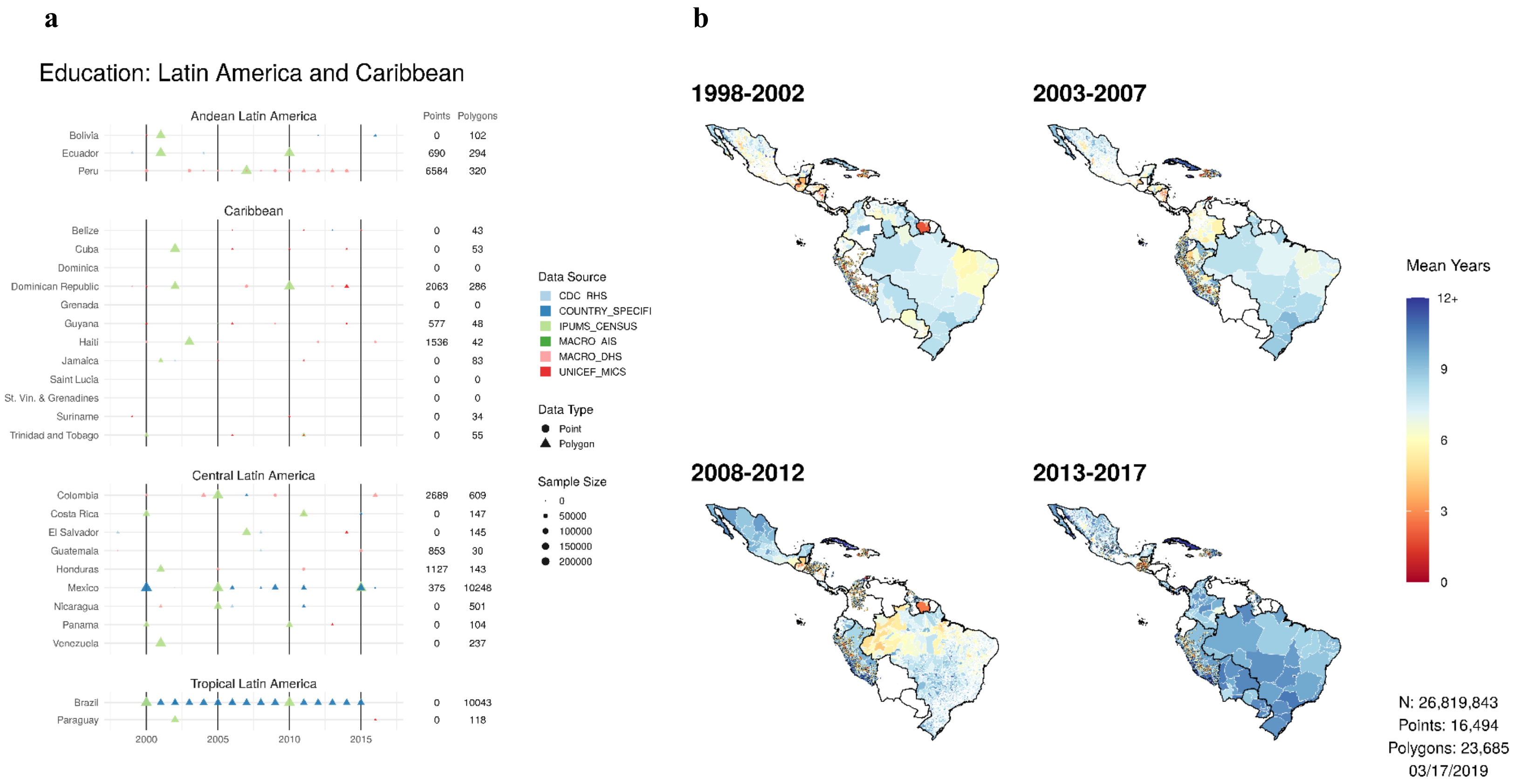

Supplementary Figure 2. Education data availability in Latin America and the Caribbean by type and country for women ages 15–49, 1998–2017

Data are shown by country and year of survey and mapped at their corresponding geopositioned coordinate or area. The total number of points and polygons (areal) for each country are plotted by data source, type, and sample size (a). Sample size represents the number of individual microdata records for each survey. Mean years of attainment for the input coordinate or area are mapped (b). This database consists of 16,494 clusters and 23,685 polygons.

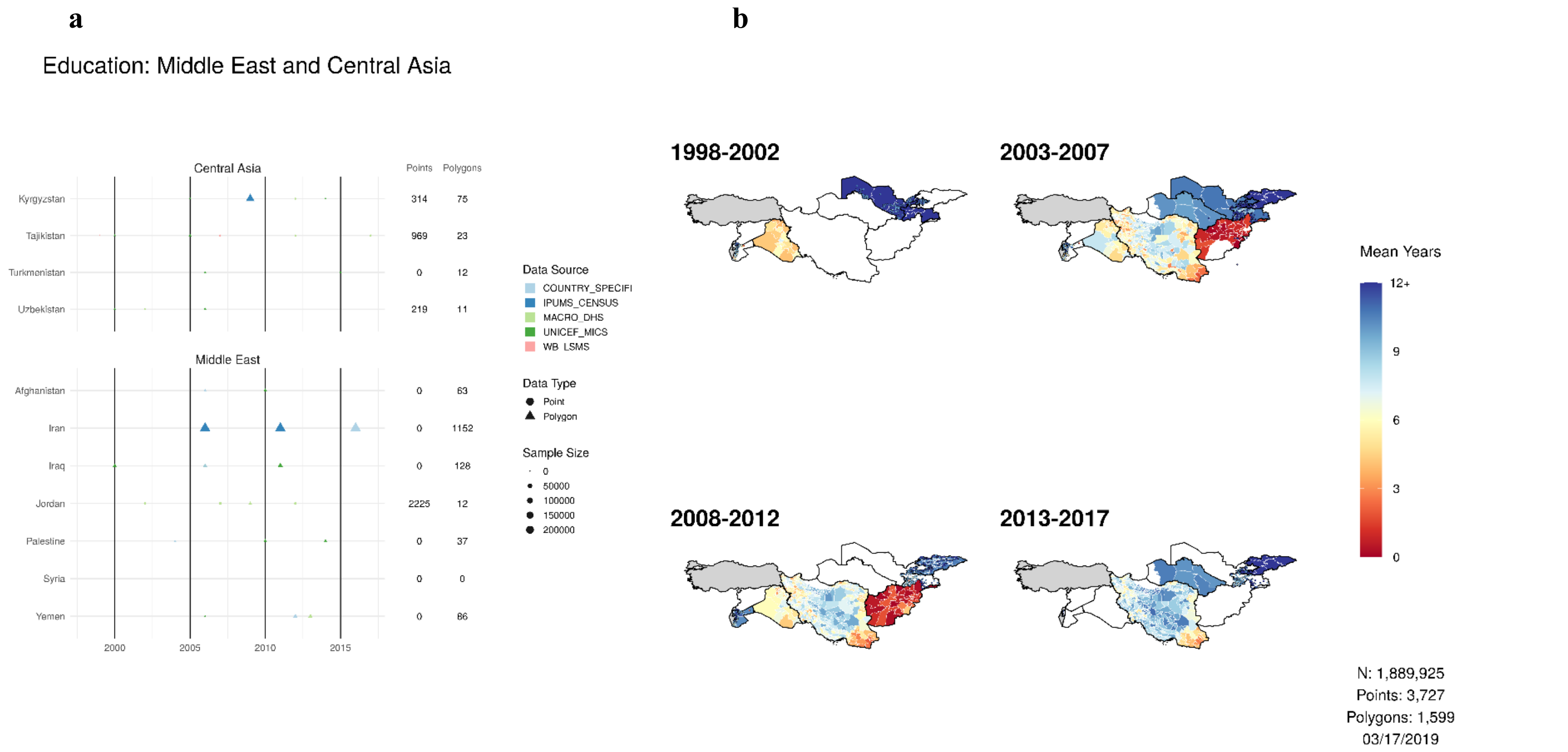

Supplementary Figure 3. Education data availability in the Middle East and Central Asia by type and country for women ages 15–49, 1998–2017

Data are shown by country and year of survey and mapped at their corresponding geopositioned coordinate or area. The total number of points and polygons (areal) for each country are plotted by data source, type, and sample size (a). Sample size represents the number of individual microdata records for each survey. Mean years of attainment for the input coordinate or area are mapped (b). This database consists of 3,727 clusters and 1,599 polygons.

a b

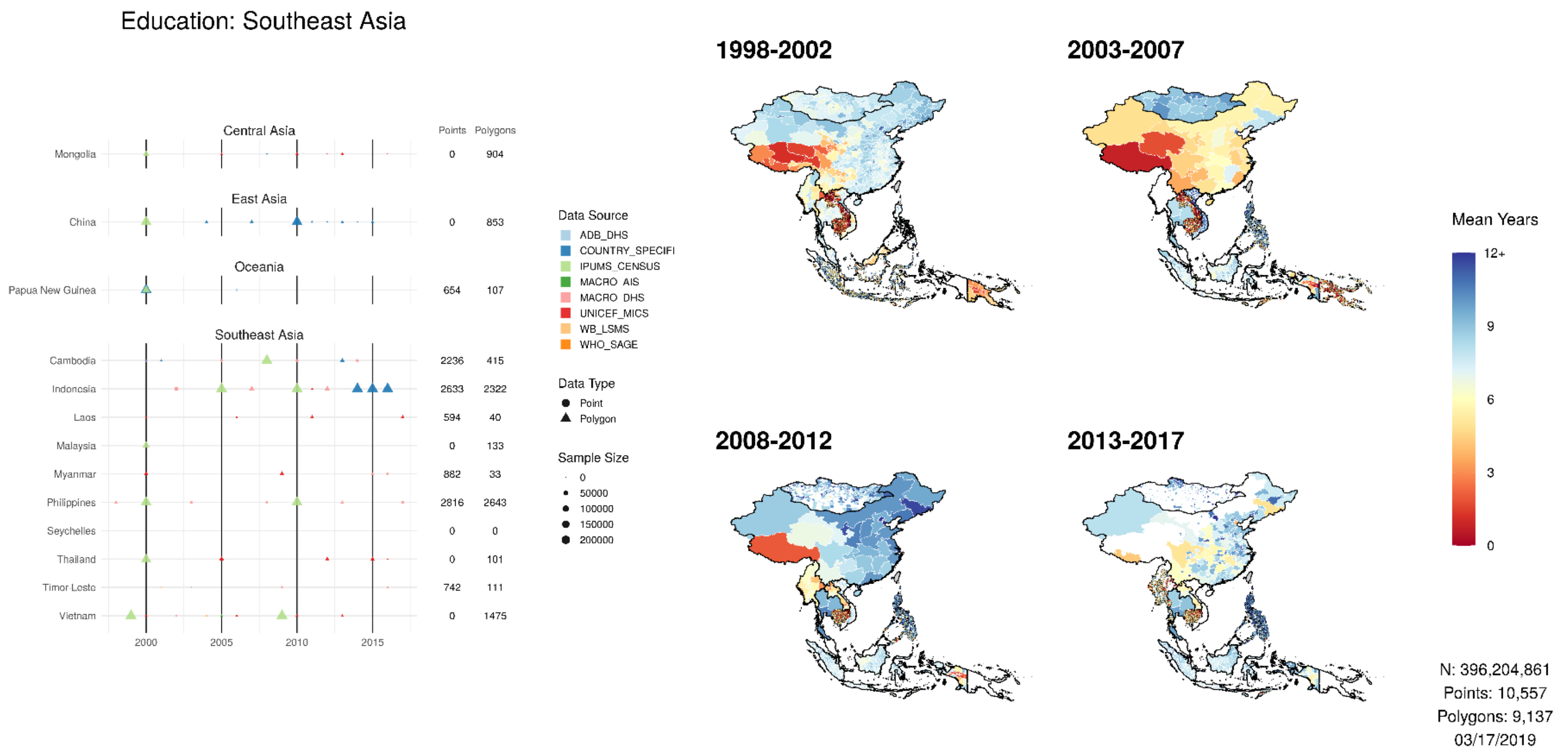

Supplementary Figure 4. Education data availability in East and Southeast Asia by type and country for women ages 15–49, 1998–2017
Data are shown by country and year of survey and mapped at their corresponding geopositioned coordinate or area. All points and polygons (areal) for each country are plotted by data source, type, and sample size (a). Sample size represents the number of individual microdata records for each survey. Mean years of attainment for the input coordinate or area are mapped (b). This database consists of 10,557 clusters and 9,137 polygons.

a b

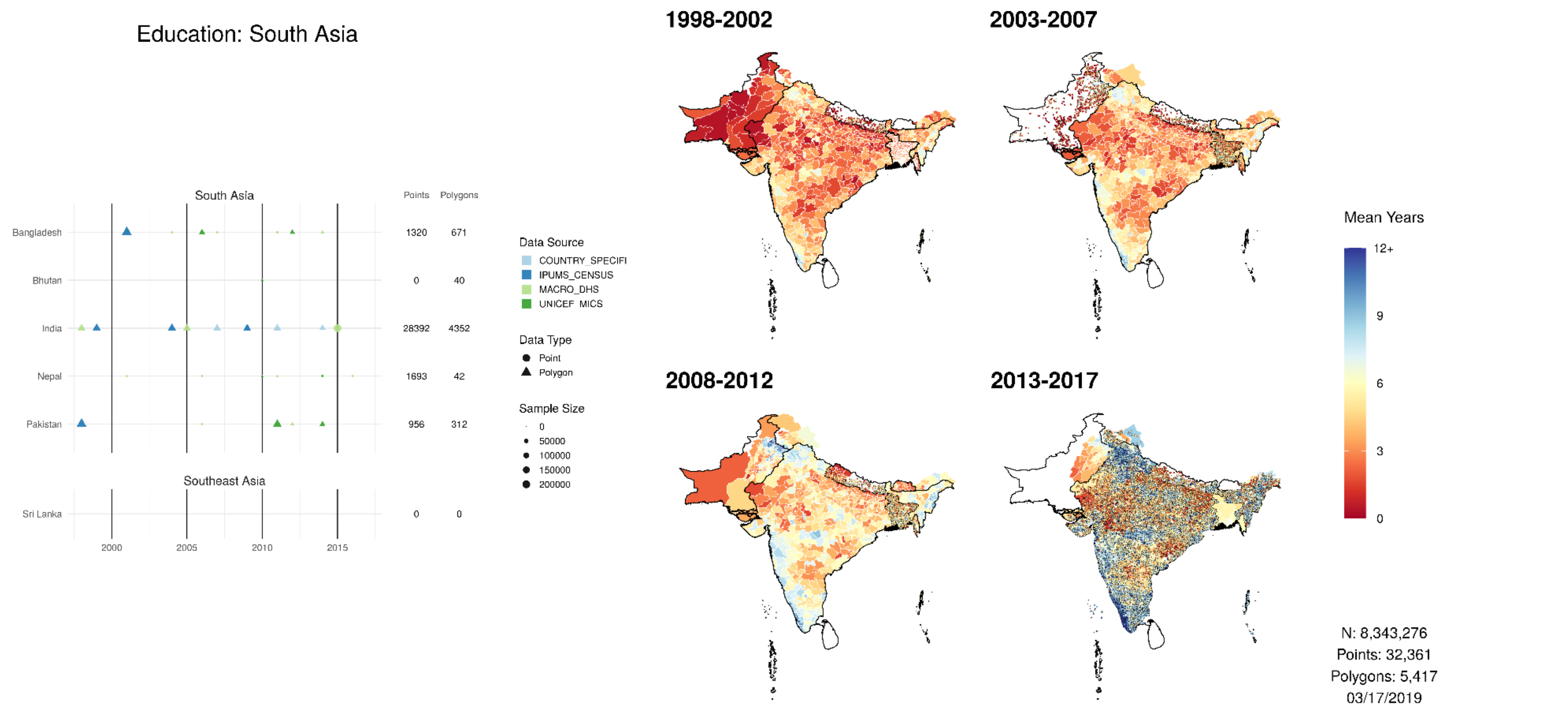

Supplementary Figure 5. Education data availability in South Asia by type and country for women ages 15–49, 1998–2017

Data are shown by country and year of survey and mapped at their corresponding geopositioned coordinate or area. All points and polygons (areal) for each country are plotted by data source, type, and sample size (a). Sample size represents the number of individual microdata records for each survey. Mean years of attainment for the input coordinate or area are mapped (b). This database consists of 32,361 clusters and 5,417 polygons.

4.0 Supplementary covariates

A variety of socioeconomic and environmental variables were used to predict educational attainment. Where available, the finest spatio-temporal resolution of gridded data sets was used. The most recent year available was substituted in for annual covariates without availability through the entire studied time period (2000–2017); the temporal availability of covariates is listed in Supplementary Table 5. One covariate raster brick, the population estimates from WorldPop, are only made available for the years 2000, 2005, 2010, and 2015. Because our model is indexed by individual year between 2000 and 2015, it is necessary for us to interpolate these population estimates between reported years. We use the exponential population growth rate calculated between each pair of years to interpolate to missing years between.

Supplementary Table 5. Covariates used in mapping

Covariate	Temporal resolution	Source	Reference
Aridity	Annual (2000–2017)	Climatic Research Unit Time-Series (CRUTS)	Harris, I., Jones, P. d., Osborn, T. j. & Lister, D. h. Updated high-resolution grids of monthly climatic observations – the CRU TS3.10 dataset. *Int. J. Climatol*. **34**, 623–642 (2014).<br><br>University of East Anglia. Climatic Research Unit TS v. 3.24 dataset. Available at: https://crudata.uea.ac.uk/cru/data/hrg/cru_ts_3.24.01/. (Accessed: 24th July 2017).
Irrigation	Static	University of Frankfurt	Goethe-Universität. Generation of a digital global map of irrigation areas. Available at: https://www.uni-frankfurt.de/45218039/Global_Irrigation_Map. (Accessed: 25th July 2017)
Nighttime lights	Annual (2000–2013)	NOAA DMSP	NOAA. Version 4 DMSP-OLS nighttime lights time series dataset. Available at: https://www.ngdc.noaa.gov/eog/dmsp/downloadV4composites.html. (Accessed: 25th July 2017)
Population	Annual (2000–2017)	WorldPop	Lloyd, C. T., Sorichetta, A. & Tatem, A. J. High resolution global gridded data for use in population studies. *Sci. Data* **4**, sdata20171 (2017).<br><br>World Pop. Get data. Available at: http://www.worldpop.org.uk/data/get_data/. (Accessed: 25th July 2017)

Covariate	Temporal resolution	Source	Reference
Travel time to nearest settlement >50,000 inhabitants	Static	Malaria Atlas Project, Big Data Institute, Nuffield Department of Medicine, University of Oxford	Weiss, D. J. *et al.* A global map of travel time to cities to assess inequalities in accessibility in 2015. *Nature* **533,** 333–336 (2018).
Urbanicity	Annual (2000–2016)	European Commission/ GHS	Pesaresi, M. et al. Operating procedure for the production of the Global Human Settlement Layer from Landsat data of the epochs 1975, 1990, 2000, and 2014. (Publications Office of the European Union, 2016).
Elevation	Static	NOAA	Hastings, David A., and Paula K. Dunbar. Global Land One-kilometer Base Elevation (GLOBE) Digital Elevation Model, Documentation, Volume 1.0. Key to Geophysical Records Documentation (KGRD) 34. National Oceanic and Atmospheric Administration, National Geophysical Data Center, 325 Broadway, Boulder, Colorado 80303, U.S.A (1999)<br><br>GLOBE Task Team and others (Hastings, David A., Paula K. Dunbar, Gerald M. Elphingstone, Mark Bootz, Hiroshi Murakami, Hiroshi Maruyama, Hiroshi Masaharu, Peter Holland, John Payne, Nevin A. Bryant, Thomas L. Logan, J.-P. Muller, Gunter Schreier, and John S. MacDonald), eds., 1999. The Global Land One-kilometer Base Elevation (GLOBE) Digital Elevation Model, Version 1.0. National Oceanic and Atmospheric Administration, National Geophysical Data Center, 325 Broadway, Boulder, Colorado 80303, U.S.A. Available at: https://www.ngdc.noaa.gov/mgg/topo/globe.html. (Accessed: 16th February 2017).
Growing Season Length	Static	FAO (derived)	FAO. GAEZ - Global Agro-Ecological Zones data portal. Available at: http://www.fao.org/nr/gaez/about-data-portal/en/. (Accessed: 3/20/2018).

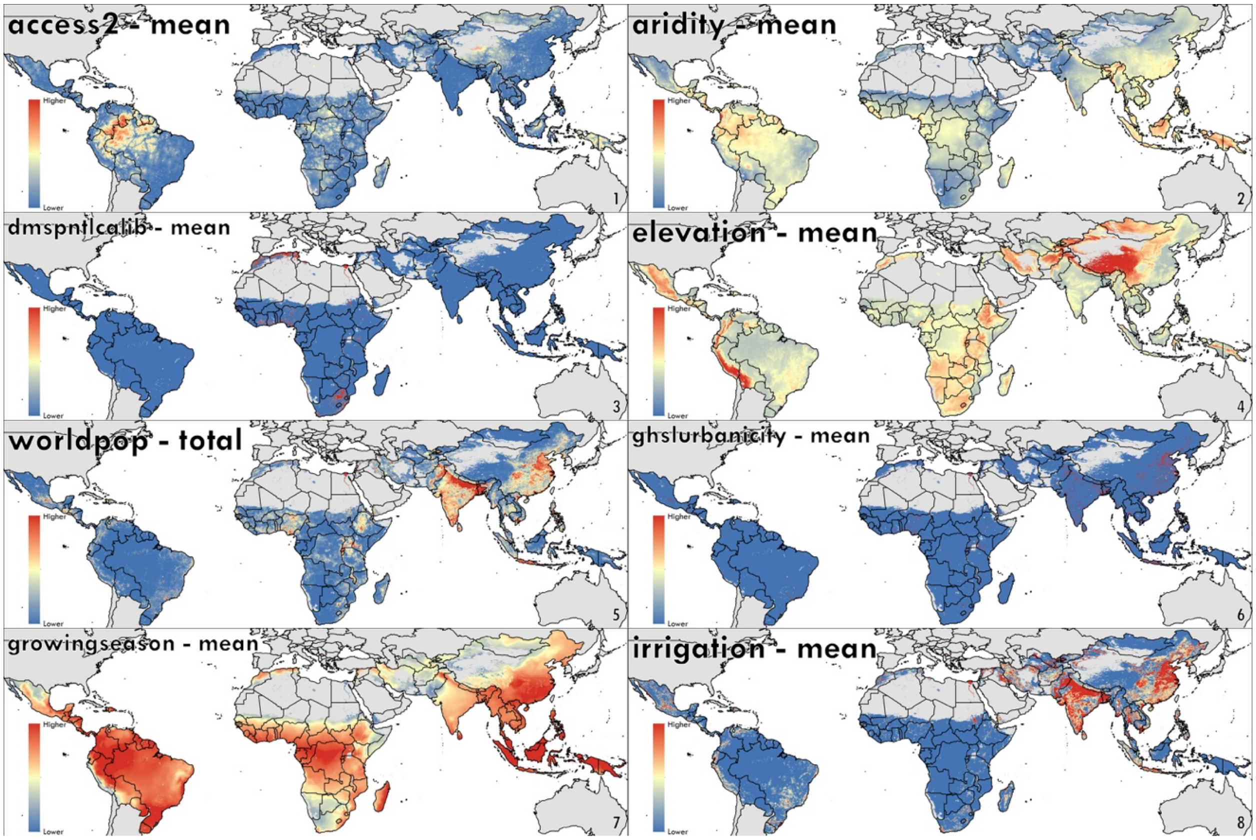

Supplementary Figure 6. Covariates

Eight covariate raster layers of possible socioeconomic and environmental correlates of educational attainment in LMICs were used as inputs for the stacking modelling process; **1** – travel time to nearest settlement; **2 –** aridity; **3 –** nighttime lights; **4 –** elevation; **5 –** population; **6 –** urbanicity; **7 –** growing season length; **8 –** irrigation. Time-varying covariates are presented for the most recent available year. The year of production of non-time-varying covariates is provided in the individual covariate citation in Supplementary Table 3.

5.0 Supplementary methods

5.1 Cluster combination and spatial integration over polygon records

Our individual-level data were collapsed (summarised) into clusters if they could be georeferenced to latitude-longitude pairs. Otherwise, we collapsed our individual-level data to the smallest polygon that could be referenced. We used survey weights and the survey package in R to account for non-equal sampling probabilities due to non-simple-random sampling[29].

Data without latitude and longitude, but that could be geolocated to an administrative area, were resampled to generate candidate point locations based on the underlying population of the administrative area. The main concept is to leverage covariate values across the polygon when performing the regression, while simultaneously accounting for a population-driven survey design. The methods used for the resampling are consistent with those used in geospatial modelling of under-5 mortality, published previously.

For each polygon-level observation, 10,000 points were randomly sampled from within the polygon (regardless of the polygon's area) using the WorldPop total population raster[30] to weight the locations of the draws. K-means clustering was performed on the candidate points to generate integration points (1 per 1,000 pixels) used in the modelling. Weights were assigned to each integration point proportionally to the number of candidate points that entered into the k-means cluster, such that the weight of each point represented the number of population-sampled locations contained within the K-means cluster location, divided by the number of sampled points generated (10,000). Each point generated by this process was assigned the mean attainment or relevant proportion of schooling observed from the survey for that polygon. These sample weights were used directly in the likelihood evaluation during model fitting[20].

5.2 Geostatistical model

5.2.1 Model geographies

Models were run for each indicator in fourteen continuous geographic regions within all LMICs chosen to align with the regions used in the Global Burden of Disease (GBD) study, which determines regions based on epidemiological homogeneity, sociodemographic similarity and geographic contiguity (see Supplementary Fig. 1–5 for lists of regions and countries). Minor changes were made to the GBD regions to ensure spatial contiguity within regions (see Extended Data Fig. 1 for an illustration of the modelling regions). All data within the spatial region, and within a one-degree buffer from the boundaries of each region, were included in each model to minimise edge effects.

5.2.2 Ensemble covariate modelling

An ensemble covariate modelling method was implemented in order to both select covariates and capture possible non-linear effects and complex interactions between them[31]. For each region, three sub-models were fit to our dataset, using all of our covariate data as explanatory predictors: generalised additive models, boosted regression trees, and lasso regression using the mgcv, glmnet, dismo, and caret packages in R. Sample weights are used in sub-models, where applicable, such that cluster locations with latitude and longitude had a sample weight of 1, while cluster locations where the latitude and longitude was generated by the polygon resampling process had a weight based on the K-means clustering process (see section 5.1, Cluster combination and spatial integration over polygon records).

Each sub-model is fit using five-fold cross-validation to avoid overfitting. The out-of-sample predictions from across the five holdouts are compiled into a single comprehensive set of predictions from that model. Additionally, the same sub-models were also run using 100% of the data, and a full set of in-sample predictions were created. The five sets of out-of-sample sub-model predictions are fed into the full geostatistical model as the explanatory covariates when performing the model fit. The in-sample predictions from the sub-models are used as the covariates when generating predictions using the fitted full geostatistical model. A recent study has shown that this ensemble approach can improve predictive validity by up to 25% over an individual model[31].

Predictions from each sub-model are generated based on patterns and relationships between the raw covariates and attainment data, while predictions from the full geostatistical model are generated based on patterns and relationships between the predictions from the ensemble of sub-models and attainment data. To discover the relationships between the sub-model prediction layers (used as covariates in the full geostatistical model) and the attainment data, the only values of the covariates (sub-model prediction layers) "seen" by the model are the values underlying the locations of surveys. As such, it is possible that estimates will be generated in areas where the values of the covariates exceed the minimum and maximum values observed by the model. In these areas, the estimates are generated by extrapolating from the patterns observed within the range of covariates underlying the survey and census data.

5.2.3 Model description

Gaussian and binomial data are modeled within a Bayesian hierarchical modelling framework using a spatially and temporally explicit hierarchical generalized linear regression model to fit mean years of education attainment and proportion of population achieving key bins of school in 14 regions across all LMICs as defined in the GBD study (see Extended Data Fig. 1)[32]. This means we fit 14 independent models for each indicator (i.e., the proportion of women with zero years of schooling). The GBD study design sought to create regions on the basis of three primary criteria: epidemiological homogeneity, sociodemographic similarity and geographic contiguity[32]. For each Gaussian indicator, we modelled the expected mean years of attainment in location i and time t using observed means in each cluster d as indicated by edu_d. Each cluster (d) is located precisely in time (t) and space (i). We model the mean years of attainment as Gaussian data given fixed precision τ and a scaling parameter s_i (defined by the sample size in the observed cluster mean).

$$edu_d | \mu_{i(d),t(d)}, s_d, \tau \sim \text{Normal}(\mu_{i(d),t(d)}, \tau * s_d)$$

$$\mu_{i,t} = \beta_0 + \mathbf{X}_{i,t}\boldsymbol{\beta} + Z_{i,t} + \epsilon_{\text{ctr}(i)} + \epsilon_{i,t}$$

For each binomial indicator, we modelled the expected probability of attaining that level of educational attainment in location i and time t using the observed number of women in cluster d, among an observed sample size N_d, who reported that level as indicated by binomial count data C_d.

$$C_d | p_{i(d),t(d)}, N_d \sim \text{Binomial}\left(p_{i(d),t(d)}, N_d\right)$$

$$\text{logit}\left(p_{i,t}\right) = \beta_0 + \mathbf{X}_{i,t}\boldsymbol{\beta} + Z_{i,t} + \epsilon_{ctr(i)} + \epsilon_{i,t}$$

We used a continuation-ratio modelling approach to account for the ordinal data structure of the binomial indicators[33]. To do this, the proportion of population with zero years of education was modelled using a binomial model. The proportion with less than primary education was modelled as those with less than primary education of those that have more than zero years of education. The same method followed for the proportion of population completing primary education. The proportion achieving secondary school or higher was estimated as the complement of sum of the three binomial models.

The remaining parameters were consistent between all indicators in both binomial and Gaussian models:

$$\sum_{h=1}^{3} \beta_h = 1$$

$$\epsilon_{\text{ctr}} \sim \text{iid Normal}(0, \gamma^2)$$

$$\epsilon_{i,t} \sim \text{iid Normal}(0, \sigma^2)$$

$$\mathbf{Z} \sim \text{GP}(0, \Sigma^{\text{space}} \otimes \Sigma^{\text{time}})$$

$$\Sigma^{\text{space}} = \frac{\omega^2}{\Gamma(\nu)2^{\nu-1}} \times (\kappa D)^{\nu} \times \mathrm{K}_{\nu}(\kappa D)$$

$$\Sigma_{j,k}^{\text{time}} = \rho^{|k-j|}$$

For indices d, i, and t, *(index) is the value of * at that index. The probabilities $p_{i,t}$ represent both the annual proportions at the space-time location and the probability that an individual had that level of attainment given that they lived at that particular location. The annual probability $p_{i,t}$ of each indicator (or $\mu_{i,t}$ for the mean indicators) was modelled as a linear combination of the three sub-models (generalised additive model (GAM), boosted regression trees (BRT), and lasso regression), rasterized covariate values $X_{i,t}$, a correlated spatiotemporal error term $Z_{i,t}$, country random effects $\epsilon_{ctr(i)}$) with one unstructured country random effect fit for each country in the modelling region and all sharing a common variance parameter, γ^2, and an independent nugget effect $\epsilon_{i,t}$ with variance parameter σ^2. Coefficients $\beta_h$in the three sub-models $h = 1, 2, 3$ represent their respective predictive weighting in the mean logit link, while the joint error term $Z_{i,t}$ accounts for residual spatiotemporal autocorrelation between individual data points that remains after accounting for the predictive effect of the sub-model covariates, the country-level random effect $\epsilon_{ctr(i)}$, and the nugget independent error term, $\epsilon_{i,t}$. The purpose of the country-level random effect is to capture spatially unstructured, unobserved country-specific variables, as there are often sharp discontinuities in educational attainment between adjacent countries due to systematic differences in governance, infrastructure, and social policy.

The residuals $Z_{i,t}$ are modelled as a three-dimensional Gaussian process (GP) in space-time centered at zero and with a covariance matrix constructed from a Kronecker product of spatial and temporal covariance kernels. The spatial covariance Σ^{space} is modelled using an isotropic and stationary Matérn function[34] and temporal covariance Σ^{time} as an annual autoregressive (AR1) function over the 18 years represented in the model. In the stationary Matérn function, Γ is the Gamma function, K_{ν} is the modified Bessel function of order $v > 0, \kappa > 0$ is a scaling parameter, D denotes the Euclidean distance, and ω^2 is the marginal variance. The scaling parameter, κ, is defined to be $\kappa = \sqrt{8v}/\delta$ where δ is a range parameter (which is about the distance where the covariance function approaches 0.1) and v is a scaling constant, which is set to 2 rather than fit from the data[35]. This parameter is difficult to reliably fit, as documented by many other analyses that set this to 2[35–37]. The number of rows and the number of columns of the spatial Matérn covariance matrix are equal to the number of spatial mesh points for a given modeling region. In the AR1 function, ρ is the autocorrelation function (ACF), and k and j are points in the time series where $|k - j|$ defines the lag. The number of rows and the number of columns of the AR1 covariance matrix are equal to the number of temporal mesh points[37]. The number of rows and the number of columns of the space-time covariance matrix $\Sigma^{space} \otimes \Sigma^{\text{time}}$) for a given modeling region are both equal to: (the number of spatial mesh points × the number of temporal mesh points).

This approach leveraged the data's residual correlation structure to more accurately predict estimates for locations with no data, while also propagating the dependence in the data through

to uncertainty estimates. The posterior distributions were fit using computationally efficient and accurate approximations in R-integrated nested Laplace approximation (INLA) with the stochastic partial differential equations (SPDE) approximation to the Gaussian process residuals using R project version 3.5.1. The SPDE approach using INLA has been demonstrated elsewhere, including the estimation of health indicators, particulate air matter, and population age structure[20,21,38,39]. Uncertainty intervals (UIs) were generated from 1,000 draws (i.e., statistically plausible candidate maps)[40] created from the posterior-estimated distributions of modelled parameters.

To transform grid cell-level estimates into a range of information useful to a wide constituency of potential users, these estimates were aggregated from the 1,000 candidate maps up to district, provincial, and national levels using 5 × 5-km^2 population data[6]. This aggregation also allowed for calibration of estimates to national GBD estimates for 2000–2017. This was achieved by calculating the ratio of the posterior mean national-level estimate from each candidate map draw in the analysis to the posterior mean national estimates from GBD, and then multiplying each cell in the posterior sample by this ratio. National level estimates from this analysis with GBD estimates can be found in Supplementary Table 44.

5.2.4 Priors

The following priors were used for all sixteen of our education models:

- $log\left(\frac{1+\rho}{1-\rho}\right) \sim Normal(\mu = 0, \sigma^2 = 1/0.15)$,
- $\theta_1 = \log(\tau) \sim Normal(\mu_{\theta_1}, \sigma^2_{\theta_1})$
- $\theta_2 = \log(\kappa) \sim Normal(\mu_2, \sigma^2_{\theta_2})$
- $\beta_0 \sim N(\mu = 0, \sigma^2 = 3^2)$,
- $\beta_h \sim N(\mu = 0, \sigma^2 = 3^2)$,
- $\left(\frac{1}{\sigma^2_{nugget}}\right) \sim gamma(\alpha = 1, \gamma = 2)$.
- $\left(\frac{1}{\sigma^2_{country}}\right) \sim gamma(\alpha = 1, \gamma = 2)$.

Given that our covariates used in INLA, i.e., the predicted outputs from the ensemble models, should be on the same scale as our predictive target, we believe that the intercept in our model should be close to zero and that the regression coefficients should sum to one. As such, we have chosen the prior for our intercept to be $\mathrm{N}(0, \sigma^2 = 3^2)$, and the prior for the fixed effect coefficients to be $\mathrm{N}(\frac{1}{\text{\# ensemble models}}, \sigma^2 = 3^2)$. The prior on the temporal correlation parameter ρ is chosen to be mean 0.91, with a 95% range of ρ falling between 0.86 and 0.93. This high correlation prior is justified by a lack of large changes over short periods of time in education, suggesting high correlation of values over time. The priors on the random effects variances were chosen to be relatively loose given that we believe our fixed effects covariates should be well correlated with our outcome of interest, which might suggest relatively small random effects values. The priors used for the nugget and the country random effects are the default in INLA. We have run a series of sensitivity tests comparing our predictions between the default INLA gamma priors and the uniform prior. Due to close concordance in predictions (=0.97) and no meaningful difference identified in the fit statistics, we have decided to maintain the default

priors. At the same time, we wanted to avoid using a prior that was so diffuse as to actually put high prior weight on large random effect variances. For stability, we used the uncorrelated multivariate normal priors that INLA automatically determines (based on the finite elements mesh) for the log-transformed spatial hyperparameters κ and τ. The mean and variance parameters for the hyperpriors selected by INLA for the meshes in each region can be found in Supplementary Table 4. In our parameterization we represent α and γ in the *gamma* distribution as scale and shape, respectively.

After fitting each model, we generated 1,000 draws of all model parameters from the approximated joint posterior distribution using the inla.posterior.sample() function in R-INLA. For each binomial indicator draw s of the model parameters we constructed a draw of p_i as:

$$p_i^{(s)} = \text{logit}^{-1}\left(\beta_0^{(s)} + \boldsymbol{\beta}_1^{(s)}\boldsymbol{X}_i + \epsilon_{\boldsymbol{i}}^{\boldsymbol{CTRY}\,(s)} + \epsilon_{\boldsymbol{i}}^{\boldsymbol{GP}\,(s)} + \epsilon_{\boldsymbol{i}}^{\boldsymbol{nug}\,(s)}\right)$$

The construction of draws for Gaussian indicators is similar:

$$p_i^{(s)} = \beta_0^{(s)} + \boldsymbol{\beta}_1^{(s)}\boldsymbol{X}_i + \epsilon_{\boldsymbol{i}}^{\boldsymbol{CTRY}\,(s)} + \epsilon_{\boldsymbol{i}}^{\boldsymbol{GP}\,(s)} + \epsilon_{\boldsymbol{i}}^{\boldsymbol{nug}\,(s)}$$

Additional processing of the output from inla.posterior.sample() is required for the spatial-temporal random effect ($\epsilon_{\boldsymbol{i}}^{\boldsymbol{GP}\,(s)}$) and the nugget effect $\epsilon_{\boldsymbol{i}}^{\boldsymbol{nug}\,(s)}$) prior to constructing $p_i^{(s)}$ according to the equation above. Specifically, for $\epsilon_{\boldsymbol{i}}^{\boldsymbol{GP}\,(s)}$, draws are generated initially only at vertices of the finite element mesh, so we project from this mesh to each location-year combination of (i) desired for prediction, i.e., the centroid of each grid cell on a 5 × 5-km^2 grid as well as all years from 2000 to 2017. For the nugget effect, we generate $\epsilon_{\boldsymbol{i}}^{\boldsymbol{nug}\,(s)}$ for each location-year combination of (i) by sampling from $\text{Normal}\left(0, {\sigma_{nugget}^2}^{(s)}\right)$. At the end of this process, we have 1,000 draws of p_i for each grid cell and year combination.

Supplementary Table 6. Spatial hyperparameter priors by region

Region	$\boldsymbol{\mu_{\theta_1}}$	$\boldsymbol{\sigma^2_{\theta_1}}$	$\boldsymbol{\mu_2}$	$\boldsymbol{\sigma^2_{\theta_2}}$
**East Asia**	0.3779940	10	-1.6435062	10
**Southeast Asia**	-0.3667678	10	-0.8987443	10
**Oceania**	0.5420207	10	-1.8075328	10
**South Asia**	0.2314676	10	-1.4969797	10
**Tropical South America**	0.1287355	10	-1.3942477	10
**Andean South America**	0.0516064	10	-1.3171185	10
**Central America & Caribbean**	0.3250544	10	-1.5905666	10
**North Africa**	0.2997445	10	-1.5652567	10
**West Sub-Saharan Africa**	0.2006548	10	-1.4661669	10
**Central Sub-Saharan Africa**	-0.2024953	10	-1.0630168	10
**Eastern Sub-Saharan Africa**	0.1372834	10	-1.4027955	10
**Southern Sub-Saharan Africa**	-0.1459584	10	-1.1195537	10
**Middle East**	-0.2211790	10	-1.0443331	10

**Central Asia**	-0.2064342	10	-1.0590779	10

5.2.5 Mesh construction

We constructed the finite elements mesh for the stochastic partial differential equation approximation to the Gaussian process regression using a simplified polygon boundary (in which coastlines and complex boundaries were smoothed) for each of the regions within our model[20]. This paper uses an improved mesh that is constructed on the S2 domain. This allows distance to be calculated along the sphere instead of using Euclidean distance between latitude and longitude coordinates. This mesh also generates denser vertices in data rich areas. We set the minimum triangle edge length to 25 kilometers, the maximum triangle length to 1000 kilometers, with the mesh extending 500 kilometers past the region's boundary. An example of finite elements mesh-constructed for East Asia mesh can be found in Supplementary Fig. 7.

5.2.6 Model fitting and estimate generation

Models were fit in INLA with methods consistent with those used in geospatial modelling of under-5 mortality, published previously[21].

Resampling K-means weights (Supplementary Methods 5.2) were used within the INLA fit by multiplying the corresponding log-likelihood evaluation for the specific observation by the observation's K-means weight. Data points that could be georeferenced to latitude-longitude locations were assigned a weight of 1. For cluster locations generated based on the polygon resampling process, the log-likelihood of those points contributed proportionate to the K-means weights, effectively diffusing the evaluation of the observation across the polygon.

As part of the ensemble modelling process, prediction surfaces from the out-of-sample ensemble sub-models were used as covariates in the spatiotemporal model. Estimates of the fixed effects beta coefficients derived from the contribution of each of the sub-models to INLA's predicted prevalence estimates, in conjunction with parameter estimates of the contribution of location and time (based on estimated parameters described in model description in the Supplementary Methods 5.3.3), were generated and can be found in Supplementary Tables 5–7. To create final estimates, the in-sample prediction surfaces of prevalence from the sub-models (serving as covariates) were used to calculate estimates of prevalence for each pixel in each year.

All estimates were generated by taking 1,000 draws from the posterior distribution, which yielded 1,000 candidate maps used to summarise the pixel- and aggregated-level statistics. For estimates at the pixel level, these draws were used directly to generate estimates and uncertainty. Aggregated estimates, in which estimates at the pixel level were summarised to administrative boundaries, were generated by creating population-weighted averages for each administrative boundary, for each draw.

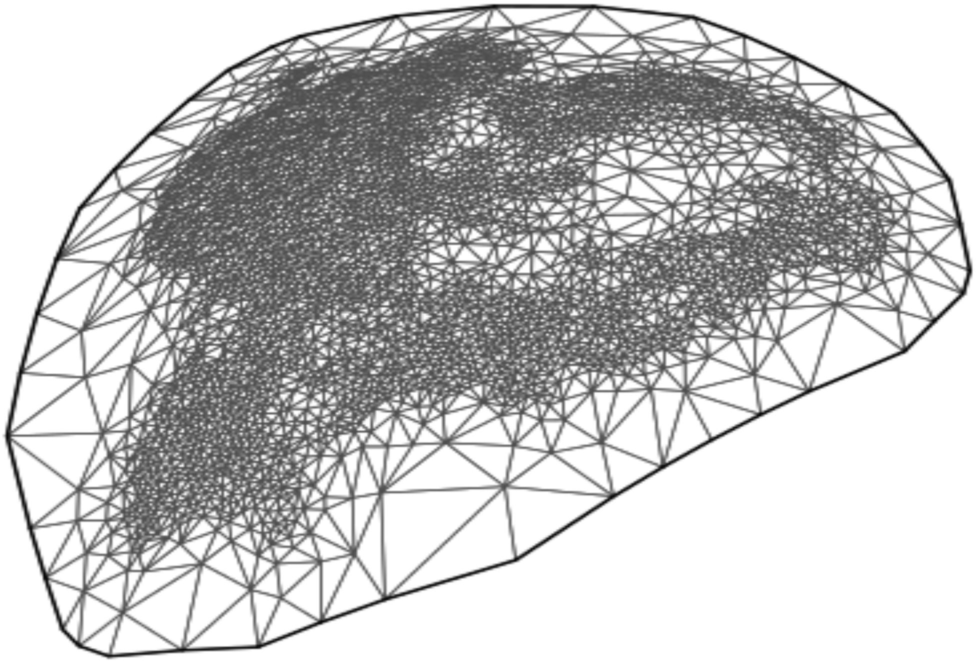

Supplementary Figure 7. Finite elements mesh
The finite elements mesh used to fit the space-time correlated error for the East Asia (EAAS) region. Both the fine-scale mesh over land in the modelling region and the coarser buffer region mesh are shown.

5.2.7 Model Results

Fitted parameters and hyperparameters, as well as their 95% uncertainty intervals are shown by indicator and region in Supplementary Tables 8–23. Spatial hyperparameters (τ and κ) and their uncertainties have been transformed into more interpretable nominal variance and range parameters. Nominal variance, approximating the variance at any single point, is calculated as $nom.var = 4\pi\kappa^2\tau^2$, and nominal range, approximating the distance before spatial correlation decays by 90%, as $range = \sqrt{8}/\kappa$.[

Supplementary Table 7. List of all modeled outcomes and the populations for which they are modelled

Indicator	Population
**Proportion of population with zero years of education**	Women, age 15–49
	Men, age 15–49
	Women, age 20–24
	Men, age 20–24
**Proportion of population with 1–5 years of education**	Women, age 15–49
	Men, age 15–49
	Women, age 20–24
	Men, age 20–24
**Proportion of population with 6–11 years of education**	Women, age 15-49
	Men, age 15–49
	Women, age 20–24
	Men, age 20–24
**Proportion of population with 12+ years of education** **This is indirectly estimated as the complement of the sum of indicators 1–3*	Women, age 15-49
	Men, age 15-49
	Women, age 20–24
	Men, age 20–24
**Mean years of educational attainment**	Women, age 15–49
	Men, age 15–49
	Women, age 20–24
	Men, age 20–24

Supplementary Table 8. Proportion with zero years, women ages 15–49, fitted parameters

Lower, median, and upper quantiles (0.025%, 0.50%, 0.975%) are displayed for the main parameters by region. The fixed effects covariates corresponding to the predicted ensemble rasters are shown in the first four columns, while fitted values for the spatiotemporal field hyperparameters and the precisions (inverse variance) for our random effects are shown in the last five columns.

		int	gam	gbm	lasso	Nominal Range	Nominal Variance	AR1 rho	Precision for IID.ID	Precision for CTRY.ID
Central sub-Saharan Africa quantiles	0.025	-1.749	2.151	1.163	-3.645	0.050	1.156	0.945	8.090	1.677
	0.5	-1.359	2.502	1.675	-3.177	0.056	1.404	0.961	8.964	3.873
	0.975	-0.970	2.853	2.186	-2.710	0.063	1.848	0.982	10.924	8.058
Eastern sub-Saharan Africa quantiles	0.025	-3.239	-0.150	0.628	-0.389	0.068	2.214	0.938	5.752	0.051
	0.5	-2.474	0.023	0.987	-0.010	0.077	2.731	0.947	5.981	0.845
	0.975	-1.709	0.196	1.345	0.369	0.084	3.226	0.953	6.235	4.358
Northern Africa quantiles	0.025	-1.330	0.218	1.512	-1.433	0.046	0.785	0.932	5.222	1.202
	0.5	-0.948	0.440	1.733	-1.173	0.049	0.882	0.939	5.333	5.245
	0.975	-0.566	0.661	1.953	-0.912	0.053	1.007	0.949	5.472	15.918
Southern sub-Saharan Africa quantiles	0.025	-3.125	0.208	-0.550	0.441	0.099	1.637	0.942	20.048	0.504
	0.5	-2.238	0.524	-0.265	0.741	0.112	2.115	0.952	21.237	2.103
	0.975	-1.351	0.840	0.019	1.041	0.128	2.725	0.960	22.256	5.162
Western sub-Saharan quantiles	0.025	-2.359	1.184	-0.958	-0.233	0.042	0.629	0.916	22.834	0.298
	0.5	-1.507	1.386	-0.552	0.165	0.048	0.750	0.928	24.625	0.886
	0.975	-0.655	1.589	-0.146	0.564	0.054	0.896	0.940	26.934	2.540
Middle East quantiles	0.025	-4.140	2.041	0.887	-3.116	0.089	4.321	0.989	5.141	4.113
	0.5	-2.901	2.378	1.332	-2.709	0.107	6.305	0.993	5.316	5.874
	0.975	-1.663	2.715	1.776	-2.304	0.130	9.127	0.994	5.515	9.663
Central Asia quantiles	0.025	-2.669	2.121	-1.060	-1.524	0.053	1.176	0.876	1.846	0.399
	0.5	-1.976	2.621	-0.635	-0.985	0.058	1.393	0.899	1.913	1.133
	0.975	-1.284	3.120	-0.211	-0.445	0.066	1.738	0.922	1.983	2.734
South Asia quantiles	0.025	-1.527	0.692	0.425	-0.634	0.081	2.889	0.958	4.770	1.233
	0.5	-0.893	0.816	0.636	-0.451	0.087	3.340	0.963	4.912	2.252
	0.975	-0.260	0.939	0.847	-0.269	0.096	3.976	0.968	5.073	4.772
East Asia quantiles	0.025	-2.312	1.725	1.683	-3.406	0.154	3.418	0.982	4.153	0.300
	0.5	-0.770	2.031	2.024	-3.054	0.197	5.479	0.987	4.322	0.713
	0.975	0.771	2.337	2.364	-2.703	0.264	9.683	0.992	4.456	1.741
Southeast Asia quantiles	0.025	-2.312	1.725	1.683	-3.406	0.154	3.418	0.982	4.153	0.300
	0.5	-0.770	2.031	2.024	-3.054	0.197	5.479	0.987	4.322	0.713
	0.975	0.771	2.337	2.364	-2.703	0.264	9.683	0.992	4.456	1.741
Oceania quantiles	0.025	-2.312	1.725	1.683	-3.406	0.154	3.418	0.982	4.153	0.300
	0.5	-0.770	2.031	2.024	-3.054	0.197	5.479	0.987	4.322	0.713
	0.975	0.771	2.337	2.364	-2.703	0.264	9.683	0.992	4.456	1.741
	0.025	-2.312	1.725	1.683	-3.406	0.154	3.418	0.982	4.153	0.300
	0.5	-0.770	2.031	2.024	-3.054	0.197	5.479	0.987	4.322	0.713

		int	gam	gbm	lasso	Nominal Range	Nominal Variance	AR1 rho	Precision for IID.ID	Precision for CTRY.ID
Andean South America quantiles	0.975	0.771	2.337	2.364	-2.703	0.264	9.683	0.992	4.456	1.741
Tropical South America quantiles	0.025	-3.606	0.117	-0.083	0.198	0.067	0.594	0.887	1.630	0.077
	0.5	-2.129	0.330	0.185	0.485	0.087	0.906	0.926	1.742	0.241
	0.975	-0.654	0.543	0.453	0.771	0.105	1.272	0.948	1.825	0.639
Central America and Caribbean quantiles	0.025	-3.765	3.671	2.935	-7.868	0.070	1.140	0.906	13.623	0.367
	0.5	-3.097	4.163	3.819	-6.981	0.076	1.300	0.916	14.932	0.989
	0.975	-2.431	4.653	4.702	-6.095	0.085	1.553	0.929	15.874	2.547

Supplementary Table 9. Proportion with 1–5 years, women ages 15–49 fitted parameters

Lower, median, and upper quantiles (0.025%, 0.50%, 0.975%) are displayed for the main parameters by region. The fixed effects covariates corresponding to the predicted ensemble rasters are shown in the first four columns, while fitted values for the spatiotemporal field hyperparameters and the precisions (inverse variance) for our random effects are shown in the last five columns.

		int	gam	gbm	lasso	Nominal Range	Nominal Variance	AR1 rho	Precision for IID.ID	Precision for CTRY.ID
Central sub-Saharan Africa quantiles	0.025	-1.048	1.461	1.667	-3.276	0.045	0.683	0.918	5.271	0.660
	0.5	-0.619	1.782	2.090	-2.873	0.053	0.849	0.936	5.688	1.564
	0.975	-0.191	2.103	2.514	-2.470	0.062	1.073	0.951	6.044	3.069
Eastern sub-Saharan Africa quantiles	0.025	-2.920	1.410	2.415	-4.713	0.131	3.562	0.981	11.000	1.782
	0.5	-1.817	1.802	3.202	-4.004	0.151	4.681	0.985	11.471	6.097
	0.975	-0.716	2.194	3.988	-3.296	0.180	6.516	0.988	12.052	25.315
Northern Africa quantiles	0.025	-1.839	0.387	1.396	-1.782	0.057	0.589	0.886	9.186	1.461
	0.5	-1.385	0.602	1.780	-1.381	0.063	0.702	0.904	9.554	4.151
	0.975	-0.931	0.816	2.163	-0.981	0.071	0.870	0.925	9.964	14.198
Southern sub-Saharan Africa quantiles	0.025	-2.575	-0.004	-1.964	1.447	0.094	0.977	0.969	34.790	0.584
	0.5	-1.739	0.528	-1.485	1.957	0.110	1.307	0.976	37.092	1.538
	0.975	-0.903	1.059	-1.006	2.467	0.127	1.783	0.982	39.145	3.520
Western sub-Saharan quantiles	0.025	-2.145	0.715	-0.022	-0.998	0.066	0.747	0.920	30.750	2.941
	0.5	-1.643	1.075	0.446	-0.521	0.078	1.010	0.935	33.503	22.946
	0.975	-1.142	1.436	0.914	-0.044	0.091	1.359	0.948	36.685	174.247
	0.025	-2.684	2.169	0.038	-2.159	0.114	3.840	0.990	6.301	0.713

		int	gam	gbm	lasso	Nominal Range	Nominal Variance	AR1 rho	Precision for IID.ID	Precision for CTRY.ID
**Middle East quantiles**	0.5	-1.351	2.477	0.388	-1.865	0.133	5.269	0.992	6.512	2.067
	0.975	-0.019	2.785	0.737	-1.571	0.155	7.105	0.994	6.704	4.907
**Central Asia quantiles**	0.025	-1.859	1.960	0.022	-1.758	0.047	0.617	0.903	7.421	0.420
	0.5	-1.213	2.217	0.268	-1.486	0.054	0.792	0.923	7.933	1.139
	0.975	-0.568	2.474	0.514	-1.213	0.065	1.076	0.943	8.372	2.632
**South Asia quantiles**	0.025	-1.859	1.960	0.022	-1.758	0.047	0.617	0.903	7.421	0.420
	0.5	-1.213	2.217	0.268	-1.486	0.054	0.792	0.923	7.933	1.139
	0.975	-0.568	2.474	0.514	-1.213	0.065	1.076	0.943	8.372	2.632
**East Asia quantiles**	0.025	-1.676	0.176	0.438	-0.874	0.053	0.217	0.804	11.553	0.406
	0.5	-1.149	0.545	0.894	-0.439	0.066	0.272	0.887	12.633	0.946
	0.975	-0.623	0.914	1.350	-0.004	0.077	0.335	0.918	14.654	1.919
**Southeast Asia quantiles**	0.025	-1.273	1.563	1.678	-3.085	0.089	0.866	0.899	9.902	1.268
	0.5	-0.841	1.781	1.996	-2.777	0.102	1.118	0.918	10.394	2.431
	0.975	-0.409	1.998	2.316	-2.470	0.118	1.523	0.937	11.042	4.292
**Oceania quantiles**	0.025	-1.273	1.563	1.678	-3.085	0.089	0.866	0.899	9.902	1.268
	0.5	-0.841	1.781	1.996	-2.777	0.102	1.118	0.918	10.394	2.431
	0.975	-0.409	1.998	2.316	-2.470	0.118	1.523	0.937	11.042	4.292
**Andean South America quantiles**	0.025	-2.303	-4.980	-0.127	0.948	0.161	1.921	0.669	87.974	0.576
	0.5	-1.529	-2.589	0.313	3.277	0.186	2.537	0.753	93.858	1.612
	0.975	-0.755	-0.200	0.750	5.604	0.209	3.238	0.815	99.985	4.113
**Tropical South America quantiles**	0.025	-3.492	1.272	-0.226	-1.314	0.127	0.857	0.853	5.103	0.234
	0.5	-2.319	1.691	0.151	-0.841	0.174	1.540	0.916	5.458	0.931
	0.975	-1.146	2.111	0.524	-0.369	0.241	2.830	0.952	5.814	2.839
**Central America and Caribbean quantiles**	0.025	-2.155	1.255	0.589	-1.606	0.058	0.651	0.915	16.379	0.623
	0.5	-1.783	1.413	0.903	-1.317	0.066	0.806	0.930	17.343	3.216
	0.975	-1.411	1.572	1.217	-1.028	0.073	0.960	0.940	18.196	9.507

Supplementary Table 10. Proportion with 6–11 years, women ages 15–49 fitted parameters

Lower, median, and upper quantiles (0.025%, 0.50%, 0.975%) are displayed for the main parameters by region. The fixed effects covariates corresponding to the predicted ensemble rasters are shown in the first four columns, while fitted values for the spatiotemporal field hyperparameters and the precisions (inverse variance) for our random effects are shown in the last five columns.

		int	gam	gbm	lasso	Nominal Range	Nominal Variance	AR1 rho	Precision for IID.ID	Precision for CTRY.ID
Central sub-Saharan Africa quantiles	0.025	1.229	4.391	4.636	-10.613	0.037	0.281	0.925	2.852	2.184
	0.5	1.507	5.193	5.527	-9.720	0.046	0.352	0.950	3.056	4.491
	0.975	1.785	5.995	6.417	-8.828	0.059	0.455	0.974	3.271	10.561
Eastern sub-Saharan Africa quantiles	0.025	-0.590	-2.100	1.419	0.925	0.104	1.475	0.968	12.431	0.139
	0.5	0.693	-1.897	1.674	1.223	0.128	2.124	0.977	12.999	0.471
	0.975	1.975	-1.694	1.929	1.522	0.153	2.964	0.983	13.512	1.434
Northern Africa quantiles	0.025	0.342	2.102	2.241	-4.206	0.039	0.283	0.741	6.140	1.667
	0.5	0.612	2.329	2.554	-3.883	0.044	0.340	0.813	6.340	8.005
	0.975	0.882	2.556	2.866	-3.561	0.048	0.391	0.847	6.503	24.527
Southern sub-Saharan Africa quantiles	0.025	0.345	-0.665	-0.171	1.090	0.083	0.602	0.967	44.009	0.716
	0.5	1.103	-0.445	0.064	1.381	0.104	0.970	0.981	46.242	1.976
	0.975	1.860	-0.226	0.299	1.673	0.122	1.343	0.986	48.770	4.471
Western sub-Saharan quantiles	0.025	-0.206	0.529	0.673	-1.274	0.041	0.209	0.902	39.380	5.646
	0.5	0.034	0.812	1.064	-0.876	0.047	0.259	0.920	43.680	29.229
	0.975	0.274	1.095	1.455	-0.480	0.054	0.323	0.936	48.713	150.100
Middle East quantiles	0.025	-1.432	1.214	0.637	-1.807	0.139	6.502	0.994	6.484	0.946
	0.5	0.480	1.520	0.965	-1.485	0.164	9.069	0.996	6.651	2.323
	0.975	2.390	1.826	1.292	-1.163	0.195	13.155	0.997	6.785	5.765
Central Asia quantiles	0.025	0.216	-4.613	2.008	2.114	0.066	0.452	0.811	13.550	1.538
	0.5	0.664	-4.084	2.287	2.797	0.082	0.663	0.864	14.473	3.993
	0.975	1.111	-3.556	2.567	3.480	0.104	1.000	0.904	15.384	9.810
South Asia quantiles	0.025	1.036	3.866	0.074	-3.897	0.056	1.110	0.963	4.511	1.683
	0.5	1.449	4.180	0.377	-3.556	0.065	1.470	0.972	4.674	2.550
	0.975	1.861	4.493	0.680	-3.216	0.079	2.004	0.979	4.801	4.113
East Asia quantiles	0.025	-0.536	1.172	1.004	-2.412	0.177	1.157	0.965	7.836	1363.578
	0.5	0.380	1.598	1.378	-1.976	0.248	2.138	0.980	8.522	12361.429
	0.975	1.300	2.020	1.750	-1.540	0.424	5.790	0.993	9.200	111000.000
Southeast Asia quantiles	0.025	0.666	2.891	3.478	-6.574	0.086	0.478	0.832	8.613	1.745
	0.5	0.963	3.307	3.885	-6.192	0.102	0.619	0.868	9.051	4.596
	0.975	1.261	3.722	4.292	-5.811	0.123	0.847	0.902	9.600	8.991
Oceania quantiles	0.025	0.666	2.891	3.478	-6.574	0.086	0.478	0.832	8.613	1.745
	0.5	0.963	3.307	3.885	-6.192	0.102	0.619	0.868	9.051	4.596
	0.975	1.261	3.722	4.292	-5.811	0.123	0.847	0.902	9.600	8.991
Andean South America quantiles	0.025	0.152	2.630	1.039	-5.683	0.069	0.411	-0.304	73.312	1.250
	0.5	0.498	3.505	1.843	-4.348	0.075	0.476	0.216	77.804	3.561
	0.975	0.845	4.380	2.647	-3.016	0.080	0.528	0.471	84.941	8.403

		int	gam	gbm	lasso	Nominal Range	Nominal Variance	AR1 rho	Precision for IID.ID	Precision for CTRY.ID
Tropical South America quantiles	0.025	0.072	0.424	1.473	-2.543	0.054	0.353	0.909	6.640	4.856
	0.5	0.483	0.870	2.019	-1.889	0.074	0.583	0.945	7.498	9.511
	0.975	0.895	1.315	2.565	-1.236	0.108	1.039	0.970	8.119	18.778
Central America and Caribbean quantiles	0.025	-0.597	1.662	-0.308	-1.365	0.154	2.053	0.969	12.293	0.701
	0.5	0.435	1.943	0.080	-1.023	0.179	2.787	0.976	13.003	1.859
	0.975	1.467	2.224	0.468	-0.682	0.210	3.951	0.983	14.371	4.361

Supplementary Table 11. Proportion with zero years, men ages 15–49 fitted parameters

Lower, median, and upper quantiles (0.025%, 0.50%, 0.975%) are displayed for the main parameters by region. The fixed effects covariates corresponding to the predicted ensemble rasters are shown in the first four columns, while fitted values for the spatiotemporal field hyperparameters and the precisions (inverse variance) for our random effects are shown in the last five columns.

		int	gam	gbm	lasso	Nominal Range	Nominal Variance	AR1 rho	Precision for IID.ID	Precision for CTRY.ID
Central sub-Saharan Africa quantiles	0.025	-2.588	2.598	1.866	-5.588	0.067	1.349	0.959	13.215	1.844
	0.5	-2.044	3.167	2.649	-4.815	0.080	1.817	0.970	14.446	4.099
	0.975	-1.502	3.735	3.430	-4.043	0.094	2.515	0.979	16.185	8.920
Eastern sub-Saharan Africa quantiles	0.025	-3.484	-0.235	1.395	-1.346	0.050	1.291	0.925	5.911	0.747
	0.5	-2.853	-0.006	1.867	-0.861	0.058	1.680	0.943	6.224	2.531
	0.975	-2.223	0.223	2.338	-0.377	0.065	2.041	0.953	6.796	9.800
Northern Africa quantiles	0.025	-2.165	0.272	0.971	-1.268	0.049	0.650	0.904	5.895	1.242
	0.5	-1.701	0.522	1.349	-0.870	0.053	0.737	0.914	6.091	3.464
	0.975	-1.239	0.770	1.726	-0.472	0.056	0.815	0.922	6.235	9.762
Southern sub-Saharan Africa quantiles	0.025	-3.050	-0.144	-0.813	0.890	0.102	1.727	0.946	21.804	0.660
	0.5	-2.108	0.240	-0.474	1.234	0.116	2.237	0.956	23.067	2.225
	0.975	-1.166	0.624	-0.135	1.577	0.134	2.978	0.964	24.347	5.520
Western sub-Saharan quantiles	0.025	-3.242	1.599	-1.014	-1.784	0.038	0.398	0.904	27.657	0.222
	0.5	-2.320	2.072	-0.143	-0.928	0.042	0.476	0.919	31.013	0.662
	0.975	-1.398	2.544	0.728	-0.074	0.047	0.576	0.935	34.542	1.803
Middle East quantiles	0.025	-5.123	1.462	2.295	-4.310	0.140	9.462	0.997	6.464	2.451
	0.5	-2.665	1.893	2.875	-3.767	0.176	14.899	0.998	6.649	4.655
	0.975	-0.209	2.323	3.455	-3.226	0.224	24.514	0.998	6.890	7.040
	0.025	-3.362	1.182	-0.907	-1.044	0.079	1.401	0.885	2.117	0.503

		int	gam	gbm	lasso	Nominal Range	Nominal Variance	AR1 rho	Precision for IID.ID	Precision for CTRY.ID
**Central Asia quantiles**	0.5	-2.515	1.791	-0.365	-0.424	0.095	1.864	0.911	2.196	1.375
	0.975	-1.669	2.399	0.176	0.195	0.110	2.447	0.933	2.262	3.529
**South Asia quantiles**	0.025	-2.431	0.673	0.242	-0.556	0.106	3.839	0.963	4.922	1.026
	0.5	-1.470	0.839	0.494	-0.333	0.120	4.777	0.969	5.054	1.974
	0.975	-0.510	1.005	0.746	-0.110	0.131	5.670	0.972	5.221	4.479
**East Asia quantiles**	0.025	-3.081	2.131	0.778	-3.371	0.161	2.842	0.979	5.410	0.580
	0.5	-1.516	2.611	1.226	-2.837	0.213	5.024	0.988	5.771	1.111
	0.975	0.048	3.090	1.673	-2.303	0.292	9.200	0.993	6.063	2.370
**Southeast Asia quantiles**	0.025	-1.407	1.901	0.553	-2.206	0.074	1.315	0.886	2.165	1.760
	0.5	-0.959	2.091	0.839	-1.930	0.083	1.534	0.909	2.204	2.271
	0.975	-0.511	2.280	1.124	-1.654	0.090	1.771	0.922	2.261	3.037
**Oceania quantiles**	0.025	-1.407	1.901	0.553	-2.206	0.074	1.315	0.886	2.165	1.760
	0.5	-0.959	2.091	0.839	-1.930	0.083	1.534	0.909	2.204	2.271
	0.975	-0.511	2.280	1.124	-1.654	0.090	1.771	0.922	2.261	3.037
**Andean South America quantiles**	0.025	-3.880	-1.577	-0.323	-0.629	0.136	1.756	0.789	109.194	0.238
	0.5	-2.995	-0.105	0.250	0.856	0.152	2.177	0.827	115.886	0.641
	0.975	-2.111	1.366	0.822	2.339	0.174	2.719	0.856	125.628	1.653
**Tropical South America quantiles**	0.025	-3.737	0.151	-0.236	-0.011	0.041	0.356	0.847	5.191	0.093
	0.5	-2.497	0.450	0.159	0.392	0.049	0.455	0.894	5.420	0.300
	0.975	-1.258	0.749	0.553	0.794	0.061	0.566	0.922	5.687	0.722
**Central America and Caribbean quantiles**	0.025	-4.045	2.190	2.961	-7.429	0.076	0.960	0.922	14.841	0.474
	0.5	-3.431	2.692	4.373	-6.065	0.089	1.251	0.942	15.858	1.292
	0.975	-2.818	3.194	5.783	-4.702	0.109	1.801	0.963	16.878	3.089

Supplementary Table 12. Proportion with 1–5 years, men ages 15–49 fitted parameters

Lower, median, and upper quantiles (0.025%, 0.50%, 0.975%) are displayed for the main parameters by region. The fixed effects covariates corresponding to the predicted ensemble rasters are shown in the first four columns, while fitted values for the spatiotemporal field hyperparameters and the precisions (inverse variance) for our random effects are shown in the last five columns.

		int	gam	gbm	lasso	Nominal Range	Nominal Variance	AR1 rho	Precision for IID.ID	Precision for CTRY.ID
**Central sub-Saharan Africa quantiles**	0.025	-1.489	1.683	1.277	-3.132	0.053	0.811	0.932	8.416	1.072
	0.5	-1.074	2.000	1.694	-2.694	0.063	1.093	0.954	9.500	2.421
	0.975	-0.659	2.317	2.111	-2.257	0.079	1.704	0.978	10.333	5.212
**Eastern sub-Saharan**	0.025	-3.035	1.070	4.191	-5.714	0.123	2.767	0.973	10.562	6.322
	0.5	-2.038	1.329	4.809	-5.138	0.146	3.931	0.981	11.194	12.713
	0.975	-1.041	1.588	5.426	-4.562	0.174	5.450	0.985	11.693	34.822

		int	gam	gbm	lasso	Nominal Range	Nominal Variance	AR1 rho	Precision for IID.ID	Precision for CTRY.ID
Africa quantiles										
Northern Africa quantiles	0.025	-1.826	0.595	0.828	-1.566	0.059	0.566	0.892	7.326	0.589
	0.5	-1.466	0.852	1.259	-1.110	0.064	0.660	0.906	7.686	3.258
	0.975	-1.106	1.108	1.690	-0.655	0.071	0.789	0.920	7.961	14.658
Southern sub-Saharan Africa quantiles	0.025	-2.351	-0.053	-1.005	0.797	0.085	0.899	0.969	37.365	0.823
	0.5	-1.633	0.355	-0.586	1.231	0.097	1.184	0.975	39.502	2.126
	0.975	-0.916	0.761	-0.166	1.664	0.113	1.634	0.981	42.022	4.893
Western sub-Saharan quantiles	0.025	-2.314	0.291	0.687	-1.597	0.067	0.528	0.899	39.429	1086.605
	0.5	-1.960	0.796	1.240	-1.030	0.078	0.674	0.918	43.600	11800.000
	0.975	-1.610	1.300	1.789	-0.471	0.091	0.896	0.937	48.180	76273.200
Middle East quantiles	0.025	-3.620	1.778	0.489	-2.217	0.153	7.816	0.997	6.881	1.427
	0.5	-1.334	2.090	0.840	-1.930	0.187	11.634	0.998	7.079	3.136
	0.975	0.950	2.401	1.191	-1.644	0.234	18.449	0.998	7.236	8.616
Central Asia quantiles	0.025	-2.183	0.982	-0.051	-0.698	0.052	0.677	0.921	6.845	0.422
	0.5	-1.416	1.246	0.212	-0.459	0.069	1.090	0.942	7.472	1.055
	0.975	-0.649	1.510	0.476	-0.219	0.090	1.705	0.958	7.907	2.702
South Asia quantiles	0.025	-2.183	0.982	-0.051	-0.698	0.052	0.677	0.921	6.845	0.422
	0.5	-1.416	1.246	0.212	-0.459	0.069	1.090	0.942	7.472	1.055
	0.975	-0.649	1.510	0.476	-0.219	0.090	1.705	0.958	7.907	2.702
East Asia quantiles	0.025	-1.835	-0.044	0.370	-0.597	0.050	0.153	0.844	10.801	0.564
	0.5	-1.366	0.325	0.839	-0.164	0.063	0.226	0.893	11.693	1.252
	0.975	-0.899	0.693	1.309	0.270	0.077	0.298	0.921	12.981	2.613
Southeast Asia quantiles	0.025	-1.835	-0.044	0.370	-0.597	0.050	0.153	0.844	10.801	0.564
	0.5	-1.366	0.325	0.839	-0.164	0.063	0.226	0.893	11.693	1.252
	0.975	-0.899	0.693	1.309	0.270	0.077	0.298	0.921	12.981	2.613
Oceania quantiles	0.025	-1.835	-0.044	0.370	-0.597	0.050	0.153	0.844	10.801	0.564
	0.5	-1.366	0.325	0.839	-0.164	0.063	0.226	0.893	11.693	1.252
	0.975	-0.899	0.693	1.309	0.270	0.077	0.298	0.921	12.981	2.613
Andean South America quantiles	0.025	-2.497	1.048	-0.915	-2.495	0.166	1.990	0.770	120.364	0.381
	0.5	-1.588	2.341	-0.352	-0.989	0.191	2.635	0.820	128.681	0.995
	0.975	-0.680	3.633	0.210	0.516	0.218	3.416	0.858	137.869	2.355
Tropical South America quantiles	0.025	-3.432	0.326	-0.280	-0.693	0.107	0.421	0.676	7.355	0.309
	0.5	-2.461	0.875	0.204	-0.077	0.172	1.031	0.885	7.879	1.090
	0.975	-1.492	1.424	0.683	0.539	0.287	2.748	0.966	8.549	3.413
Central America and Caribbean quantiles	0.025	-2.220	1.084	0.442	-1.246	0.059	0.622	0.914	15.666	1.069
	0.5	-1.829	1.223	0.749	-0.972	0.066	0.745	0.927	16.554	3.243
	0.975	-1.438	1.361	1.056	-0.697	0.074	0.897	0.939	17.336	8.374

Supplementary Table 13. Proportion with 6–11 years, men ages 15–49 fitted parameters

Lower, median, and upper quantiles (0.025%, 0.50%, 0.975%) are displayed for the main parameters by region. The fixed effects covariates corresponding to the predicted ensemble rasters are shown in the first four columns, while fitted values for the spatiotemporal field hyperparameters and the precisions (inverse variance) for our random effects are shown in the last five columns.

		int	gam	gbm	lasso	Nominal Range	Nominal Variance	AR1 rho	Precision for IID.ID	Precision for CTRY.ID
Central sub-Saharan Africa quantiles	0.025	0.649	2.992	3.150	-6.857	0.043	0.231	0.884	5.533	5.079
	0.5	0.867	3.548	3.737	-6.284	0.053	0.285	0.914	5.970	7.611
	0.975	1.084	4.103	4.322	-5.711	0.074	0.366	0.943	6.478	12.046
Eastern sub-Saharan Africa quantiles	0.025	-0.572	-2.196	1.874	0.609	0.088	0.906	0.960	12.607	0.199
	0.5	0.475	-1.995	2.110	0.885	0.103	1.208	0.970	13.172	0.636
	0.975	1.522	-1.794	2.346	1.161	0.126	1.756	0.980	13.828	1.878
Northern Africa quantiles	0.025	0.286	2.413	3.469	-5.987	0.035	0.159	0.694	6.460	3.329
	0.5	0.570	2.729	3.854	-5.582	0.038	0.177	0.748	6.594	9.447
	0.975	0.855	3.044	4.238	-5.178	0.042	0.203	0.814	6.776	38.892
Southern sub-Saharan Africa quantiles	0.025	0.027	-0.895	-0.212	1.358	0.097	0.655	0.965	44.773	1.065
	0.5	0.742	-0.666	0.021	1.646	0.115	0.934	0.974	47.104	2.787
	0.975	1.456	-0.437	0.253	1.933	0.134	1.272	0.979	50.766	6.545
Western sub-Saharan quantiles	0.025	-0.324	1.196	0.036	-1.311	0.041	0.162	0.915	46.092	6.625
	0.5	-0.103	1.507	0.406	-0.913	0.047	0.201	0.931	51.269	31.698
	0.975	0.117	1.817	0.777	-0.516	0.053	0.251	0.944	56.645	152.982
Middle East quantiles	0.025	-0.662	2.041	-0.023	-2.019	0.108	4.059	0.995	6.475	1.655
	0.5	0.726	2.355	0.344	-1.699	0.132	5.957	0.996	6.826	4.041
	0.975	2.112	2.670	0.710	-1.379	0.161	8.874	0.998	7.044	9.353
Central Asia quantiles	0.025	0.130	1.637	0.938	-2.595	0.081	0.657	0.776	15.241	1.510
	0.5	0.669	2.023	1.290	-2.313	0.103	1.023	0.837	16.208	3.929
	0.975	1.207	2.408	1.642	-2.031	0.131	1.618	0.884	17.468	9.577
South Asia quantiles	0.025	0.130	1.637	0.938	-2.595	0.081	0.657	0.776	15.241	1.510
	0.5	0.669	2.023	1.290	-2.313	0.103	1.023	0.837	16.208	3.929
	0.975	1.207	2.408	1.642	-2.031	0.131	1.618	0.884	17.468	9.577
East Asia quantiles	0.025	0.130	1.637	0.938	-2.595	0.081	0.657	0.776	15.241	1.510
	0.5	0.669	2.023	1.290	-2.313	0.103	1.023	0.837	16.208	3.929
	0.975	1.207	2.408	1.642	-2.031	0.131	1.618	0.884	17.468	9.577
Southeast Asia quantiles	0.025	0.130	1.637	0.938	-2.595	0.081	0.657	0.776	15.241	1.510
	0.5	0.669	2.023	1.290	-2.313	0.103	1.023	0.837	16.208	3.929
	0.975	1.207	2.408	1.642	-2.031	0.131	1.618	0.884	17.468	9.577
Oceania quantiles	0.025	0.130	1.637	0.938	-2.595	0.081	0.657	0.776	15.241	1.510
	0.5	0.669	2.023	1.290	-2.313	0.103	1.023	0.837	16.208	3.929
	0.975	1.207	2.408	1.642	-2.031	0.131	1.618	0.884	17.468	9.577
Andean South	0.025	0.130	1.637	0.938	-2.595	0.081	0.657	0.776	15.241	1.510
	0.5	0.669	2.023	1.290	-2.313	0.103	1.023	0.837	16.208	3.929
	0.975	1.207	2.408	1.642	-2.031	0.131	1.618	0.884	17.468	9.577

		int	gam	gbm	lasso	Nominal Range	Nominal Variance	AR1 rho	Precision for IID.ID	Precision for CTRY.ID
America quantiles										
Tropical South America quantiles	0.025	-0.004	0.042	1.382	-2.062	0.051	0.278	0.923	7.015	5.267
	0.5	0.349	0.513	1.931	-1.444	0.075	0.519	0.961	7.524	18.079
	0.975	0.702	0.984	2.479	-0.826	0.128	1.188	0.985	8.041	73.510
Central America and Caribbean quantiles	0.025	-0.087	1.410	-0.688	-0.762	0.109	1.453	0.966	10.611	0.721
	0.5	0.640	1.681	-0.284	-0.396	0.126	1.965	0.975	11.383	1.785
	0.975	1.367	1.950	0.119	-0.031	0.162	3.186	0.987	11.928	4.722

Supplementary Table 14. Proportion with zero years, women ages 20–24 fitted parameters

Lower, median, and upper quantiles (0.025%, 0.50%, 0.975%) are displayed for the main parameters by region. The fixed effects covariates corresponding to the predicted ensemble rasters are shown in the first four columns, while fitted values for the spatiotemporal field hyperparameters and the precisions (inverse variance) for our random effects are shown in the last five columns.

		int	gam	gbm	lasso	Nominal Range	Nominal Variance	AR1 rho	Precision for IID.ID	Precision for CTRY.ID
Central sub-Saharan Africa quantiles	0.025	-2.355	1.501	0.817	-3.368	0.067	1.389	0.959	18.561	1.144
	0.5	-1.730	2.014	1.613	-2.625	0.082	2.013	0.973	23.993	2.548
	0.975	-1.105	2.527	2.404	-1.882	0.100	2.850	0.981	28.764	5.555
Eastern sub-Saharan Africa quantiles	0.025	-3.842	-0.364	1.030	-1.066	0.054	1.509	0.919	6.613	0.547
	0.5	-3.168	-0.099	1.608	-0.509	0.063	1.935	0.940	7.202	2.198
	0.975	-2.496	0.167	2.185	0.048	0.071	2.389	0.954	7.935	8.488
Northern Africa quantiles	0.025	-2.160	-0.363	1.419	-1.266	0.043	1.188	0.905	10.788	4.802
	0.5	-1.810	-0.036	1.829	-0.792	0.049	1.360	0.918	11.858	21.478
	0.975	-1.461	0.291	2.239	-0.320	0.054	1.567	0.930	14.024	118.098
Southern sub-Saharan Africa quantiles	0.025	-4.003	0.146	-1.492	-0.051	0.119	2.284	0.944	19.357	0.371
	0.5	-2.618	0.965	-0.712	0.748	0.146	3.349	0.959	21.176	0.988
	0.975	-1.234	1.785	0.068	1.541	0.180	5.190	0.973	23.587	2.360
Western sub-Saharan quantiles	0.025	-3.163	0.276	-1.717	-1.650	0.047	0.634	0.903	1958.978	0.139
	0.5	-2.120	0.746	0.099	0.156	0.056	0.838	0.927	15900.000	0.506
	0.975	-1.082	1.216	1.916	1.956	0.064	1.041	0.950	77657.443	1.337
	0.025	-5.741	2.155	1.107	-4.781	0.167	14.290	0.992	4.873	1.924

		int	gam	gbm	lasso	Nominal Range	Nominal Variance	AR1 rho	Precision for IID.ID	Precision for CTRY.ID
Middle East quantiles	0.5	-2.650	2.948	1.999	-3.946	0.222	26.122	0.997	5.165	6.742
	0.975	0.438	3.740	2.894	-3.117	0.335	69.330	0.999	5.506	25.736
Central Asia quantiles	0.025	-3.688	2.112	-1.263	-2.049	0.064	1.690	0.836	1.741	0.300
	0.5	-2.672	2.839	-0.571	-1.264	0.089	2.615	0.887	1.843	0.836
	0.975	-1.657	3.565	0.118	-0.483	0.112	3.724	0.918	1.928	2.094
South Asia quantiles	0.025	-2.063	0.320	0.874	-0.810	0.092	3.859	0.977	11.094	0.641
	0.5	-1.153	0.457	1.127	-0.585	0.105	4.939	0.981	11.797	1.285
	0.975	-0.244	0.594	1.380	-0.359	0.122	6.515	0.985	12.396	2.591
East Asia quantiles	0.025	-2.402	1.217	1.431	-3.017	0.081	1.289	0.956	4.566	0.153
	0.5	-1.410	1.651	1.872	-2.523	0.121	2.519	0.977	4.800	0.506
	0.975	-0.419	2.085	2.313	-2.029	0.168	4.525	0.986	5.146	1.231
Southeast Asia quantiles	0.025	-1.131	2.290	0.382	-2.479	0.075	2.052	0.945	5.062	0.850
	0.5	-0.557	2.513	0.686	-2.199	0.082	2.472	0.956	5.310	1.852
	0.975	0.018	2.736	0.990	-1.919	0.090	2.904	0.962	5.645	3.377
Oceania quantiles	0.025	-1.131	2.290	0.382	-2.479	0.075	2.052	0.945	5.062	0.850
	0.5	-0.557	2.513	0.686	-2.199	0.082	2.472	0.956	5.310	1.852
	0.975	0.018	2.736	0.990	-1.919	0.090	2.904	0.962	5.645	3.377
Andean South America quantiles	0.025	-3.700	-0.245	0.284	0.159	0.109	2.175	0.062	66.883	0.232
	0.5	-2.813	0.066	0.552	0.382	0.123	2.717	0.274	74.146	0.653
	0.975	-1.931	0.377	0.820	0.605	0.134	3.205	0.535	79.813	1.981
Tropical South America quantiles	0.025	-4.007	0.048	-0.184	-0.085	0.049	0.715	0.862	1.928	0.085
	0.5	-2.595	0.350	0.267	0.384	0.063	1.031	0.922	2.096	0.271
	0.975	-1.184	0.652	0.717	0.852	0.076	1.409	0.952	2.246	0.778
Central America and Caribbean quantiles	0.025	-4.424	3.635	7.365	-16.247	0.070	1.131	0.856	10.902	0.681
	0.5	-3.919	4.773	9.921	-13.692	0.084	1.478	0.906	12.900	2.533
	0.975	-3.414	5.907	12.473	-11.136	0.111	2.142	0.954	14.795	8.887

Supplementary Table 15. Proportion with 1–5 years, women ages 20–24 fitted parameters

Lower, median, and upper quantiles (0.025%, 0.50%, 0.975%) are displayed for the main parameters by region. The fixed effects covariates corresponding to the predicted ensemble rasters are shown in the first four columns, while fitted values for the spatiotemporal field hyperparameters and the precisions (inverse variance) for our random effects are shown in the last five columns.

		int	gam	gbm	lasso	Nominal Range	Nominal Variance	AR1 rho	Precision for IID.ID	Precision for CTRY.ID
Central sub-Saharan Africa quantiles	0.025	-1.361	1.933	0.901	-3.473	0.040	0.577	0.906	13.977	0.915
	0.5	-0.949	2.378	1.514	-2.893	0.052	0.729	0.935	17.301	1.970
	0.975	-0.538	2.824	2.129	-2.316	0.063	0.923	0.952	25.541	4.345
Eastern sub-Saharan Africa quantiles	0.025	-5.721	1.702	1.927	-5.518	0.292	19.621	0.996	11.511	27.606
	0.5	-1.368	2.309	3.130	-4.439	0.384	34.174	0.998	12.521	65.516
	0.975	2.981	2.915	4.333	-3.361	0.516	62.652	0.999	13.605	338.168
Northern Africa quantiles	0.025	-2.553	-0.098	1.579	-1.941	0.055	0.790	0.837	28.437	0.548
	0.5	-1.979	0.212	2.143	-1.355	0.061	0.932	0.864	32.368	1.919
	0.975	-1.405	0.522	2.707	-0.769	0.068	1.130	0.894	36.976	5.499
Southern sub-Saharan Africa quantiles	0.025	-3.323	1.221	-2.410	-1.537	0.094	1.381	0.981	20.447	0.297
	0.5	-2.156	2.396	-1.239	-0.150	0.124	2.332	0.989	22.515	0.960
	0.975	-0.990	3.569	-0.070	1.228	0.167	4.145	0.994	24.863	2.225
Western sub-Saharan quantiles	0.025	-2.783	0.441	-1.400	-0.237	0.051	0.826	0.864	111.925	1.143
	0.5	-2.145	0.952	-0.564	0.614	0.075	1.293	0.917	234.303	9.209
	0.975	-1.507	1.462	0.262	1.471	0.094	1.792	0.939	623.105	72.769
Middle East quantiles	0.025	-5.226	1.331	0.724	-2.547	0.222	19.353	0.996	6.593	0.929
	0.5	-1.327	1.816	1.240	-2.056	0.274	29.524	0.997	6.956	2.744
	0.975	2.569	2.301	1.755	-1.566	0.353	50.819	0.999	7.279	6.795
Central Asia quantiles	0.025	-2.680	2.603	0.243	-3.119	0.038	0.562	0.873	8.530	0.137
	0.5	-1.860	3.062	0.609	-2.671	0.054	0.898	0.919	9.307	0.560
	0.975	-1.041	3.521	0.974	-2.224	0.070	1.306	0.944	10.110	1.374
South Asia quantiles	0.025	-2.680	2.603	0.243	-3.119	0.038	0.562	0.873	8.530	0.137
	0.5	-1.860	3.062	0.609	-2.671	0.054	0.898	0.919	9.307	0.560
	0.975	-1.041	3.521	0.974	-2.224	0.070	1.306	0.944	10.110	1.374
East Asia quantiles	0.025	-2.157	0.312	0.061	-1.391	0.072	0.300	0.749	9.630	0.260
	0.5	-1.493	0.897	0.816	-0.712	0.105	0.466	0.834	11.263	0.656
	0.975	-0.829	1.482	1.570	-0.035	0.138	0.664	0.891	12.781	1.475
Southeast Asia quantiles	0.025	-1.629	1.669	1.425	-3.284	0.094	0.790	0.902	16.382	1.412
	0.5	-1.058	1.974	1.879	-2.853	0.131	1.390	0.940	18.752	2.614
	0.975	-0.488	2.278	2.336	-2.425	0.183	2.513	0.965	21.478	4.748
Oceania quantiles	0.025	-1.629	1.669	1.425	-3.284	0.094	0.790	0.902	16.382	1.412
	0.5	-1.058	1.974	1.879	-2.853	0.131	1.390	0.940	18.752	2.614
	0.975	-0.488	2.278	2.336	-2.425	0.183	2.513	0.965	21.478	4.748
	0.025	-2.949	-0.606	-0.194	0.588	0.066	0.850	0.686	24.134	0.302
	0.5	-2.347	-0.152	0.070	1.083	0.074	1.013	0.777	25.717	1.114

		int	gam	gbm	lasso	Nominal Range	Nominal Variance	AR1 rho	Precision for IID.ID	Precision for CTRY.ID
Andean South America quantiles	0.975	-1.747	0.302	0.331	1.578	0.083	1.216	0.842	27.693	2.851
Tropical South America quantiles	0.025	-3.751	1.390	-0.353	-2.116	0.128	1.105	0.925	4.363	0.099
	0.5	-2.573	2.014	0.314	-1.324	0.181	2.017	0.963	4.921	12.044
	0.975	-1.396	2.638	0.964	-0.527	0.267	4.122	0.986	5.569	155.288
Central America and Caribbean quantiles	0.025	-3.071	2.057	1.390	-3.857	0.092	1.721	0.963	16.075	1.016
	0.5	-2.401	2.332	1.981	-3.313	0.116	2.684	0.984	17.572	3.566
	0.975	-1.732	2.607	2.571	-2.770	0.172	5.551	0.997	18.838	11.121

Supplementary Table 16. Proportion with 6–11 years, women ages 20–24 fitted parameters

Lower, median, and upper quantiles (0.025%, 0.50%, 0.975%) are displayed for the main parameters by region. The fixed effects covariates corresponding to the predicted ensemble rasters are shown in the first four columns, while fitted values for the spatiotemporal field hyperparameters and the precisions (inverse variance) for our random effects are shown in the last five columns.

		int	gam	gbm	lasso	Nominal Range	Nominal Variance	AR1 rho	Precision for IID.ID	Precision for CTRY.ID
Central sub-Saharan Africa quantiles	0.025	1.095	2.222	2.827	-6.600	0.028	0.375	0.890	5.165	2.667
	0.5	1.347	3.023	3.667	-5.690	0.038	0.474	0.926	6.097	6.450
	0.975	1.599	3.822	4.507	-4.780	0.049	0.615	0.955	7.527	16.589
Eastern sub-Saharan Africa quantiles	0.025	-1.291	0.196	0.630	-0.667	0.126	2.552	0.985	12.270	0.103
	0.5	0.212	0.421	0.932	-0.353	0.158	3.931	0.990	13.016	0.404
	0.975	1.713	0.645	1.233	-0.038	0.203	6.494	0.993	13.756	1.179
Northern Africa quantiles	0.025	-0.085	1.278	1.978	-3.300	0.039	0.331	0.735	17.254	1.349
	0.5	0.250	1.552	2.351	-2.902	0.044	0.392	0.787	19.296	5.605
	0.975	0.584	1.825	2.723	-2.506	0.049	0.452	0.822	21.780	18.279
Southern sub-Saharan Africa quantiles	0.025	0.305	-0.172	-0.121	0.461	0.057	0.370	0.955	37.259	0.538
	0.5	0.902	0.109	0.137	0.754	0.067	0.498	0.966	40.344	1.439
	0.975	1.499	0.389	0.395	1.048	0.079	0.682	0.974	43.311	3.331
Western sub-Saharan quantiles	0.025	-0.749	0.802	0.397	-1.566	0.043	0.313	0.883	60.767	2.034
	0.5	-0.387	1.136	0.906	-1.042	0.050	0.392	0.910	82.730	12.575
	0.975	-0.025	1.470	1.415	-0.519	0.059	0.494	0.932	113.272	118.979
	0.025	-1.339	1.313	0.213	-1.662	0.134	6.277	0.995	8.099	0.838
	0.5	0.493	1.654	0.630	-1.284	0.164	9.476	0.996	8.419	2.153

		int	gam	gbm	lasso	Nominal Range	Nominal Variance	AR1 rho	Precision for IID.ID	Precision for CTRY.ID
**Middle East quantiles**	0.975	2.323	1.995	1.046	-0.906	0.217	17.309	0.998	8.686	4.826
**Central Asia quantiles**	0.025	-0.114	2.094	0.385	-2.591	0.042	0.285	0.739	11.592	1.446
	0.5	0.226	2.476	0.757	-2.233	0.050	0.367	0.812	12.498	4.230
	0.975	0.565	2.857	1.129	-1.874	0.062	0.490	0.877	13.801	9.878
**South Asia quantiles**	0.025	-0.114	2.094	0.385	-2.591	0.042	0.285	0.739	11.592	1.446
	0.5	0.226	2.476	0.757	-2.233	0.050	0.367	0.812	12.498	4.230
	0.975	0.565	2.857	1.129	-1.874	0.062	0.490	0.877	13.801	9.878
**East Asia quantiles**	0.025	-0.572	1.933	0.718	-3.613	0.157	0.465	0.858	24.146	1.445
	0.5	0.019	2.639	1.276	-2.914	0.226	0.816	0.918	27.838	7.522
	0.975	0.610	3.344	1.833	-2.216	0.345	1.585	0.960	32.490	57.642
**Southeast Asia quantiles**	0.025	0.166	2.287	2.306	-4.937	0.103	0.602	0.880	14.088	1.768
	0.5	0.644	2.768	2.753	-4.521	0.134	0.944	0.920	15.757	3.683
	0.975	1.121	3.248	3.201	-4.105	0.176	1.500	0.948	17.913	7.259
**Oceania quantiles**	0.025	0.166	2.287	2.306	-4.937	0.103	0.602	0.880	14.088	1.768
	0.5	0.644	2.768	2.753	-4.521	0.134	0.944	0.920	15.757	3.683
	0.975	1.121	3.248	3.201	-4.105	0.176	1.500	0.948	17.913	7.259
**Andean South America quantiles**	0.025	-0.567	-0.130	-0.042	-0.203	0.076	0.628	-0.848	95.774	1.166
	0.5	-0.141	0.281	0.388	0.332	0.096	0.887	0.150	103.969	3.548
	0.975	0.285	0.691	0.817	0.866	0.110	1.104	0.663	111.272	10.622
**Tropical South America quantiles**	0.025	-0.423	0.418	0.612	-1.814	0.063	0.427	0.905	8.970	1.794
	0.5	0.046	0.924	1.201	-1.125	0.086	0.687	0.945	11.018	7.664
	0.975	0.514	1.429	1.790	-0.437	0.121	1.310	0.976	12.889	25.633
**Central America and Caribbean quantiles**	0.025	-0.423	0.418	0.612	-1.814	0.063	0.427	0.905	8.970	1.794
	0.5	0.046	0.924	1.201	-1.125	0.086	0.687	0.945	11.018	7.664
	0.975	0.514	1.429	1.790	-0.437	0.121	1.310	0.976	12.889	25.633

Supplementary Table 17. Proportion with zero years, men ages 20–24 fitted parameters

Lower, median, and upper quantiles (0.025%, 0.50%, 0.975%) are displayed for the main parameters by region. The fixed effects covariates corresponding to the predicted ensemble rasters are shown in the first four columns, while fitted values for the spatiotemporal field hyperparameters and the precisions (inverse variance) for our random effects are shown in the last five columns.

		int	gam	gbm	lasso	Nominal Range	Nominal Variance	AR1 rho	Precision for IID.ID	Precision for CTRY.ID
**Central sub-Saharan**	0.025	-3.217	2.242	-0.395	-4.313	0.122	1.549	0.961	19.928	1.912
	0.5	-2.225	3.172	0.873	-3.043	0.157	2.446	0.976	23.879	4.535
	0.975	-1.234	4.102	2.139	-1.777	0.207	4.258	0.986	30.630	10.275

		int	gam	gbm	lasso	Nominal Range	Nominal Variance	AR1 rho	Precision for IID.ID	Precision for CTRY.ID
**Africa quantiles**										
**Eastern sub-Saharan Africa quantiles**	0.025	-3.744	-0.305	1.139	-1.475	0.044	0.948	0.943	7.377	0.848
	0.5	-3.217	0.035	1.809	-0.844	0.054	1.247	0.957	8.076	3.399
	0.975	-2.690	0.374	2.480	-0.214	0.063	1.559	0.969	9.084	11.824
**Northern Africa quantiles**	0.025	-2.872	-0.383	0.991	-1.643	0.046	0.802	0.887	14.160	1.114
	0.5	-2.381	0.041	1.783	-0.824	0.053	0.982	0.912	15.643	3.450
	0.975	-1.891	0.465	2.575	-0.006	0.060	1.176	0.929	17.793	10.420
**Southern sub-Saharan Africa quantiles**	0.025	-3.685	-0.930	-2.080	1.112	0.107	1.947	0.939	26.783	0.408
	0.5	-2.368	0.052	-1.143	2.094	0.131	3.171	0.964	29.513	1.192
	0.975	-1.052	1.033	-0.204	3.065	0.159	4.716	0.975	31.770	2.826
**Western sub-Saharan quantiles**	0.025	-3.581	1.103	-2.727	-5.581	0.048	0.289	0.891	1931.458	0.234
	0.5	-2.679	2.313	0.778	-2.087	0.058	0.379	0.926	15391.062	0.721
	0.975	-1.780	3.520	4.340	1.340	0.072	0.517	0.951	73400.000	2.020
**Middle East quantiles**	0.025	-5.562	1.300	2.341	-5.648	0.181	12.462	0.994	6.195	4.288
	0.5	-2.380	2.225	3.436	-4.658	0.229	19.735	0.996	6.985	17.661
	0.975	0.800	3.148	4.533	-3.676	0.294	32.744	0.997	7.530	94.822
**Central Asia quantiles**	0.025	-3.984	0.373	-1.050	-2.044	0.088	1.383	0.859	2.295	0.268
	0.5	-3.010	1.609	0.131	-0.732	0.106	1.922	0.905	2.561	0.790
	0.975	-2.038	2.842	1.304	0.576	0.134	2.858	0.944	2.751	2.095
**South Asia quantiles**	0.025	-2.769	0.290	0.743	-0.933	0.103	3.440	0.967	14.197	1.165
	0.5	-1.854	0.502	1.104	-0.606	0.120	4.650	0.974	15.010	2.549
	0.975	-0.940	0.714	1.465	-0.280	0.141	6.599	0.981	16.137	4.959
**East Asia quantiles**	0.025	-2.809	1.648	1.210	-4.370	0.092	1.112	0.939	6.524	0.379
	0.5	-2.018	2.510	1.943	-3.453	0.127	1.947	0.969	7.223	0.930
	0.975	-1.228	3.372	2.676	-2.537	0.209	4.730	0.992	7.908	3.309
**Southeast Asia quantiles**	0.025	-1.819	1.898	0.655	-2.483	0.079	1.708	0.935	6.282	1.009
	0.5	-1.229	2.129	1.019	-2.147	0.090	2.206	0.953	6.611	1.962
	0.975	-0.639	2.359	1.382	-1.813	0.104	2.753	0.964	7.088	3.654
**Oceania quantiles**	0.025	-1.819	1.898	0.655	-2.483	0.079	1.708	0.935	6.282	1.009
	0.5	-1.229	2.129	1.019	-2.147	0.090	2.206	0.953	6.611	1.962
	0.975	-0.639	2.359	1.382	-1.813	0.104	2.753	0.964	7.088	3.654
**Andean South America quantiles**	0.025	-1.819	1.898	0.655	-2.483	0.079	1.708	0.935	6.282	1.009
	0.5	-1.229	2.129	1.019	-2.147	0.090	2.206	0.953	6.611	1.962
	0.975	-0.639	2.359	1.382	-1.813	0.104	2.753	0.964	7.088	3.654
**Tropical South America quantiles**	0.025	-4.183	0.273	-0.804	-0.385	0.052	0.505	0.879	4.509	0.120
	0.5	-2.936	0.783	-0.101	0.321	0.069	0.745	0.935	5.077	0.368
	0.975	-1.689	1.292	0.604	1.017	0.086	1.064	0.967	5.670	1.067
**Central America and**	0.025	-4.351	2.080	7.016	-14.610	0.073	0.793	0.862	13.833	0.696
	0.5	-3.825	3.051	9.856	-11.906	0.093	1.200	0.926	16.068	2.352
	0.975	-3.299	4.019	12.696	-9.206	0.121	1.890	0.963	20.264	6.495

		int	gam	gbm	lasso	Nominal Range	Nominal Variance	AR1 rho	Precision for IID.ID	Precision for CTRY.ID
**Caribbean quantiles**										

Supplementary Table 18. Proportion with 1–5 years, men ages 20–24 fitted parameters

Lower, median, and upper quantiles (0.025%, 0.50%, 0.975%) are displayed for the main parameters by region. The fixed effects covariates corresponding to the predicted ensemble rasters are shown in the first four columns, while fitted values for the spatiotemporal field hyperparameters and the precisions (inverse variance) for our random effects are shown in the last five columns.

		int	gam	gbm	lasso	Nominal Range	Nominal Variance	AR1 rho	Precision for IID.ID	Precision for CTRY.ID
**Central sub-Saharan Africa quantiles**	0.025	-1.772	1.761	0.402	-2.963	0.058	0.580	0.896	28.927	1.835
	0.5	-1.405	2.227	1.041	-2.269	0.072	0.820	0.929	37.571	4.495
	0.975	-1.039	2.694	1.683	-1.578	0.091	1.104	0.952	50.190	10.452
**Eastern sub-Saharan Africa quantiles**	0.025	-5.678	1.370	4.347	-7.015	0.309	16.102	0.995	11.428	1017.872
	0.5	-1.324	1.752	5.336	-6.088	0.419	29.546	0.997	12.291	4791.368
	0.975	3.027	2.133	6.324	-5.161	0.585	57.237	0.998	13.150	16698.411
**Northern Africa quantiles**	0.025	-2.736	-0.198	1.168	-1.667	0.075	0.915	0.875	29.948	0.613
	0.5	-2.104	0.176	1.811	-0.987	0.087	1.204	0.903	34.637	2.218
	0.975	-1.473	0.549	2.453	-0.307	0.100	1.508	0.920	39.051	6.688
**Southern sub-Saharan Africa quantiles**	0.025	-2.710	-0.299	-0.176	-1.048	0.077	0.807	0.978	27.941	0.616
	0.5	-1.958	0.544	0.607	-0.148	0.097	1.252	0.986	30.596	1.694
	0.975	-1.206	1.388	1.391	0.740	0.137	2.201	0.992	33.816	3.871
**Western sub-Saharan quantiles**	0.025	-2.872	-0.373	0.285	-2.225	0.082	0.749	0.855	1810.944	2255.081
	0.5	-2.370	0.361	1.570	-0.928	0.100	1.000	0.893	15300.000	17800.000
	0.975	-1.863	1.093	2.849	0.369	0.119	1.353	0.922	83981.503	93530.334
**Middle East quantiles**	0.025	-5.573	1.550	0.917	-2.968	0.282	30.095	0.998	7.657	1.862
	0.5	-0.519	2.038	1.441	-2.479	0.376	53.347	0.999	8.105	4.042
	0.975	4.532	2.526	1.965	-1.991	0.499	96.435	0.999	8.577	8.551
**Central Asia quantiles**	0.025	-2.734	1.299	-0.122	-1.432	0.042	0.565	0.911	9.343	0.244
	0.5	-1.883	1.718	0.298	-1.015	0.051	0.780	0.940	10.157	0.628
	0.975	-1.033	2.137	0.718	-0.600	0.066	1.107	0.961	11.201	1.500
**South Asia quantiles**	0.025	-2.734	1.299	-0.122	-1.432	0.042	0.565	0.911	9.343	0.244
	0.5	-1.883	1.718	0.298	-1.015	0.051	0.780	0.940	10.157	0.628
	0.975	-1.033	2.137	0.718	-0.600	0.066	1.107	0.961	11.201	1.500
**East Asia quantiles**	0.025	-2.019	0.311	-0.073	-1.207	0.073	0.231	0.777	11.400	0.328
	0.5	-1.549	0.925	0.635	-0.560	0.110	0.382	0.863	13.065	1.334
	0.975	-1.079	1.538	1.344	0.085	0.153	0.582	0.910	14.954	3.620

		int	gam	gbm	lasso	Nominal Range	Nominal Variance	AR1 rho	Precision for IID.ID	Precision for CTRY.ID
Southeast Asia quantiles	0.025	-2.112	1.646	1.001	-3.086	0.113	0.837	0.909	17.887	1.412
	0.5	-1.458	2.029	1.537	-2.566	0.155	1.475	0.944	20.534	2.761
	0.975	-0.805	2.411	2.080	-2.053	0.214	2.679	0.967	23.758	5.133
Oceania quantiles	0.025	-2.112	1.646	1.001	-3.086	0.113	0.837	0.909	17.887	1.412
	0.5	-1.458	2.029	1.537	-2.566	0.155	1.475	0.944	20.534	2.761
	0.975	-0.805	2.411	2.080	-2.053	0.214	2.679	0.967	23.758	5.133
Andean South America quantiles	0.025	-2.928	-0.706	-1.210	0.240	0.155	0.881	0.820	27541.651	0.301
	0.5	-2.089	0.136	-0.422	1.290	0.182	1.180	0.865	63216.340	0.820
	0.975	-1.250	0.975	0.357	2.340	0.216	1.590	0.901	143000.000	2.030
Tropical South America quantiles	0.025	-4.966	-0.703	-1.066	-0.100	0.207	1.458	0.940	9.848	0.340
	0.5	-2.409	0.166	-0.179	1.026	0.361	4.576	0.984	12.139	2.041
	0.975	0.146	1.033	0.660	2.172	0.807	22.704	0.998	15.008	10.842
Central America and Caribbean quantiles	0.025	-3.094	1.721	0.956	-2.876	0.091	1.304	0.941	14.380	0.745
	0.5	-2.479	1.956	1.470	-2.426	0.106	1.771	0.957	15.642	2.450
	0.975	-1.863	2.190	1.984	-1.976	0.130	2.547	0.971	16.712	6.743

Supplementary Table 19. Proportion with 6–11 years, men ages 20–24 fitted parameters

Lower, median, and upper quantiles (0.025%, 0.50%, 0.975%) are displayed for the main parameters by region. The fixed effects covariates corresponding to the predicted ensemble rasters are shown in the first four columns, while fitted values for the spatiotemporal field hyperparameters and the precisions (inverse variance) for our random effects are shown in the last five columns.

		int	gam	gbm	lasso	Nominal Range	Nominal Variance	AR1 rho	Precision for IID.ID	Precision for CTRY.ID
Central sub-Saharan Africa quantiles	0.025	0.718	1.118	2.371	-4.859	0.041	0.394	0.860	10.158	2.798
	0.5	0.968	1.826	3.157	-3.983	0.051	0.501	0.906	13.482	7.772
	0.975	1.218	2.532	3.943	-3.107	0.064	0.660	0.935	16.661	22.624
Eastern sub-Saharan Africa quantiles	0.025	-1.340	0.143	0.853	-0.868	0.142	2.302	0.984	12.835	0.129
	0.5	0.177	0.392	1.170	-0.562	0.177	3.496	0.989	13.451	0.437
	0.975	1.694	0.641	1.487	-0.256	0.231	5.835	0.993	14.320	1.315
Northern Africa quantiles	0.025	-0.206	0.917	2.998	-4.338	0.043	0.260	0.574	26.201	0.628
	0.5	0.283	1.273	3.498	-3.772	0.049	0.310	0.666	29.334	2.196
	0.975	0.772	1.630	3.999	-3.207	0.055	0.355	0.727	32.877	6.018
Southern sub-Saharan	0.025	0.100	-0.285	-0.258	0.658	0.062	0.334	0.948	39.634	0.957
	0.5	0.596	0.003	0.015	0.981	0.076	0.479	0.962	42.919	2.601
	0.975	1.092	0.292	0.288	1.305	0.094	0.717	0.973	46.447	6.147

		int	gam	gbm	lasso	Nominal Range	Nominal Variance	AR1 rho	Precision for IID.ID	Precision for CTRY.ID
**Africa quantiles**										
**Western sub-Saharan quantiles**	0.025	-0.604	1.041	0.061	-1.531	0.048	0.252	0.898	105.570	12.885
	0.5	-0.359	1.398	0.580	-0.978	0.057	0.332	0.923	149.667	123.037
	0.975	-0.114	1.754	1.099	-0.425	0.068	0.434	0.943	222.815	2192.364
**Middle East quantiles**	0.025	-0.521	1.214	0.514	-1.957	0.096	3.592	0.994	9.184	1.087
	0.5	0.624	1.586	0.965	-1.550	0.116	5.136	0.995	9.638	3.132
	0.975	1.769	1.958	1.415	-1.144	0.150	8.305	0.997	9.995	7.344
**Central Asia quantiles**	0.025	0.124	1.338	0.938	-2.503	0.040	0.275	0.463	12.114	1.370
	0.5	0.469	1.787	1.384	-2.171	0.048	0.343	0.638	13.233	3.823
	0.975	0.813	2.235	1.830	-1.840	0.060	0.437	0.755	14.274	8.920
**South Asia quantiles**	0.025	0.124	1.338	0.938	-2.503	0.040	0.275	0.463	12.114	1.370
	0.5	0.469	1.787	1.384	-2.171	0.048	0.343	0.638	13.233	3.823
	0.975	0.813	2.235	1.830	-1.840	0.060	0.437	0.755	14.274	8.920
**East Asia quantiles**	0.025	-0.896	0.792	0.925	-2.679	0.192	0.463	0.873	38.058	1.636
	0.5	0.074	1.482	1.542	-2.024	0.317	1.105	0.943	45.137	7.166
	0.975	1.043	2.172	2.159	-1.371	0.523	2.579	0.974	52.868	38.834
**Southeast Asia quantiles**	0.025	-0.140	1.377	2.107	-3.732	0.120	0.642	0.874	16.595	1.505
	0.5	0.423	1.831	2.505	-3.336	0.166	1.135	0.920	18.458	3.140
	0.975	0.985	2.285	2.903	-2.940	0.242	2.190	0.954	20.592	6.220
**Oceania quantiles**	0.025	-0.140	1.377	2.107	-3.732	0.120	0.642	0.874	16.595	1.505
	0.5	0.423	1.831	2.505	-3.336	0.166	1.135	0.920	18.458	3.140
	0.975	0.985	2.285	2.903	-2.940	0.242	2.190	0.954	20.592	6.220
**Andean South America quantiles**	0.025	-0.570	0.509	-0.575	-0.440	0.054	0.342	-0.506	122.050	0.752
	0.5	0.000	0.954	-0.196	0.242	0.091	0.820	0.511	130.606	1.963
	0.975	0.569	1.399	0.182	0.924	0.118	1.276	0.798	138.658	6.597
**Tropical South America quantiles**	0.025	-0.287	-0.176	0.884	-1.684	0.057	0.298	0.852	7.893	3.558
	0.5	0.062	0.391	1.531	-0.922	0.077	0.475	0.916	9.195	19.078
	0.975	0.410	0.958	2.177	-0.160	0.109	0.785	0.955	10.467	81.096
**Central America and Caribbean quantiles**	0.025	-0.431	1.753	0.685	-2.575	0.110	1.297	0.943	11.487	0.553
	0.5	0.336	2.028	1.129	-2.157	0.130	1.786	0.958	12.382	1.395
	0.975	1.102	2.303	1.572	-1.740	0.159	2.596	0.971	13.827	3.544

Supplementary Table 20. Mean years, women ages 15–49 fitted parameters

Lower, median, and upper quantiles (0.025%, 0.50%, 0.975%) are displayed for the main parameters by region. The fixed effects covariates corresponding to the predicted ensemble rasters are shown in the first four columns, while fitted values for the spatiotemporal field hyperparameters and the precisions (inverse variance) for our random effects are shown in the last five columns.

		int	gam	gbm	lasso	Nominal Range	Nominal Variance	AR1 rho	Precision for IID.ID	Precision for CTRY.ID
Central sub-Saharan Africa quantiles	0.025	-0.752	0.348	0.163	0.304	0.067	1.426	0.961	4.722	0.930
	0.5	-0.186	0.395	0.238	0.367	0.083	2.007	0.974	5.516	2.360
	0.975	0.380	0.443	0.313	0.430	0.106	3.070	0.984	7.397	5.520
Eastern sub-Saharan Africa quantiles	0.025	-0.753	0.526	0.180	0.154	0.017	1.323	0.985	4.078	0.616
	0.5	-0.188	0.557	0.237	0.206	0.031	2.038	0.992	4.375	2.341
	0.975	0.376	0.588	0.294	0.257	0.041	2.647	0.995	4.685	7.749
Northern Africa quantiles	0.025	-0.666	0.393	-0.004	0.508	0.042	1.774	0.917	6.480	0.913
	0.5	-0.135	0.418	0.037	0.546	0.049	2.169	0.932	6.997	3.463
	0.975	0.394	0.442	0.078	0.583	0.055	2.619	0.944	7.656	12.674
Southern sub-Saharan Africa quantiles	0.025	-1.696	0.929	-0.494	0.419	0.104	2.910	0.971	6.255	0.140
	0.5	-0.597	0.972	-0.449	0.477	0.129	4.329	0.982	6.601	0.392
	0.975	0.502	1.015	-0.403	0.535	0.152	6.071	0.987	6.988	0.895
Western sub-Saharan quantiles	0.025	-1.416	0.644	-0.051	0.269	0.031	1.705	0.937	9.517	0.166
	0.5	-0.523	0.683	-0.003	0.320	0.035	1.993	0.952	13.151	0.613
	0.975	0.369	0.722	0.044	0.372	0.040	2.341	0.966	16.962	2.607
Middle East quantiles	0.025	-1.381	0.495	0.104	0.260	0.152	30.654	0.999	2.948	0.325
	0.5	-0.013	0.529	0.160	0.311	0.236	73.899	0.999	3.081	0.915
	0.975	1.355	0.563	0.216	0.362	0.349	161.160	1.000	3.232	2.523
Central Asia quantiles	0.025	-0.602	0.613	0.037	0.163	0.034	1.211	0.949	2.448	0.176
	0.5	0.220	0.656	0.108	0.236	0.041	1.623	0.966	2.621	0.460
	0.975	1.042	0.699	0.178	0.309	0.052	2.199	0.979	2.797	1.099
South Asia quantiles	0.025	-1.180	0.214	0.208	0.496	0.147	15.591	0.991	4.564	0.296
	0.5	0.059	0.234	0.242	0.525	0.175	22.281	0.993	4.848	0.596
	0.975	1.297	0.253	0.275	0.553	0.219	34.530	0.995	5.419	1.076
East Asia quantiles	0.025	-0.936	0.345	-0.003	0.503	0.070	2.941	0.976	1.279	0.134
	0.5	-0.053	0.391	0.050	0.559	0.089	4.125	0.985	1.371	0.365
	0.975	0.830	0.437	0.102	0.616	0.110	5.900	0.991	1.452	0.906
Southeast Asia quantiles	0.025	-0.162	0.287	0.033	0.571	0.086	2.596	0.962	4.171	0.646
	0.5	0.573	0.311	0.078	0.612	0.104	3.764	0.972	4.523	1.306
	0.975	1.308	0.334	0.123	0.652	0.128	5.345	0.979	4.874	2.463
Oceania quantiles	0.025	-0.162	0.287	0.033	0.571	0.086	2.596	0.962	4.171	0.646
	0.5	0.573	0.311	0.078	0.612	0.104	3.764	0.972	4.523	1.306
	0.975	1.308	0.334	0.123	0.652	0.128	5.345	0.979	4.874	2.463
Andean South America quantiles	0.025	-0.563	0.978	-0.267	0.221	0.206	2.089	0.014	84.569	1099.152
	0.5	-0.048	0.996	-0.244	0.248	0.239	2.695	0.481	98.598	11896.816
	0.975	0.467	1.014	-0.221	0.276	0.287	3.546	0.844	115.700	60201.968
Tropical South America quantiles	0.025	-0.896	0.176	-0.035	0.649	0.045	1.456	0.884	1218.805	0.052
	0.5	0.151	0.222	0.044	0.734	0.067	1.980	0.939	13600.000	0.207
	0.975	1.200	0.268	0.123	0.819	0.088	2.780	0.965	68500.000	0.536
	0.025	-0.519	0.741	-0.012	0.125	0.064	2.667	0.938	3.923	0.690

		int	gam	gbm	lasso	Nominal Range	Nominal Variance	AR1 rho	Precision for IID.ID	Precision for CTRY.ID
Central America and Caribbean quantiles	0.5	0.068	0.778	0.039	0.183	0.071	3.225	0.949	4.209	2.494
	0.975	0.654	0.816	0.089	0.241	0.080	4.038	0.960	4.489	8.752

Supplementary Table 21. Mean years, men ages 15–49 fitted parameters

Lower, median, and upper quantiles (0.025%, 0.50%, 0.975%) are displayed for the main parameters by region. The fixed effects covariates corresponding to the predicted ensemble rasters are shown in the first four columns, while fitted values for the spatiotemporal field hyperparameters and the precisions (inverse variance) for our random effects are shown in the last five columns.

		int	gam	gbm	lasso	Nominal Range	Nominal Variance	AR1 rho	Precision for IID.ID	Precision for CTRY.ID
Central sub-Saharan Africa quantiles	0.025	-0.838	0.421	0.069	0.308	0.060	1.121	0.962	5.386	0.577
	0.5	-0.265	0.476	0.149	0.375	0.077	1.627	0.976	6.196	1.369
	0.975	0.307	0.530	0.229	0.442	0.098	2.355	0.985	7.058	3.117
Eastern sub-Saharan Africa quantiles	0.025	-0.794	0.728	0.011	0.119	0.032	1.110	0.988	4.382	0.676
	0.5	-0.232	0.765	0.066	0.169	0.038	1.369	0.992	5.214	2.183
	0.975	0.330	0.803	0.121	0.219	0.049	1.755	0.994	5.714	7.074
Northern Africa quantiles	0.025	-0.995	0.348	-0.010	0.530	0.052	1.578	0.924	6.712	0.473
	0.5	-0.375	0.376	0.044	0.580	0.058	1.871	0.935	8.027	1.631
	0.975	0.245	0.403	0.097	0.631	0.067	2.297	0.947	8.852	4.694
Southern sub-Saharan Africa quantiles	0.025	-1.636	0.986	-0.470	0.360	0.098	3.060	0.980	6.504	0.231
	0.5	-0.578	1.026	-0.431	0.405	0.121	4.616	0.987	6.957	0.605
	0.975	0.480	1.065	-0.391	0.451	0.151	7.422	0.992	7.469	1.452
Western sub-Saharan quantiles	0.025	-1.253	0.624	-0.055	0.272	0.027	0.665	0.923	15.851	0.180
	0.5	-0.482	0.671	-0.001	0.329	0.034	0.885	0.943	21.750	0.751
	0.975	0.289	0.718	0.054	0.386	0.040	1.105	0.959	30.816	2.595
Middle East quantiles	0.025	-1.375	0.535	0.018	0.311	0.208	44.042	0.999	3.226	0.501
	0.5	-0.005	0.571	0.072	0.357	0.273	75.594	1.000	3.377	1.597
	0.975	1.364	0.606	0.126	0.404	0.373	143.783	1.000	3.541	4.663
Central Asia quantiles	0.025	-0.559	0.536	0.067	0.225	0.040	1.072	0.948	3.149	0.222
	0.5	0.205	0.582	0.131	0.287	0.049	1.447	0.965	3.399	0.607
	0.975	0.968	0.628	0.195	0.349	0.063	2.087	0.980	3.633	1.511
South Asia quantiles	0.025	-1.255	0.235	0.202	0.471	0.168	19.088	0.994	4.559	0.321
	0.5	0.056	0.257	0.240	0.503	0.211	30.257	0.996	4.806	0.628
	0.975	1.367	0.280	0.278	0.534	0.258	45.091	0.997	5.198	1.165
	0.025	-1.135	0.227	0.012	0.575	0.080	2.959	0.979	1.851	0.217
	0.5	-0.243	0.283	0.074	0.643	0.111	5.022	0.988	2.015	0.689

		int	gam	gbm	lasso	Nominal Range	Nominal Variance	AR1 rho	Precision for IID.ID	Precision for CTRY.ID
East Asia quantiles	0.975	0.648	0.339	0.136	0.711	0.149	8.369	0.993	2.152	1.827
Southeast Asia quantiles	0.025	-0.188	0.281	0.056	0.538	0.077	2.152	0.963	3.458	0.414
	0.5	0.584	0.304	0.109	0.587	0.099	3.641	0.979	3.678	0.799
	0.975	1.356	0.328	0.162	0.635	0.124	5.399	0.985	3.965	1.491
Oceania quantiles	0.025	-0.188	0.281	0.056	0.538	0.077	2.152	0.963	3.458	0.414
	0.5	0.584	0.304	0.109	0.587	0.099	3.641	0.979	3.678	0.799
	0.975	1.356	0.328	0.162	0.635	0.124	5.399	0.985	3.965	1.491
Andean South America quantiles	0.025	-0.090	0.417	-0.654	1.105	0.115	0.705	0.631	11.315	1356.584
	0.5	0.281	0.444	-0.603	1.159	0.143	1.006	0.824	12.116	12965.278
	0.975	0.652	0.470	-0.552	1.213	0.181	1.473	0.915	13.150	69925.585
Tropical South America quantiles	0.025	-1.144	0.167	-0.045	0.643	0.052	0.873	0.835	6.924	0.083
	0.5	-0.098	0.220	0.045	0.734	0.070	1.385	0.914	13.960	0.237
	0.975	0.948	0.274	0.135	0.826	0.109	2.117	0.963	41.711	0.645
Central America and Caribbean quantiles	0.025	-0.562	0.637	-0.187	0.380	0.068	2.031	0.936	3.896	0.382
	0.5	0.052	0.676	-0.125	0.448	0.076	2.438	0.947	4.142	1.240
	0.975	0.665	0.716	-0.062	0.516	0.090	3.103	0.960	4.471	3.019

Supplementary Table 22. Mean years, women ages 20–24 fitted parameters

Lower, median, and upper quantiles (0.025%, 0.50%, 0.975%) are displayed for the main parameters by region. The fixed effects covariates corresponding to the predicted ensemble rasters are shown in the first four columns, while fitted values for the spatiotemporal field hyperparameters and the precisions (inverse variance) for our random effects are shown in the last five columns.

		int	gam	gbm	lasso	Nominal Range	Nominal Variance	AR1 rho	Precision for IID.ID	Precision for CTRY.ID
Central sub-Saharan Africa quantiles	0.025	-0.769	0.303	0.121	0.360	0.060	1.680	0.954	3.834	0.533
	0.5	-0.155	0.355	0.210	0.435	0.076	2.377	0.970	4.465	1.412
	0.975	0.459	0.407	0.298	0.510	0.097	3.411	0.981	5.154	3.361
Eastern sub-Saharan Africa quantiles	0.025	-0.672	0.593	-0.058	0.316	0.032	1.574	0.979	5.123	0.439
	0.5	-0.045	0.628	-0.001	0.373	0.040	2.209	0.986	5.623	1.745
	0.975	0.582	0.664	0.056	0.429	0.047	2.830	0.990	6.117	6.040
Northern Africa quantiles	0.025	-0.640	0.305	0.004	0.550	0.044	2.631	0.894	4.383	0.487
	0.5	-0.063	0.335	0.061	0.604	0.049	3.092	0.911	4.772	2.659
	0.975	0.514	0.366	0.118	0.657	0.056	3.740	0.928	5.274	10.781
Southern sub-Saharan	0.025	-1.776	0.922	-0.355	0.280	0.077	1.704	0.975	10.038	0.093
	0.5	-0.775	0.966	-0.307	0.341	0.092	2.391	0.982	10.690	0.271
	0.975	0.226	1.010	-0.260	0.402	0.108	3.415	0.988	11.528	0.615

		int	gam	gbm	lasso	Nominal Range	Nominal Variance	AR1 rho	Precision for IID.ID	Precision for CTRY.ID
**Africa quantiles**										
**Western sub-Saharan quantiles**	0.025	-1.889	0.400	-0.082	0.517	0.031	1.444	0.950	20.779	0.057
	0.5	-0.794	0.445	-0.026	0.581	0.036	1.764	0.967	38.708	0.188
	0.975	0.299	0.490	0.030	0.645	0.042	2.263	0.977	110.729	0.547
**Middle East quantiles**	0.025	-1.228	0.405	0.089	0.355	0.081	8.324	0.993	2.941	0.279
	0.5	-0.159	0.445	0.148	0.407	0.098	12.102	0.995	3.093	0.916
	0.975	0.909	0.484	0.207	0.460	0.126	19.678	0.997	3.219	2.319
**Central Asia quantiles**	0.025	-0.663	0.619	0.102	0.065	0.034	1.450	0.898	1.891	0.129
	0.5	0.237	0.665	0.184	0.151	0.044	2.028	0.936	2.034	0.342
	0.975	1.137	0.711	0.266	0.237	0.054	2.708	0.956	2.184	0.809
**South Asia quantiles**	0.025	-0.663	0.619	0.102	0.065	0.034	1.450	0.898	1.891	0.129
	0.5	0.237	0.665	0.184	0.151	0.044	2.028	0.936	2.034	0.342
	0.975	1.137	0.711	0.266	0.237	0.054	2.708	0.956	2.184	0.809
**East Asia quantiles**	0.025	-1.136	0.223	-0.017	0.619	0.052	3.138	0.981	1.282	0.066
	0.5	-0.109	0.276	0.039	0.685	0.073	4.479	0.988	1.390	0.159
	0.975	0.918	0.329	0.095	0.750	0.091	6.114	0.992	1.505	0.408
**Southeast Asia quantiles**	0.025	-0.267	0.230	0.105	0.547	0.096	4.406	0.965	2.877	0.414
	0.5	0.654	0.254	0.154	0.592	0.118	6.574	0.975	3.152	0.829
	0.975	1.574	0.277	0.204	0.637	0.149	9.795	0.982	3.412	1.610
**Oceania quantiles**	0.025	-0.267	0.230	0.105	0.547	0.096	4.406	0.965	2.877	0.414
	0.5	0.654	0.254	0.154	0.592	0.118	6.574	0.975	3.152	0.829
	0.975	1.574	0.277	0.204	0.637	0.149	9.795	0.982	3.412	1.610
**Andean South America quantiles**	0.025	-0.962	0.957	-0.293	0.248	0.170	2.945	0.047	46.963	1209.715
	0.5	-0.405	0.982	-0.265	0.283	0.199	3.761	0.383	54.302	14318.265
	0.975	0.152	1.007	-0.237	0.318	0.229	4.886	0.663	62.993	72811.134
**Tropical South America quantiles**	0.025	-0.952	0.214	-0.045	0.553	0.052	1.477	0.896	1103.715	0.070
	0.5	0.102	0.267	0.067	0.666	0.065	1.940	0.939	11000.000	0.224
	0.975	1.160	0.319	0.179	0.780	0.081	2.710	0.970	63600.000	0.595
**Central America and Caribbean quantiles**	0.025	-0.673	0.727	0.011	0.079	0.053	2.520	0.926	4.390	0.807
	0.5	-0.214	0.778	0.068	0.154	0.058	2.964	0.939	4.745	4.908
	0.975	0.245	0.829	0.126	0.229	0.065	3.601	0.952	5.099	21.510

Supplementary Table 23. Mean years, men ages 20–24 fitted parameters

Lower, median, and upper quantiles (0.025%, 0.50%, 0.975%) are displayed for the main parameters by region. The fixed effects covariates corresponding to the predicted ensemble rasters are shown in the first four columns, while fitted values for the spatiotemporal field hyperparameters and the precisions (inverse variance) for our random effects are shown in the last five columns.

		int	gam	gbm	lasso	Nominal Range	Nominal Variance	AR1 rho	Precision for IID.ID	Precision for CTRY.ID
**Central sub-Saharan Africa quantiles**	0.025	-0.822	0.358	0.009	0.381	0.065	1.121	0.943	5.300	0.560
	0.5	-0.261	0.420	0.110	0.469	0.081	1.533	0.965	6.259	1.367
	0.975	0.300	0.482	0.212	0.557	0.111	2.202	0.983	7.961	3.531
**Eastern sub-Saharan Africa quantiles**	0.025	-0.808	0.726	-0.096	0.211	0.041	1.252	0.982	5.750	0.368
	0.5	-0.094	0.769	-0.038	0.269	0.052	1.828	0.987	6.234	1.220
	0.975	0.620	0.812	0.019	0.328	0.064	2.473	0.990	6.806	4.022
**Northern Africa quantiles**	0.025	-0.885	0.207	0.019	0.568	0.057	2.112	0.902	6.086	0.262
	0.5	-0.153	0.244	0.105	0.651	0.064	2.522	0.917	6.667	0.957
	0.975	0.579	0.282	0.191	0.733	0.075	3.169	0.935	7.549	2.614
**Southern sub-Saharan Africa quantiles**	0.025	-1.567	0.941	-0.357	0.291	0.068	1.471	0.977	10.107	0.187
	0.5	-0.642	0.983	-0.319	0.337	0.091	2.482	0.986	11.023	0.519
	0.975	0.283	1.024	-0.282	0.382	0.117	4.005	0.992	12.331	1.188
**Western sub-Saharan quantiles**	0.025	-1.758	0.340	-0.092	0.562	0.025	0.701	0.887	801.415	0.130
	0.5	-0.867	0.391	-0.027	0.636	0.044	0.990	0.934	10100.000	0.499
	0.975	0.023	0.443	0.038	0.710	0.058	1.270	0.955	59800.000	1.740
**Middle East quantiles**	0.025	-1.324	0.412	0.061	0.376	0.095	10.104	0.995	3.367	0.731
	0.5	-0.160	0.453	0.119	0.428	0.118	15.763	0.997	3.651	2.351
	0.975	1.003	0.495	0.177	0.479	0.151	25.554	0.998	3.859	6.889
**Central Asia quantiles**	0.025	-0.832	0.561	0.087	0.124	0.034	1.092	0.878	2.690	0.196
	0.5	0.022	0.614	0.170	0.216	0.056	1.874	0.937	2.895	0.510
	0.975	0.875	0.667	0.254	0.307	0.074	2.786	0.960	3.215	1.344
**South Asia quantiles**	0.025	-0.384	0.186	0.157	0.542	0.066	3.514	0.975	4.721	0.302
	0.5	0.343	0.214	0.205	0.582	0.079	4.798	0.982	5.073	0.578
	0.975	1.070	0.241	0.252	0.621	0.102	7.163	0.988	5.433	1.080
**East Asia quantiles**	0.025	-1.279	0.113	-0.021	0.714	0.065	2.321	0.981	2.255	0.165
	0.5	-0.461	0.169	0.046	0.785	0.078	3.194	0.989	2.448	0.437
	0.975	0.358	0.224	0.113	0.856	0.099	4.501	0.994	2.614	1.152
**Southeast Asia quantiles**	0.025	-0.187	0.255	0.082	0.526	0.071	2.178	0.935	2.585	0.309
	0.5	0.618	0.280	0.140	0.580	0.095	4.204	0.968	2.959	0.636
	0.975	1.423	0.305	0.198	0.634	0.120	6.511	0.979	3.241	1.178
**Oceania quantiles**	0.025	-0.187	0.255	0.082	0.526	0.071	2.178	0.935	2.585	0.309
	0.5	0.618	0.280	0.140	0.580	0.095	4.204	0.968	2.959	0.636
	0.975	1.423	0.305	0.198	0.634	0.120	6.511	0.979	3.241	1.178
**Andean South America quantiles**	0.025	-0.628	0.272	-0.849	1.440	0.094	0.926	0.330	11.577	1597.043
	0.5	-0.270	0.304	-0.794	1.489	0.119	1.325	0.688	12.476	15224.582
	0.975	0.089	0.337	-0.738	1.539	0.145	1.830	0.861	13.445	74726.256
**Tropical South America quantiles**	0.025	-1.130	0.147	-0.005	0.542	0.056	0.938	0.495	1130.000	0.087
	0.5	-0.110	0.209	0.121	0.670	0.077	1.710	0.893	11800.000	0.270
	0.975	0.906	0.271	0.246	0.798	0.119	2.690	0.959	64600.000	0.676

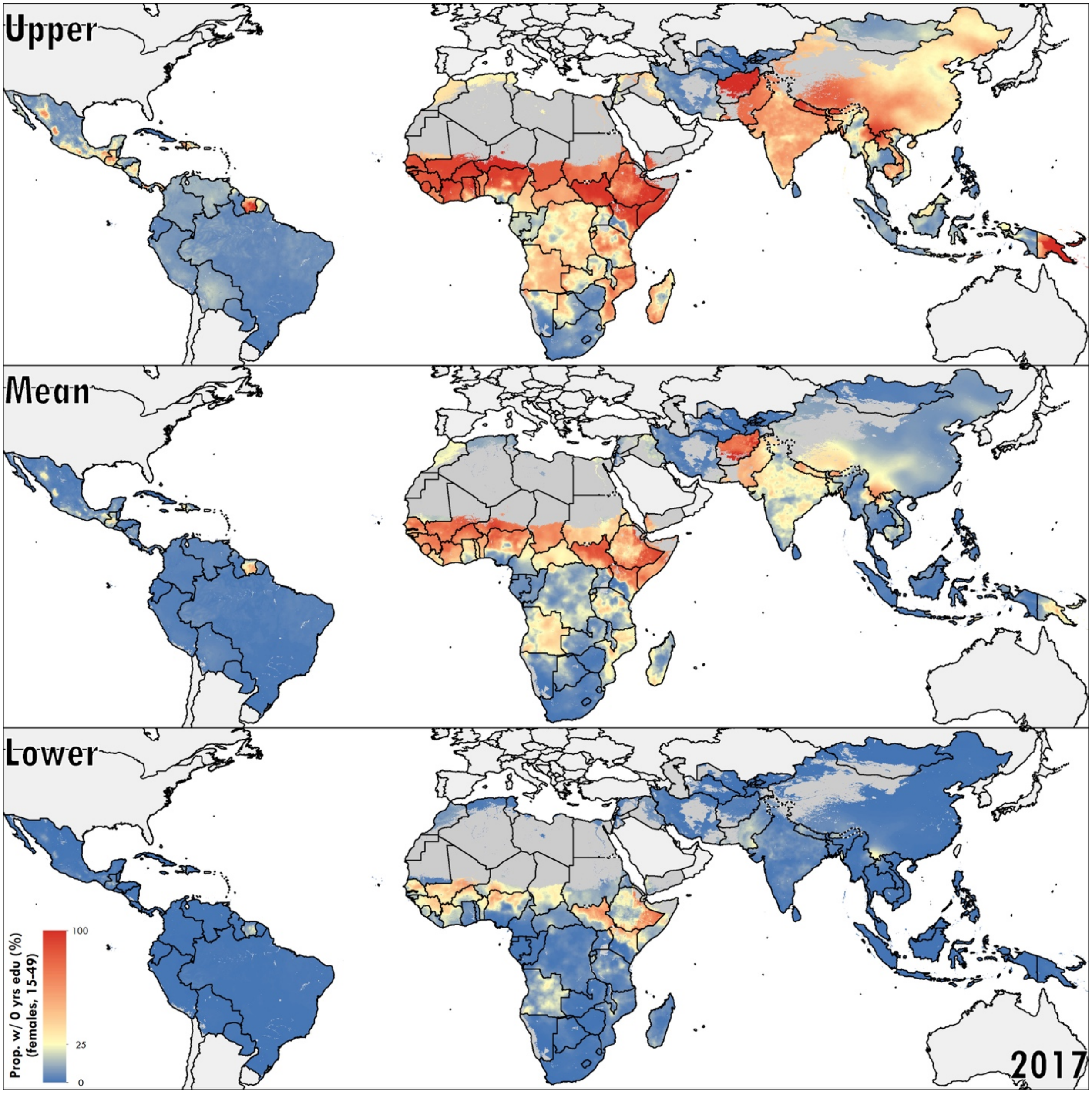

Supplementary Figure 8. Zero years, women ages 15–49 posterior means and 95% uncertainty interval, 2017

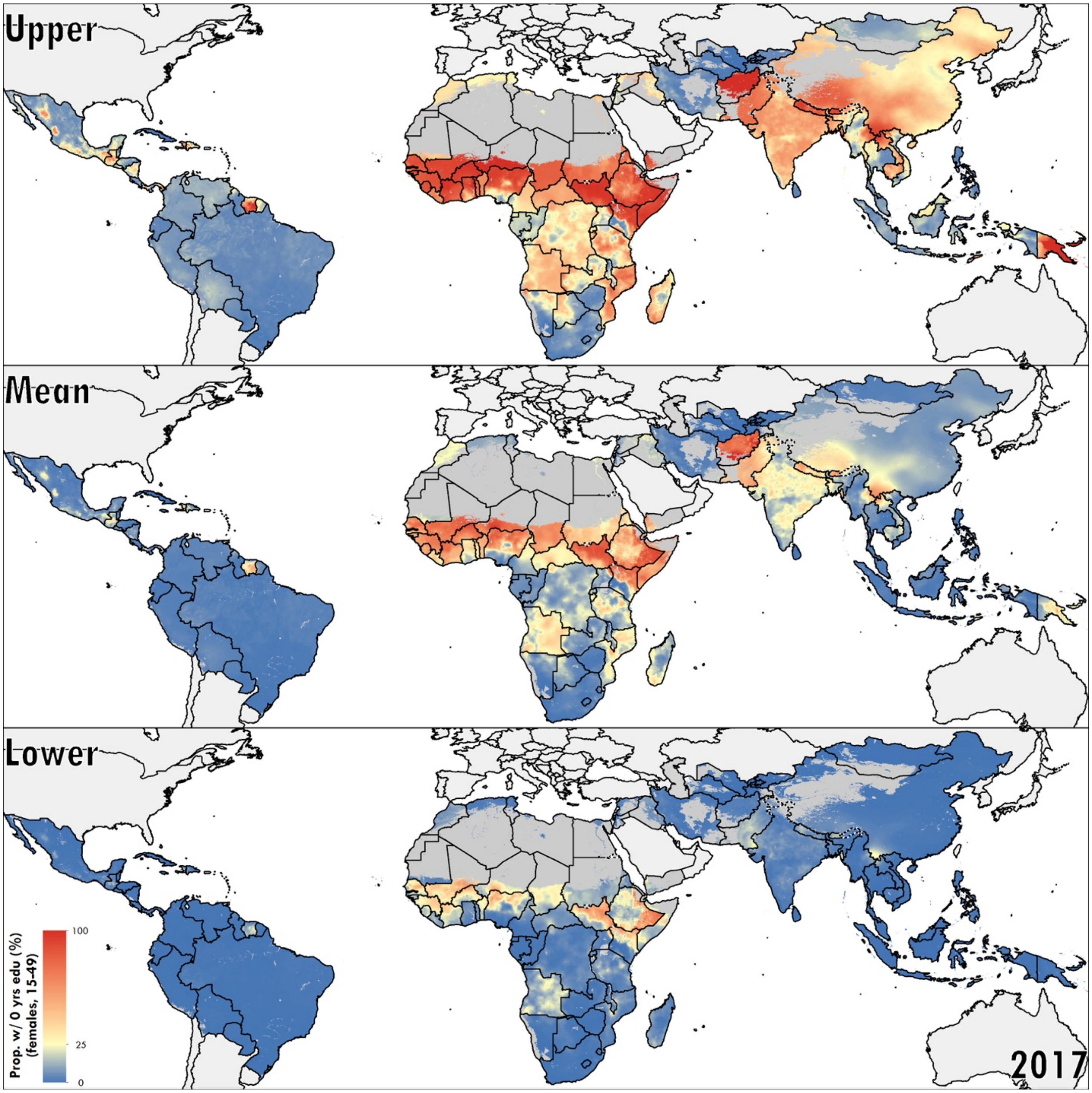

Supplementary Figure 9. Zero years, men ages 15–49 posterior means and 95% uncertainty interval, 2017

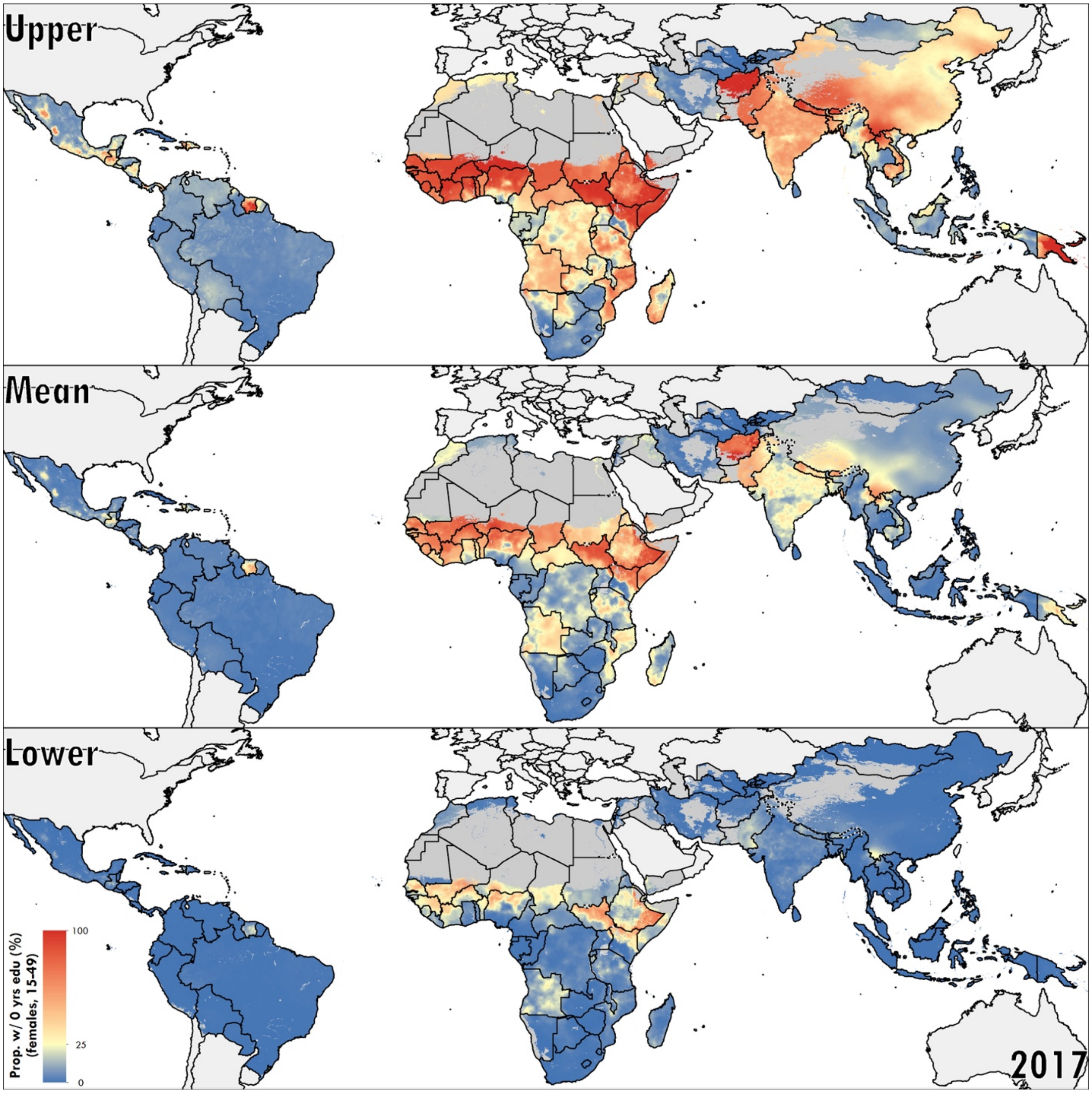

Supplementary Figure 10. Zero years, women ages 20–24 posterior means and 95% uncertainty interval, 2017

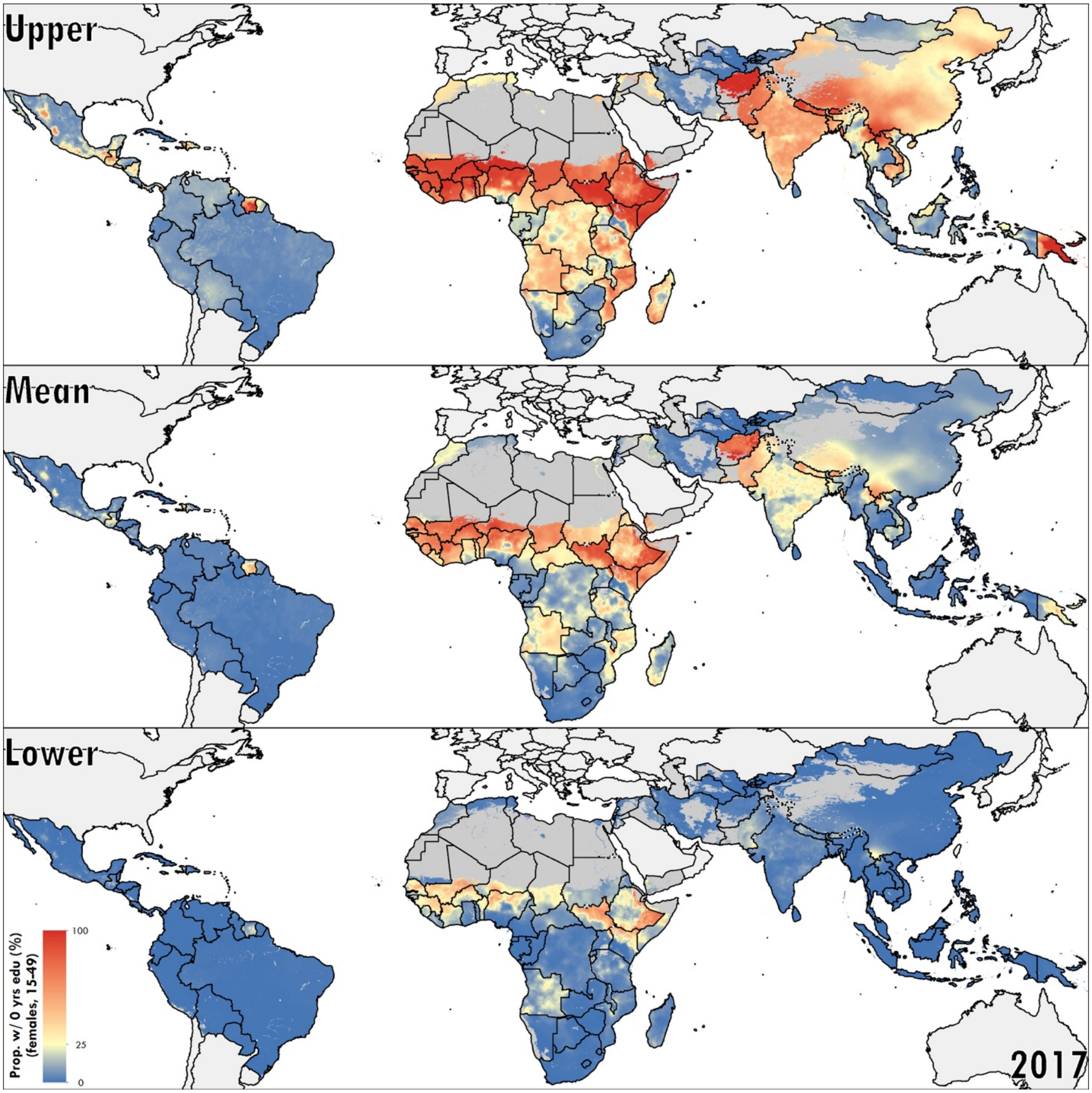

Supplementary Figure 11. Zero years, men ages 20–24 posterior means and 95% uncertainty interval, 2017

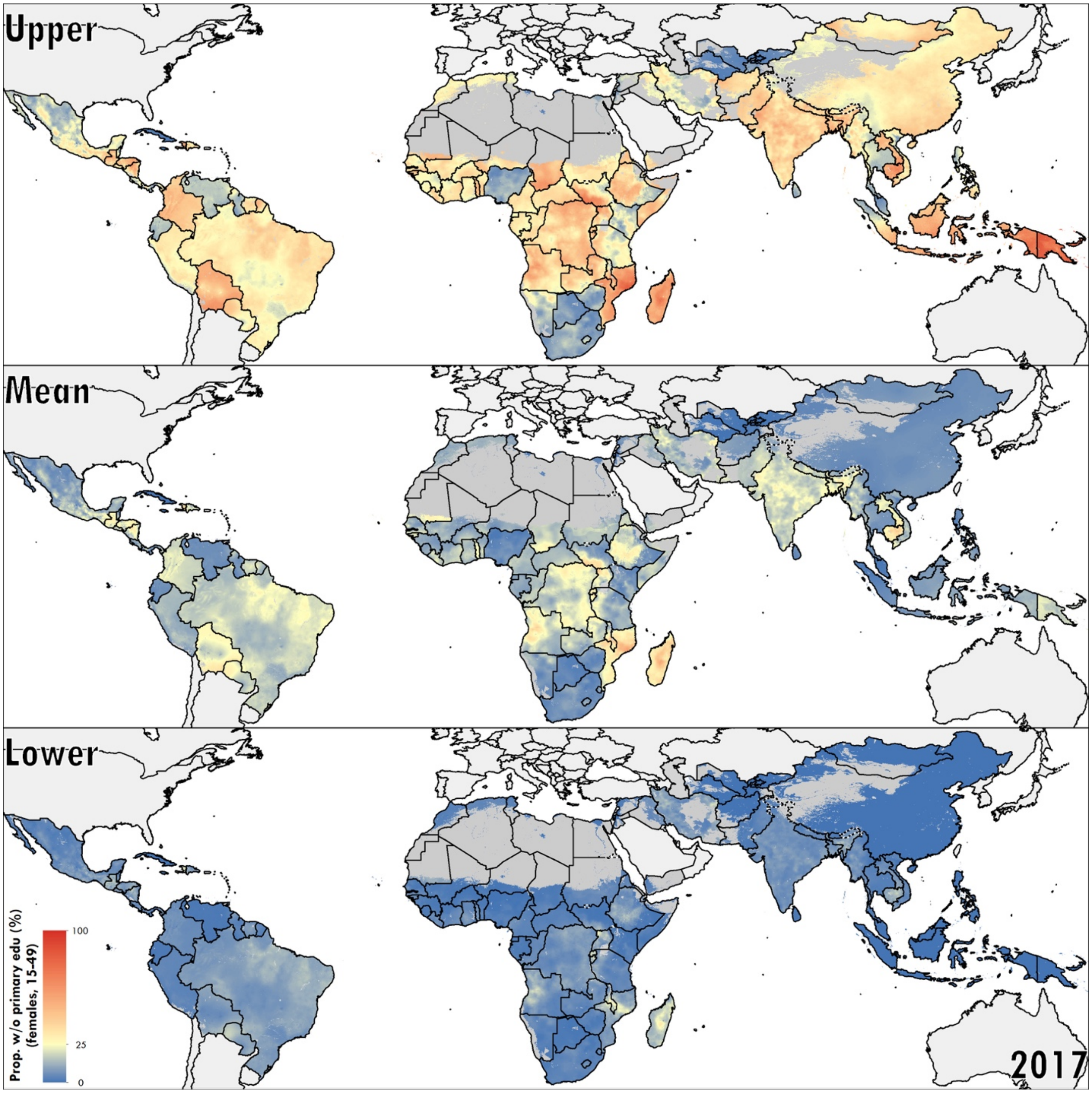

Supplementary Figure 12. 1–5 years, women ages 15–49 posterior means and 95% uncertainty interval, 2017

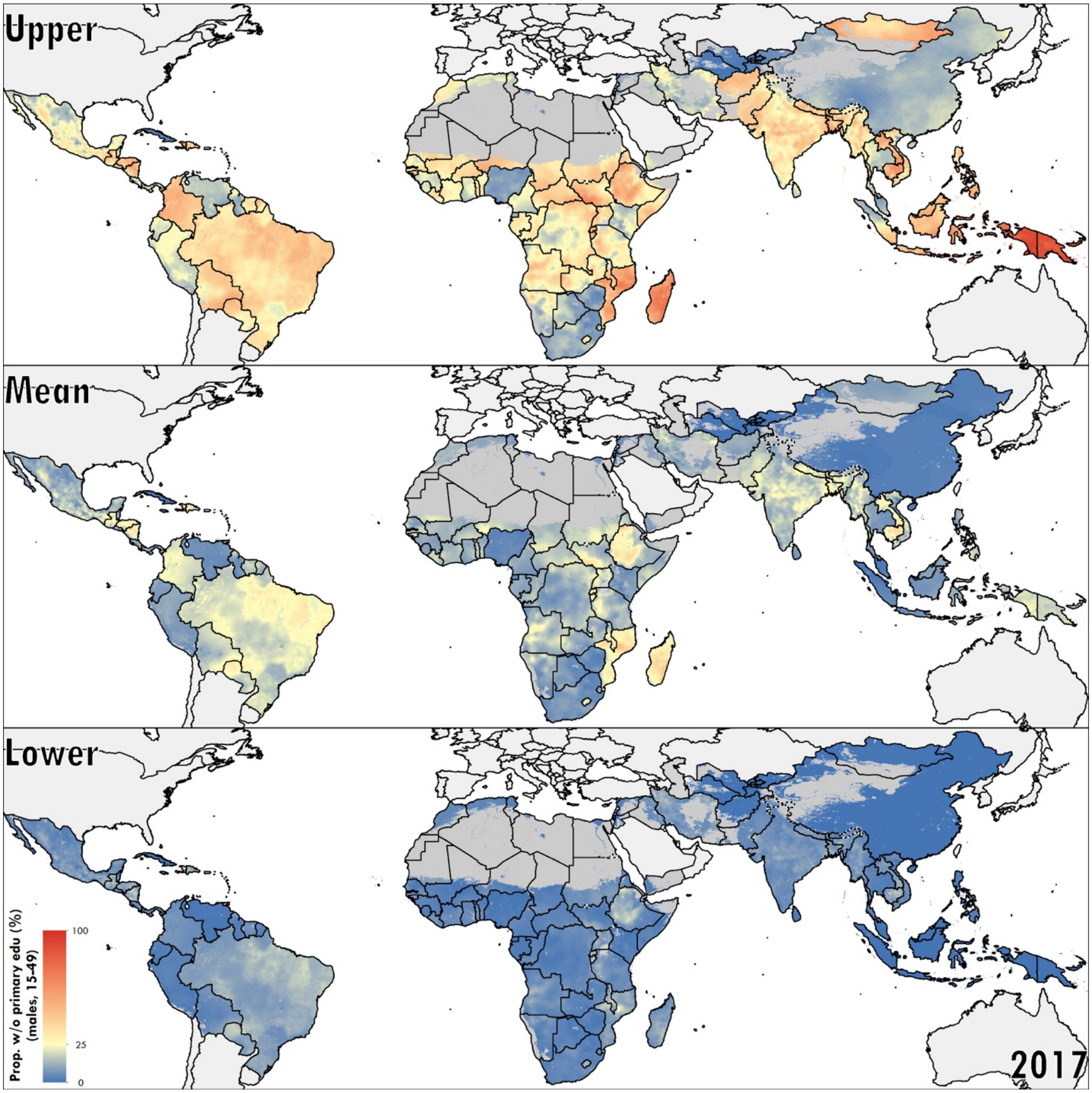

Supplementary Figure 13. 1–5 years, men ages 15–49 posterior means and 95% uncertainty interval, 2017

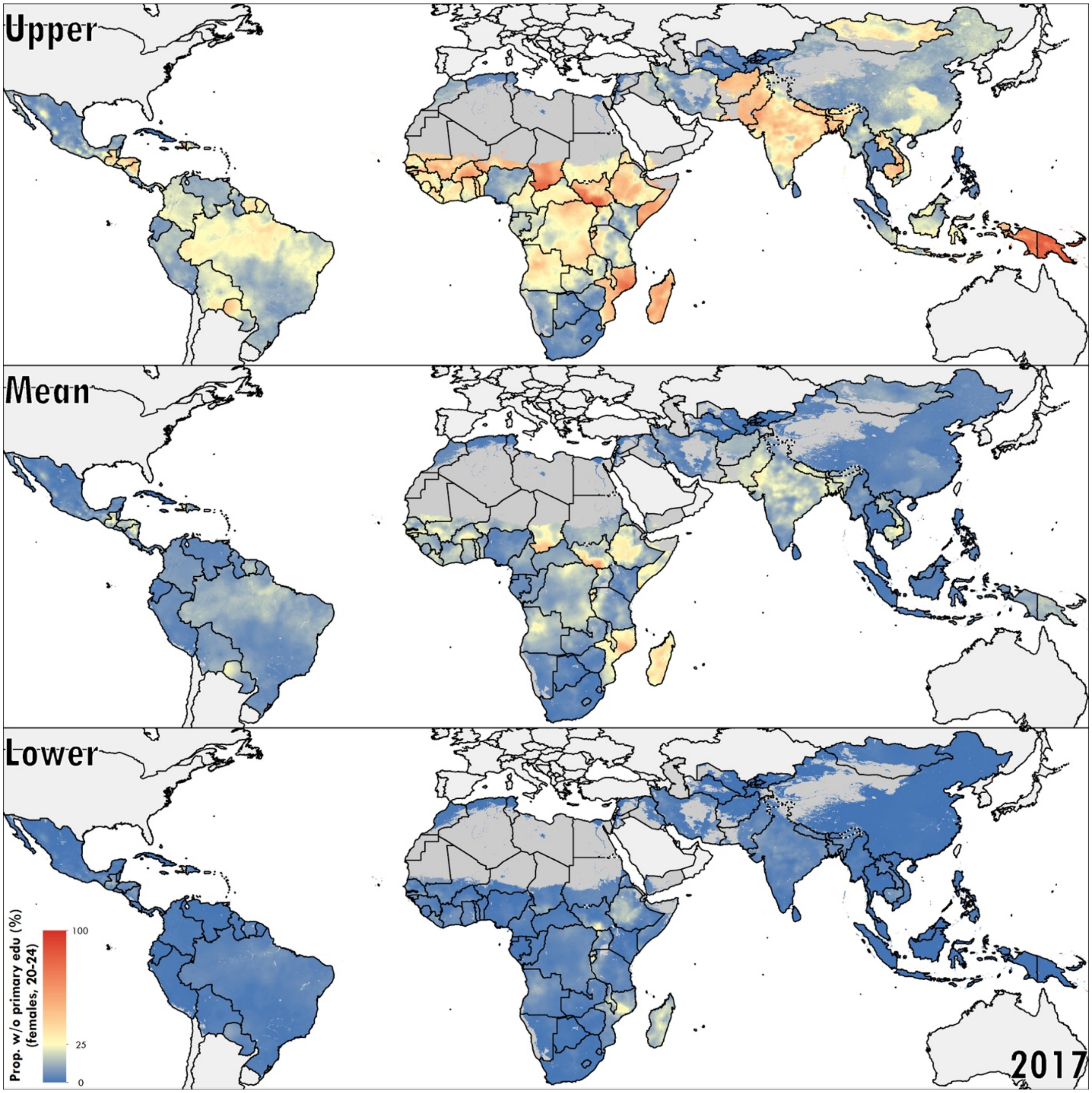

Supplementary Figure 14. 1–5 years, women ages 20–24 posterior means and 95% uncertainty interval, 2017

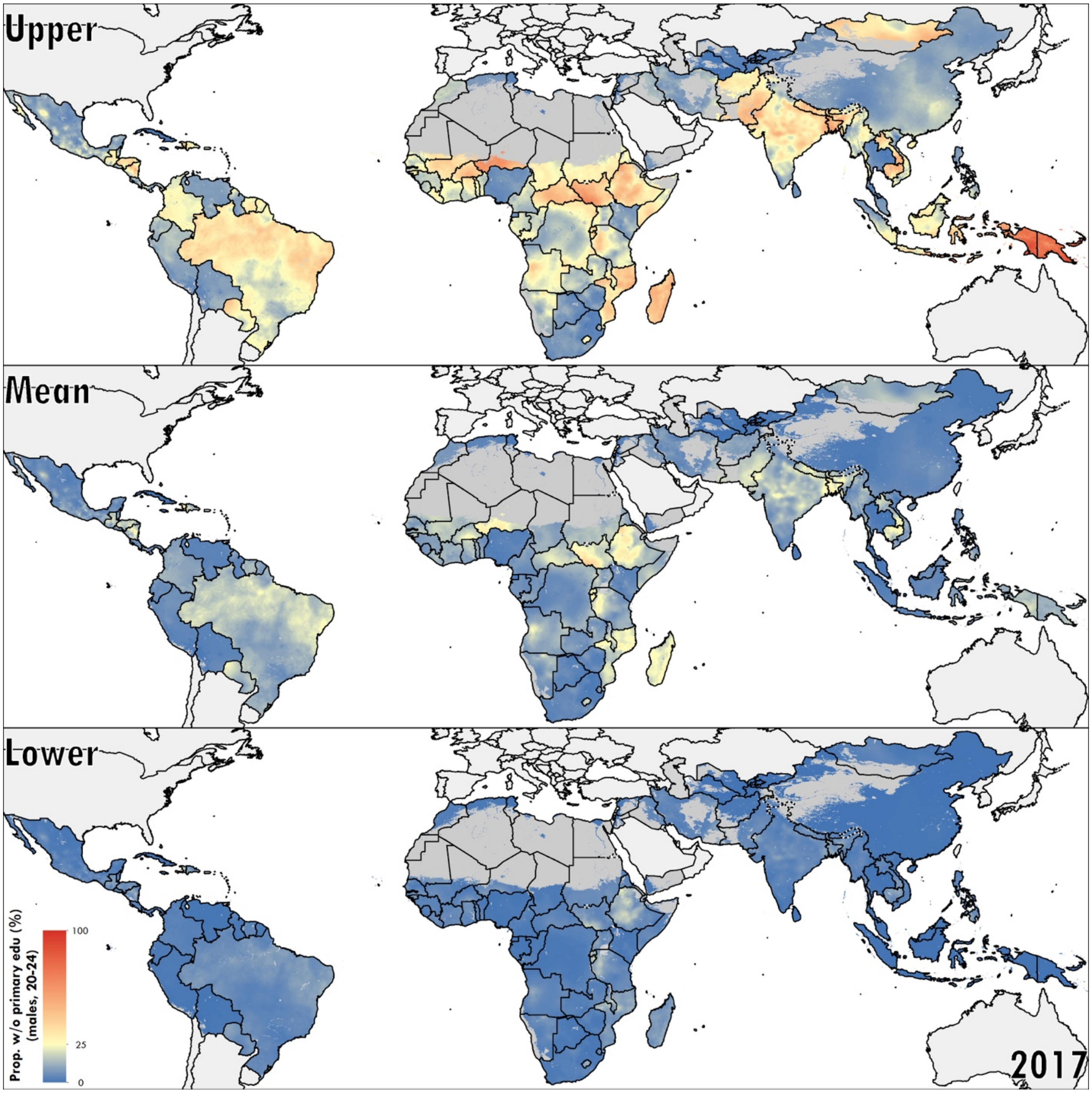

Supplementary Figure 15. 1–5 years, men ages 20–24 posterior means and 95% uncertainty interval, 2017

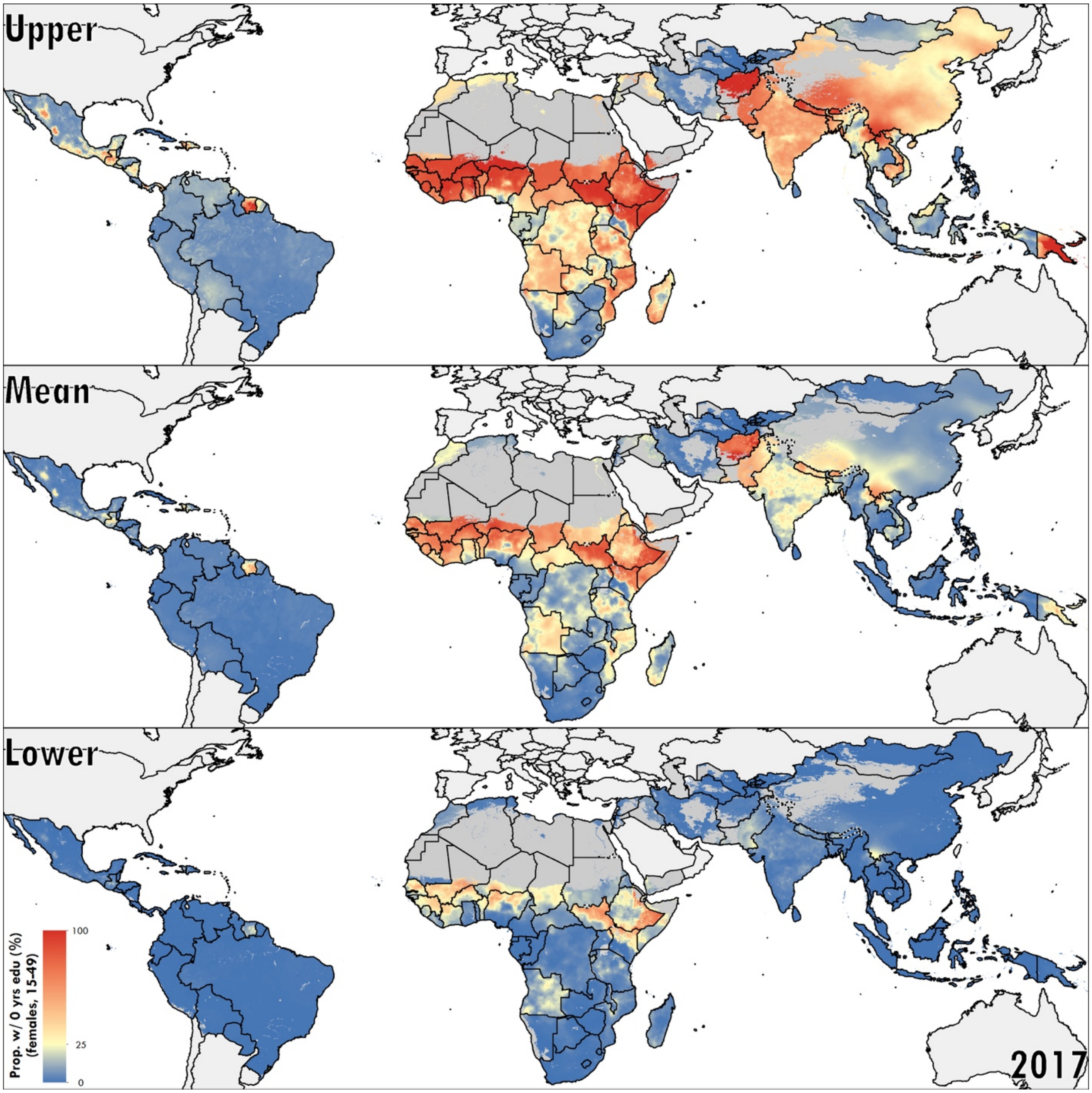

Supplementary Figure 16. 6–11 years, women ages 15–49 posterior means and 95% uncertainty interval, 2017

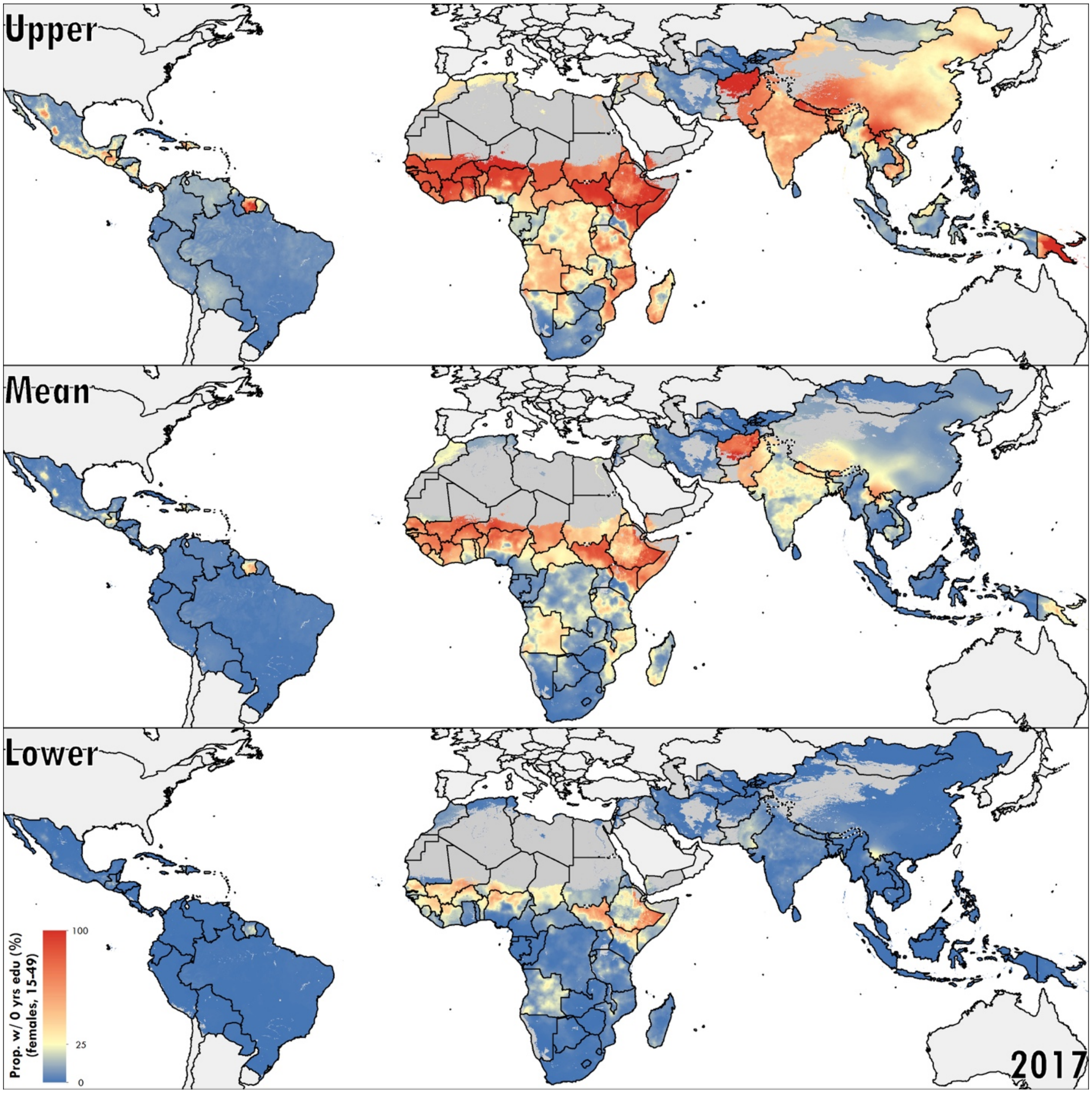

Supplementary Figure 17. 6–11 years, men ages 15–49 posterior means and 95% uncertainty interval, 2017

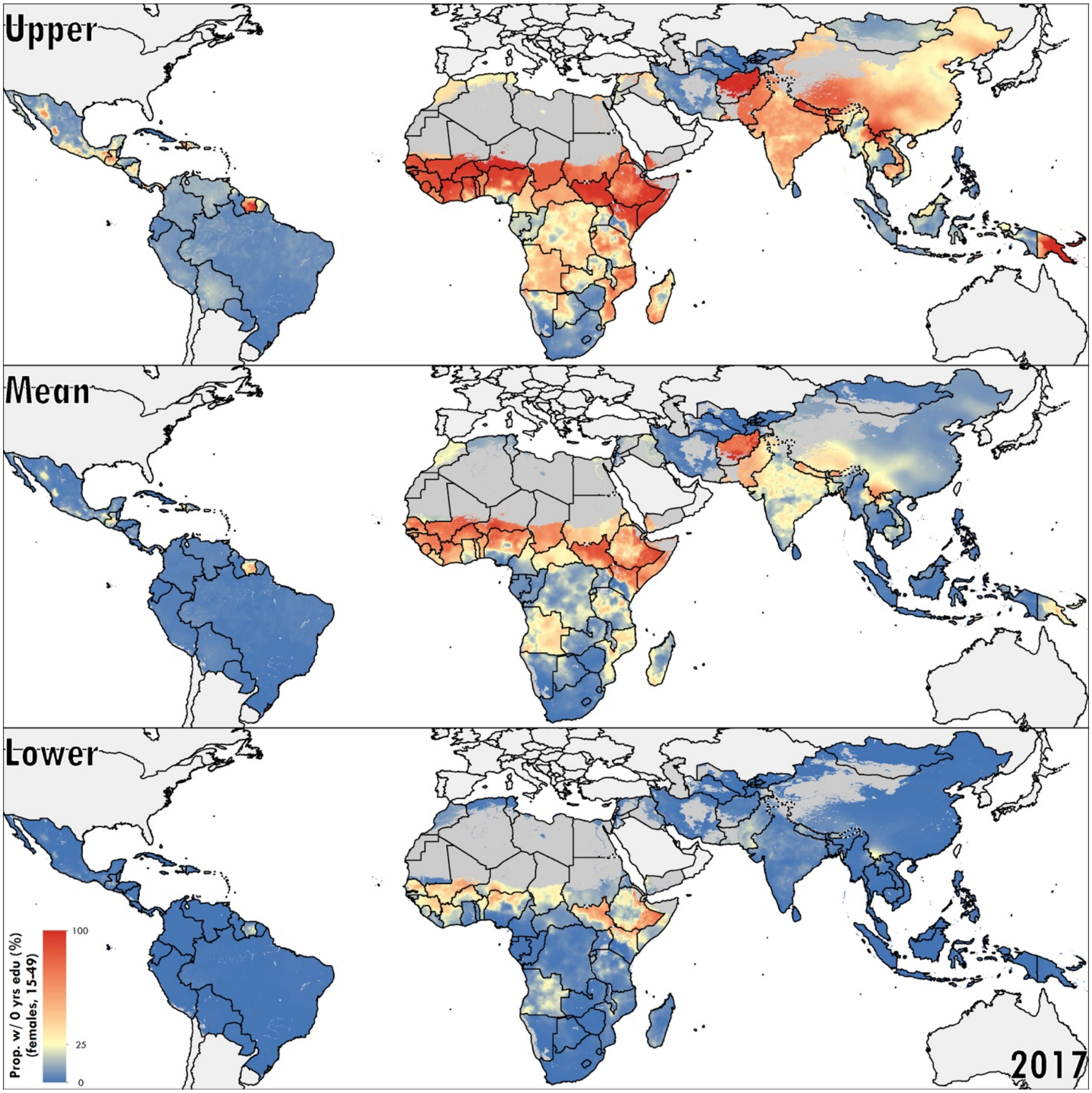

Supplementary Figure 18. 6–11 years, women ages 20–24 posterior means and 95% uncertainty interval, 2017

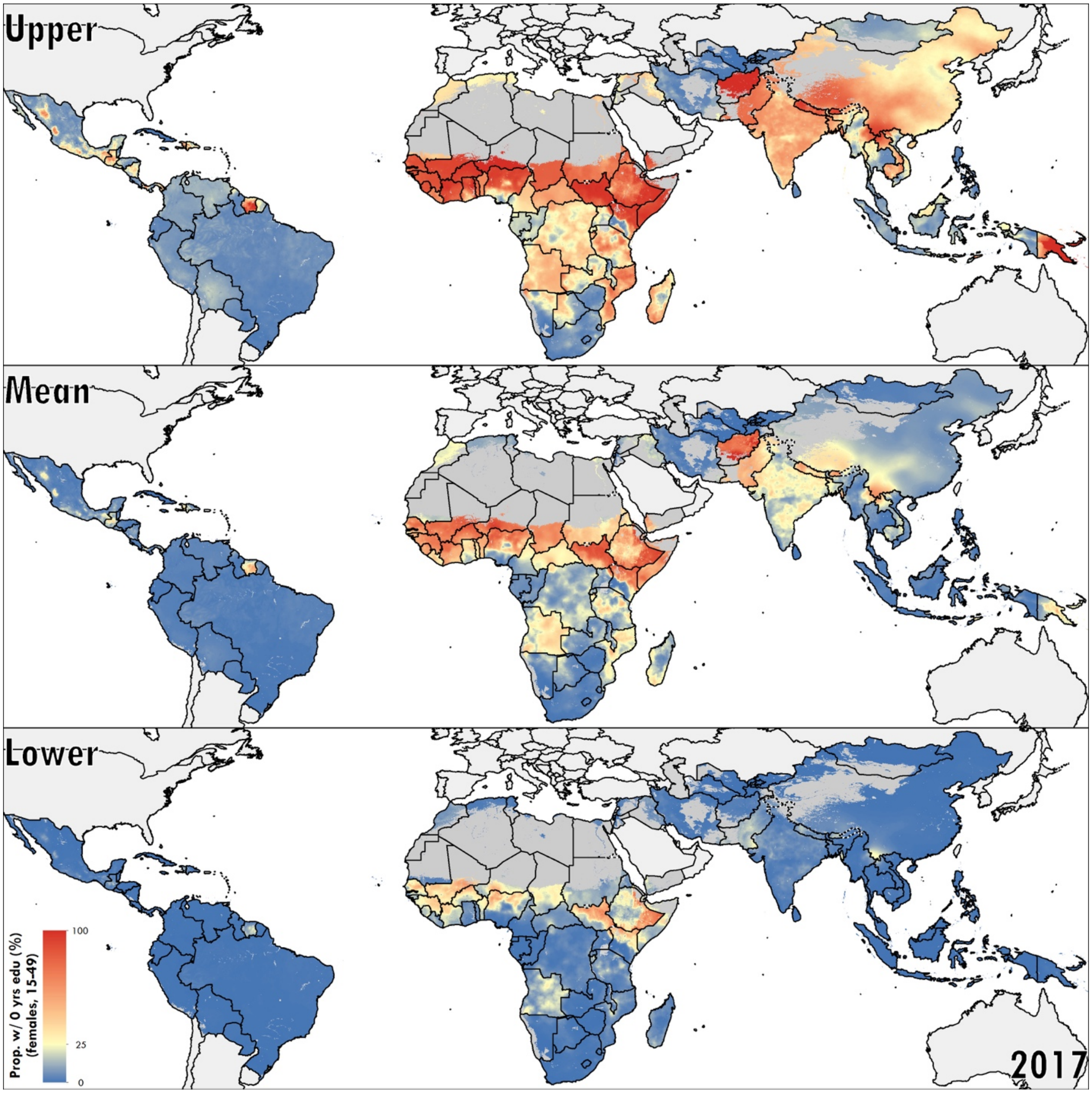

Supplementary Figure 19. 6–11 years, men ages 20–24 posterior means and 95% uncertainty interval, 2017

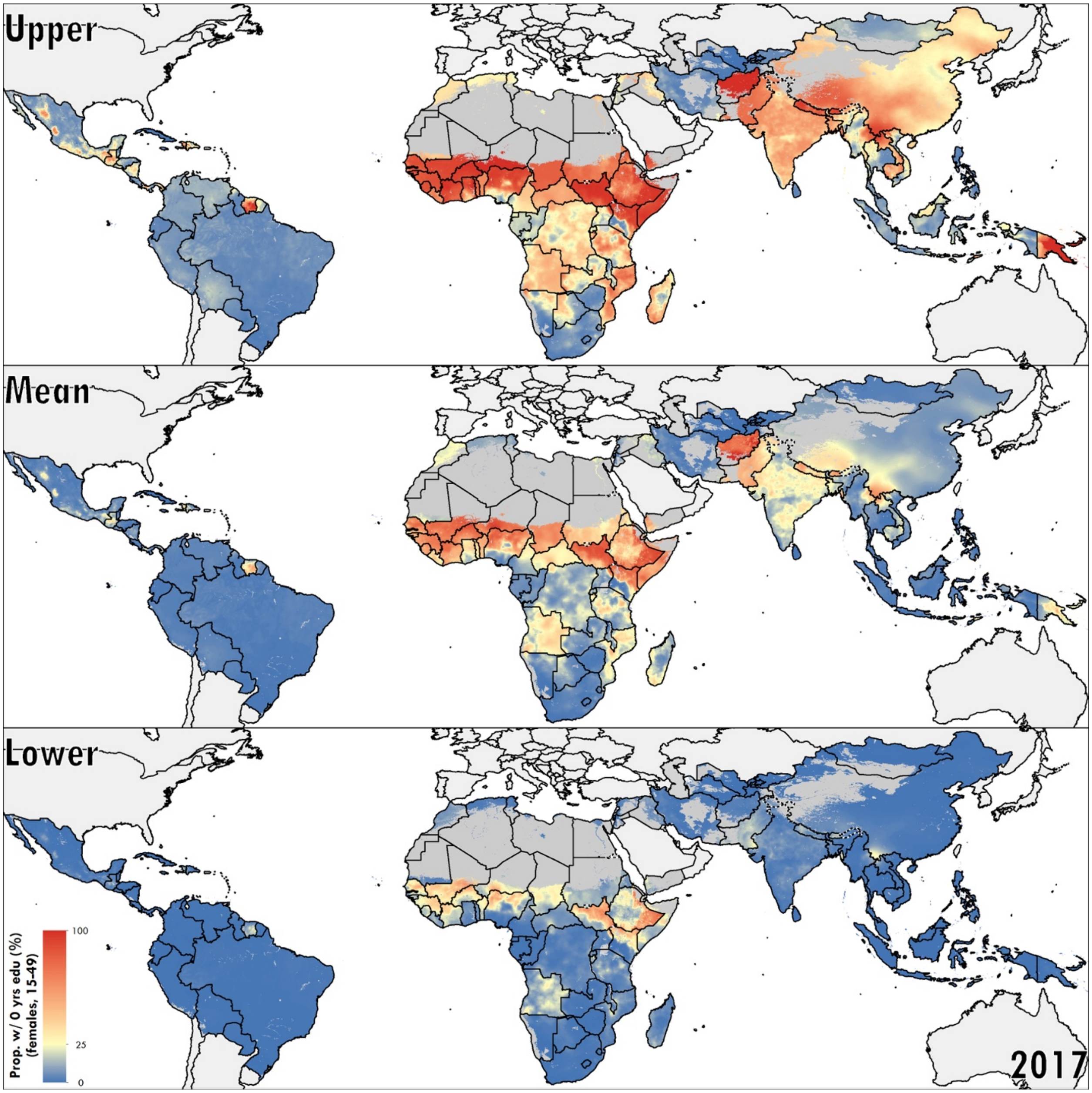

Supplementary Figure 20. 12+ years, women ages 15–49 posterior means and 95% uncertainty interval, 2017

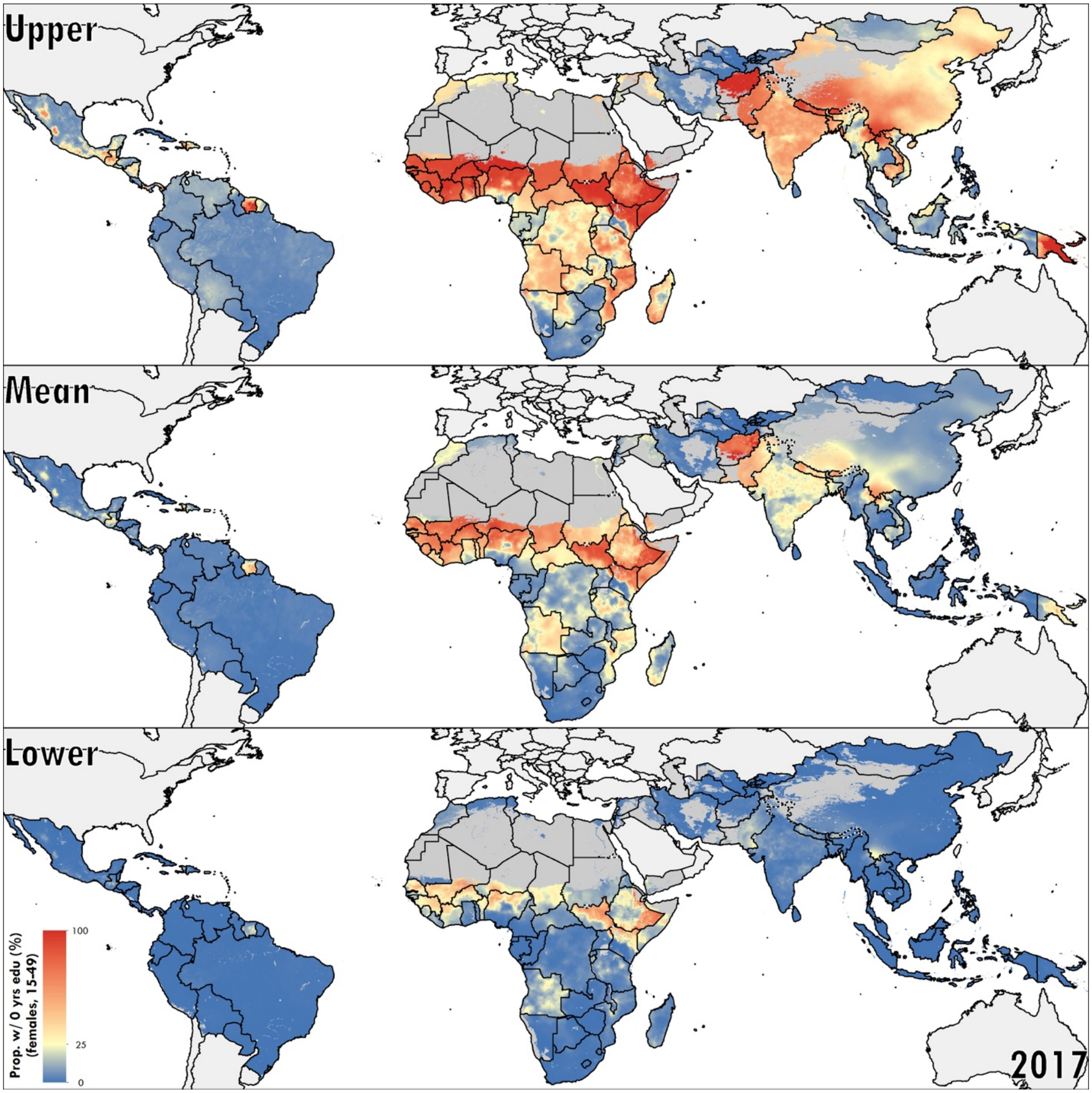

Supplementary Figure 21. 12+ years, men ages 15–49 posterior means and 95% uncertainty interval, 2017

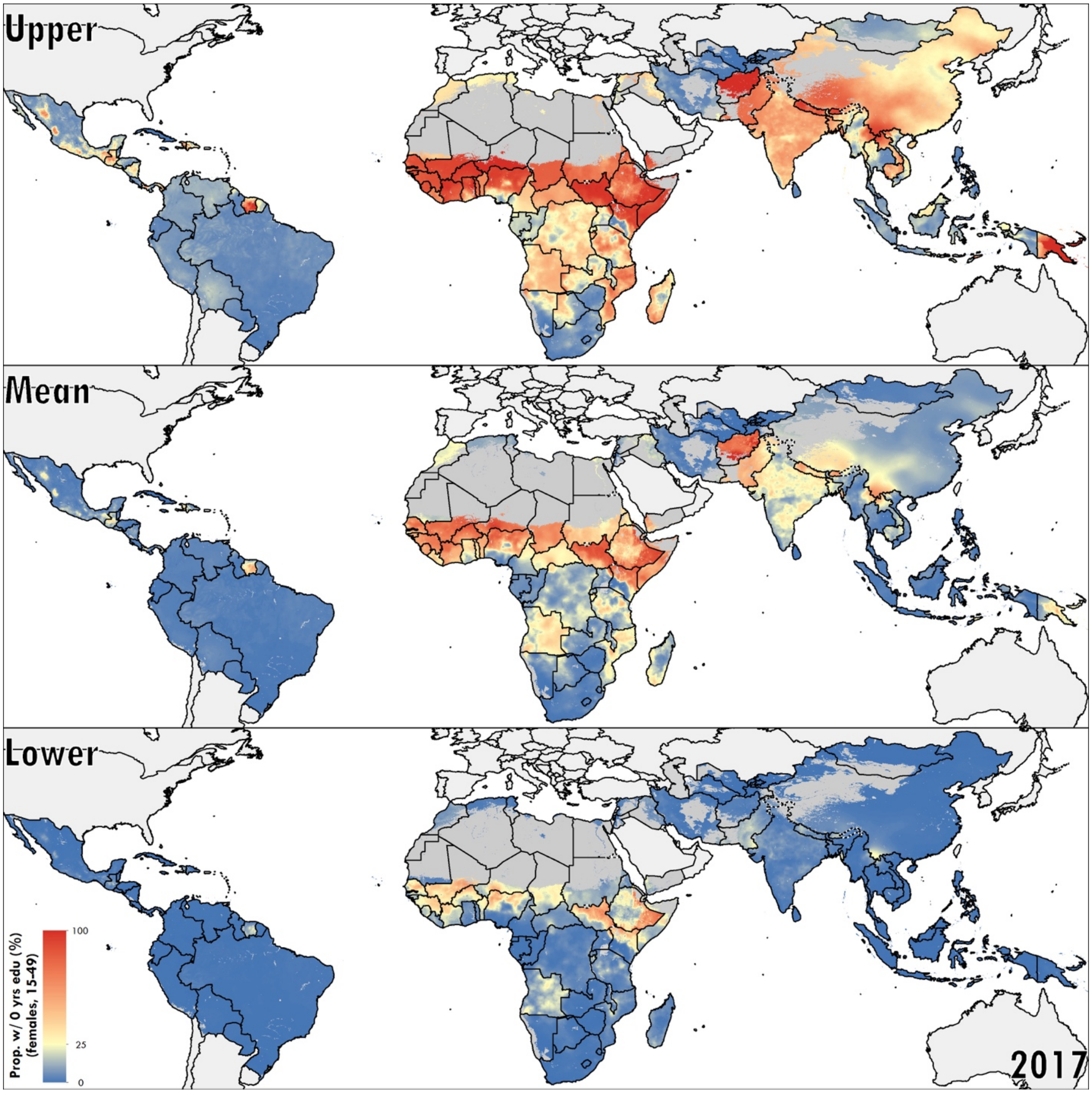

Supplementary Figure 22. 12+ years, women ages 20–24 posterior means and 95% uncertainty interval, 2017

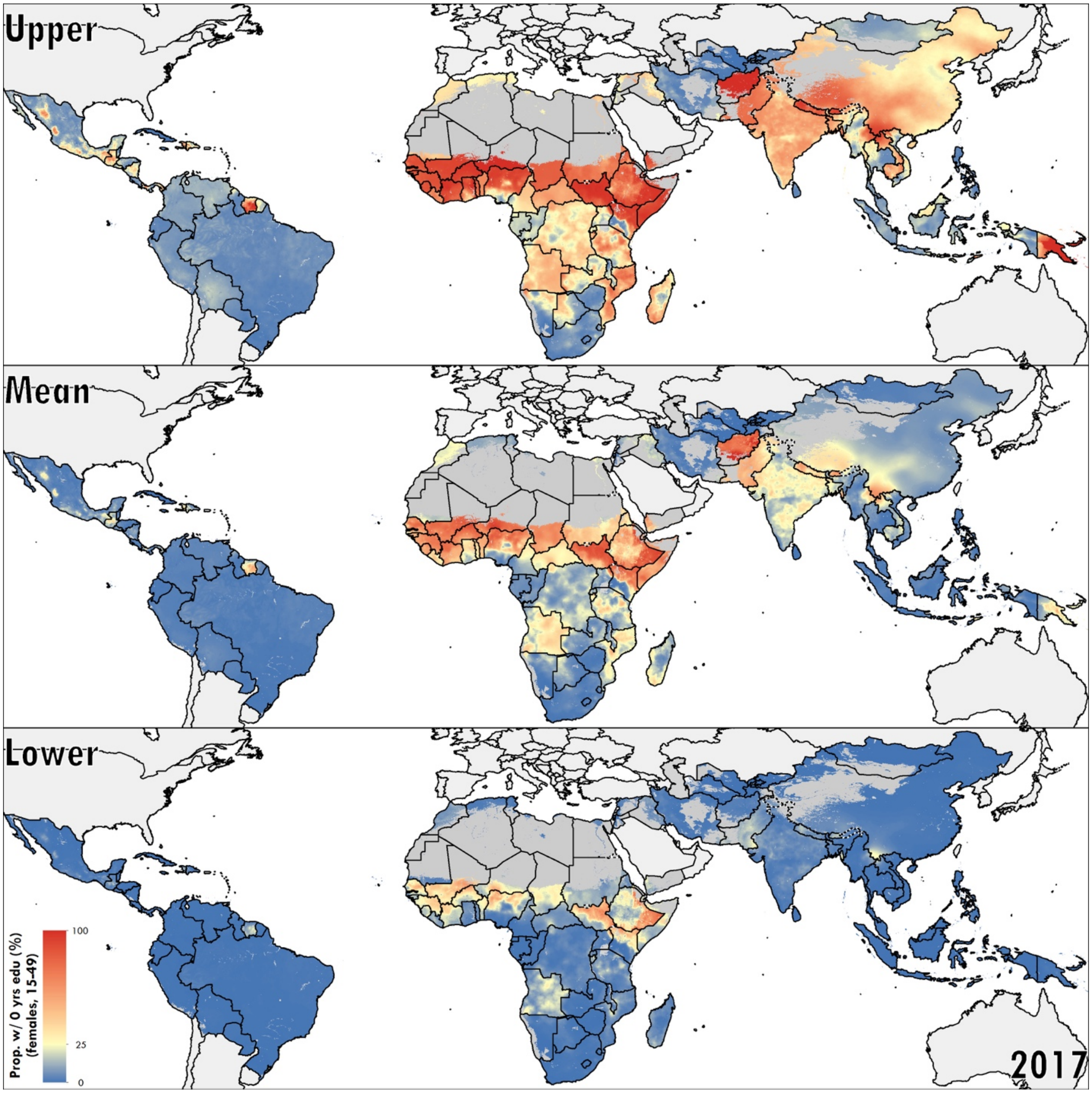

Supplementary Figure 23. 12+ years, men ages 20–24 posterior means and 95% uncertainty interval, 2017

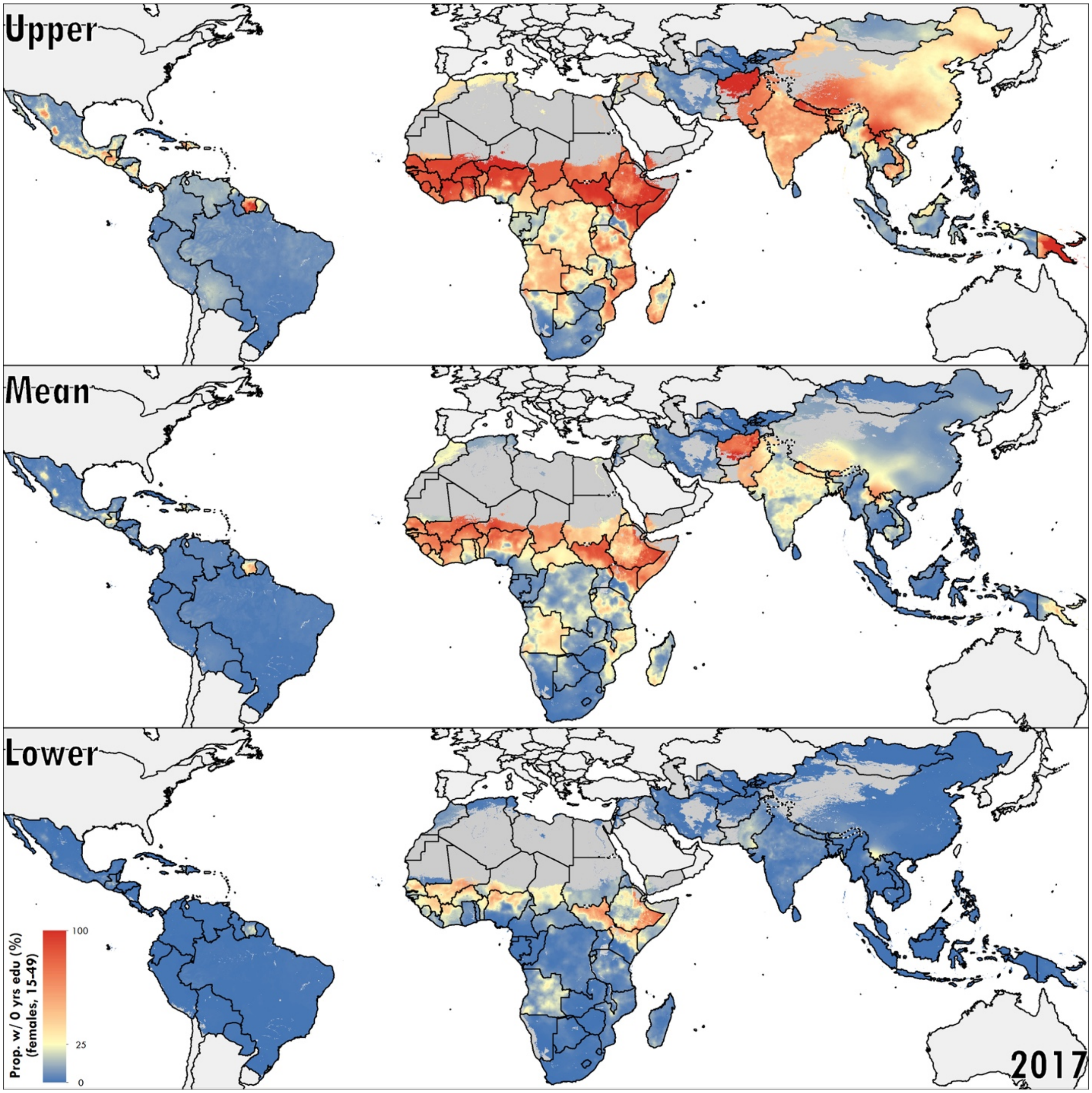

Supplementary Figure 24. Mean years, women ages 15–49 posterior means and 95% uncertainty interval

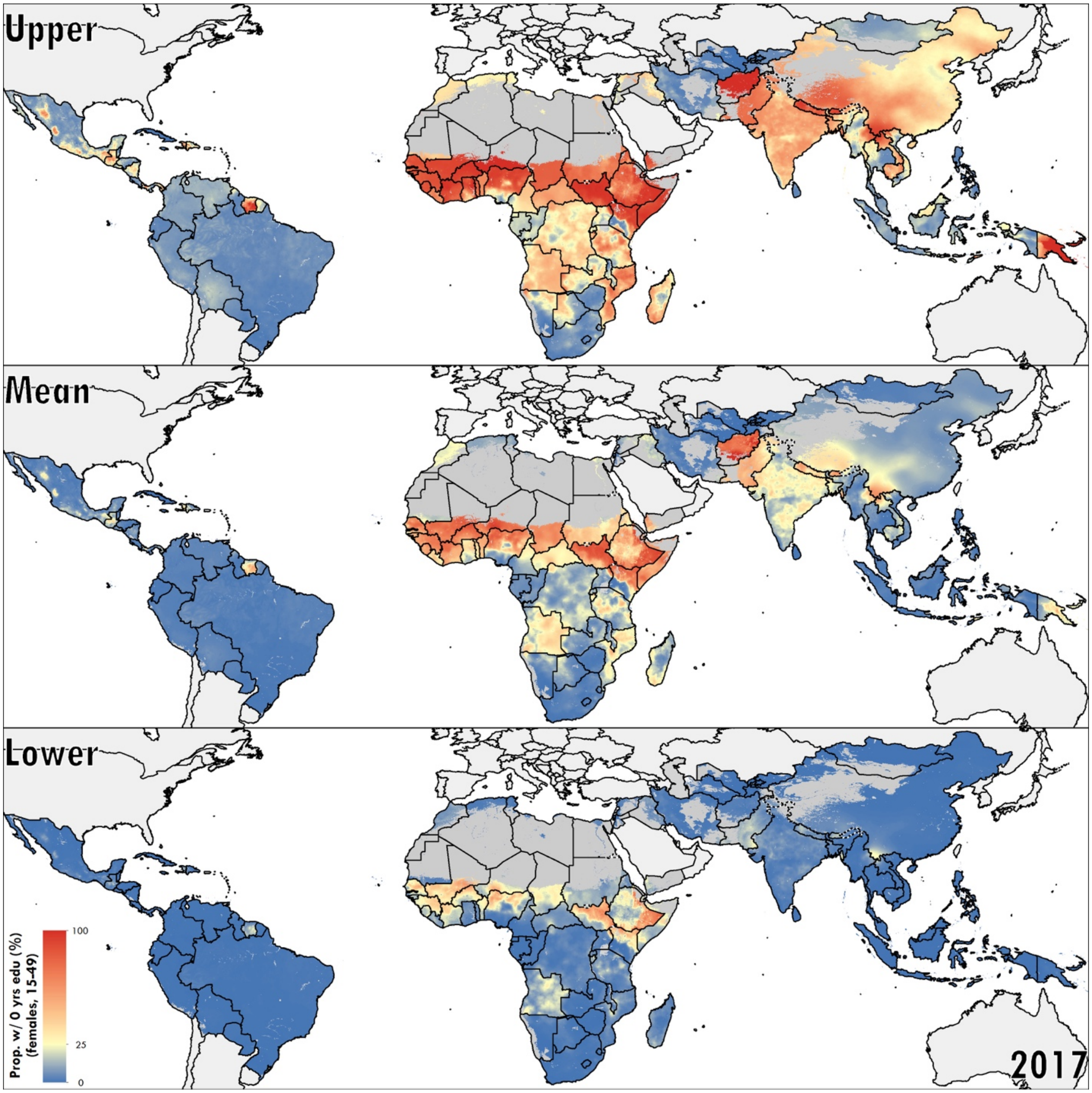

Supplementary Figure 25. Mean years, men ages 15–49 posterior means and 95% uncertainty interval

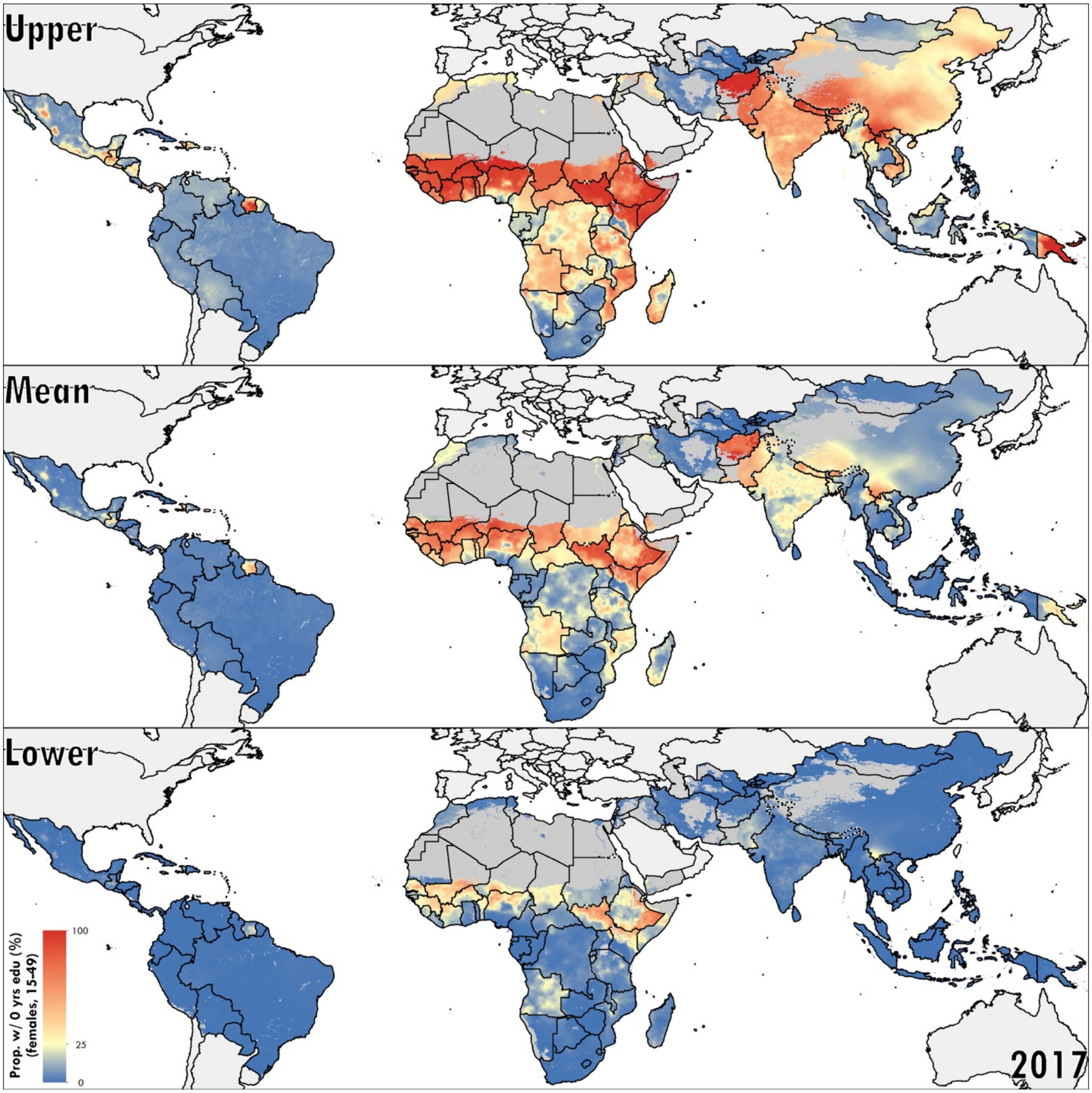

Supplementary Figure 26. Mean years, women ages 20–24 posterior means and 95% uncertainty interval

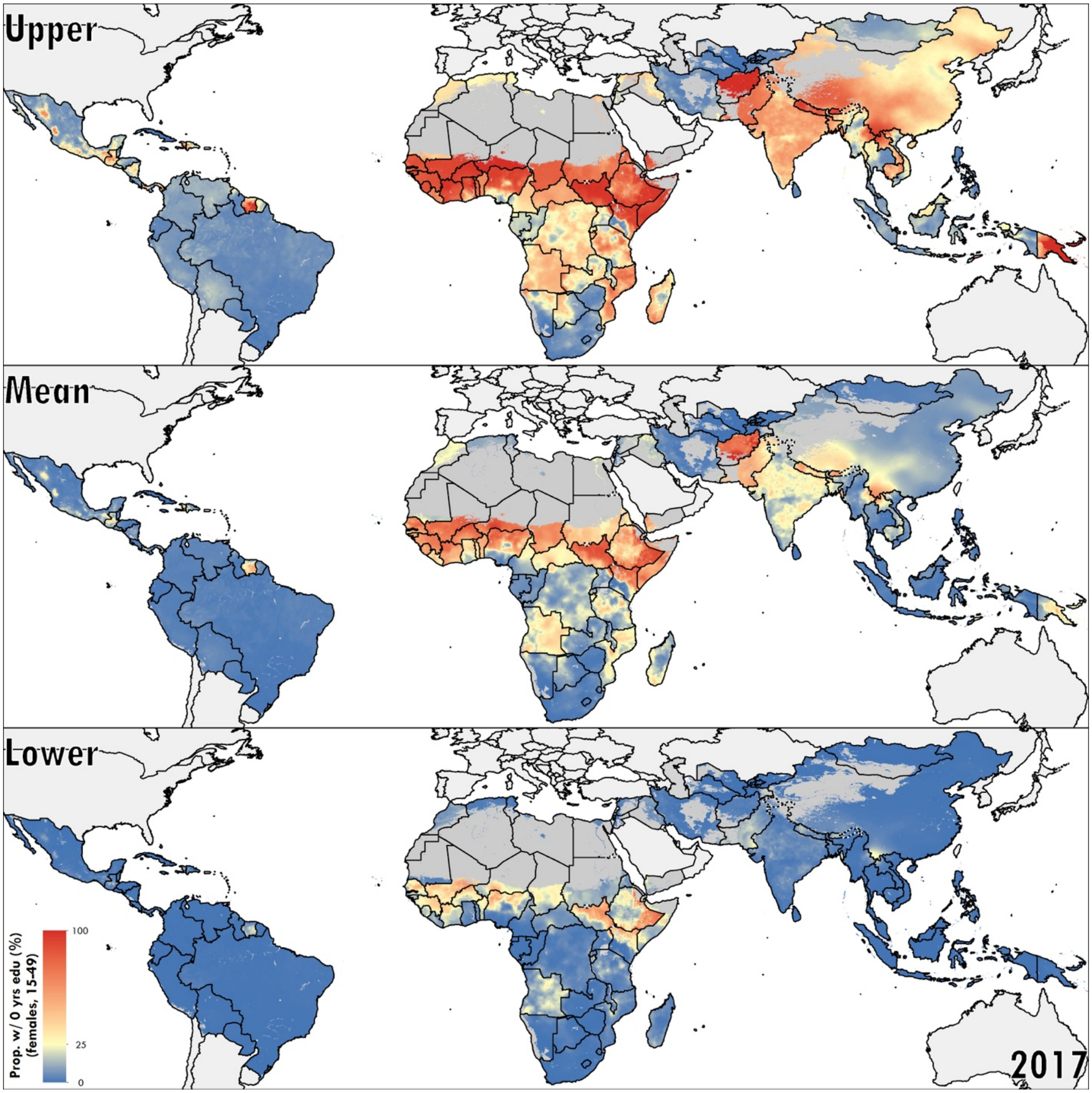

Supplementary Figure 27. Mean years, men ages 20–24 posterior means and 95% uncertainty interval

5.3 Model validation

5.3.1 In-sample metrics

For each indicator, we generated a suite of diagnostic plots for each region and country estimated, in order to assess the in-sample performance of our model and compare to national-level estimates produced by GBD.

To explore residual error over space and time, absolute error (data minus predicted posterior mean estimates at the corresponding pixels) was produced at five-year intervals (2000, 2005, 2010, and 2015) for each modelled region (see Supplementary Figs 28–75).

5.3.2 Metrics of predictive validity

In order to assess the predictive validity of our estimates, we validated our models using spatially stratified five-fold out-of-sample cross-validation. To do so, we first split all survey data into five groups by randomly sorting a list of unique identifiers for each survey, calculating the cumulative number of spatial points represented by the surveys in this list, and then dividing the list into five parts at the point where this number of spatial points was closest to 20%, 40%, 60%, and 80% of the total. This resulted in five groups that were approximately equal in terms of the total number of spatial points and contain entire surveys (i.e., all the data points derived from each survey were contained exclusively within only one fold). These spatial partitions were then allocated to one of five folds for cross-validation. For validation, each geostatistical model was run five times, each time holding out data from one of the folds, generating a set of out-of-sample predictions for the held-out data. For each indicator, a full suite of out-of-sample predictions over the entire dataset was generated by combing the out-of-sample predictions from the five cross-validation runs.

Using these out-of-sample predictions, we then calculated mean error (ME, or bias), root-mean-squared-error (RMSE, which summarises total variance), and 95% coverage of our predictive intervals (the proportion of observed out-of-sample data that fall within our predicted 95% credible intervals) aggregated up to different administrative levels (0, 1, and 2) as defined by FAO Global Administrative Unit Layers (GAUL). Administrative level 0 (admin 0) borders correspond to national boundaries, admin 1 borders generally correspond to regions, provinces, or state-level boundaries within a country, and admin 2 borders correspond to the next finer subdivision, often districts, within regions. These metrics are summarised in Supplementary Tables 24–43 for each indicator and are calculated across all regions. Included in the sample tables for comparison are the same metrics calculated on in-sample predictions.

Supplementary Table 24. In sample predictive metrics for zero years of education (women, ages 15–49), aggregated to different administrative levels (admin 0, admin 1, and admin 2)

Aggregation	Year	Median SS	Mean Err	RMSE	Corr.	95% Cov.
Admin 0	2000	12914.7806	0.0064	0.0113	0.9980	0.9116
Admin 0	2005	15225.4068	0.0069	0.0183	0.9971	0.9505
Admin 0	2010	19025.0000	-0.0011	0.0020	0.9993	0.9925
Admin 0	2015	13782.0000	0.0018	0.0050	0.9996	0.9574
Admin 1	2000	1228.0000	0.0064	0.0162	0.9960	0.9116
Admin 1	2005	750.0000	0.0069	0.0274	0.9942	0.9505
Admin 1	2010	1077.8178	-0.0011	0.0049	0.9966	0.9925
Admin 1	2015	716.5000	0.0018	0.0126	0.9971	0.9574
Admin 2	2000	114.9720	0.0064	0.0307	0.9858	0.9116
Admin 2	2005	31.0000	0.0069	0.0510	0.9801	0.9505
Admin 2	2010	41.1510	-0.0011	0.0092	0.9883	0.9925
Admin 2	2015	31.6316	0.0018	0.0308	0.9846	0.9574

Supplementary Table 25. In sample predictive metrics for zero years of education (men, ages 15–49), aggregated to different administrative levels (admin 0, admin 1, and admin2)

Aggregation	Year	Median SS	Mean Err	RMSE	Corr.	95% Cov.
Admin 0	2000	11706.6693	0.0049	0.0083	0.9975	0.9105
Admin 0	2005	13164.3894	0.0050	0.0116	0.9978	0.9441
Admin 0	2010	17541.0000	0.0007	0.0010	0.9996	0.9675
Admin 0	2015	12977.0000	0.0016	0.0034	0.9995	0.9562
Admin 1	2000	1126.0000	0.0049	0.0121	0.9955	0.9105
Admin 1	2005	690.0000	0.0050	0.0187	0.9943	0.9441
Admin 1	2010	1028.4576	0.0007	0.0022	0.9986	0.9675
Admin 1	2015	671.0000	0.0016	0.0086	0.9962	0.9562
Admin 2	2000	111.7156	0.0049	0.0270	0.9791	0.9105
Admin 2	2005	28.8900	0.0050	0.0386	0.9758	0.9441
Admin 2	2010	38.1188	0.0007	0.0047	0.9932	0.9675
Admin 2	2015	30.0000	0.0016	0.0247	0.9732	0.9562

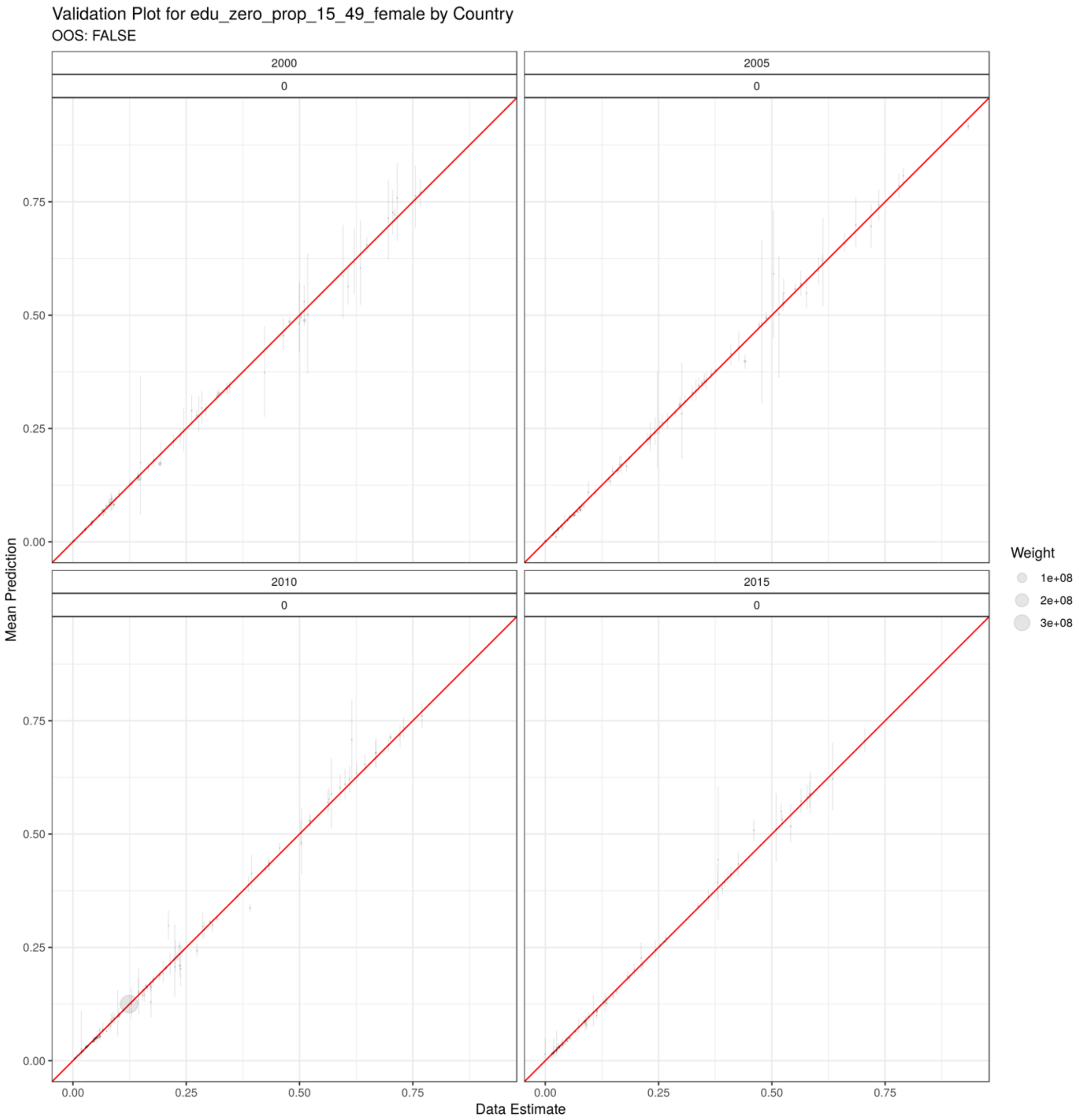

Supplementary Figure 28. Zero years education (women, ages 15–49) admin 0 aggregation
Comparison of in-sample education predictions aggregated to admin 0 with 95% uncertainty intervals plotted against admin 1 aggregated data observations.

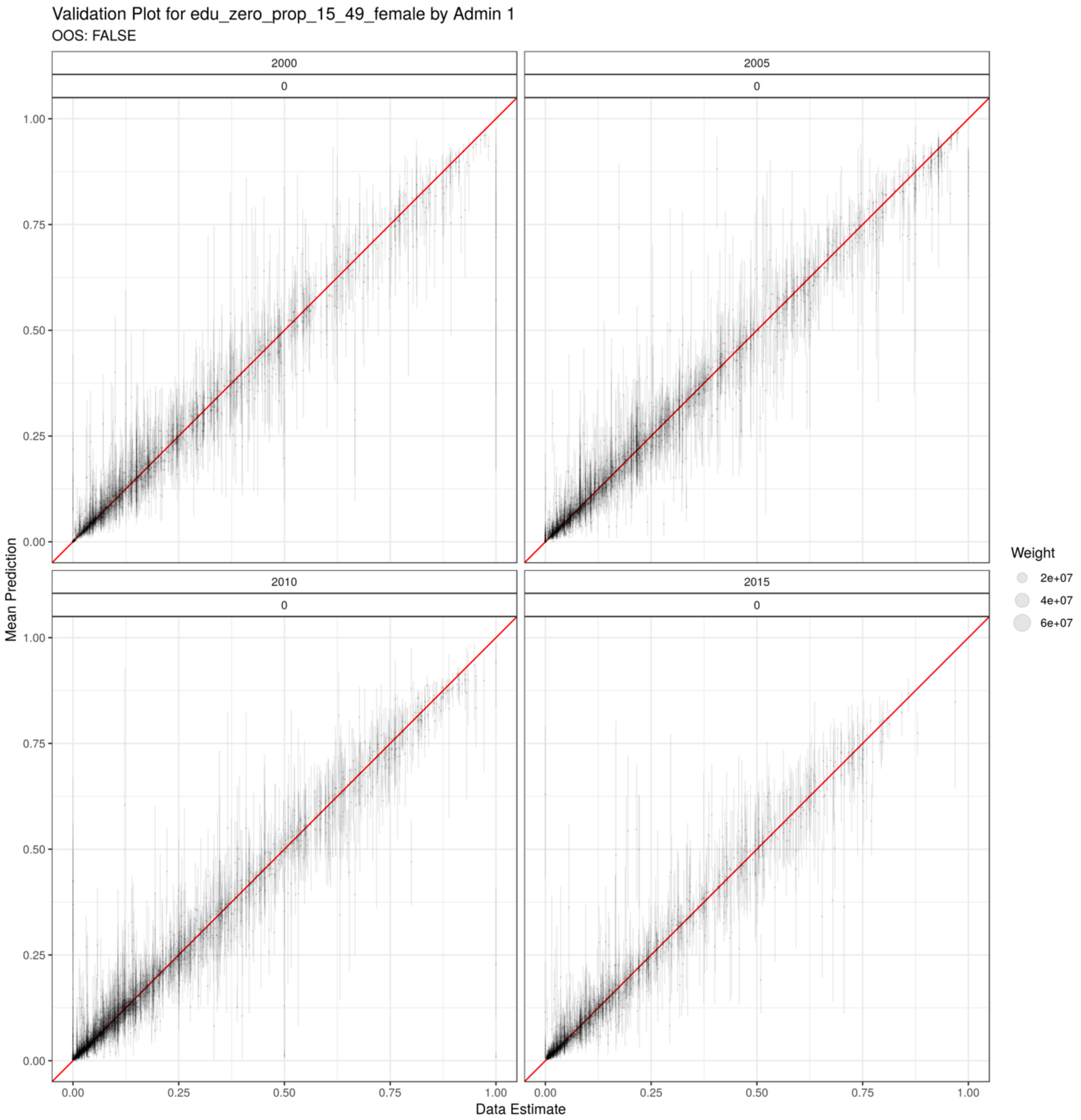

Supplementary Figure 29. Zero years education (women, ages 15–49) admin 1 aggregation
Comparison of in-sample education predictions aggregated to admin 1 with 95% uncertainty intervals plotted against admin 1 aggregated data observations.

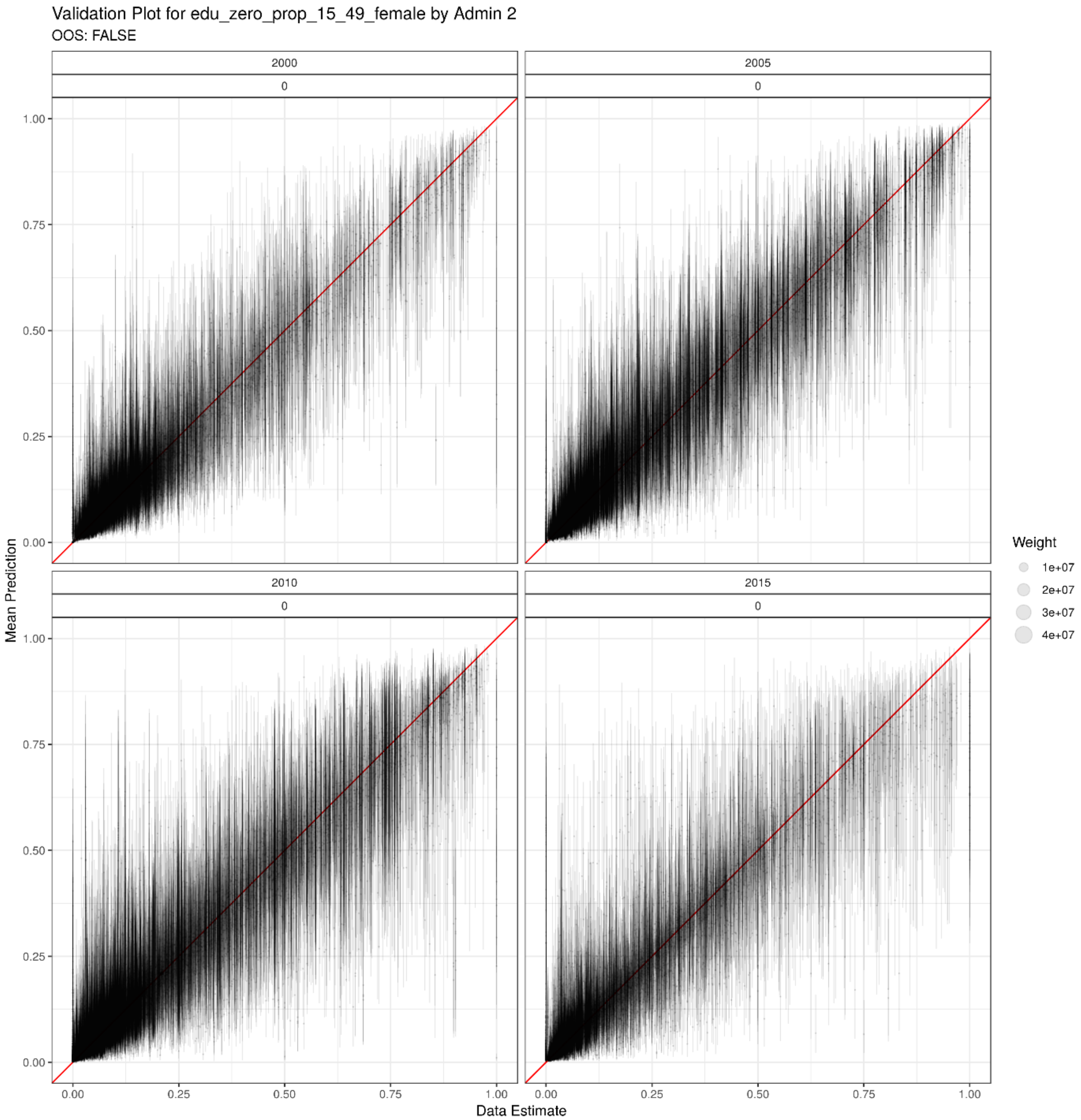

Supplementary Figure 30. Zero years education (women, ages 15–49) admin 2 aggregation
Comparison of in-sample education predictions aggregated to admin 2 with 95% uncertainty intervals plotted against admin 1 aggregated data observations.

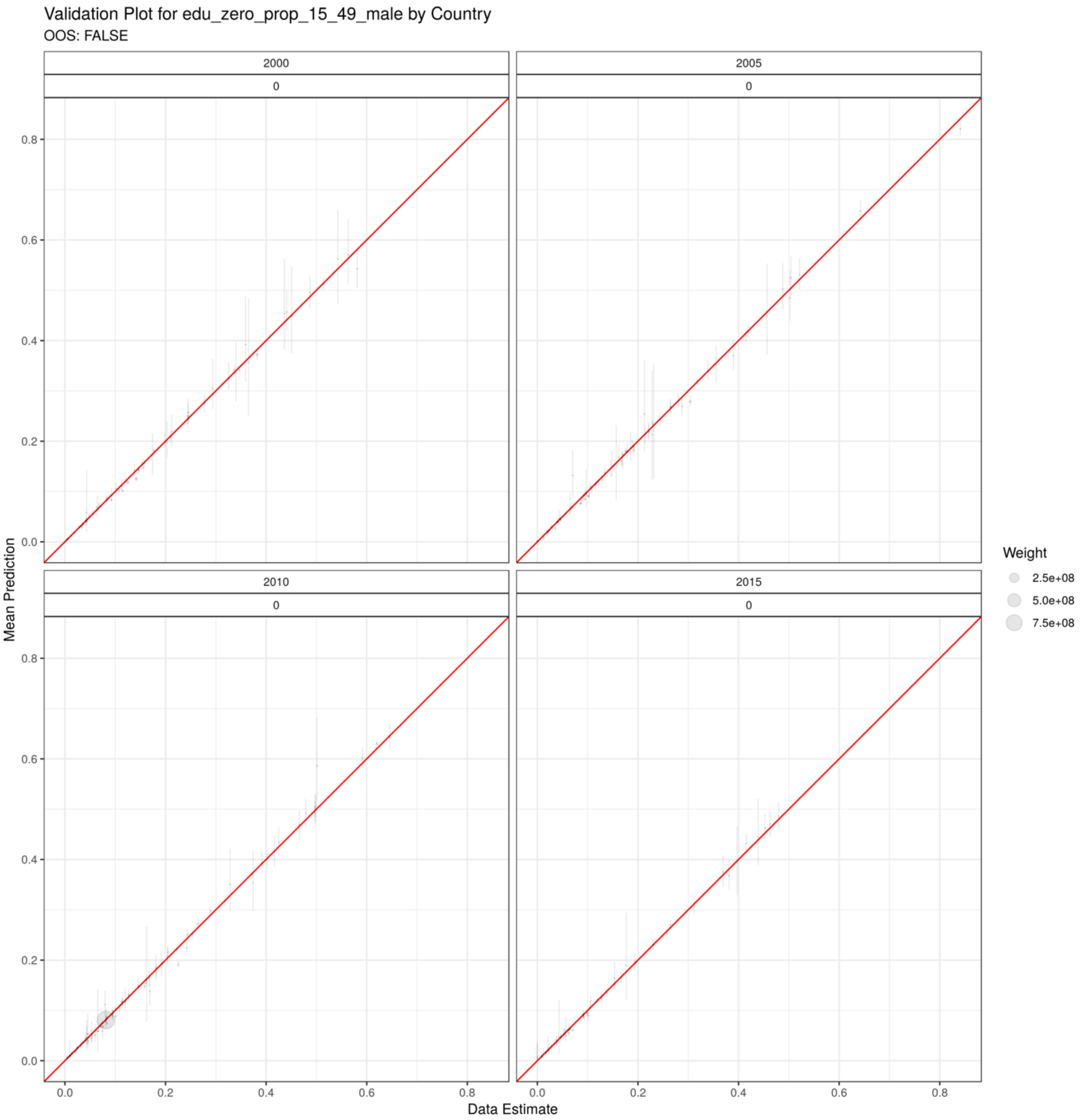

Supplementary Figure 31. Zero years education (men, ages 15–49) admin 0 aggregation
Comparison of in-sample education predictions aggregated to admin 0 with 95% uncertainty intervals plotted against admin 1 aggregated data observations.

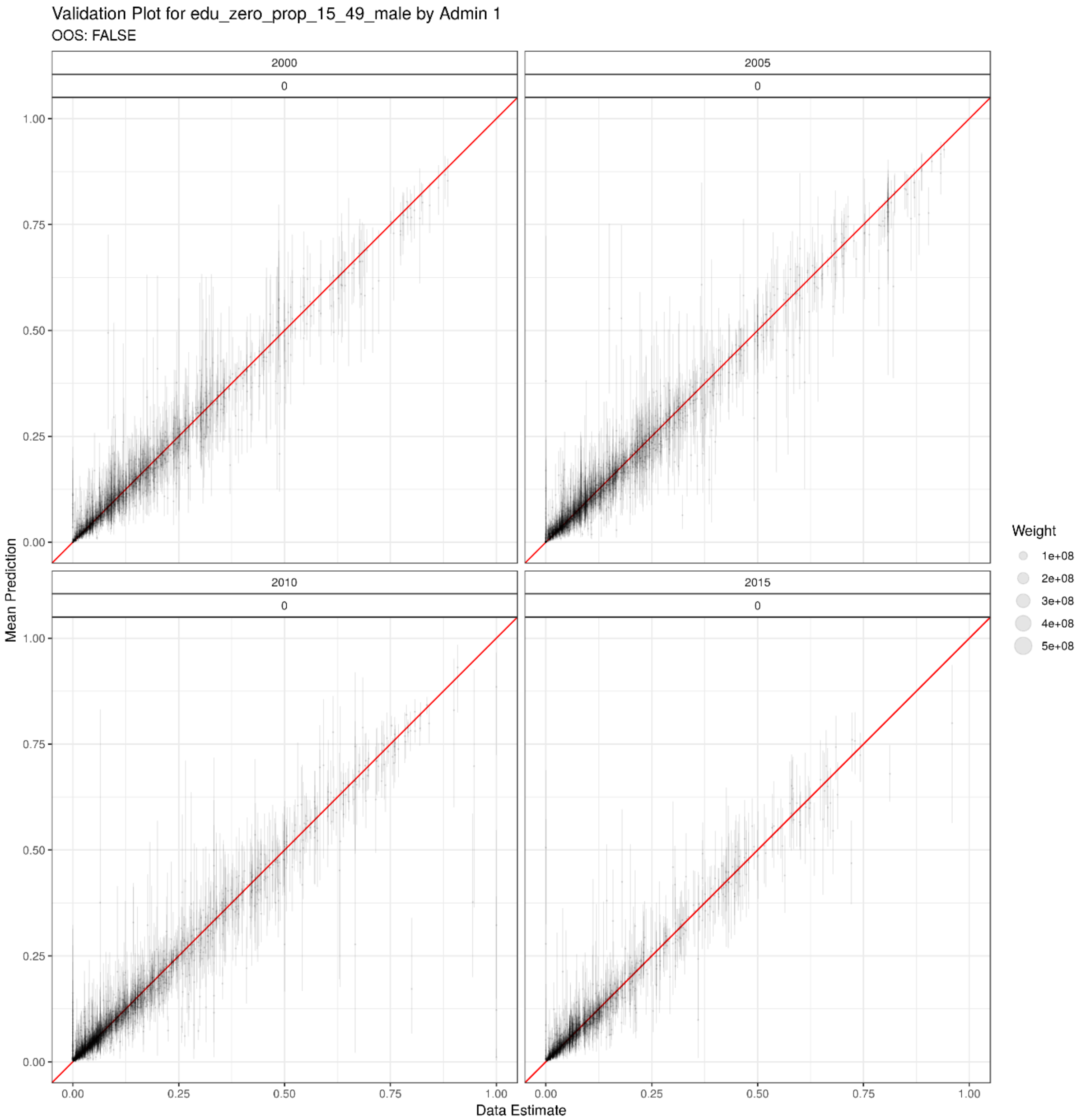

Supplementary Figure 32. Zero years education (men, ages 15–49) admin 1 aggregation
Comparison of in-sample education predictions aggregated to admin 1 with 95% uncertainty intervals plotted against admin 1 aggregated data observations.

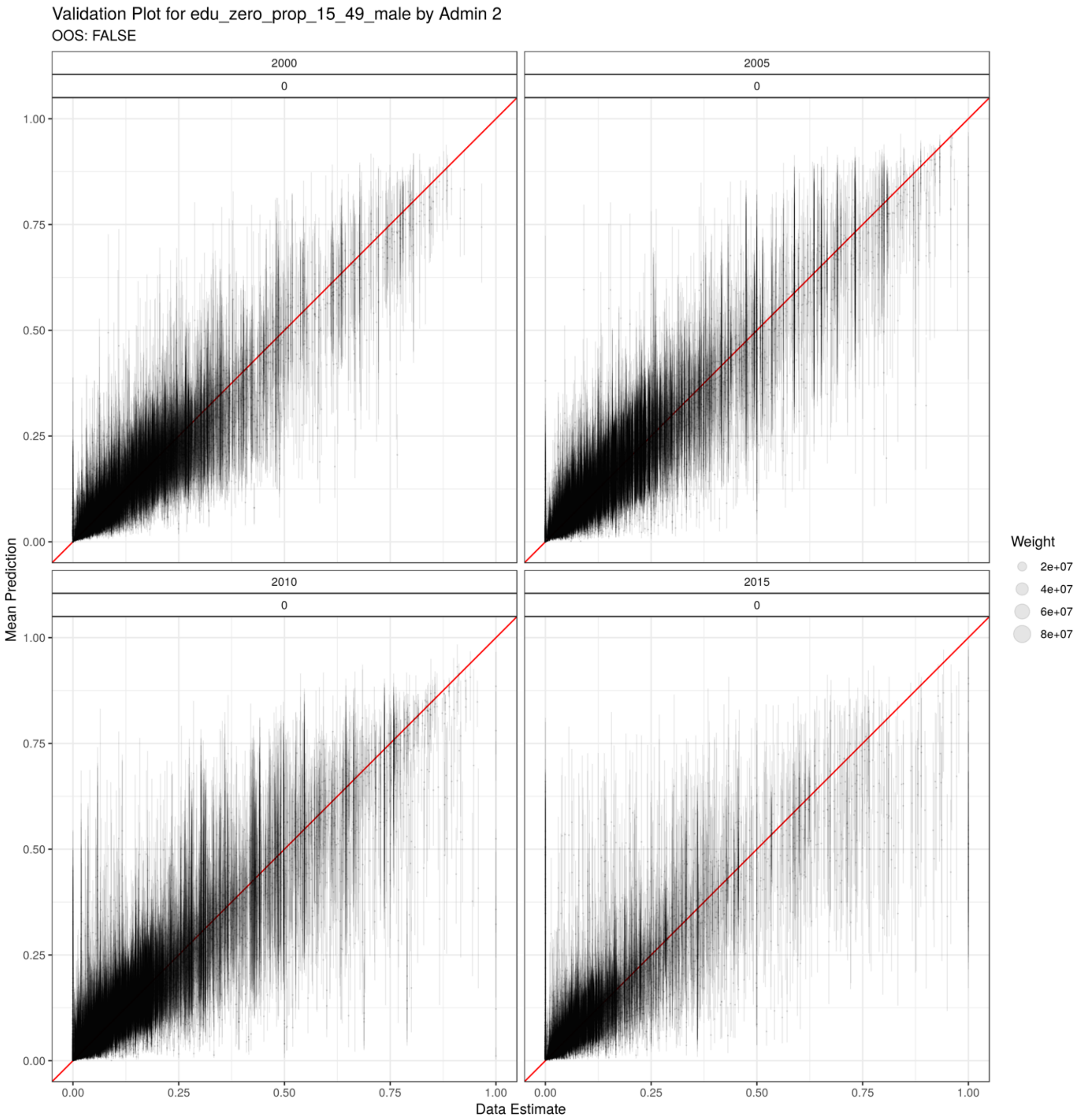

Supplementary Figure 33. Zero years education (men, ages 15–49) admin 2 aggregation
Comparison of in-sample education predictions aggregated to admin 2 with 95% uncertainty intervals plotted against admin 1 aggregated data observations.

Supplementary Table 26. In sample predictive metrics for zero years of education (women, ages 20-24), aggregated to admin 0, admin 1, and admin 2

Aggregation	Year	Median SS	Mean Err	RMSE	Corr.	95% Cov.
Admin 0	2000	2528.1849	0.0014	0.0062	0.9991	0.9214
Admin 0	2005	2962.2179	0.0034	0.0114	0.9983	0.9470
Admin 0	2010	3493.0000	-0.0003	0.0013	0.9998	0.9587
Admin 0	2015	2362.0000	-0.0007	0.0033	0.9998	0.9707
Admin 1	2000	215.0000	0.0014	0.0108	0.9975	0.9214
Admin 1	2005	138.0000	0.0034	0.0195	0.9961	0.9470
Admin 1	2010	190.0000	-0.0003	0.0045	0.9990	0.9587
Admin 1	2015	128.0000	-0.0007	0.0131	0.9958	0.9707
Admin 2	2000	19.0000	0.0014	0.0238	0.9887	0.9214
Admin 2	2005	5.0471	0.0034	0.0449	0.9808	0.9470
Admin 2	2010	6.2313	-0.0003	0.0106	0.9946	0.9587
Admin 2	2015	4.8115	-0.0007	0.0350	0.9738	0.9707

Supplementary Table 27. In sample predictive metrics for zero years of education (men, ages 20–24), aggregated to admin 0, admin 1, and admin 2

Aggregation	Year	Median SS	Mean Err	RMSE	Corr.	95% Cov.
Admin 0	2000	2141.7815	0.0002	0.0039	0.9990	0.9104
Admin 0	2005	2206.0000	0.0027	0.0066	0.9988	0.9398
Admin 0	2010	2828.0000	0.0003	0.0007	0.9998	0.9666
Admin 0	2015	2306.0000	-0.0001	0.0019	0.9996	0.9676
Admin 1	2000	192.0000	0.0002	0.0084	0.9965	0.9104
Admin 1	2005	119.5162	0.0027	0.0154	0.9942	0.9398
Admin 1	2010	174.1984	0.0003	0.0019	0.9987	0.9666
Admin 1	2015	113.0000	-0.0001	0.0101	0.9926	0.9676
Admin 2	2000	18.9655	0.0002	0.0228	0.9785	0.9104
Admin 2	2005	4.9025	0.0027	0.0372	0.9691	0.9398
Admin 2	2010	5.9620	0.0003	0.0049	0.9918	0.9666
Admin 2	2015	4.3568	-0.0001	0.0299	0.9477	0.9676

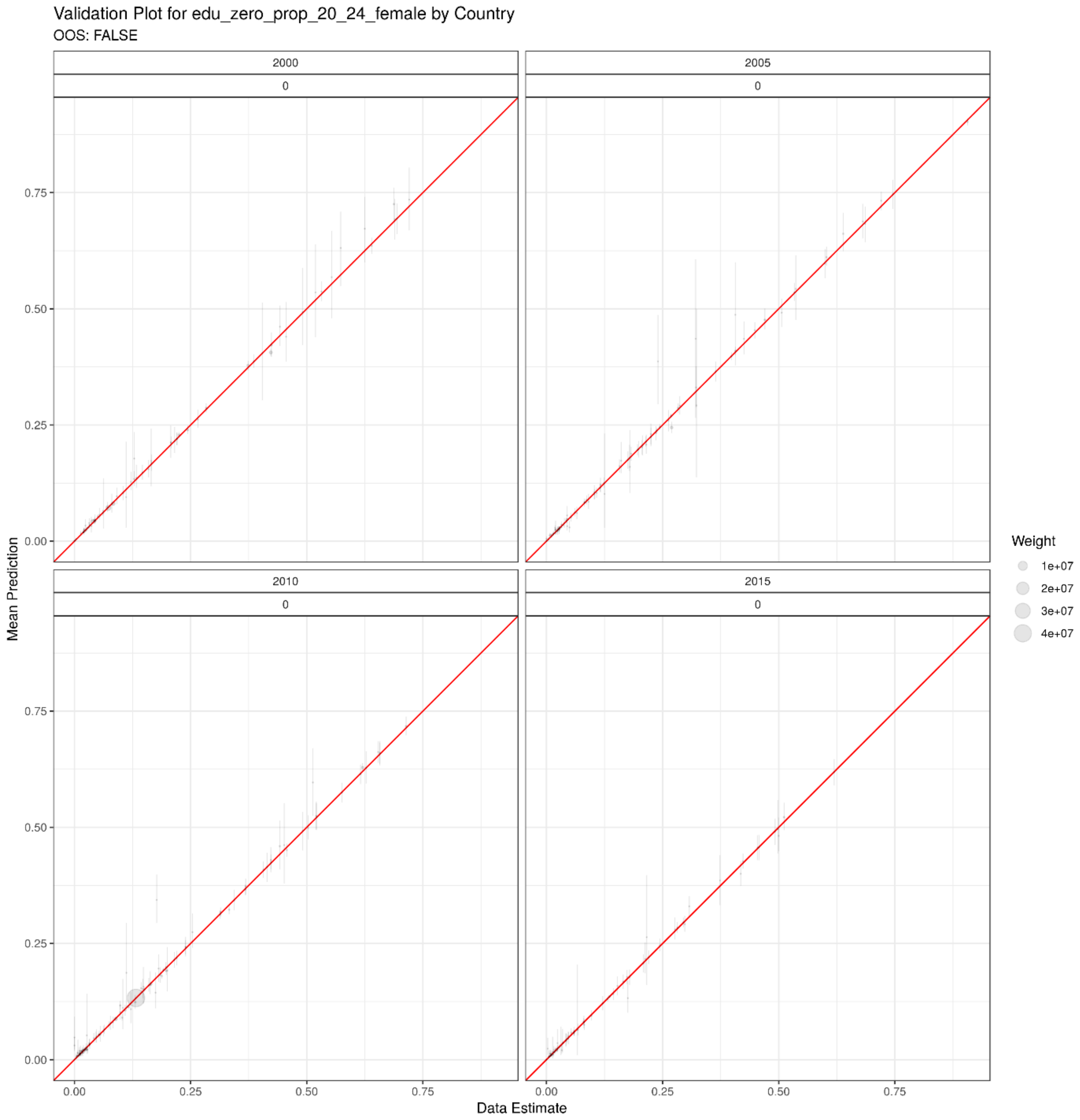

Supplementary Figure 34. Zero years education (women, ages 20–24) admin 0 aggregation
Comparison of in-sample education predictions aggregated to admin 0 with 95% uncertainty intervals plotted against admin 1 aggregated data observations.

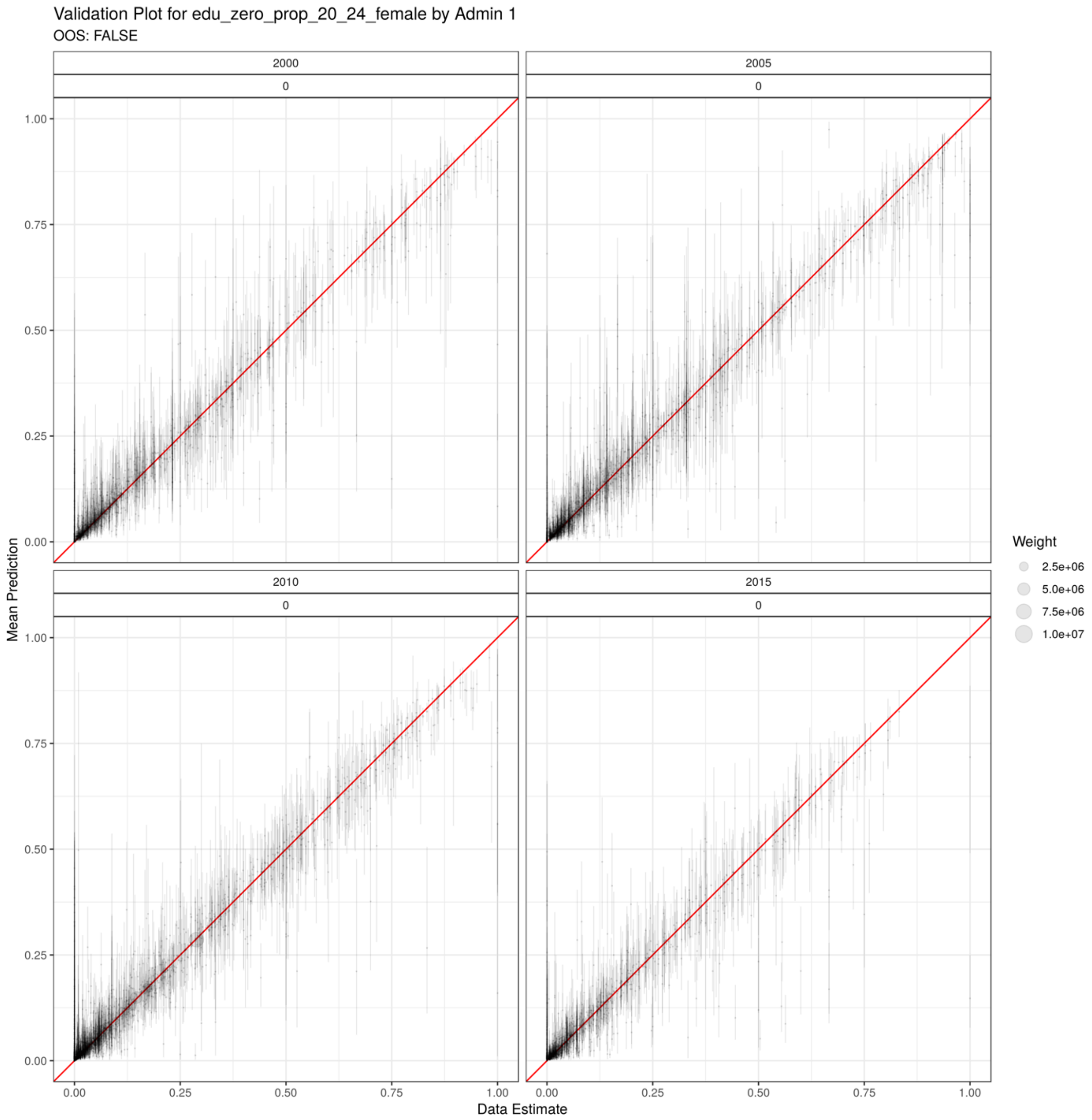

Supplementary Figure 35. Zero years education (women, ages 20–24) admin 1 aggregation
Comparison of in-sample education predictions aggregated to admin 1 with 95% uncertainty intervals plotted against admin 1 aggregated data observations.

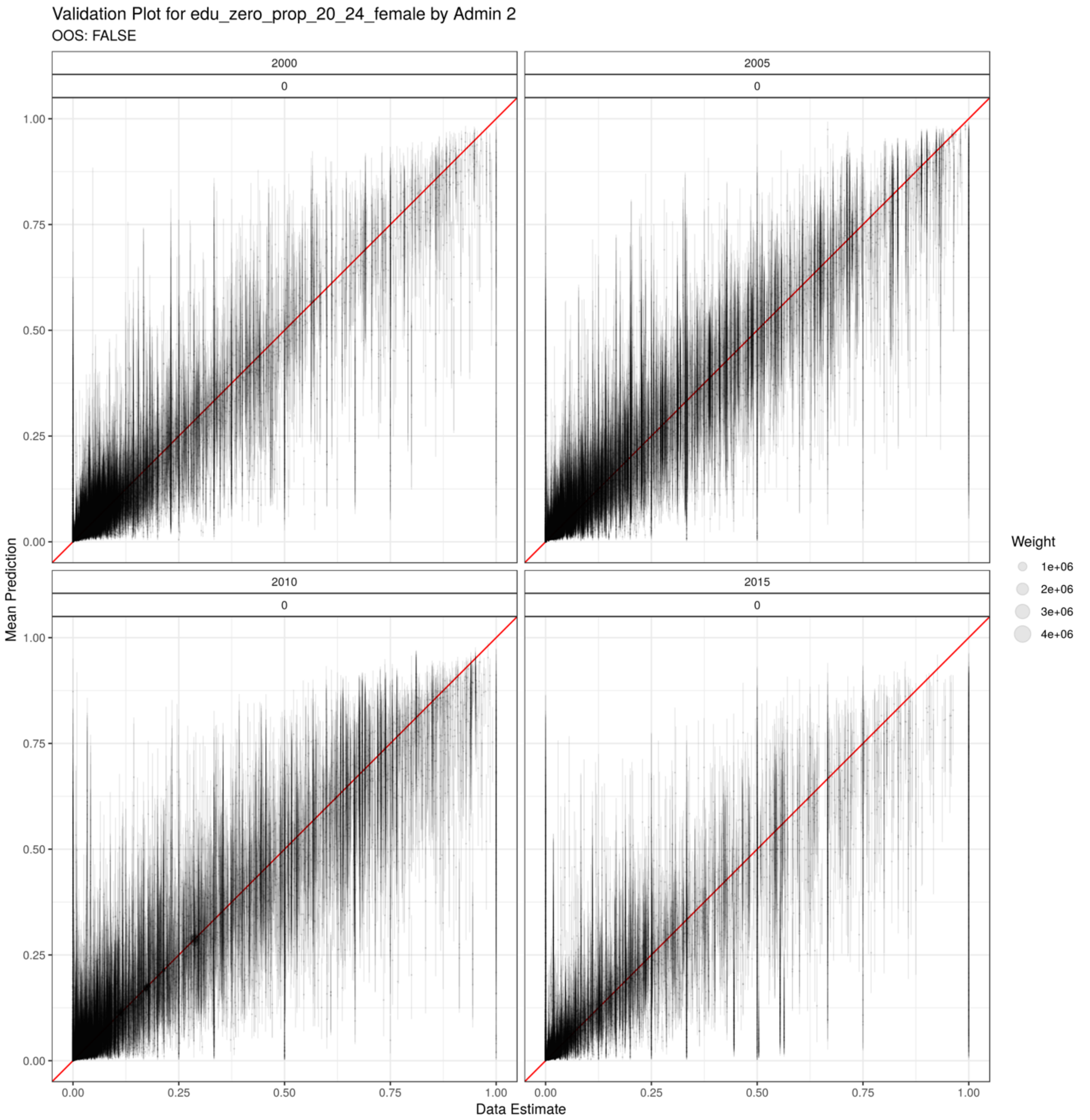

Supplementary Figure 36. Zero years education (women, ages 20–24) admin 2 aggregation
Comparison of in-sample education predictions aggregated to admin 2 with 95% uncertainty intervals plotted against admin 1 aggregated data observations.

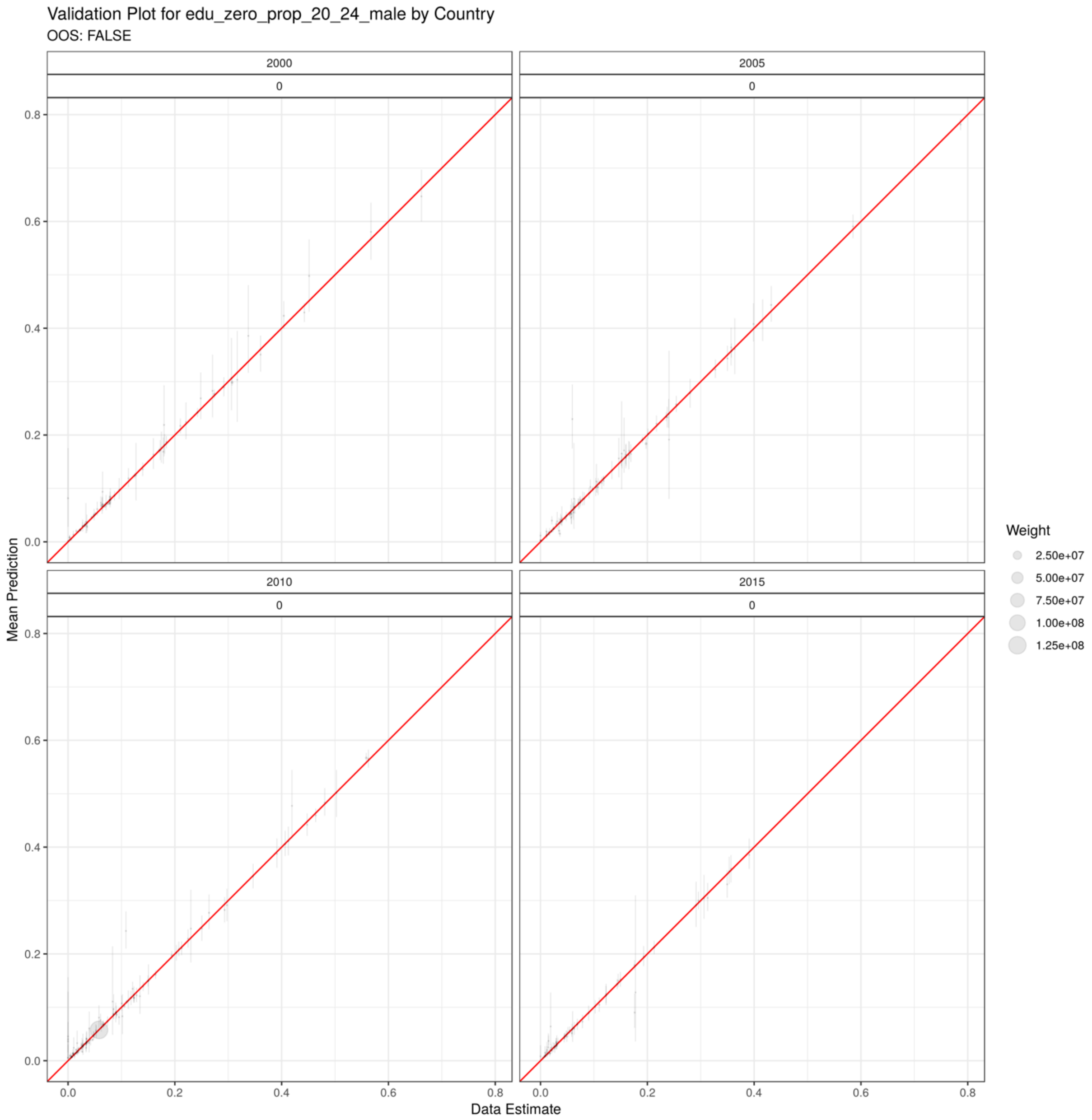

Supplementary Figure 37. Zero years education (men, ages 20–24) admin 0 aggregation
Comparison of in-sample education predictions aggregated to admin 0 with 95% uncertainty intervals plotted against admin 1 aggregated data observations.

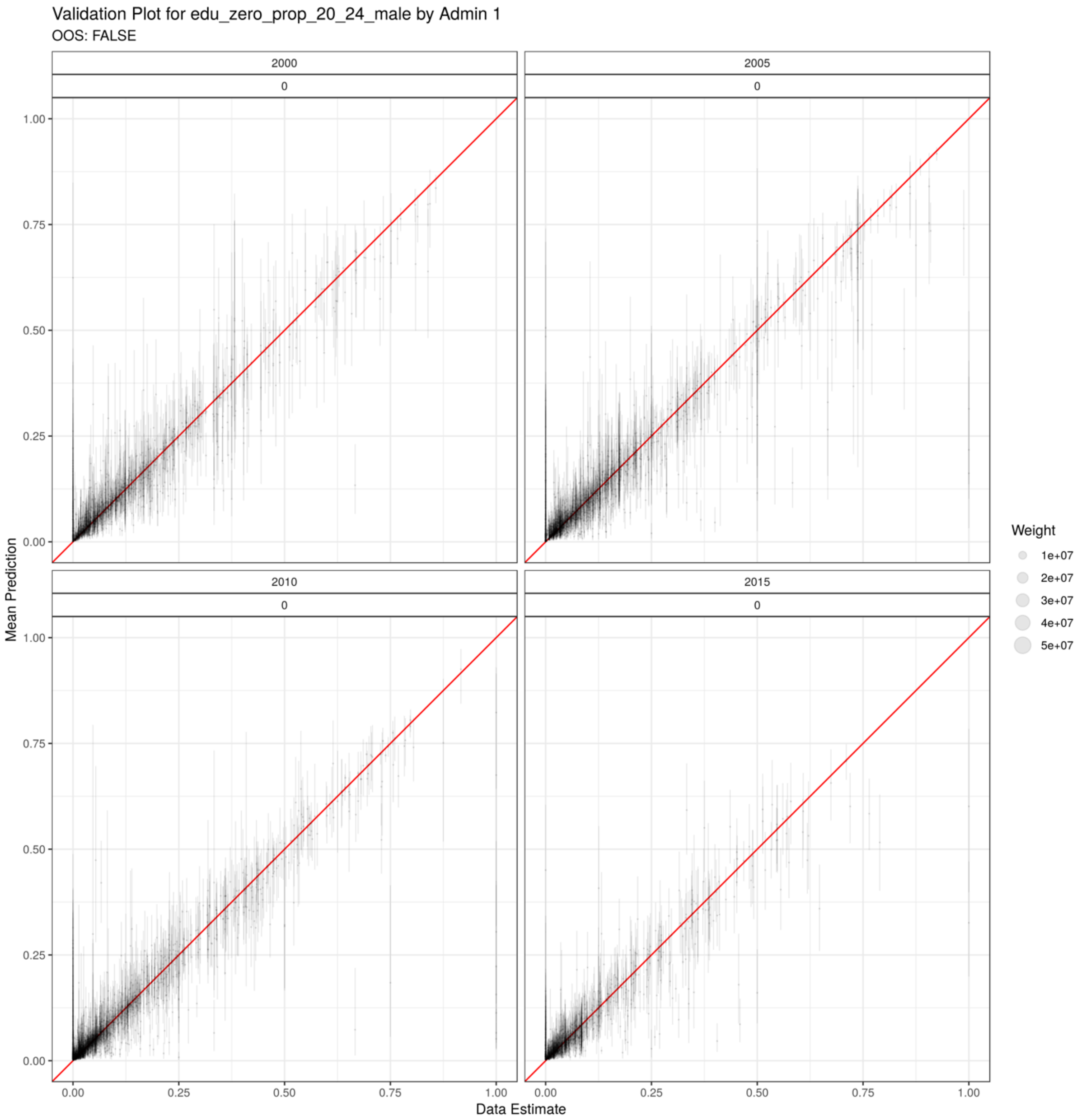

Supplementary Figure 38. Zero years education (men, ages 20–24) admin 1 aggregation
Comparison of in-sample education predictions aggregated to admin 1 with 95% uncertainty intervals plotted against admin 1 aggregated data observations.

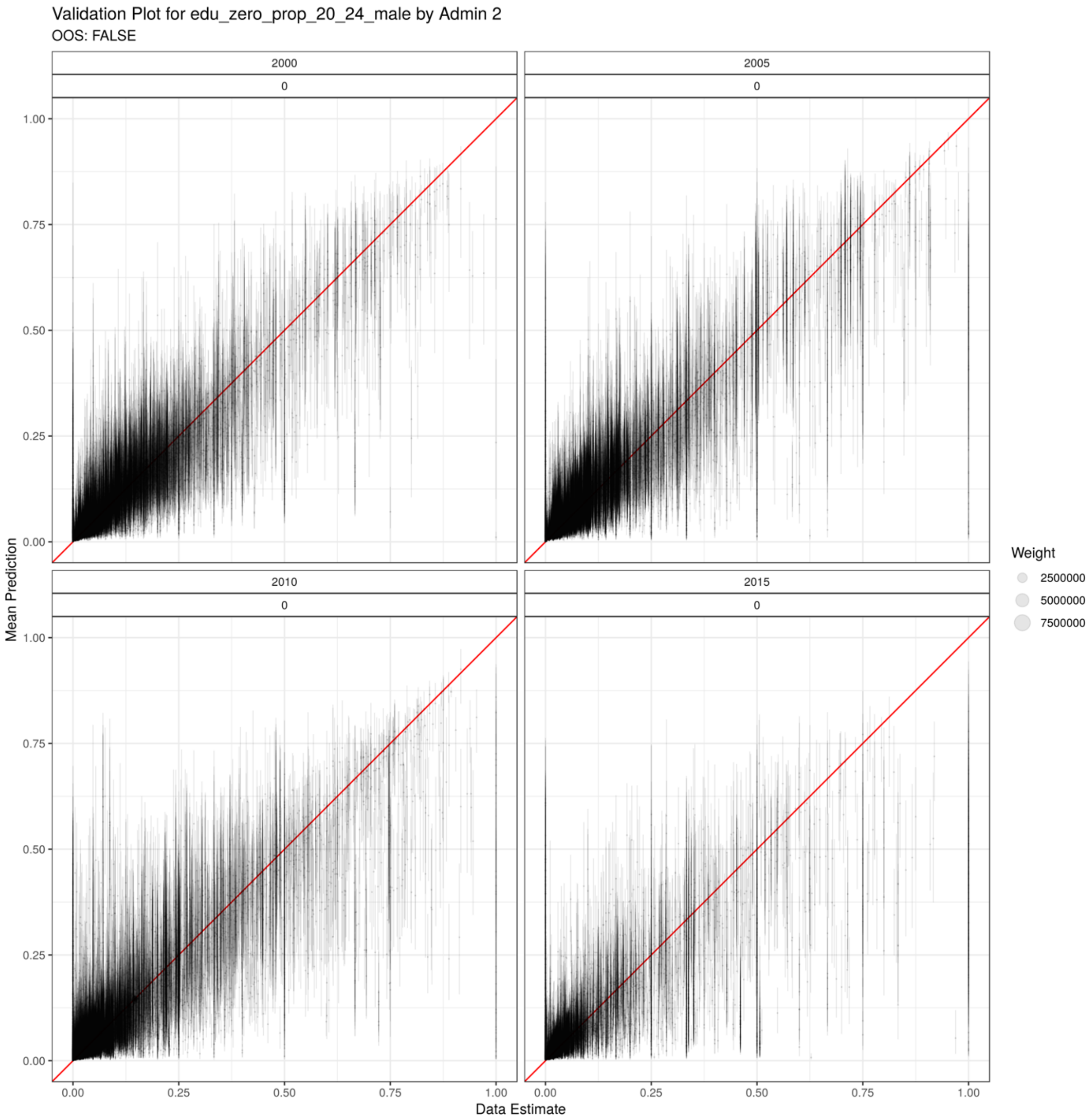

Supplementary Figure 39. Zero years education (men, ages 20–24) admin 2 aggregation

Comparison of in-sample education predictions aggregated to admin 2 with 95% uncertainty intervals plotted against admin 1 aggregated data observations.

Supplementary Table 28. In sample predictive metrics for 1–5 years of education (women, ages 15–49), aggregated to admin 0, admin 1, and admin 2

Aggregation	Year	Median SS	Mean Err	RMSE	Corr.	95% Cov.
Admin 0	2000	9603.0000	-0.0130	0.0244	0.9950	0.7946
Admin 0	2005	11733.3631	-0.0007	0.0061	0.9993	0.9291
Admin 0	2010	12925.9901	-0.0003	0.0027	0.9978	0.9842
Admin 0	2015	11955.0000	0.0037	0.0071	0.9981	0.9422
Admin 1	2000	911.0000	-0.0130	0.0277	0.9927	0.7946
Admin 1	2005	539.5000	-0.0007	0.0132	0.9969	0.9291
Admin 1	2010	821.0000	-0.0003	0.0039	0.9963	0.9842
Admin 1	2015	522.0000	0.0037	0.0127	0.9921	0.9422
Admin 2	2000	95.0000	-0.0130	0.0486	0.9643	0.7946
Admin 2	2005	26.2430	-0.0007	0.0319	0.9831	0.9291
Admin 2	2010	34.0882	-0.0003	0.0089	0.9824	0.9842
Admin 2	2015	28.0000	0.0037	0.0317	0.9594	0.9422

Supplementary Table 29. In sample predictive metrics for 1–5 years of education (men, ages 15–49), aggregated to admin 0, admin 1, and admin 2

Aggregation	Year	Median SS	Mean Err	RMSE	Corr.	95% Cov.
Admin 0	2000	10184.9599	-0.0137	0.0261	0.9963	0.7892
Admin 0	2005	10867.2664	-0.0003	0.0048	0.9996	0.9253
Admin 0	2010	14125.0000	0.0000	0.0010	0.9994	0.9896
Admin 0	2015	11661.0445	0.0035	0.0066	0.9959	0.9479
Admin 1	2000	978.0000	-0.0137	0.0303	0.9934	0.7892
Admin 1	2005	553.6495	-0.0003	0.0121	0.9974	0.9253
Admin 1	2010	870.5000	0.0000	0.0020	0.9982	0.9896
Admin 1	2015	569.0000	0.0035	0.0110	0.9898	0.9479
Admin 2	2000	97.8408	-0.0137	0.0530	0.9613	0.7892
Admin 2	2005	25.7144	-0.0003	0.0310	0.9844	0.9253
Admin 2	2010	33.8747	0.0000	0.0053	0.9869	0.9896
Admin 2	2015	27.7524	0.0035	0.0293	0.9465	0.9479

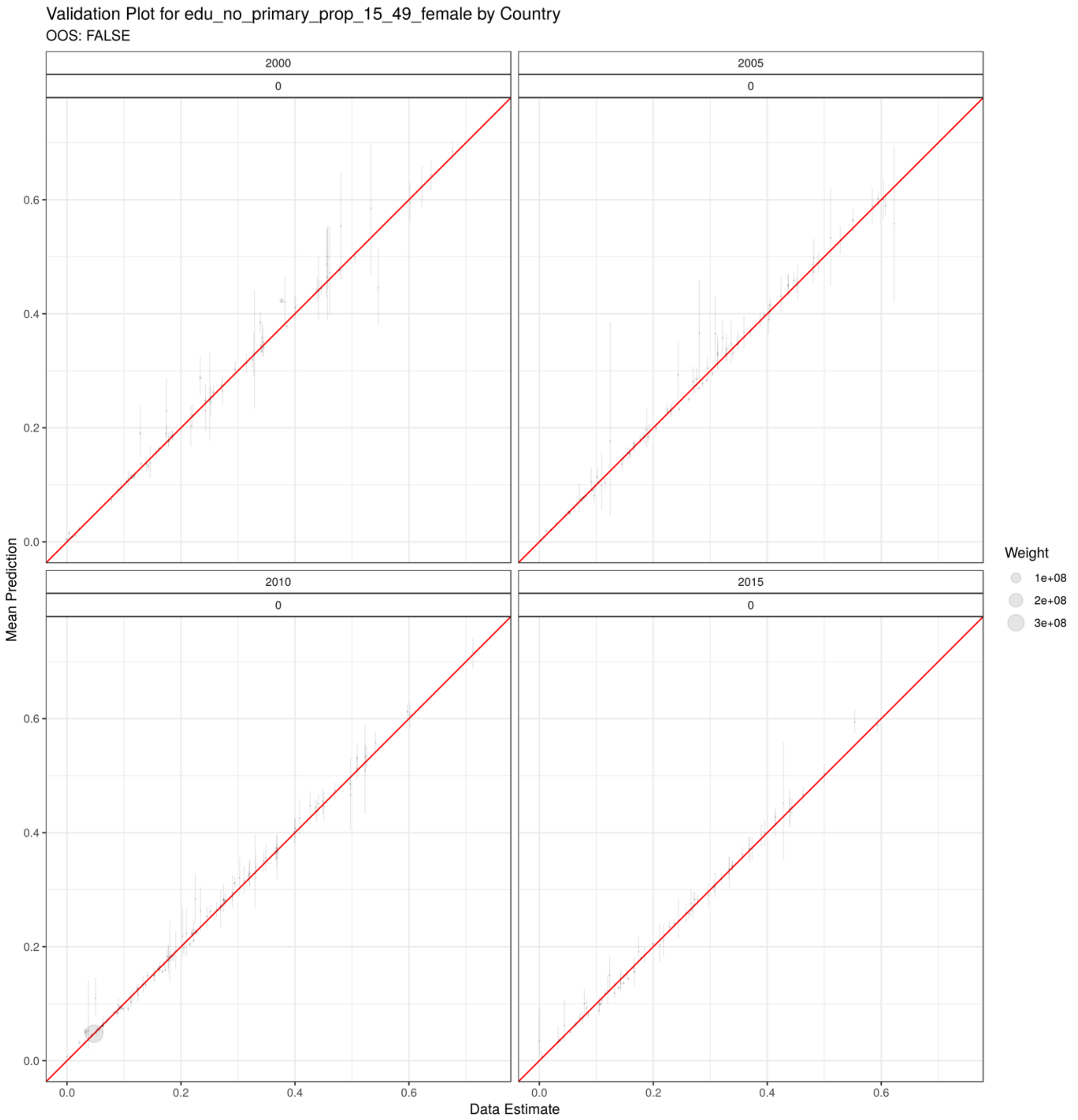

Supplementary Figure 40. 1–5 years education (women, ages 15–49) admin 0 aggregation
Comparison of in-sample education predictions aggregated to admin 0 with 95% uncertainty intervals plotted against admin 1 aggregated data observations.

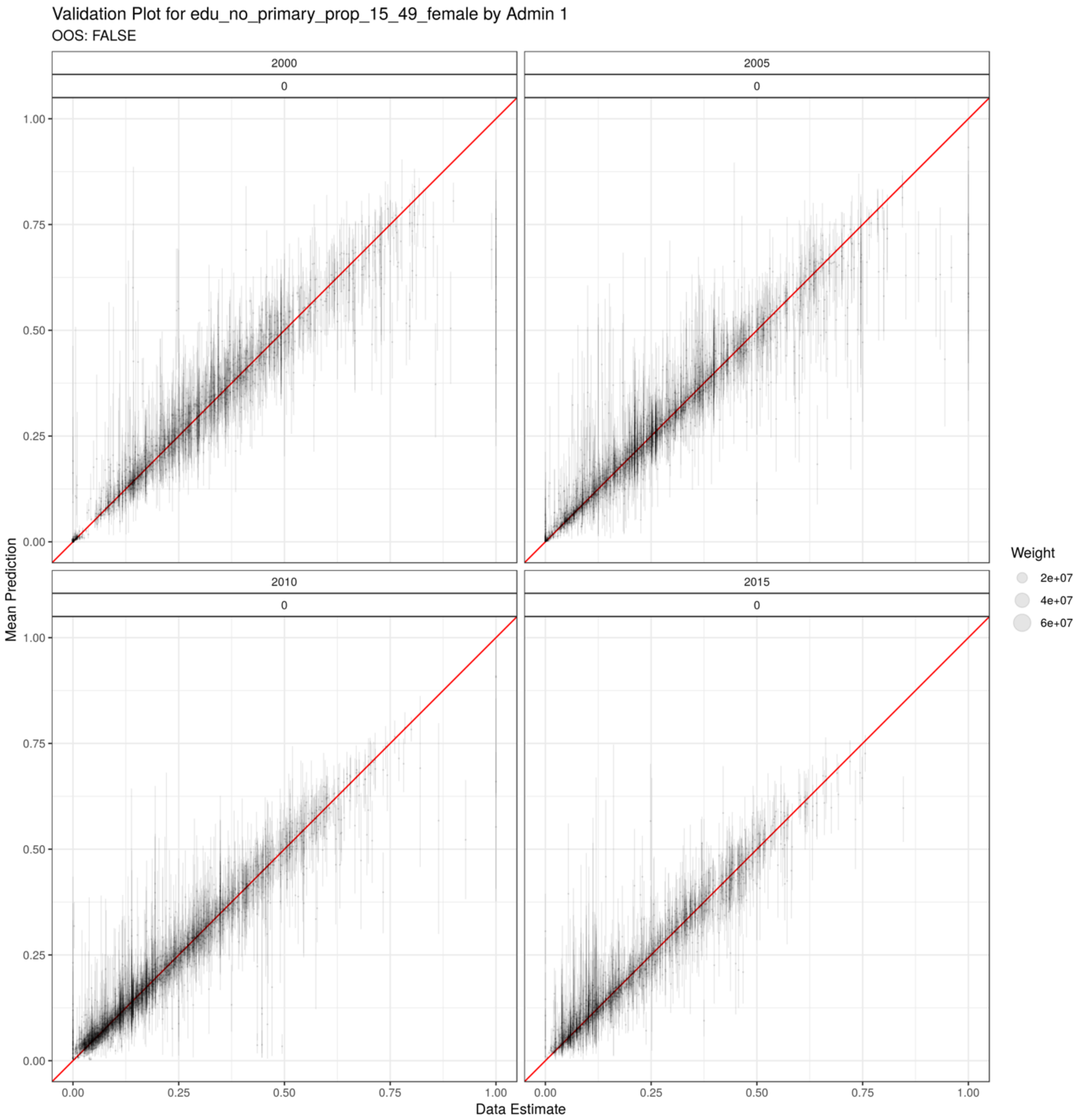

Supplementary Figure 41. 1–5 years education (women, ages 15–49) admin 1 aggregation
Comparison of in-sample education predictions aggregated to admin 1 with 95% uncertainty intervals plotted against admin 1 aggregated data observations.

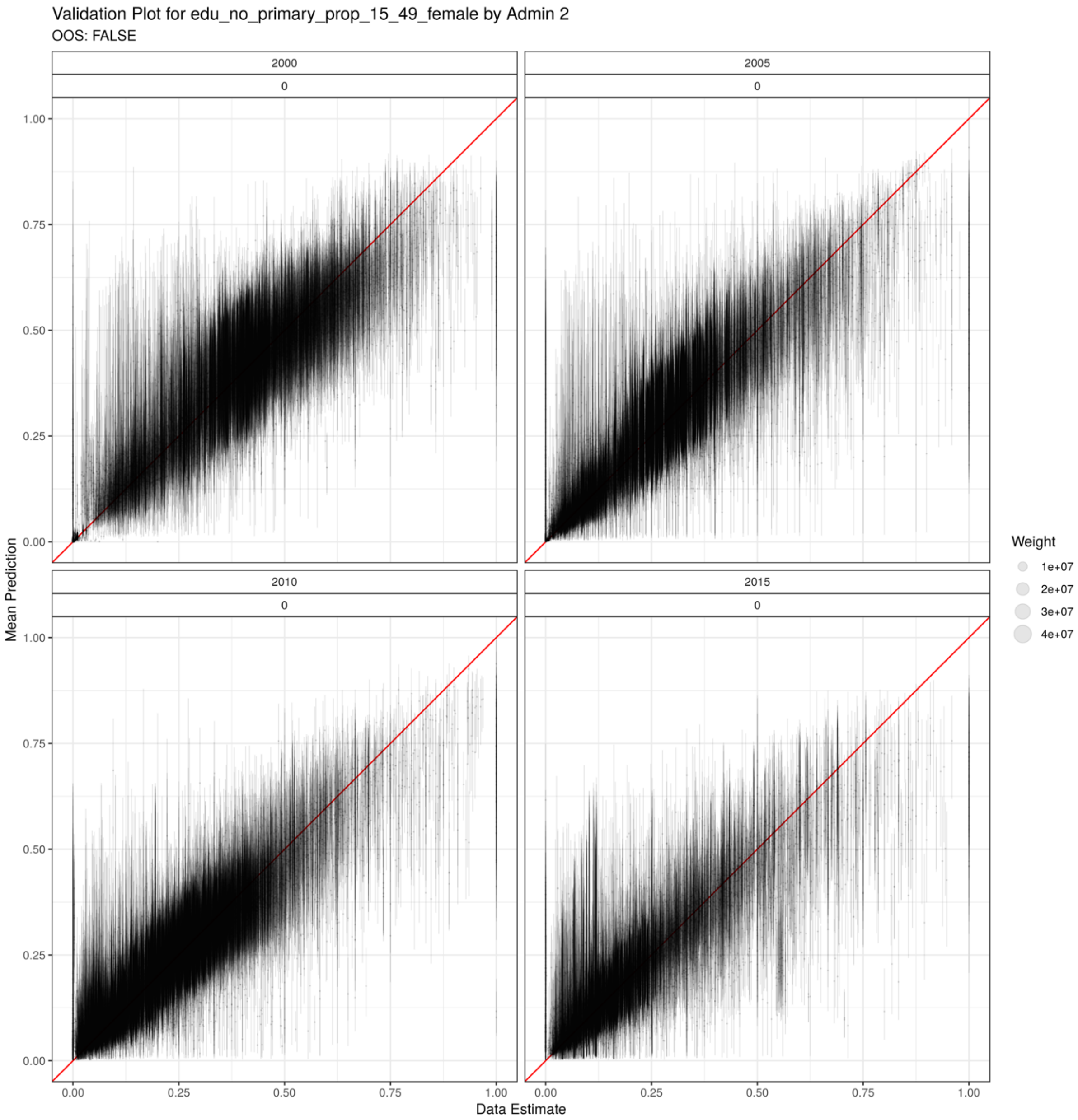

Supplementary Figure 42. 1–5 years education (women, ages 15–49) admin 2 aggregation
Comparison of in-sample education predictions aggregated to admin 2 with 95% uncertainty intervals plotted against admin 1 aggregated data observations.

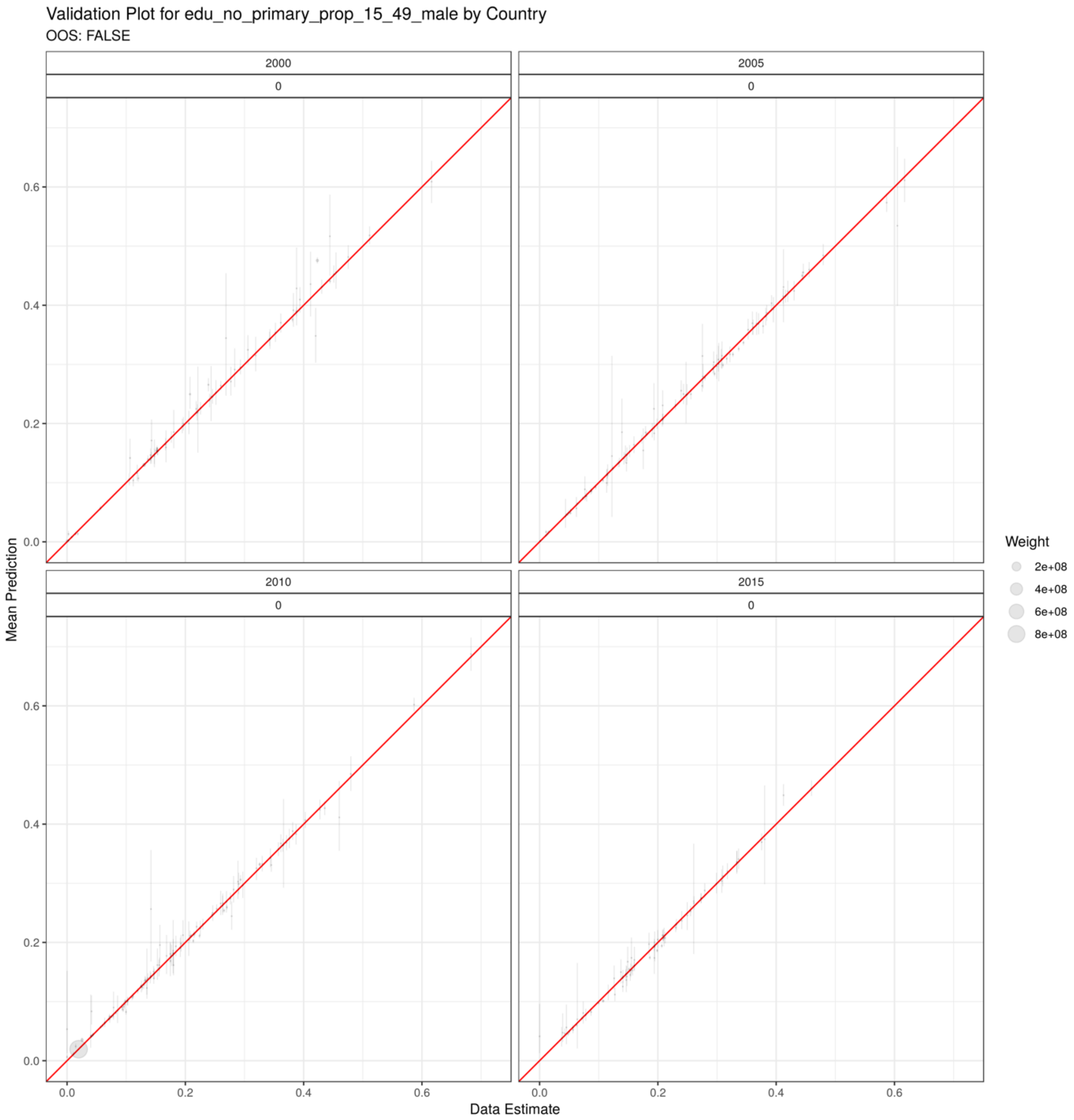

Supplementary Figure 43. 1–5 years education (men, ages 15–49) admin 0 aggregation
Comparison of in-sample education predictions aggregated to admin 0 with 95% uncertainty intervals plotted against admin 1 aggregated data observations.

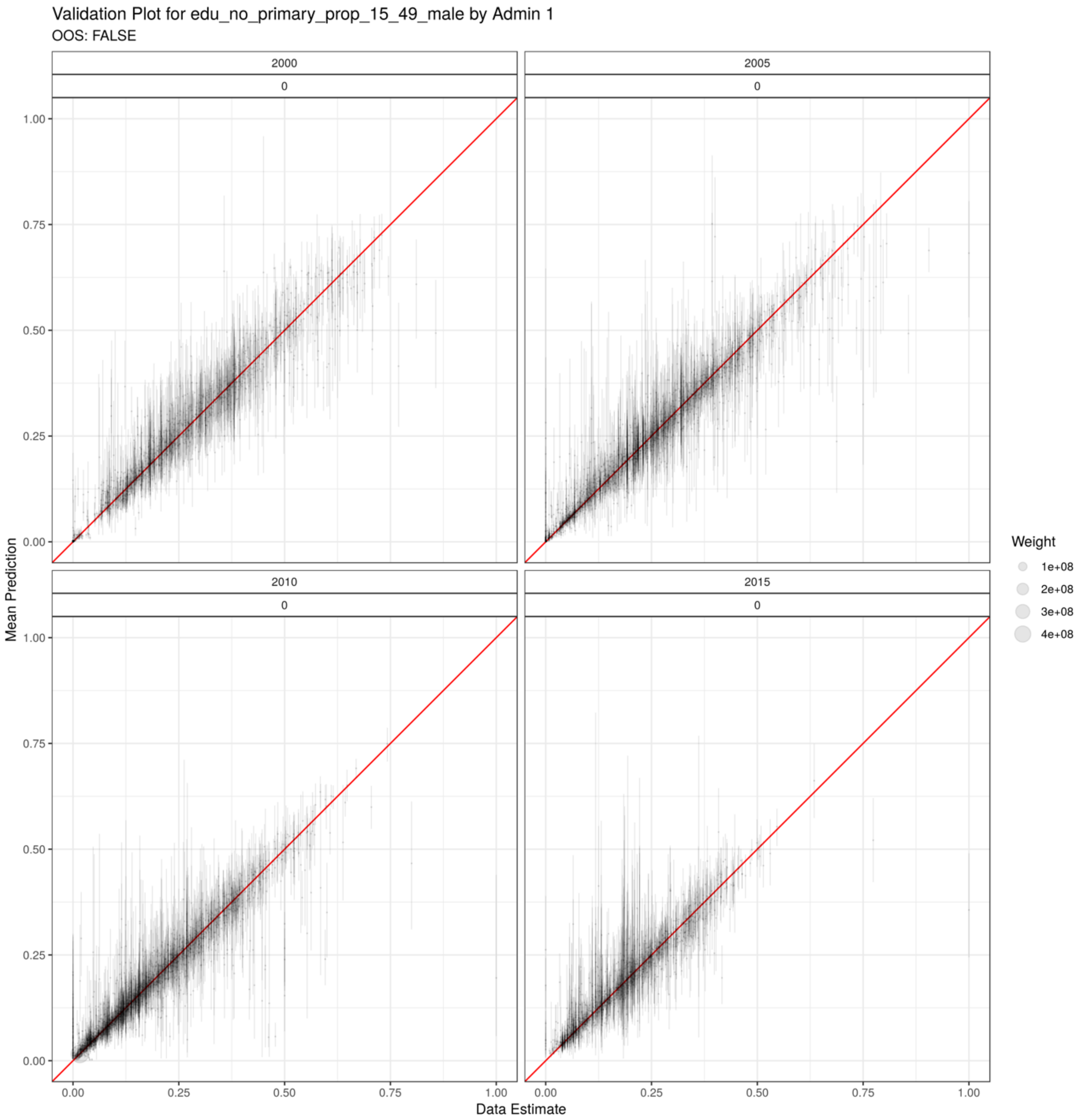

Supplementary Figure 44. 1–5 years education (men, ages 15–49) admin 1 aggregation
Comparison of in-sample education predictions aggregated to admin 1 with 95% uncertainty intervals plotted against admin 1 aggregated data observations.

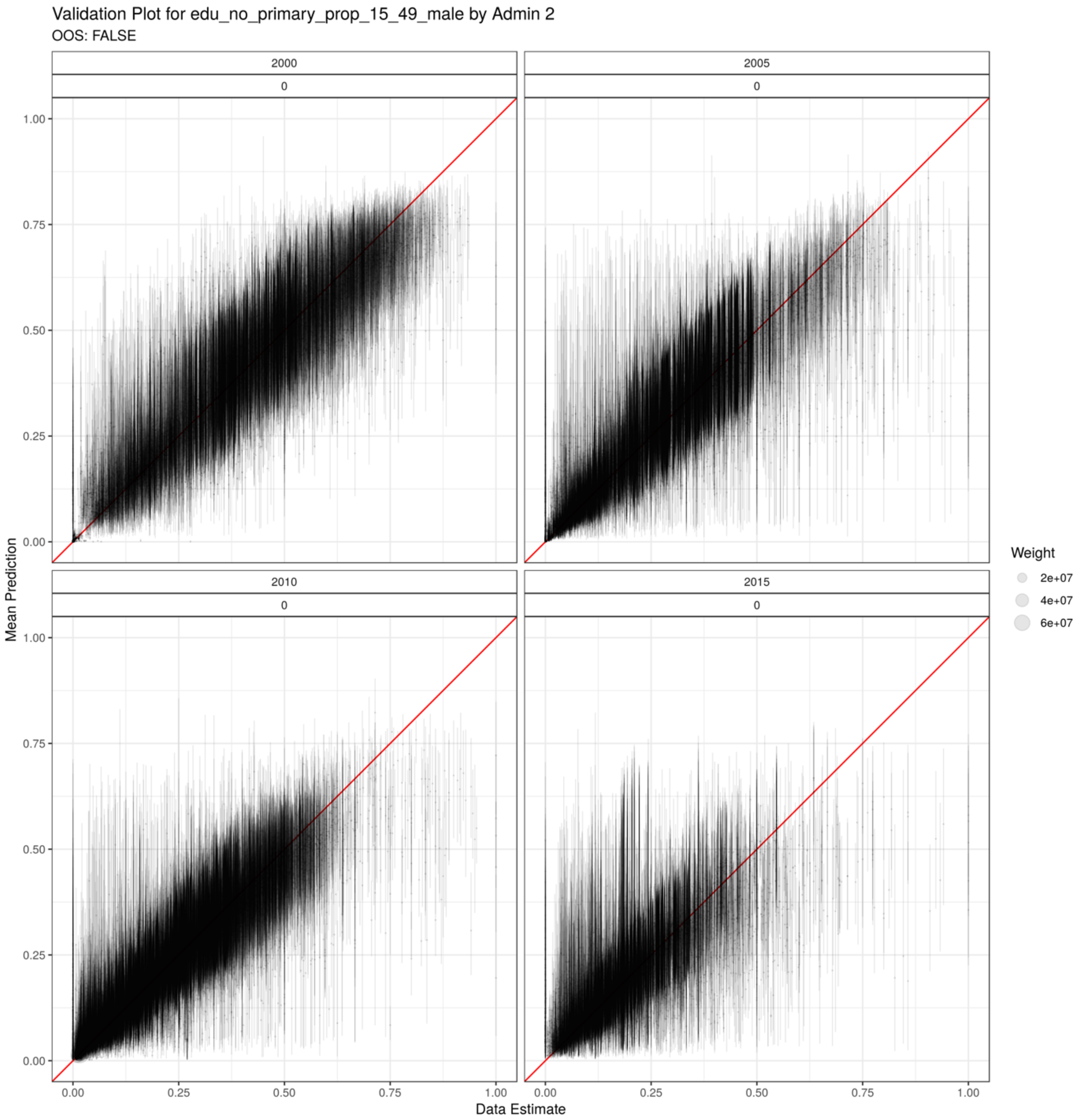

Supplementary Figure 45. 1–5 years education (men, ages 15–49) admin 2 aggregation

Comparison of in-sample education predictions aggregated to admin 2 with 95% uncertainty intervals plotted against admin 1 aggregated data observations.

Supplementary Table 30. In sample predictive metrics for 1–5 years of education (women, ages 20–24), aggregated to admin 0, admin 1, and admin 2

Aggregation	Year	Median SS	Mean Err	RMSE	Corr.	95% Cov.
Admin 0	2000	1949.0000	-0.0076	0.0144	0.9977	0.9188
Admin 0	2005	2418.0000	-0.0009	0.0045	0.9995	0.9308
Admin 0	2010	2517.5000	-0.0004	0.0009	0.9998	0.9696
Admin 0	2015	2035.5557	0.0003	0.0025	0.9996	0.9546
Admin 1	2000	175.0000	-0.0076	0.0187	0.9951	0.9188
Admin 1	2005	104.1411	-0.0009	0.0142	0.9951	0.9308
Admin 1	2010	143.0000	-0.0004	0.0041	0.9985	0.9696
Admin 1	2015	99.0000	0.0003	0.0148	0.9879	0.9546
Admin 2	2000	17.0000	-0.0076	0.0424	0.9654	0.9188
Admin 2	2005	4.5759	-0.0009	0.0359	0.9729	0.9308
Admin 2	2010	5.4401	-0.0004	0.0114	0.9885	0.9696
Admin 2	2015	4.2984	0.0003	0.0383	0.9345	0.9546

Supplementary Table 31. In sample predictive metrics for 1–5 years of education (men, ages 20–24), aggregated to admin 0, admin 1, and admin 2

Aggregation	Year	Median SS	Mean Err	RMSE	Corr.	95% Cov.
Admin 0	2000	1899.0000	-0.0092	0.0182	0.9981	0.9087
Admin 0	2005	1911.0000	-0.0006	0.0041	0.9995	0.9301
Admin 0	2010	2453.0000	0.0009	0.0010	0.9999	0.8542
Admin 0	2015	2151.7327	0.0007	0.0028	0.9989	0.9501
Admin 1	2000	164.5000	-0.0092	0.0227	0.9953	0.9087
Admin 1	2005	100.8876	-0.0006	0.0143	0.9945	0.9301
Admin 1	2010	153.0000	0.0009	0.0025	0.9973	0.8542
Admin 1	2015	100.5000	0.0007	0.0131	0.9819	0.9501
Admin 2	2000	17.0000	-0.0092	0.0486	0.9605	0.9087
Admin 2	2005	4.4767	-0.0006	0.0353	0.9712	0.9301
Admin 2	2010	5.4210	0.0009	0.0060	0.9814	0.8542
Admin 2	2015	4.0400	0.0007	0.0366	0.8956	0.9501

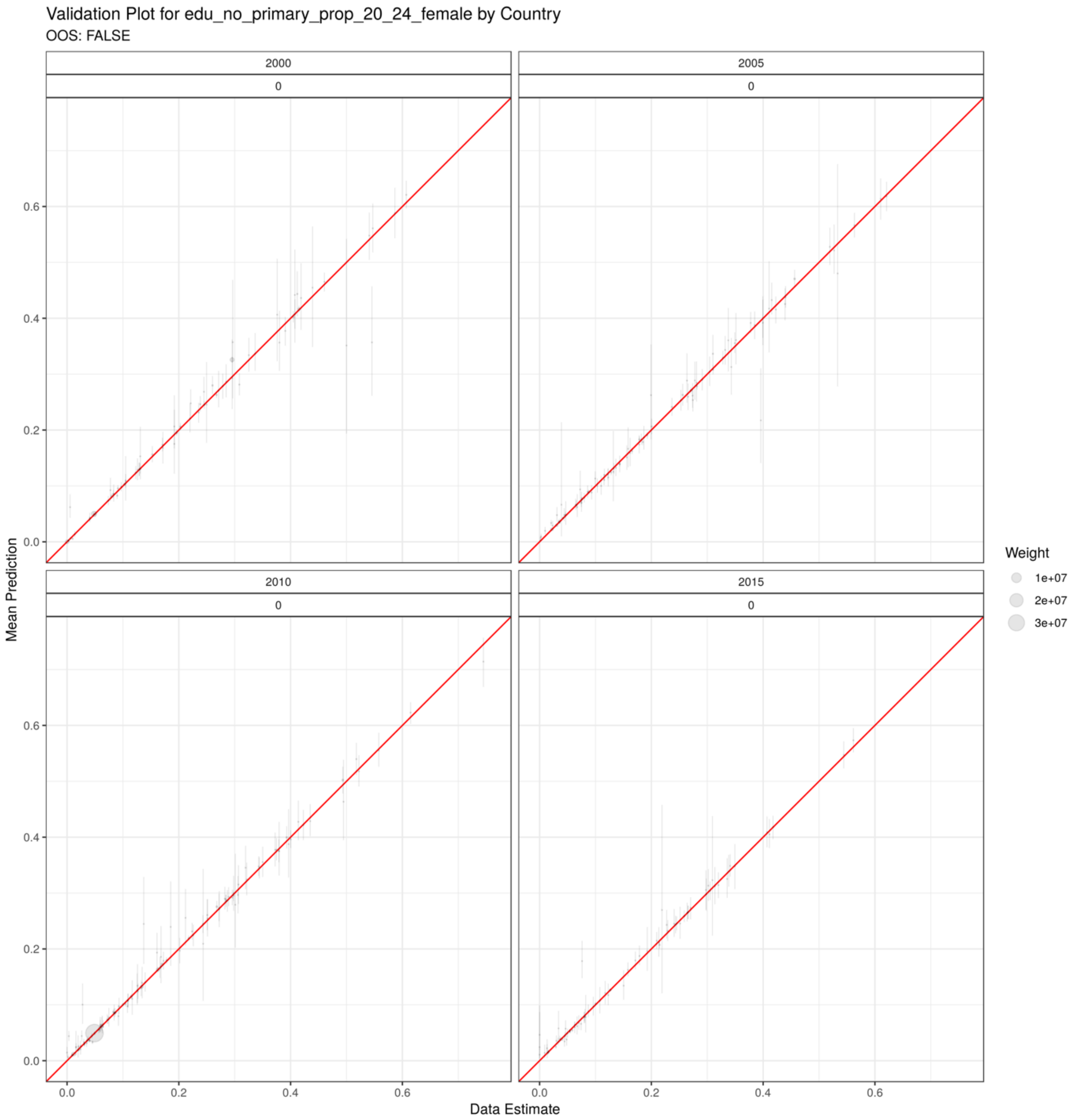

Supplementary Figure 46. 1–5 years education (women, ages 20–24) admin 0 aggregation

Comparison of in-sample education predictions aggregated to admin 0 with 95% uncertainty intervals plotted against admin 1 aggregated data observations.

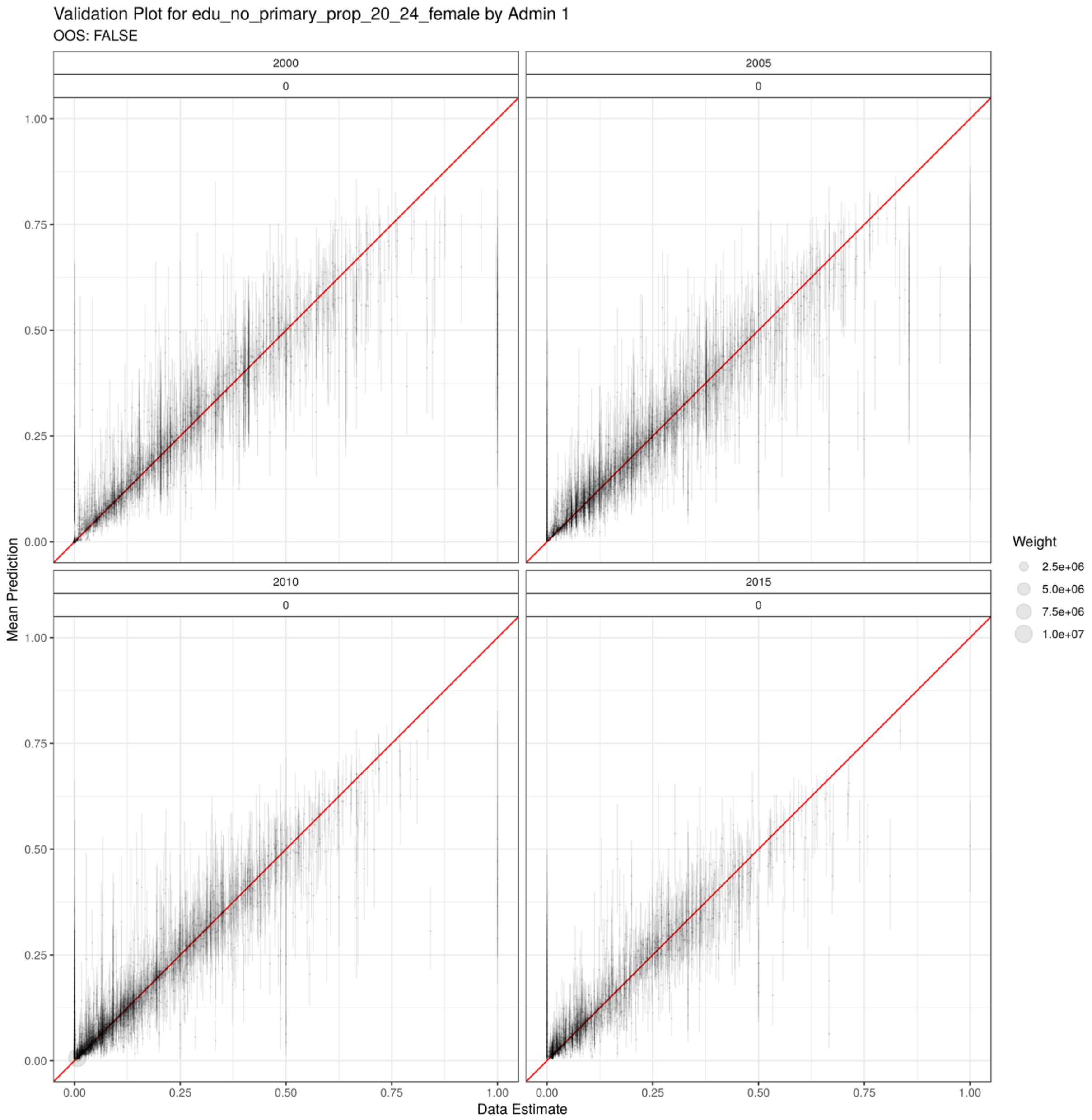

Supplementary Figure 47. 1–5 years education (women, ages 20–24) admin 1 aggregation
Comparison of in-sample education predictions aggregated to admin 1 with 95% uncertainty intervals plotted against admin 1 aggregated data observations.

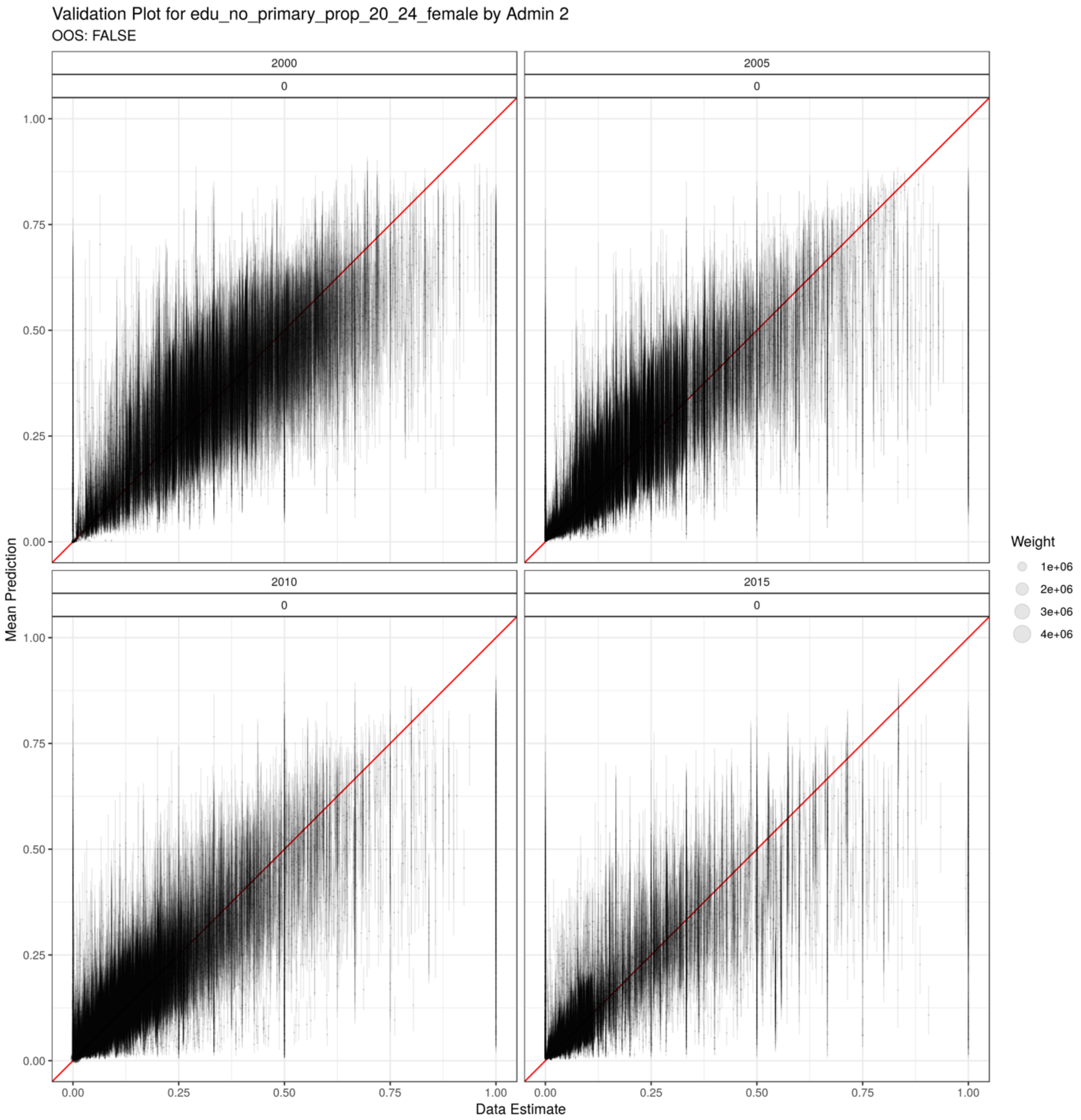

Supplementary Figure 48. 1–5 years education (women, ages 20–24) admin 2 aggregation

Comparison of in-sample education predictions aggregated to admin 2 with 95% uncertainty intervals plotted against admin 1 aggregated data observations.

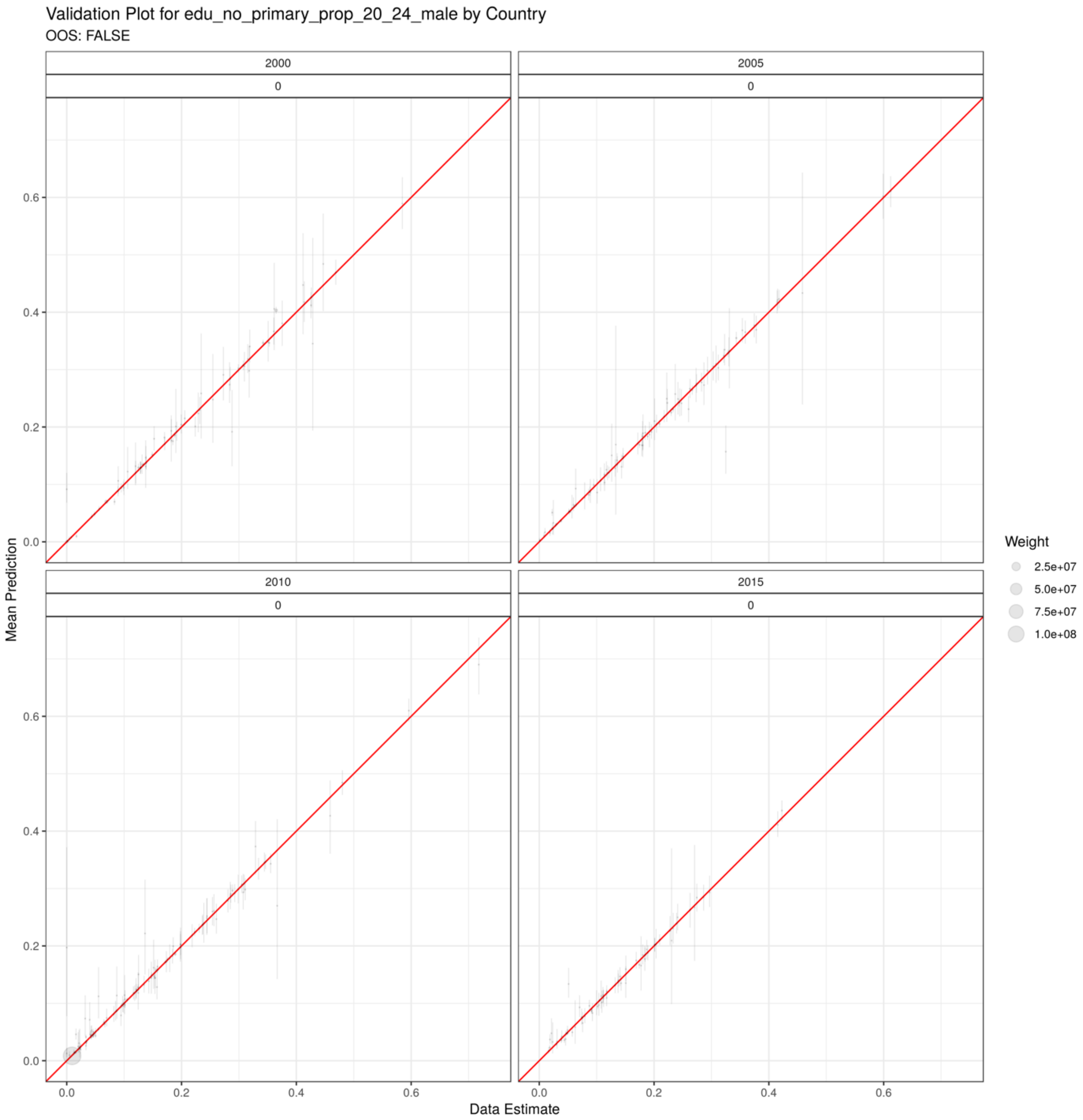

Supplementary Figure 49. 1–5 years education (men, ages 20–24) admin 0 aggregation
Comparison of in-sample education predictions aggregated to admin 0 with 95% uncertainty intervals plotted against admin 1 aggregated data observations.

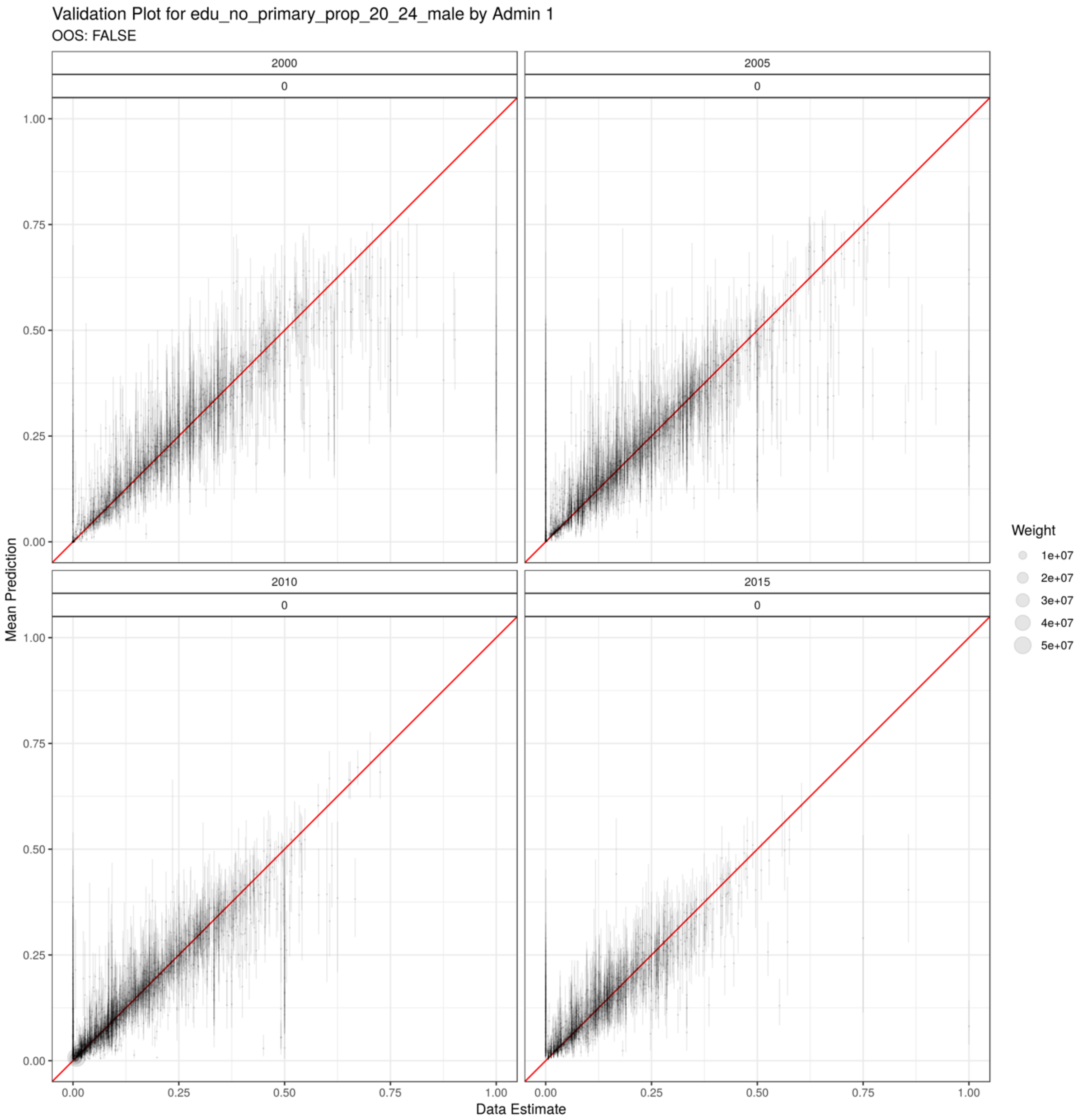

Supplementary Figure 50. 1–5 years education (men, ages 20–24) admin 1 aggregation
Comparison of in-sample education predictions aggregated to admin 1 with 95% uncertainty intervals plotted against admin 1 aggregated data observations.

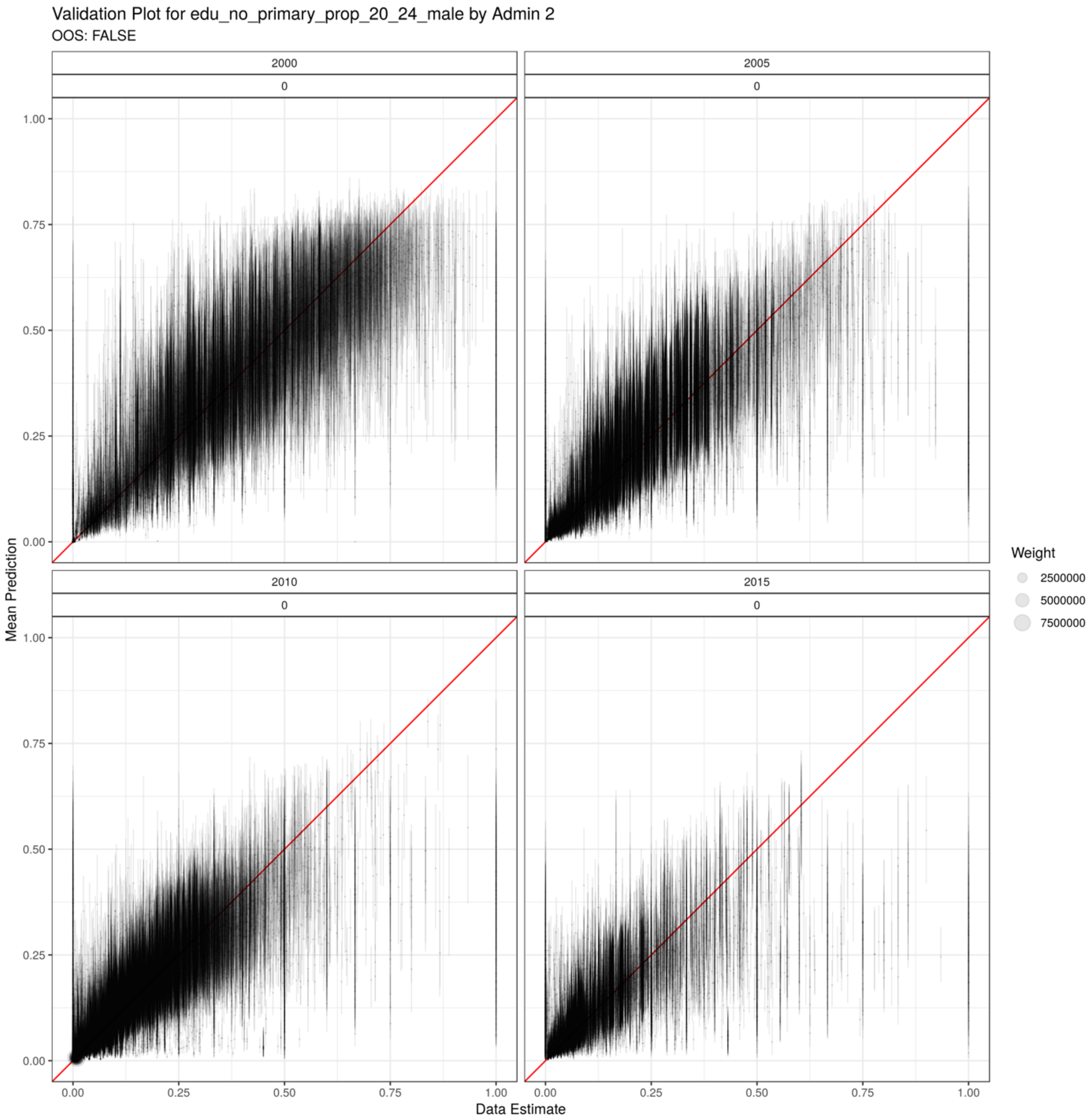

Supplementary Figure 51. 1–5 years education (men, ages 20–24) admin 2 aggregation
Comparison of in-sample education predictions aggregated to admin 2 with 95% uncertainty intervals plotted against admin 1 aggregated data observations.

Supplementary Table 32. In sample predictive metrics for 6–11 years of education (women, ages 15–49), aggregated to admin 0, admin 1, and admin 2

Aggregation	Year	Median SS	Mean Err	RMSE	Corr.	95% Cov.
Admin 0	2000	8013.0000	-0.0028	0.0246	0.9766	0.7954
Admin 0	2005	8303.5776	-0.0159	0.0229	0.9946	0.9227
Admin 0	2010	9281.6587	0.0029	0.0038	0.9986	0.9863
Admin 0	2015	9057.9153	-0.0074	0.0091	0.9988	0.9494
Admin 1	2000	689.4149	-0.0028	0.0317	0.9679	0.7954
Admin 1	2005	386.0000	-0.0159	0.0309	0.9853	0.9227
Admin 1	2010	612.0000	0.0029	0.0066	0.9927	0.9863
Admin 1	2015	385.0104	-0.0074	0.0157	0.9941	0.9494
Admin 2	2000	60.1334	-0.0028	0.0465	0.9405	0.7954
Admin 2	2005	19.3895	-0.0159	0.0569	0.9401	0.9227
Admin 2	2010	28.0000	0.0029	0.0126	0.9722	0.9863
Admin 2	2015	22.9372	-0.0074	0.0495	0.9487	0.9494

Supplementary Table 33. In sample predictive metrics for 6–11 years of education (men, ages 15–49), aggregated to admin 0, admin 1, and admin 2

Aggregation	Year	Median SS	Mean Err	RMSE	Corr.	95% Cov.
Admin 0	2000	7186.2870	-0.0564	0.0738	0.8947	0.5447
Admin 0	2005	7755.3660	-0.0758	0.0929	0.9238	0.5910
Admin 0	2010	10720.3243	0.0908	0.0948	-0.1162	0.6381
Admin 0	2015	9095.5051	-0.0616	0.0826	0.8499	0.6543
Admin 1	2000	669.0000	-0.0564	0.0948	0.7626	0.5447
Admin 1	2005	347.4343	-0.0758	0.1177	0.8029	0.5910
Admin 1	2010	622.0000	0.0908	0.1083	-0.1640	0.6381
Admin 1	2015	477.9025	-0.0616	0.1106	0.6712	0.6543
Admin 2	2000	56.0000	-0.0564	0.1179	0.6403	0.5447
Admin 2	2005	18.1650	-0.0758	0.1463	0.6816	0.5910
Admin 2	2010	28.3070	0.0908	0.1337	-0.0772	0.6381
Admin 2	2015	24.0000	-0.0616	0.1493	0.4812	0.6543

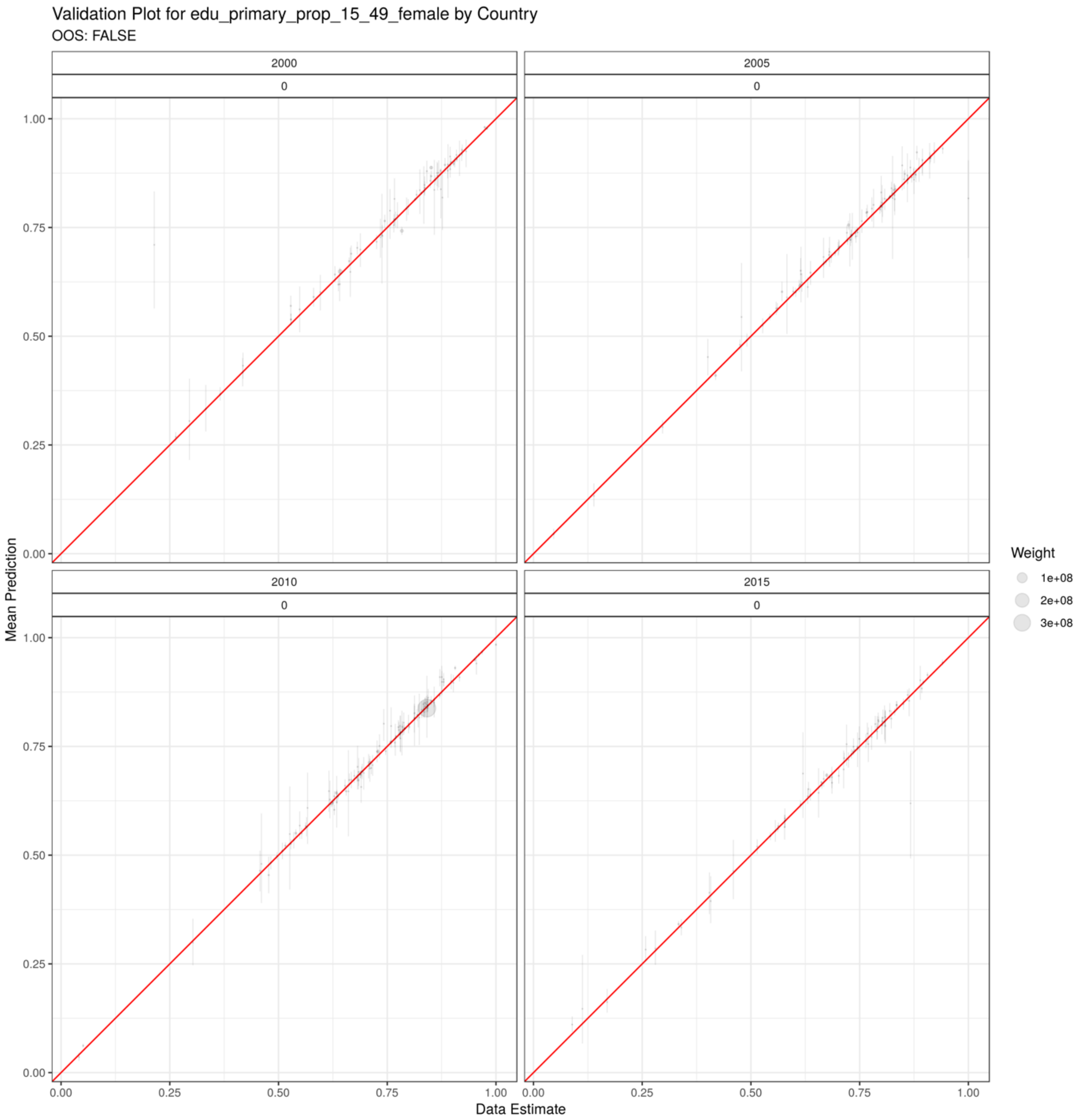

Supplementary Figure 52. 6–11 years education (women, ages 15–49) admin 0 aggregation
Comparison of in-sample education predictions aggregated to admin 0 with 95% uncertainty intervals plotted against admin 1 aggregated data observations.

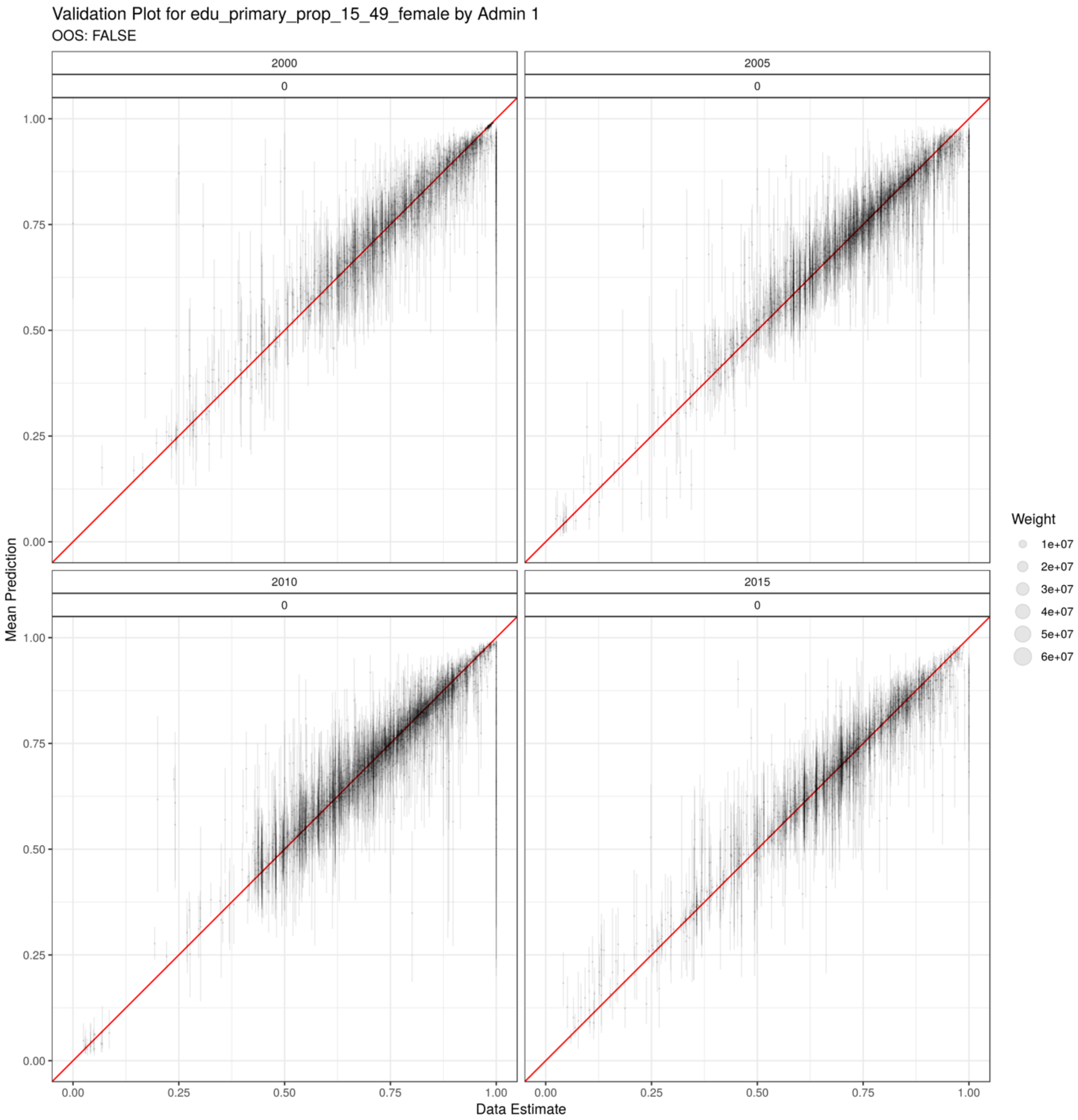

Supplementary Figure 53. 6–11 years education (women, ages 15–49) admin 1 aggregation
Comparison of in-sample education predictions aggregated to admin 1 with 95% uncertainty intervals plotted against admin 1 aggregated data observations.

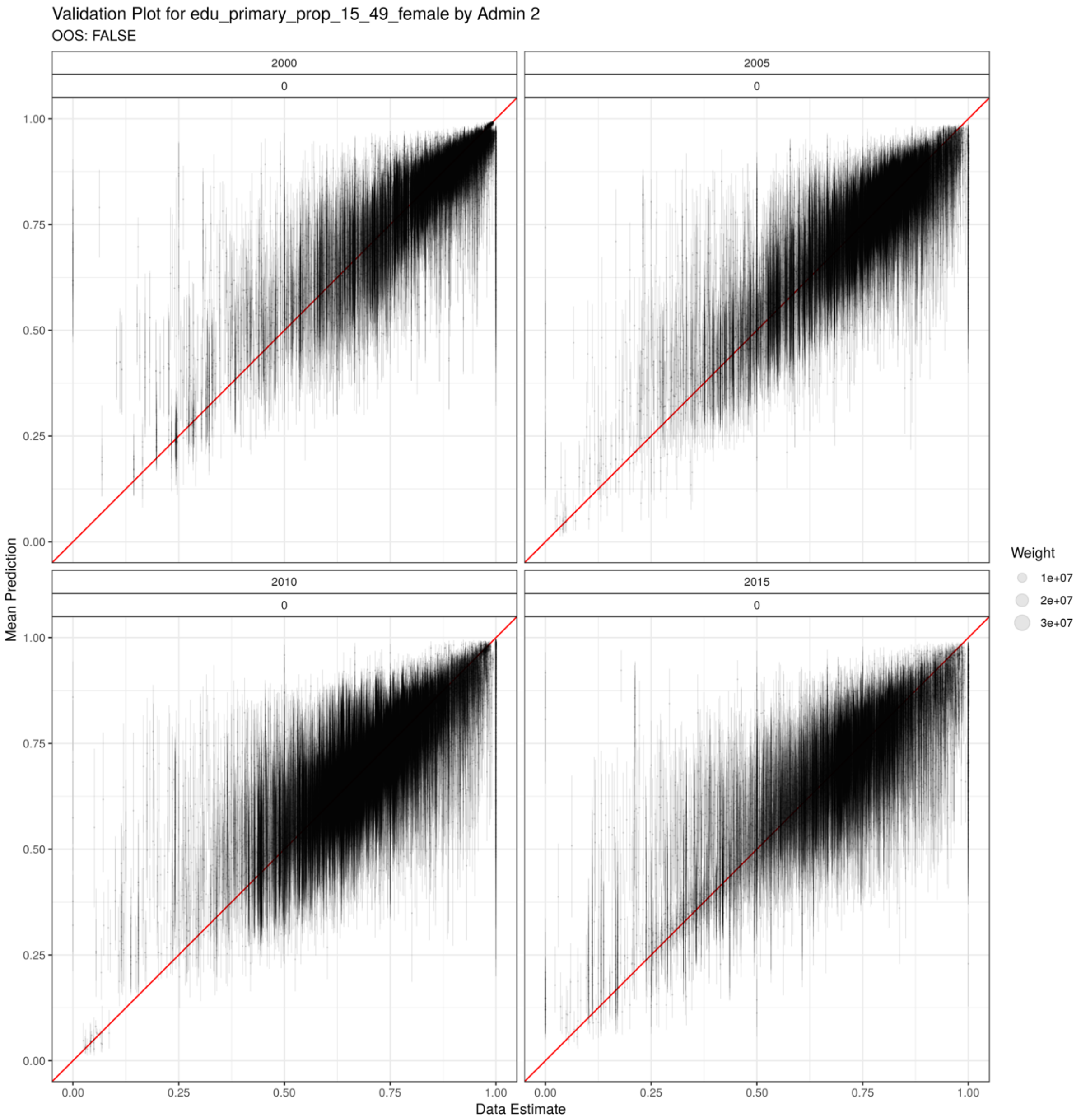

Supplementary Figure 54. 6–11 years education (women, ages 15–49) admin 2 aggregation
Comparison of in-sample education predictions aggregated to admin 2 with 95% uncertainty intervals plotted against admin 1 aggregated data observations.

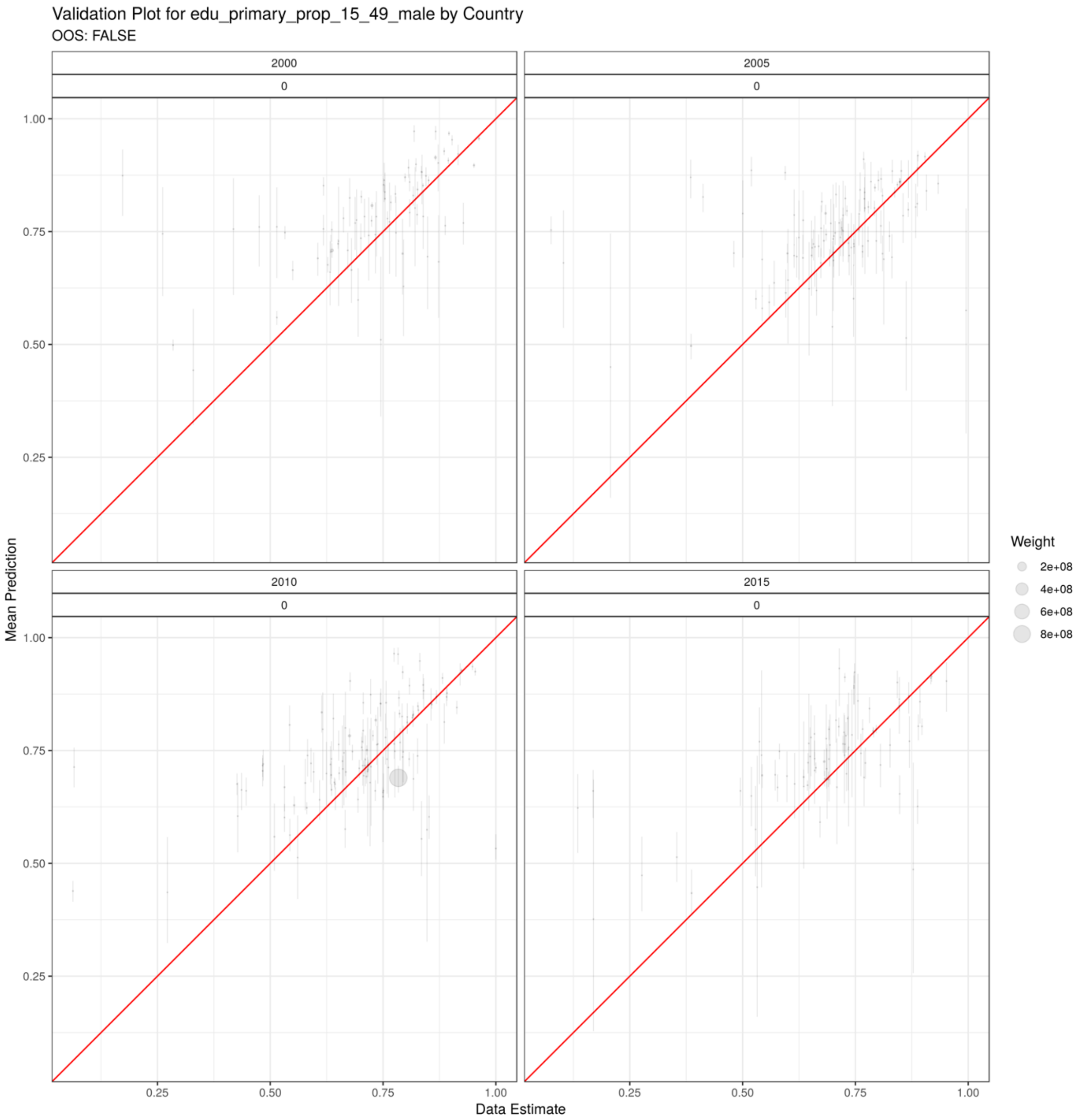

Supplementary Figure 55. 6–11 years education (men, ages 15–49) admin 2 aggregation

Comparison of in-sample education predictions aggregated to admin 2 with 95% uncertainty intervals plotted against admin 1 aggregated data observations.

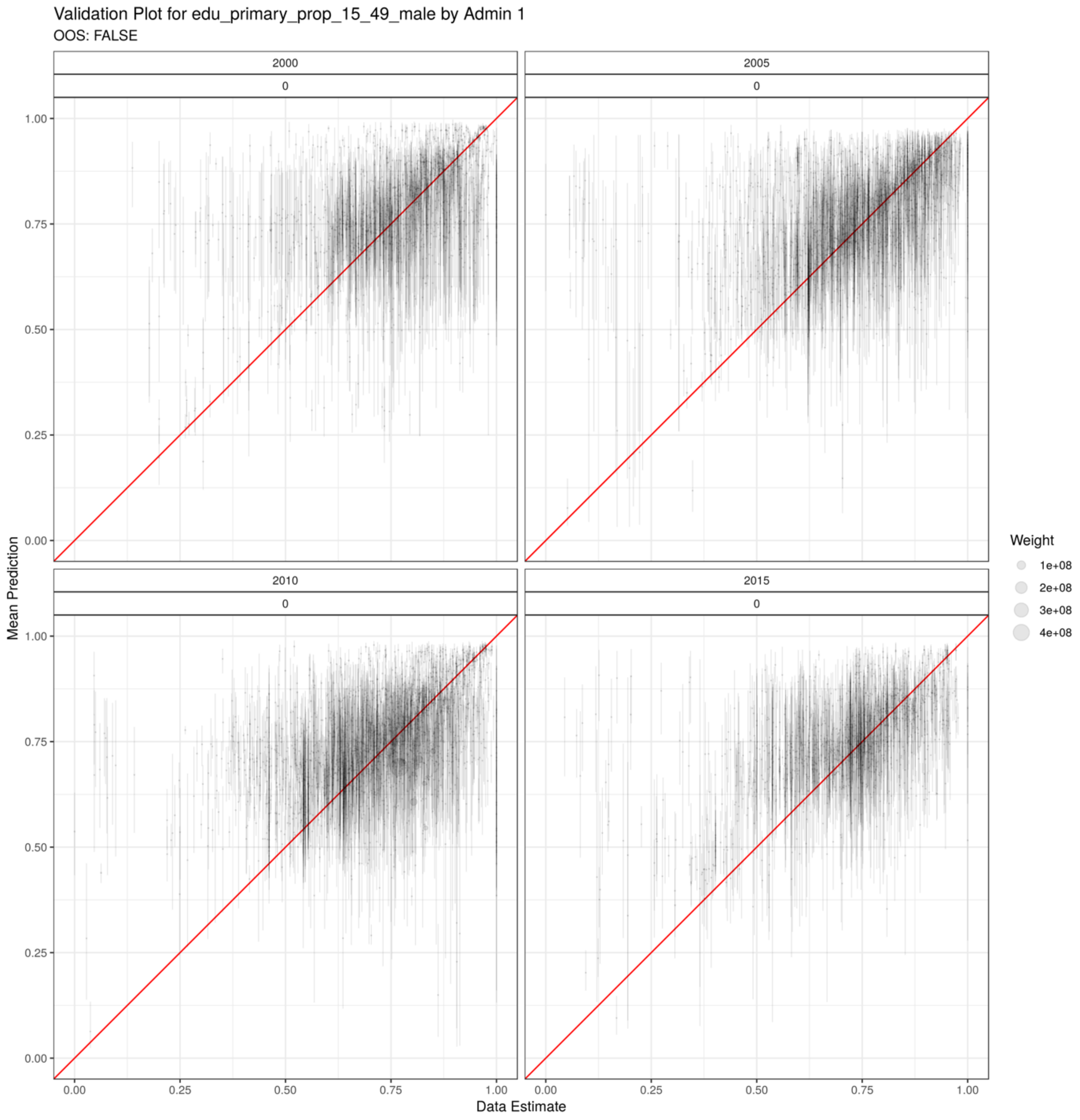

Supplementary Figure 56. 6–11 years education (men, ages 15–49) admin 2 aggregation
Comparison of in-sample education predictions aggregated to admin 2 with 95% uncertainty intervals plotted against admin 1 aggregated data observations.

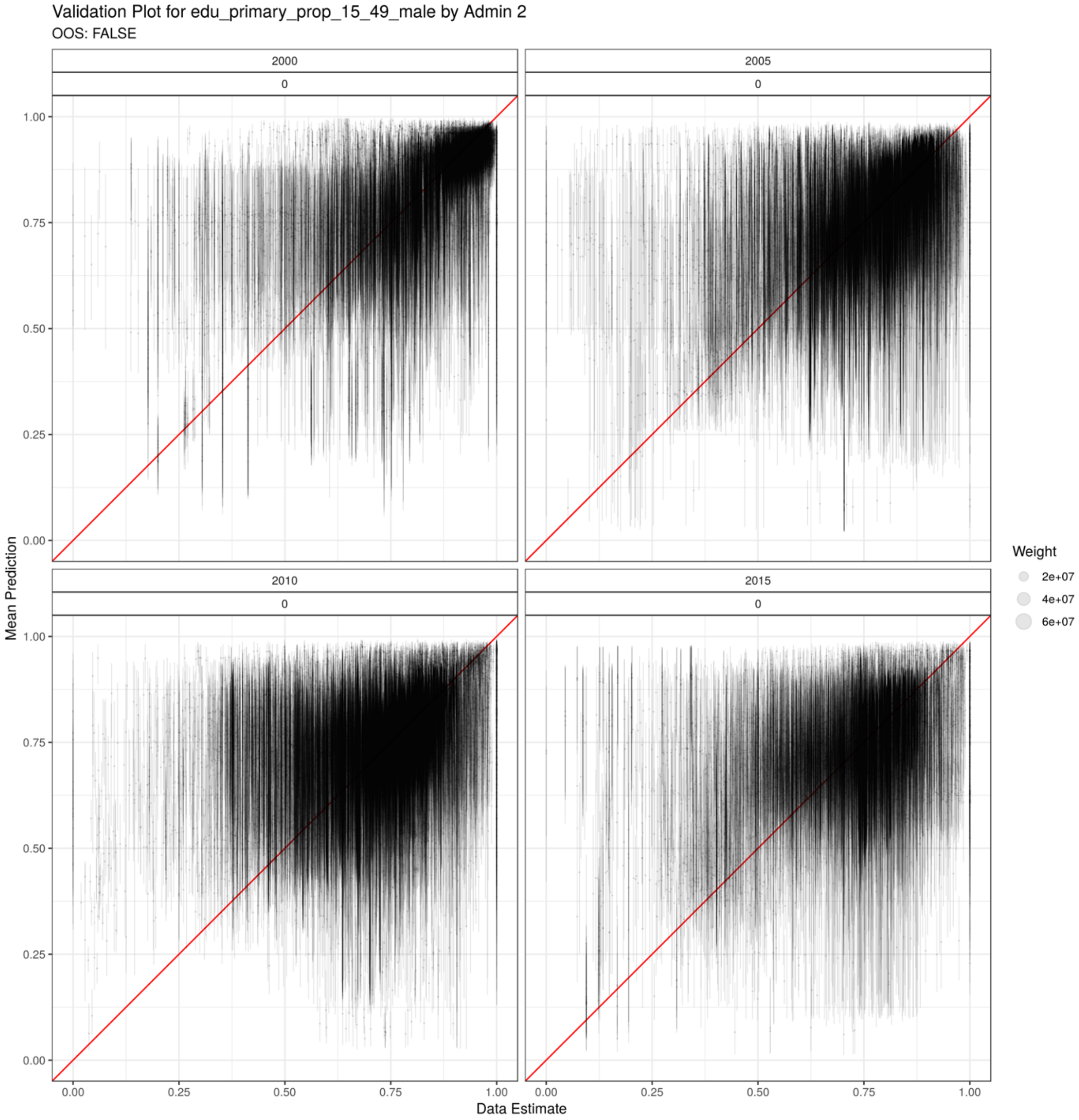

Supplementary Figure 57. 6–11 years education (men, ages 15–49) admin 2 aggregation
Comparison of in-sample education predictions aggregated to admin 2 with 95% uncertainty intervals plotted against admin 1 aggregated data observations.

Supplementary Table 34. In sample predictive metrics for 6–11 years of education (women, ages 20–24), aggregated to admin 0, admin 1, and admin 2

Aggregation	Year	Median SS	Mean Err	RMSE	Corr.	95% Cov.
Admin 0	2000	1693.0779	-0.0047	0.0136	0.9971	0.8695
Admin 0	2005	1661.0000	-0.0103	0.0163	0.9949	0.9396
Admin 0	2010	1981.4157	0.0012	0.0030	0.9995	0.9536
Admin 0	2015	1642.0670	-0.0042	0.0079	0.9984	0.9497
Admin 1	2000	147.0000	-0.0047	0.0198	0.9939	0.8695
Admin 1	2005	79.0000	-0.0103	0.0285	0.9814	0.9396
Admin 1	2010	115.5750	0.0012	0.0077	0.9974	0.9536
Admin 1	2015	75.0342	-0.0042	0.0195	0.9913	0.9497
Admin 2	2000	12.1809	-0.0047	0.0423	0.9738	0.8695
Admin 2	2005	3.8181	-0.0103	0.0614	0.9279	0.9396
Admin 2	2010	4.9258	0.0012	0.0186	0.9852	0.9536
Admin 2	2015	4.0000	-0.0042	0.0639	0.9352	0.9497

Supplementary Table 35. In sample predictive metrics for 6–11 years of education (men, ages 20–24), aggregated to admin 0, admin 1, and admin 2

Aggregation	Year	Median SS	Mean Err	RMSE	Corr.	95% Cov.
Admin 0	2000	1481.0256	-0.0038	0.0122	0.9978	0.8853
Admin 0	2005	1620.1010	-0.0100	0.0157	0.9953	0.9354
Admin 0	2010	1764.0000	0.0000	0.0011	0.9992	0.9778
Admin 0	2015	1875.0000	-0.0038	0.0067	0.9988	0.9475
Admin 1	2000	140.3899	-0.0038	0.0183	0.9943	0.8853
Admin 1	2005	81.2930	-0.0100	0.0281	0.9801	0.9354
Admin 1	2010	128.0000	0.0000	0.0046	0.9985	0.9778
Admin 1	2015	82.0000	-0.0038	0.0195	0.9902	0.9475
Admin 2	2000	11.4167	-0.0038	0.0396	0.9736	0.8853
Admin 2	2005	3.6464	-0.0100	0.0597	0.9245	0.9354
Admin 2	2010	4.9080	0.0000	0.0105	0.9921	0.9778
Admin 2	2015	3.7725	-0.0038	0.0653	0.9266	0.9475

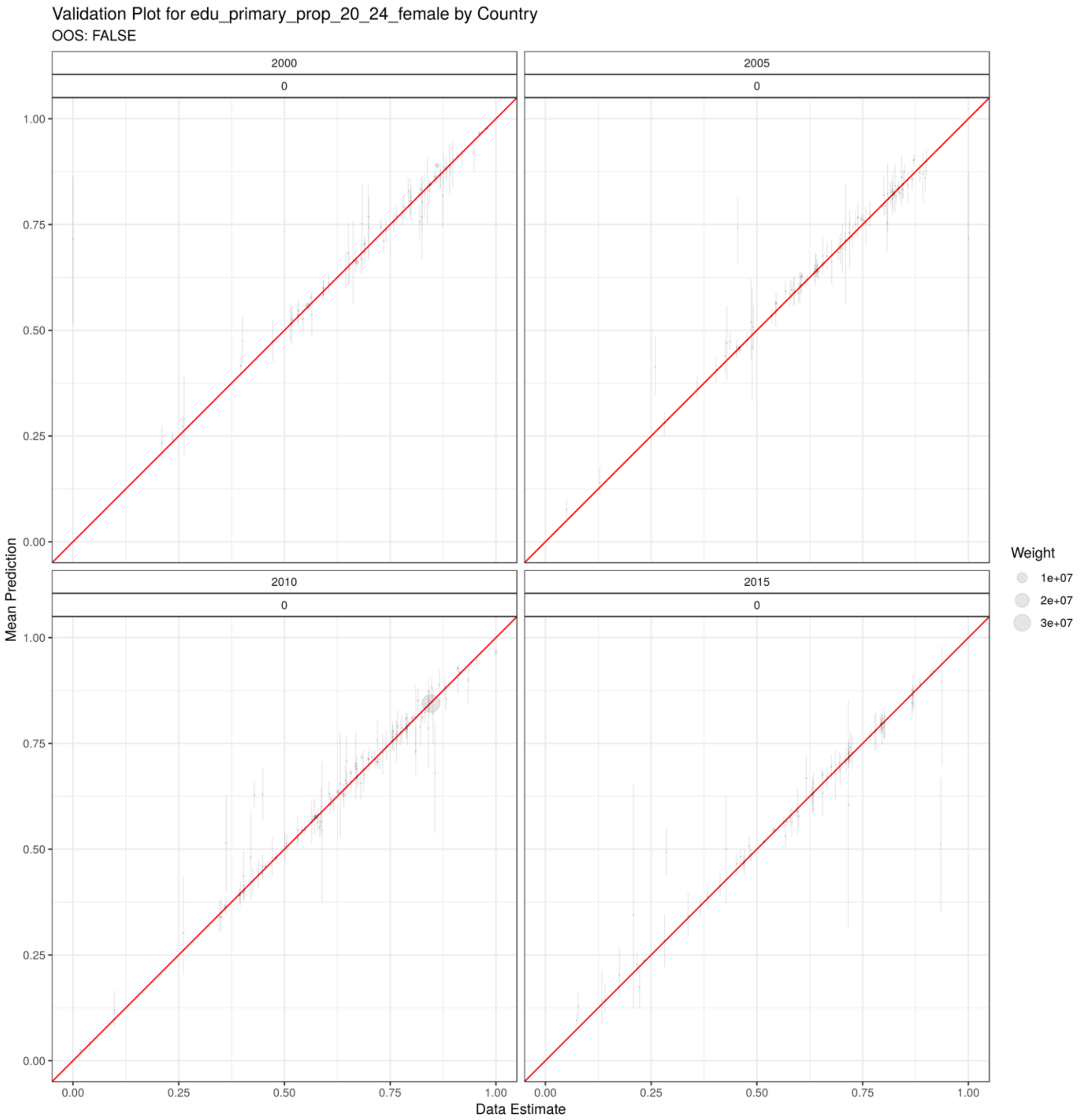

Supplementary Figure 58. 6–11 years education (women, ages 20–24) admin 0 aggregation
Comparison of in-sample education predictions aggregated to admin 0 with 95% uncertainty intervals plotted against admin 1 aggregated data observations.

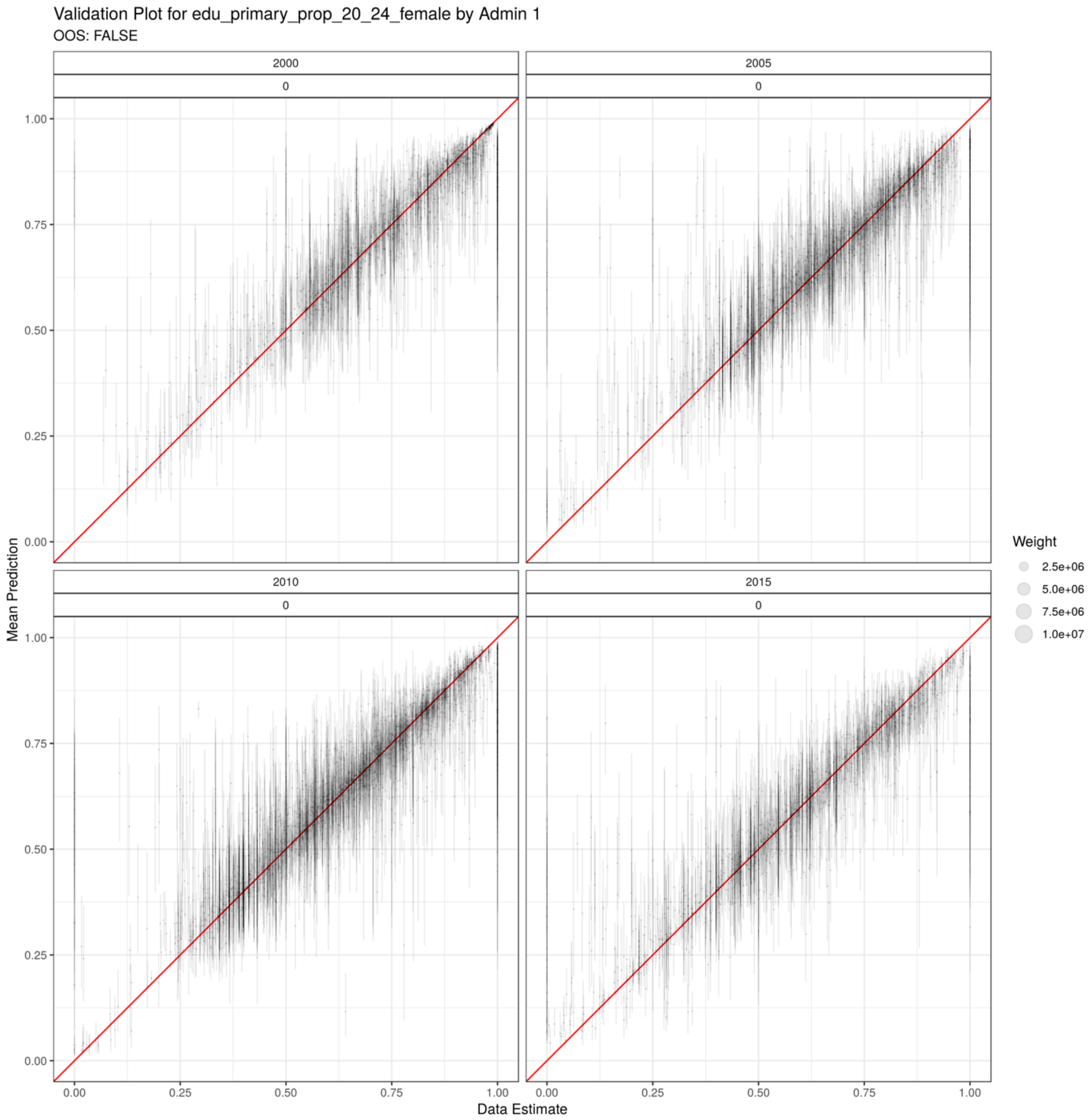

Supplementary Figure 59. 6–11 years education (women, ages 20–24) admin 1 aggregation
Comparison of in-sample education predictions aggregated to admin 1 with 95% uncertainty intervals plotted against admin 1 aggregated data observations.

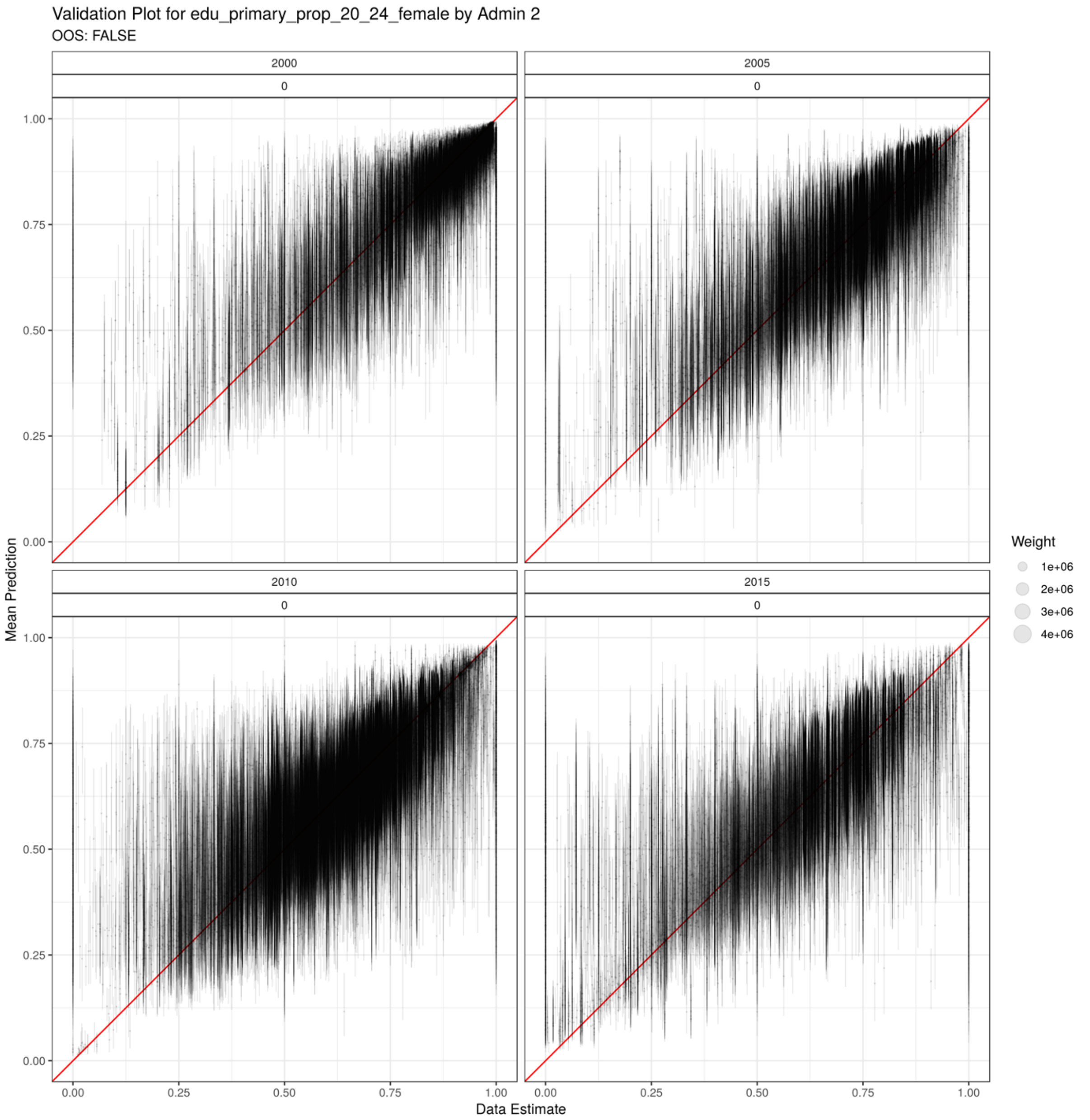

Supplementary Figure 60. 6–11 years education (women, ages 20–24) admin 2 aggregation
Comparison of in-sample education predictions aggregated to admin 2 with 95% uncertainty intervals plotted against admin 1 aggregated data observations.

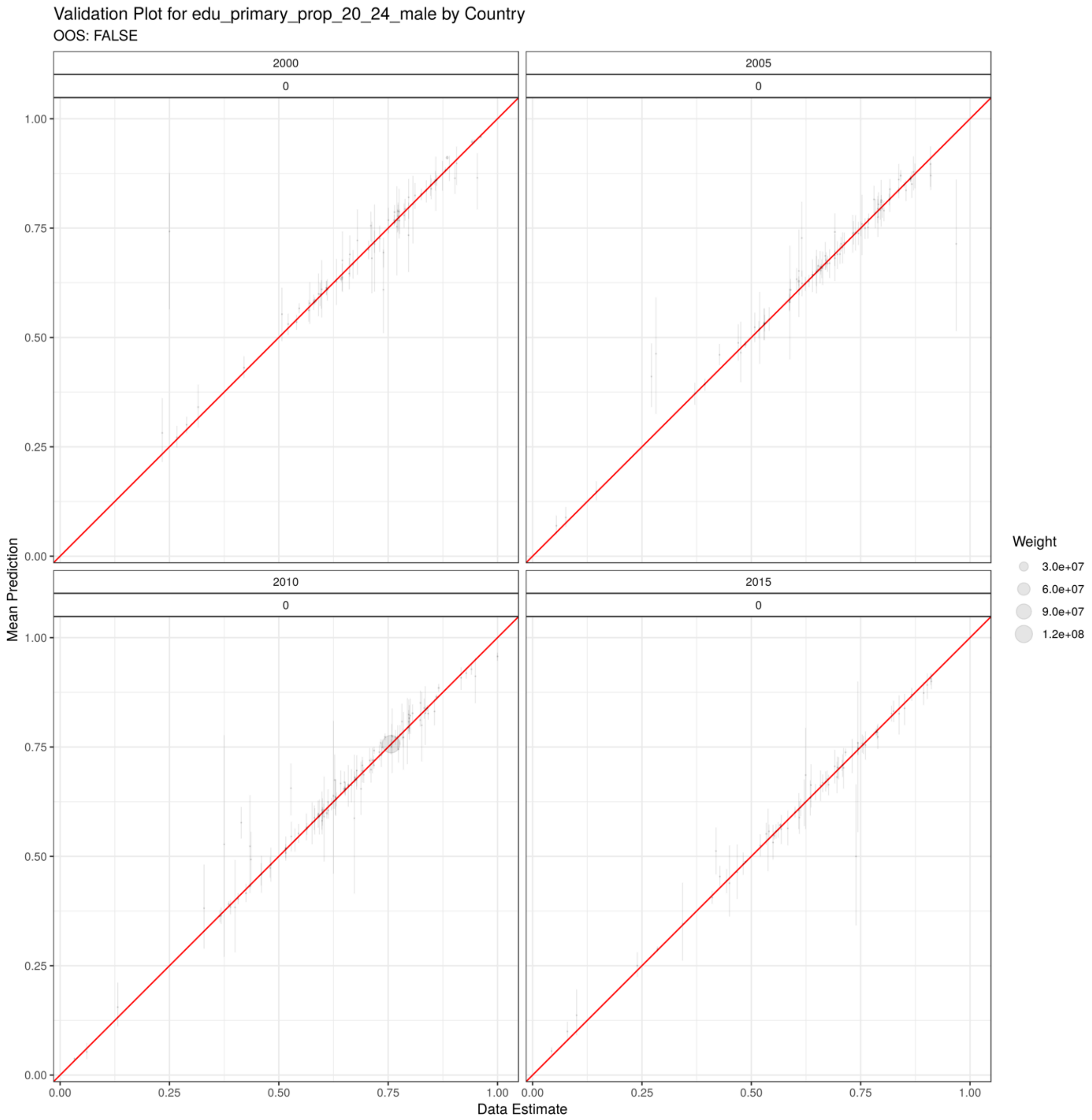

Supplementary Figure 61. 6–11 years education (men, ages 20–24) admin 0 aggregation
Comparison of in-sample education predictions aggregated to admin 0 with 95% uncertainty intervals plotted against admin 1 aggregated data observations.

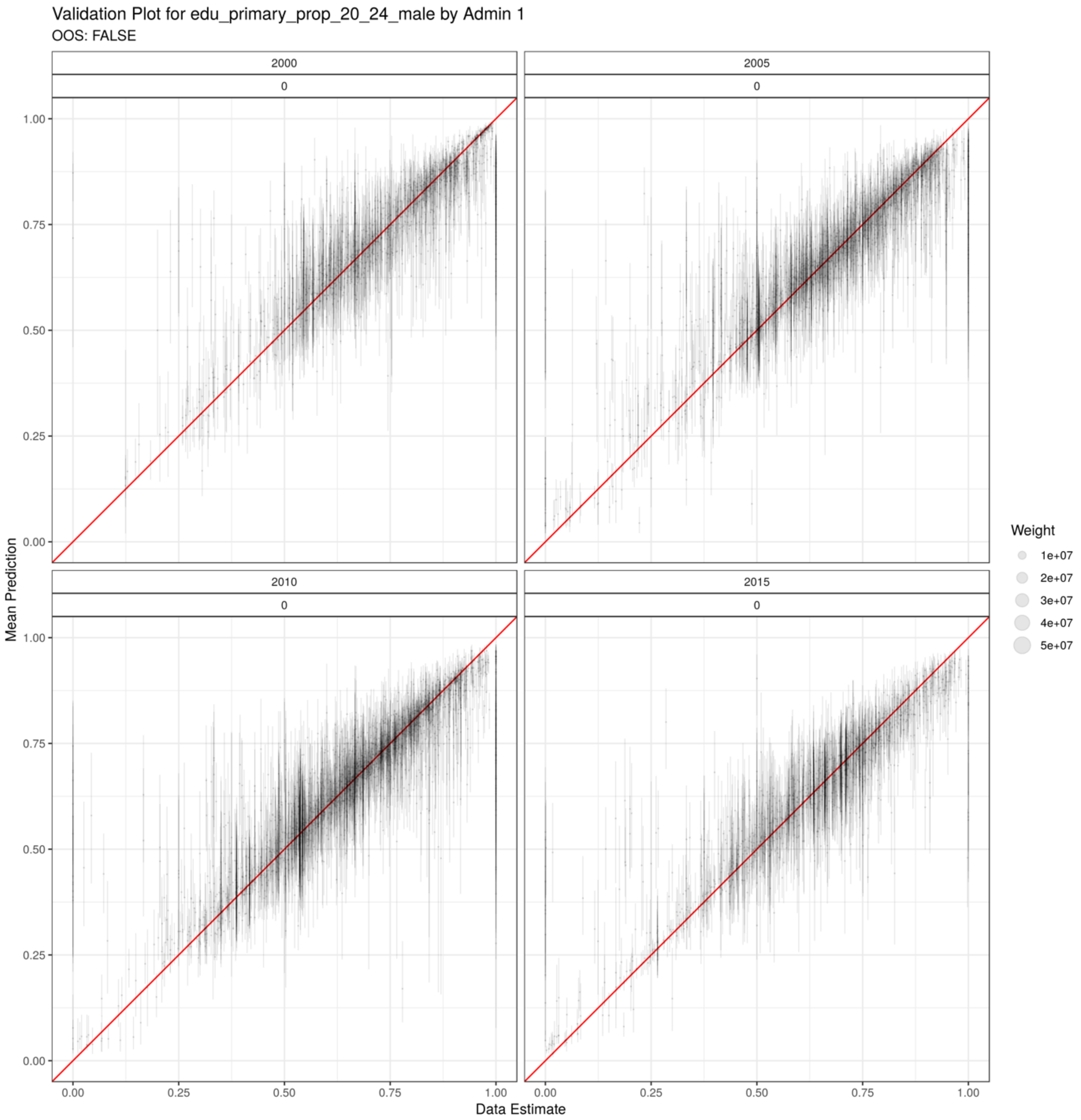

Supplementary Figure 62. 6–11 years education (men, ages 20–24) admin 1 aggregation
Comparison of in-sample education predictions aggregated to admin 1 with 95% uncertainty intervals plotted against admin 1 aggregated data observations.

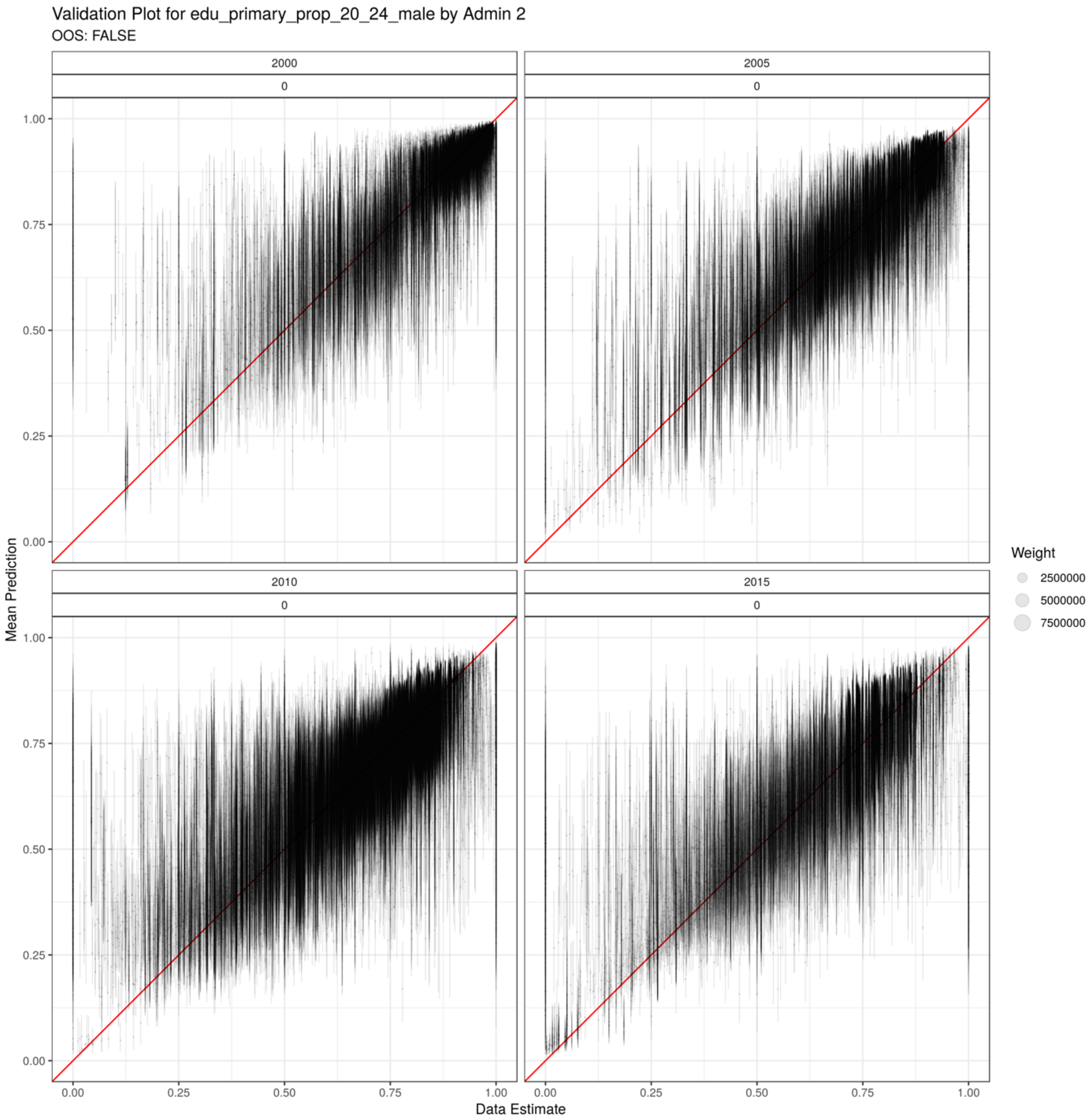

Supplementary Figure 63. 6–11 years education (men, ages 20–24) admin 2 aggregation
Comparison of in-sample education predictions aggregated to admin 2 with 95% uncertainty intervals plotted against admin 1 aggregated data observations.

Supplementary Table 36. In sample predictive metrics for mean years of education (women, ages 15–49), aggregated to admin 0, admin 1, and admin 2

Aggregation	Year	Median SS	Mean Err	RMSE	Corr.	95% Cov.
Admin 0	2000	12914.7806	-0.0131	0.2030	0.9938	0.9994
Admin 0	2005	15225.4068	-0.0815	0.1724	0.9977	0.9978
Admin 0	2010	19025.0000	-0.3599	0.3658	0.9915	0.9999
Admin 0	2015	13782.0000	-0.1180	0.2024	0.9952	0.9989
Admin 1	2000	1228.0000	-0.0131	0.2611	0.9916	0.9994
Admin 1	2005	750.0000	-0.0815	0.3053	0.9934	0.9978
Admin 1	2010	1077.8178	-0.3599	0.3759	0.9878	0.9999
Admin 1	2015	716.5000	-0.1180	0.2802	0.9924	0.9989
Admin 2	2000	114.9720	-0.0131	0.5549	0.9683	0.9994
Admin 2	2005	31.0000	-0.0815	0.7336	0.9654	0.9978
Admin 2	2010	41.1510	-0.3599	0.4123	0.9623	0.9999
Admin 2	2015	31.6316	-0.1180	0.5917	0.9695	0.9989

Supplementary Table 37. In sample predictive metrics for mean years of education (men, ages 15–49), aggregated to admin 0, admin 1, and admin 2

Aggregation	Year	Median SS	Mean Err	RMSE	Corr.	95% Cov.
Admin 0	2000	11706.67	-0.01185	0.211605	0.986663	0.999952
Admin 0	2005	13164.39	-0.05985	0.154427	0.996391	0.999056
Admin 0	2010	17541	-0.41332	0.415926	0.988207	0.99999
Admin 0	2015	12977	-0.11687	0.182033	0.989548	0.999756
Admin 1	2000	1126	-0.01185	0.268604	0.986174	0.999952
Admin 1	2005	690	-0.05985	0.286045	0.990389	0.999056
Admin 1	2010	1028.458	-0.41332	0.41939	0.986606	0.99999
Admin 1	2015	671	-0.11687	0.253781	0.986784	0.999756
Admin 2	2000	111.7156	-0.01185	0.580402	0.951606	0.999952
Admin 2	2005	28.89	-0.05985	0.665189	0.957281	0.999056
Admin 2	2010	38.1188	-0.41332	0.432549	0.961373	0.99999
Admin 2	2015	30	-0.11687	0.564951	0.949483	0.999756

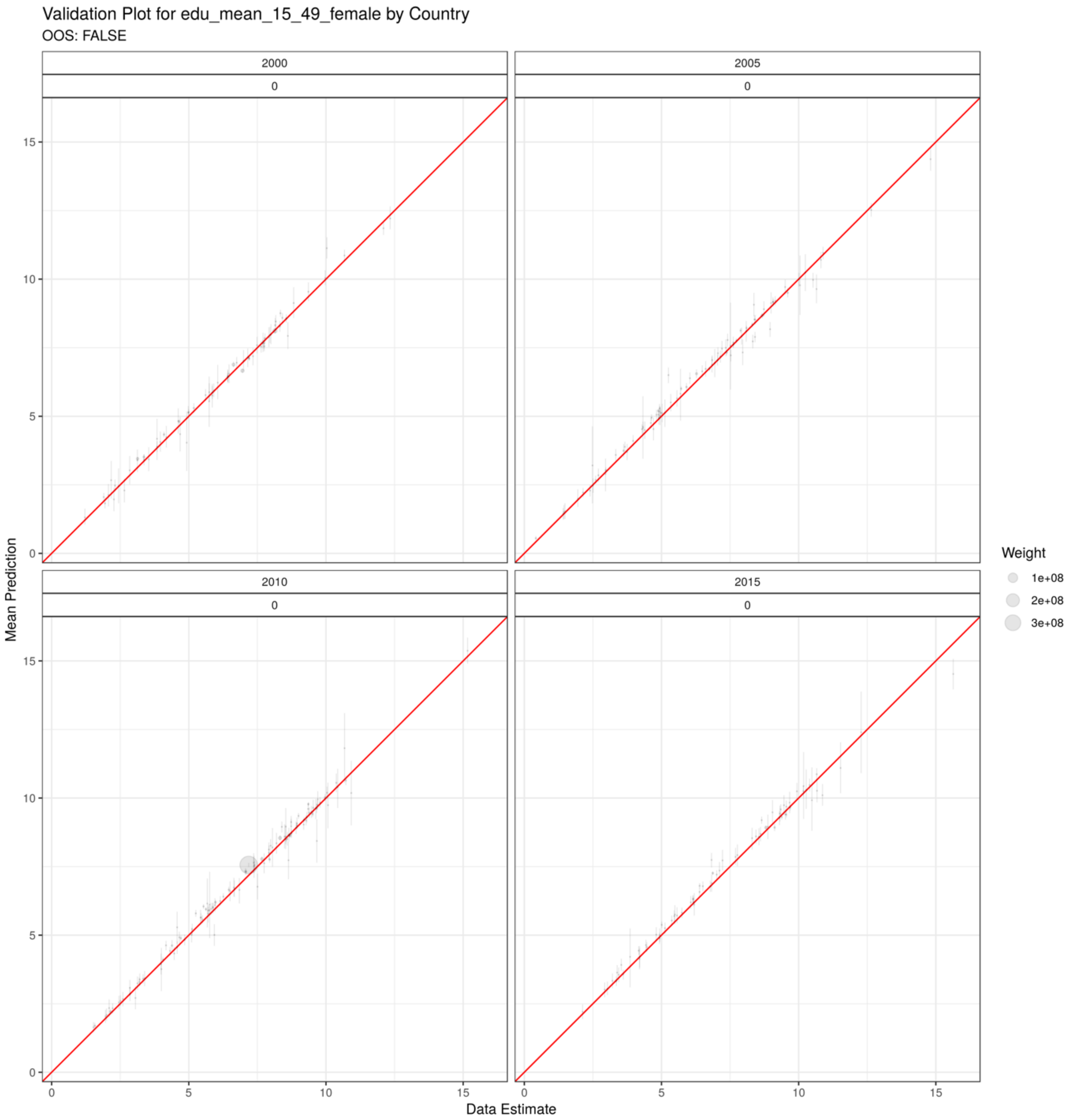

Supplementary Figure 64. Mean years education (women, ages 15–49) admin 0 aggregation
Comparison of in-sample education predictions aggregated to admin 0 with 95% uncertainty intervals plotted against admin 1 aggregated data observations.

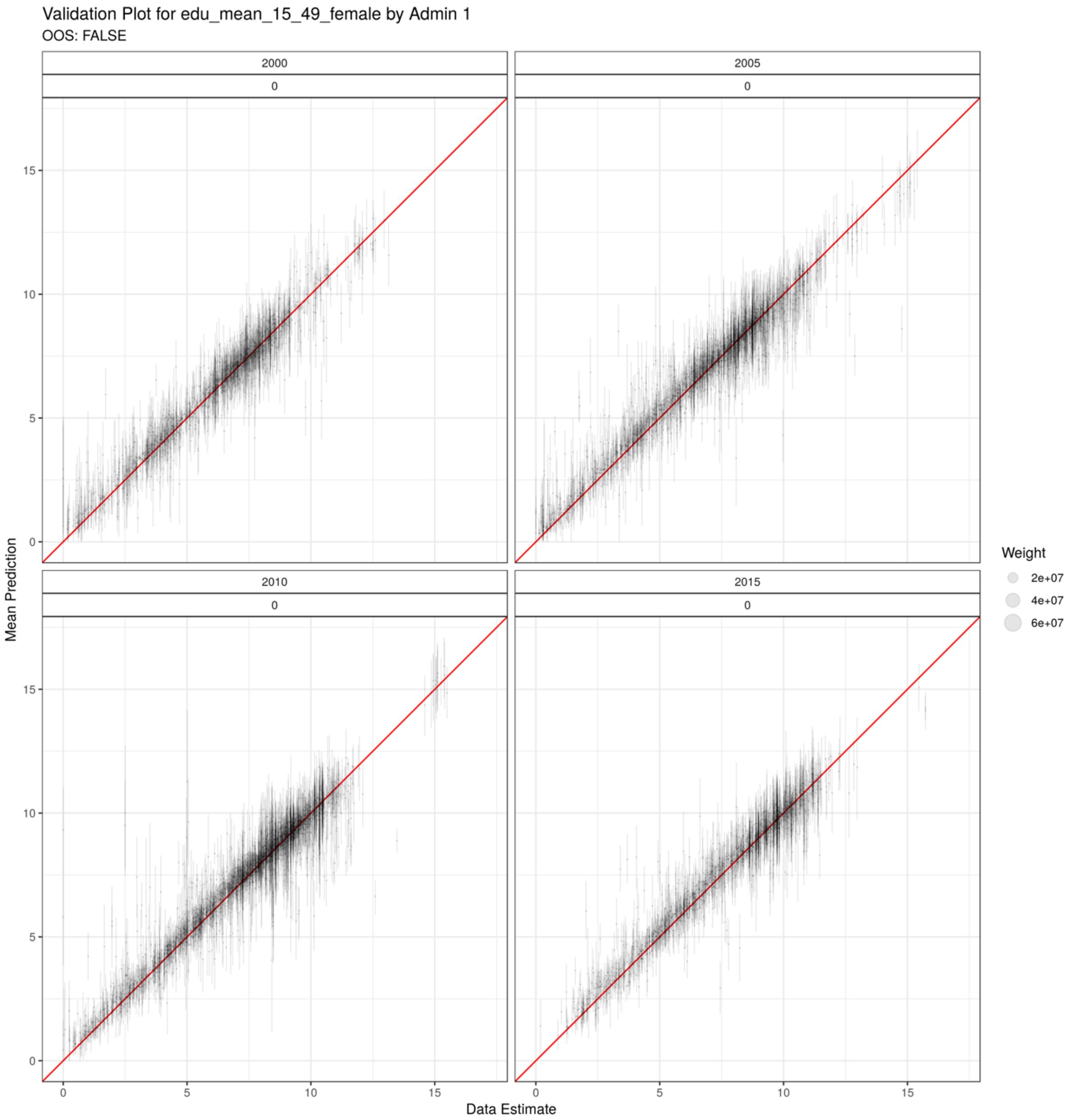

Supplementary Figure 65. Mean years education (women, ages 15–49) admin 1 aggregation
Comparison of in-sample education predictions aggregated to admin 1 with 95% uncertainty intervals plotted against admin 1 aggregated data observations.

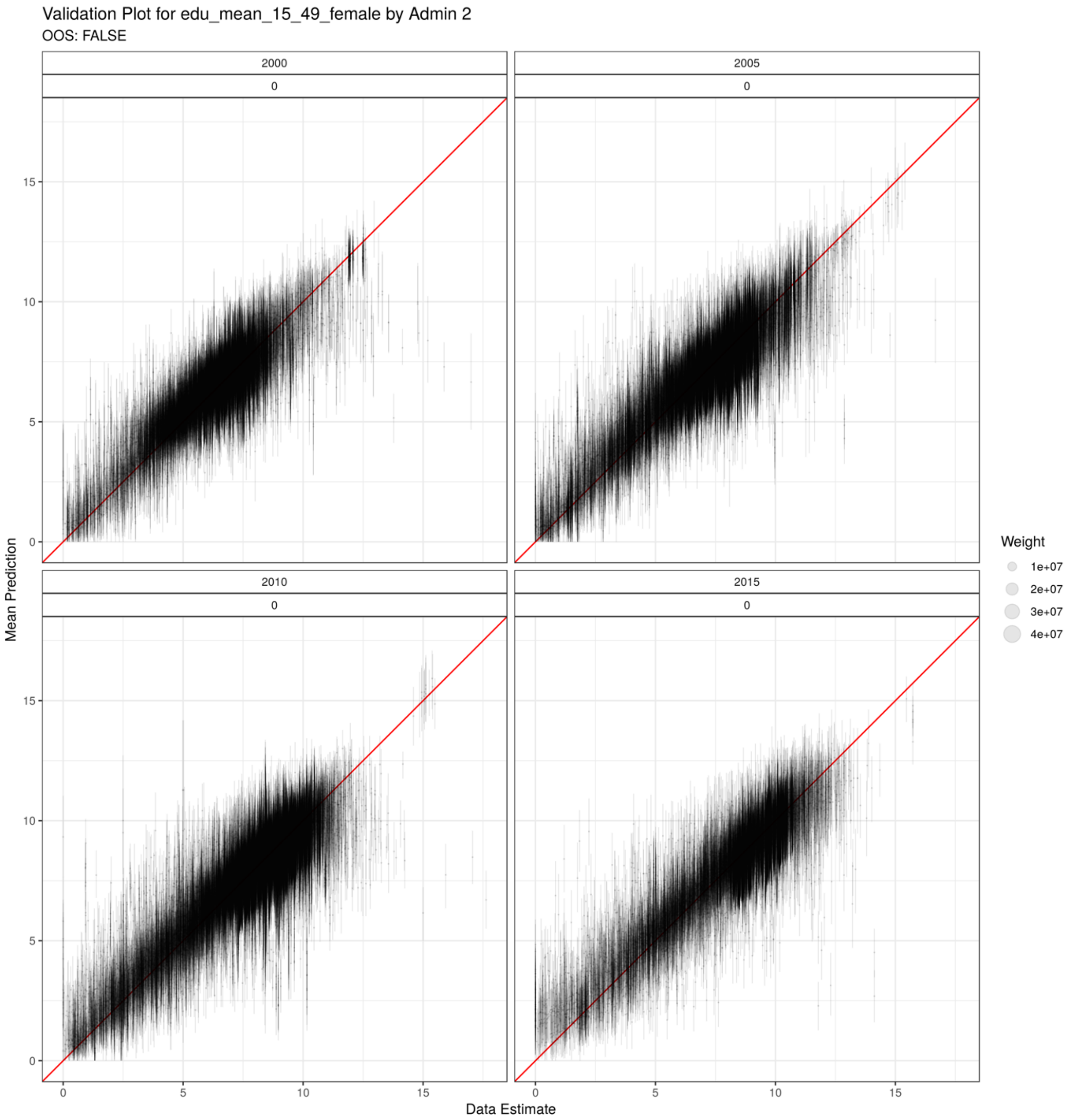

Supplementary Figure 66. Mean years education (women, ages 15–49) admin 2 aggregation
Comparison of in-sample education predictions aggregated to admin 2 with 95% uncertainty intervals plotted against admin 1 aggregated data observations.

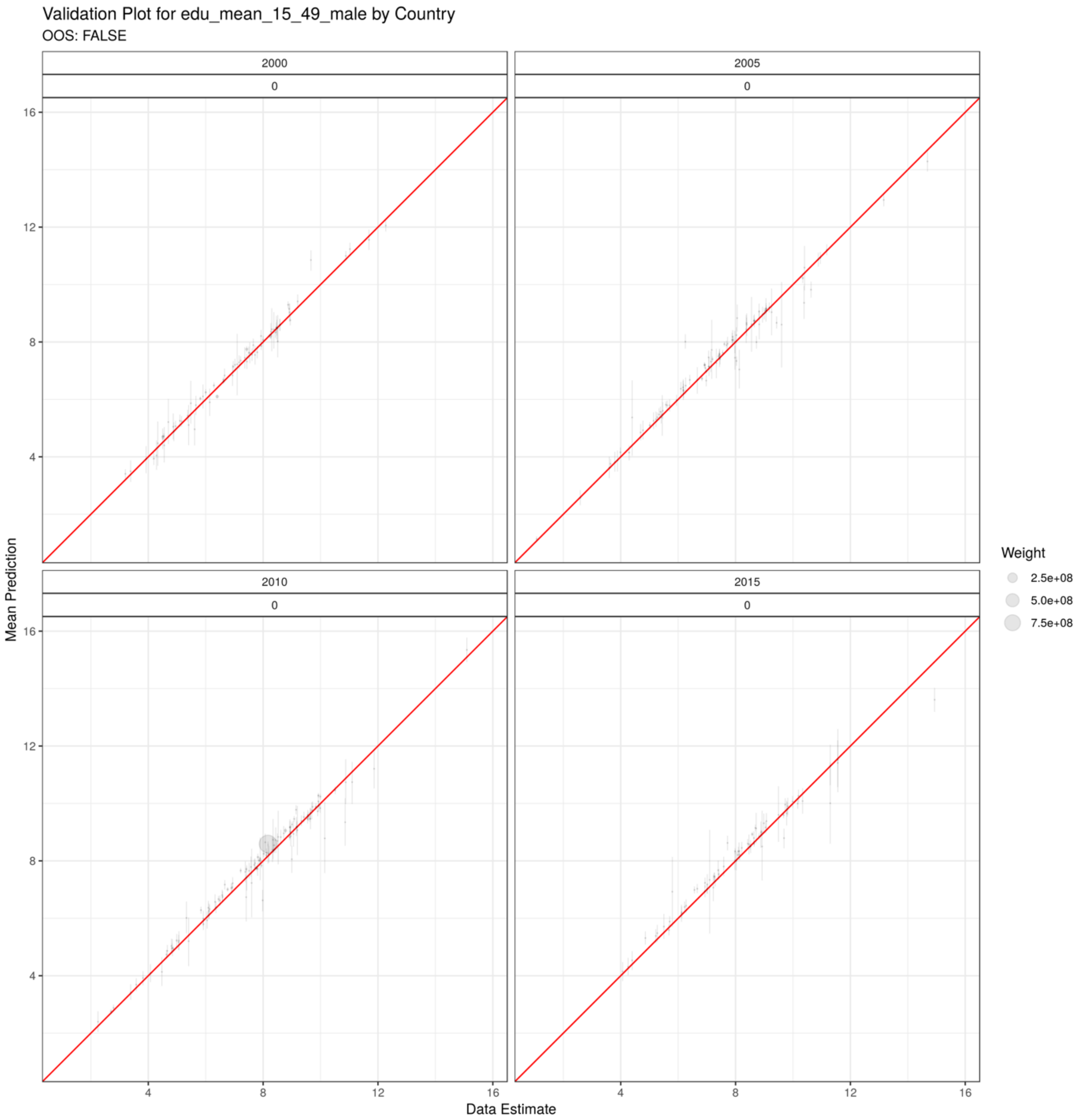

Supplementary Figure 67. Mean years education (men, ages 15–49) admin 0 aggregation
Comparison of in-sample education predictions aggregated to admin 0 with 95% uncertainty intervals plotted against admin 1 aggregated data observations.

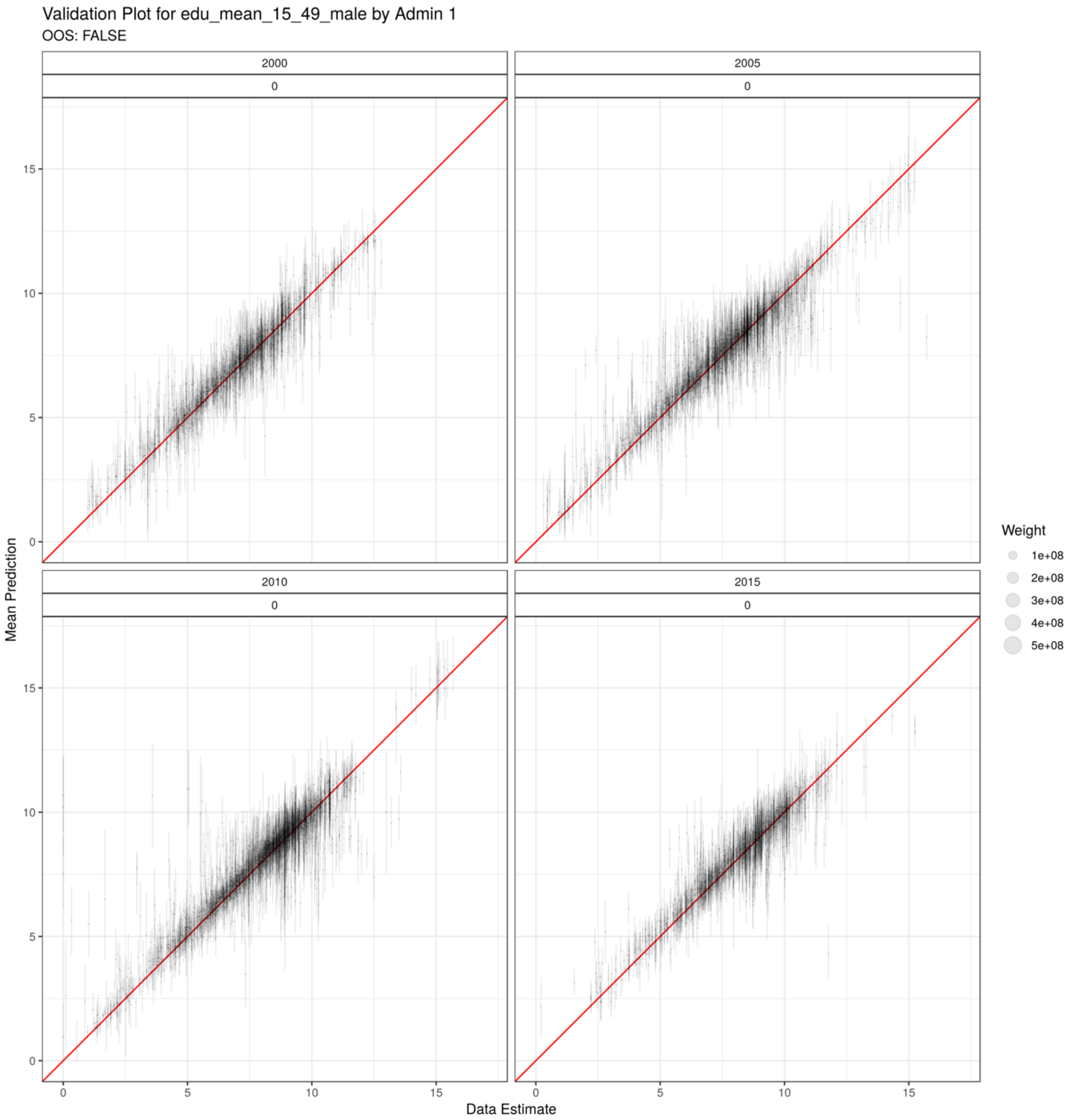

Supplementary Figure 68. Mean years education (men, ages 15–49) admin 1 aggregation
Comparison of in-sample education predictions aggregated to admin 1 with 95% uncertainty intervals plotted against admin 1 aggregated data observations.

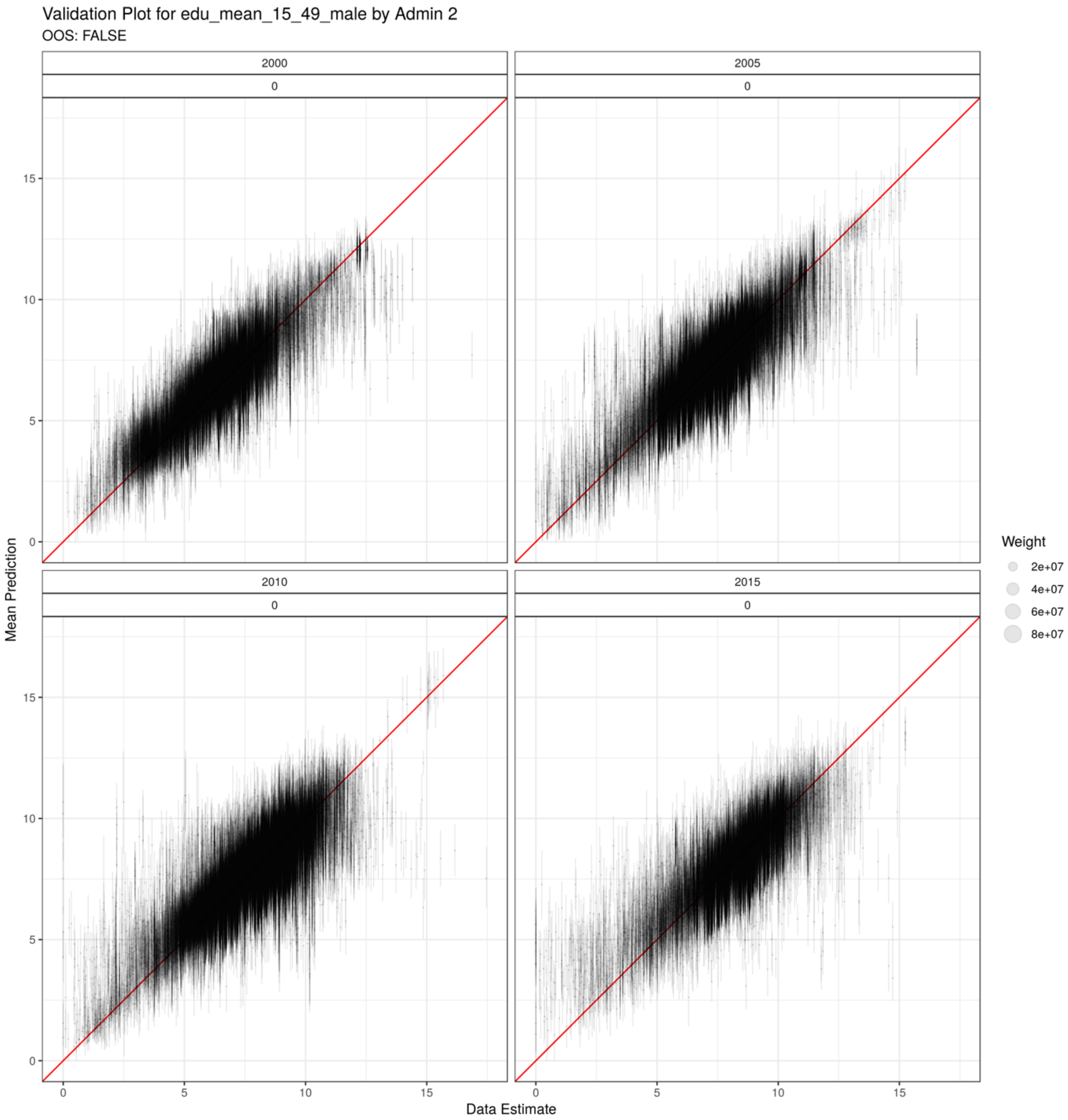

Supplementary Figure 69. Mean years education (men, ages 15–49) admin 2 aggregation

Comparison of in-sample education predictions aggregated to admin 2 with 95% uncertainty intervals plotted against admin 1 aggregated data observations.

Supplementary Table 38. In sample predictive metrics for mean years of education (women, ages 20–24), aggregated to admin 0, admin 1, and admin 2

Aggregation	Year	Median SS	Mean Err	RMSE	Corr.	95% Cov.
Admin 0	2000	2528.1849	-0.0236	0.1506	0.9976	0.9997
Admin 0	2005	2962.2179	-0.0589	0.1409	0.9984	0.9973
Admin 0	2010	3493.0000	-0.3365	0.3448	0.9958	0.9999
Admin 0	2015	2362.0000	-0.1001	0.1591	0.9975	0.9987
Admin 1	2000	215.0000	-0.0236	0.2148	0.9954	0.9997
Admin 1	2005	138.0000	-0.0589	0.2860	0.9946	0.9973
Admin 1	2010	190.0000	-0.3365	0.3528	0.9971	0.9999
Admin 1	2015	128.0000	-0.1001	0.2658	0.9930	0.9987
Admin 2	2000	19.0000	-0.0236	0.5171	0.9760	0.9997
Admin 2	2005	5.0471	-0.0589	0.7262	0.9688	0.9973
Admin 2	2010	6.2313	-0.3365	0.4070	0.9870	0.9999
Admin 2	2015	4.8115	-0.1001	0.6210	0.9659	0.9987

Supplementary Table 39. In sample predictive metrics for mean years of education (men, ages 20–24), aggregated to admin 0, admin 1, and admin 2

Aggregation	Year	Median SS	Mean Err	RMSE	Corr.	95% Cov.
Admin 0	2000	2141.782	0.014528	0.140889	0.995687	0.999944
Admin 0	2005	2206	-0.03624	0.106466	0.998316	0.999445
Admin 0	2010	2828	-0.31648	0.31901	0.99316	0.999985
Admin 0	2015	2306	-0.07158	0.122757	0.995043	0.999774
Admin 1	2000	192	0.014528	0.206854	0.993133	0.999944
Admin 1	2005	119.5162	-0.03624	0.27403	0.990543	0.999445
Admin 1	2010	174.1984	-0.31648	0.322961	0.99581	0.999985
Admin 1	2015	113	-0.07158	0.233732	0.986468	0.999774
Admin 2	2000	18.9655	0.014528	0.524231	0.963966	0.999944
Admin 2	2005	4.9025	-0.03624	0.655556	0.95533	0.999445
Admin 2	2010	5.962	-0.31648	0.341078	0.984378	0.999985
Admin 2	2015	4.3568	-0.07158	0.622937	0.929952	0.999774

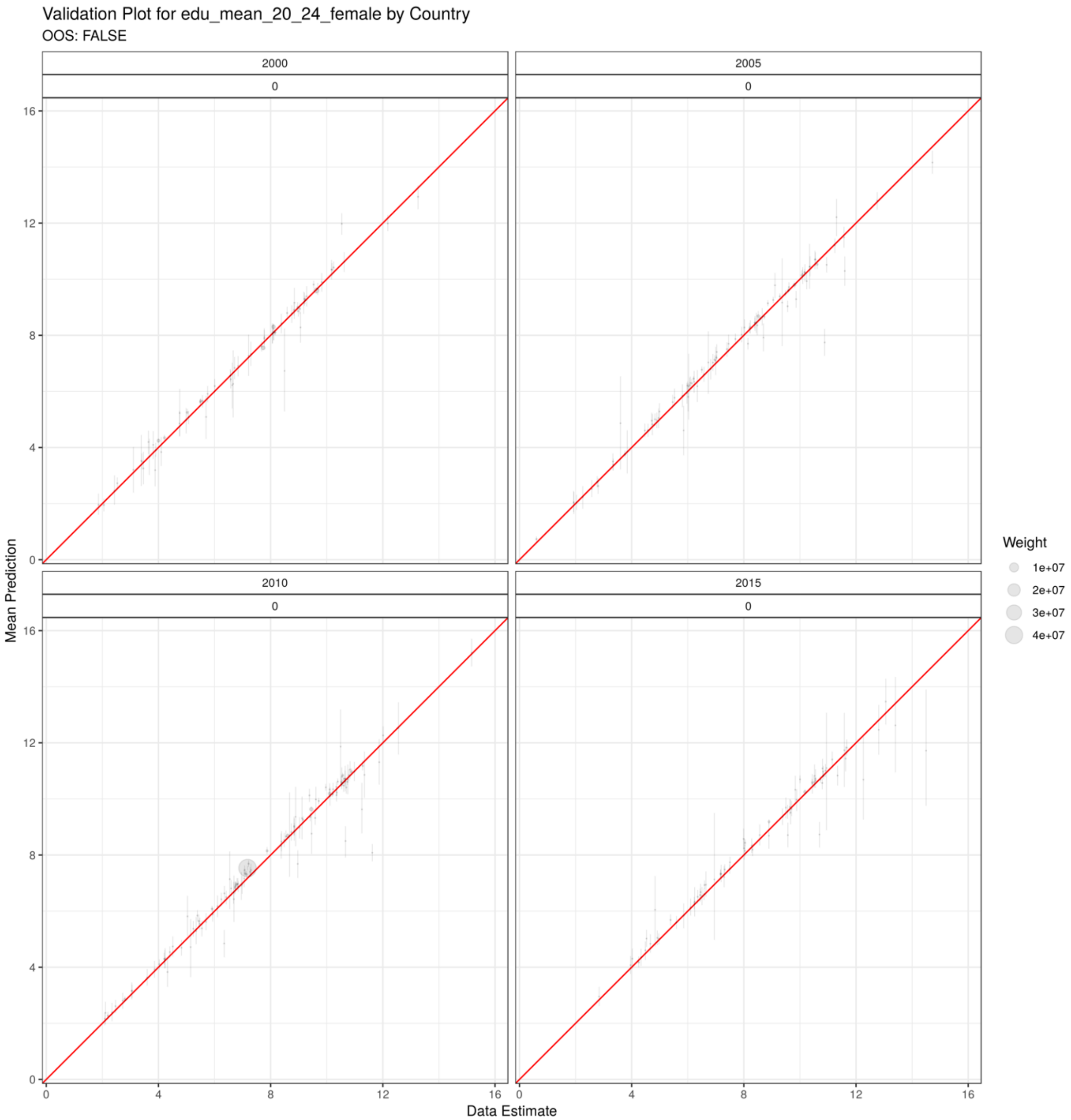

Supplementary Figure 70. Mean years education (women, ages 20–24) admin 0 aggregation

Comparison of in-sample education predictions aggregated to admin 0 with 95% uncertainty intervals plotted against admin 1 aggregated data observations.

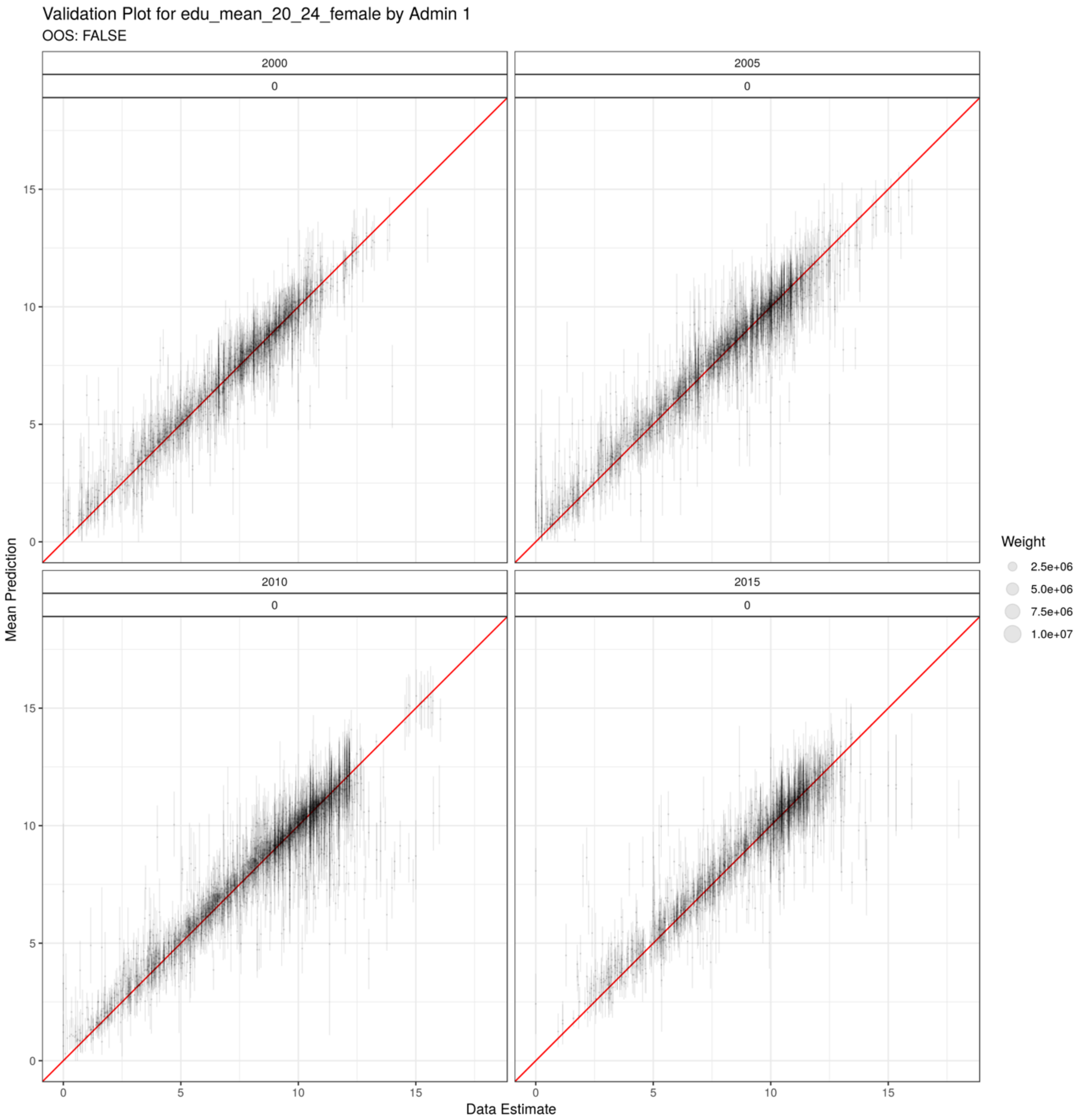

Supplementary Figure 71. Mean years education (women, ages 20–24) admin 1 aggregation
Comparison of in-sample education predictions aggregated to admin 1 with 95% uncertainty intervals plotted against admin 1 aggregated data observations.

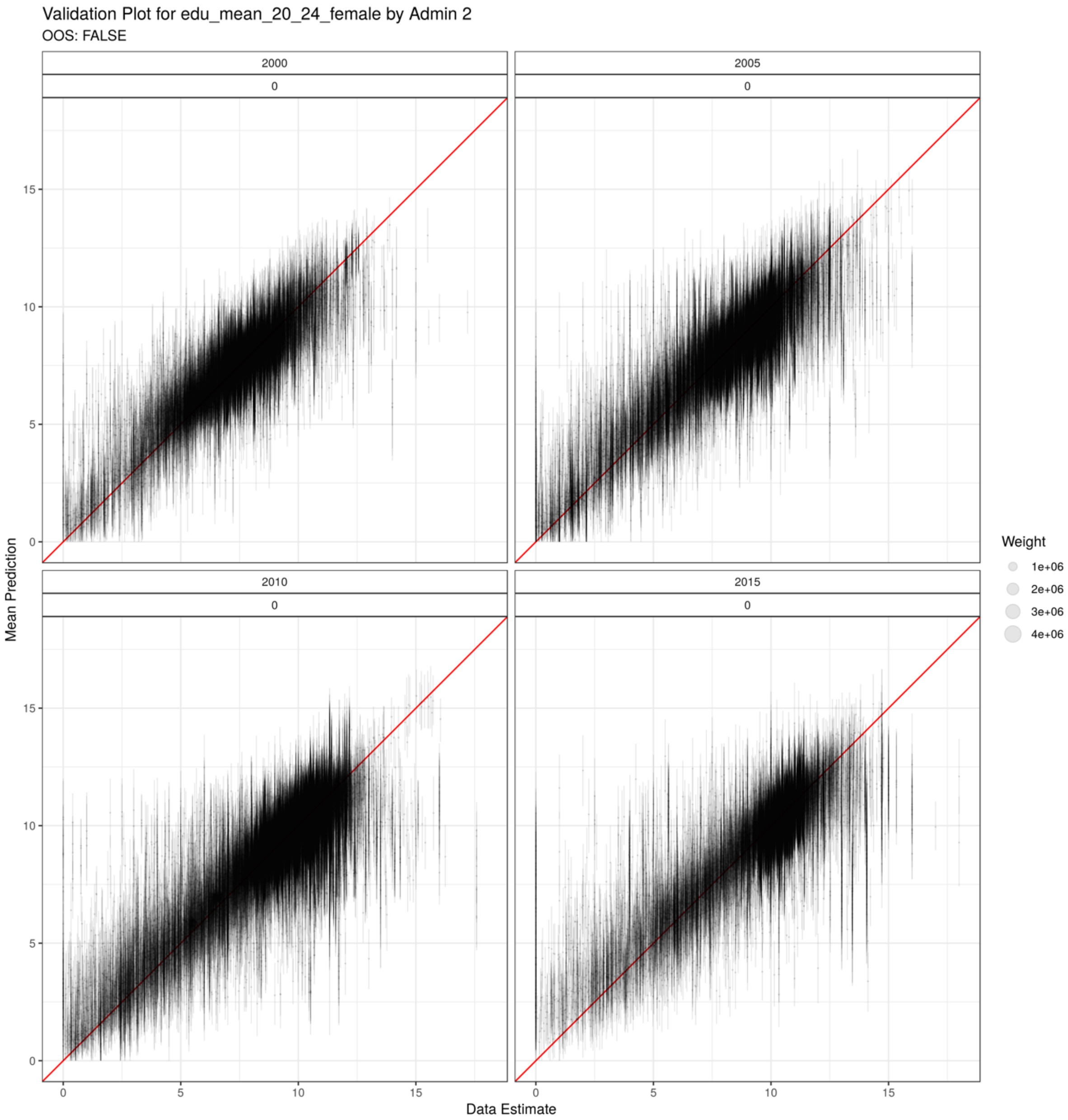

Supplementary Figure 72. Mean years education (women, ages 20–24) admin 2 aggregation
Comparison of in-sample education predictions aggregated to admin 2 with 95% uncertainty intervals plotted against admin 1 aggregated data observations.

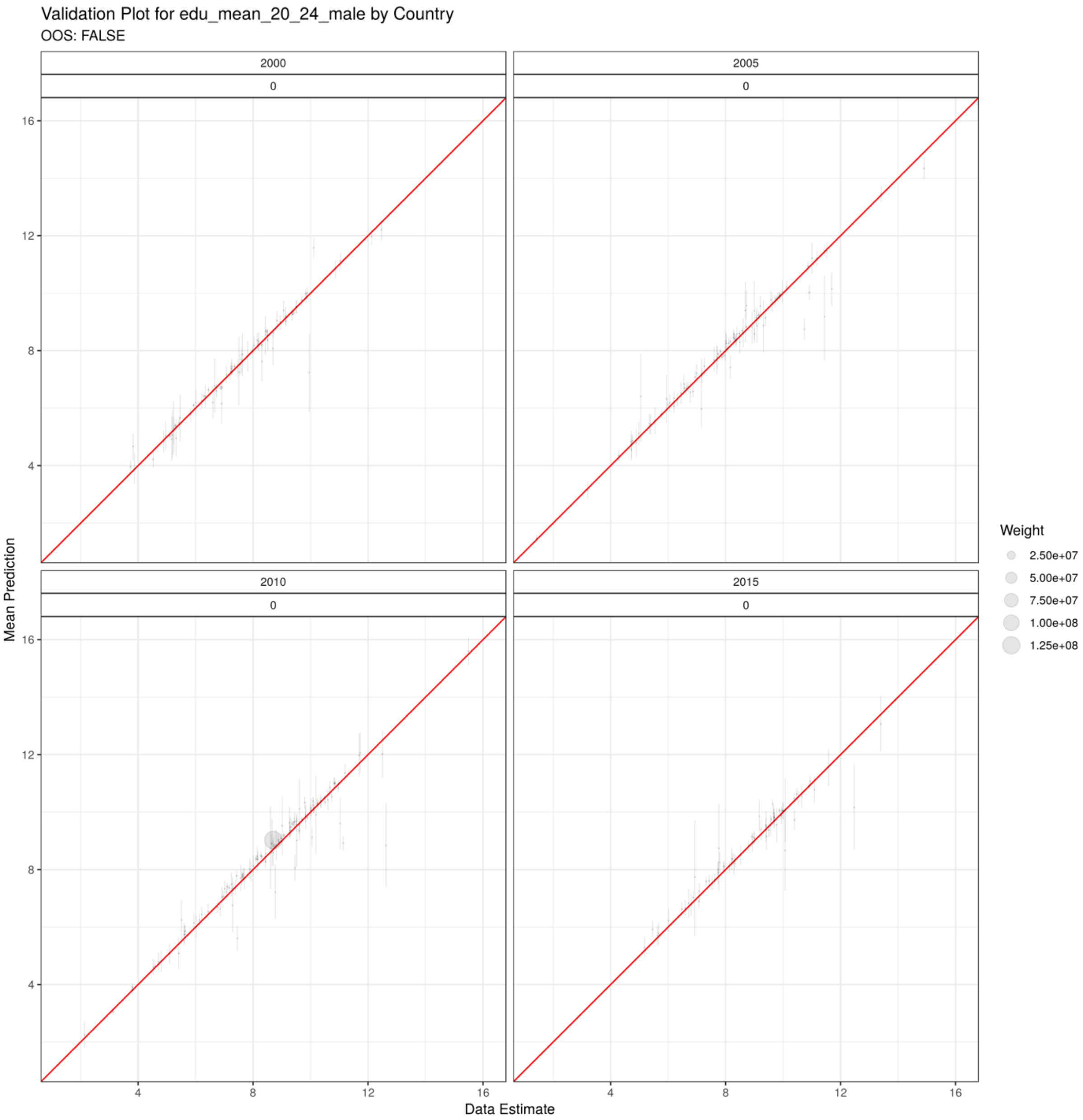

Supplementary Figure 73. Mean years education (men, ages 20–24) admin 0 aggregation
Comparison of in-sample education predictions aggregated to admin 0 with 95% uncertainty intervals plotted against admin 1 aggregated data observations.

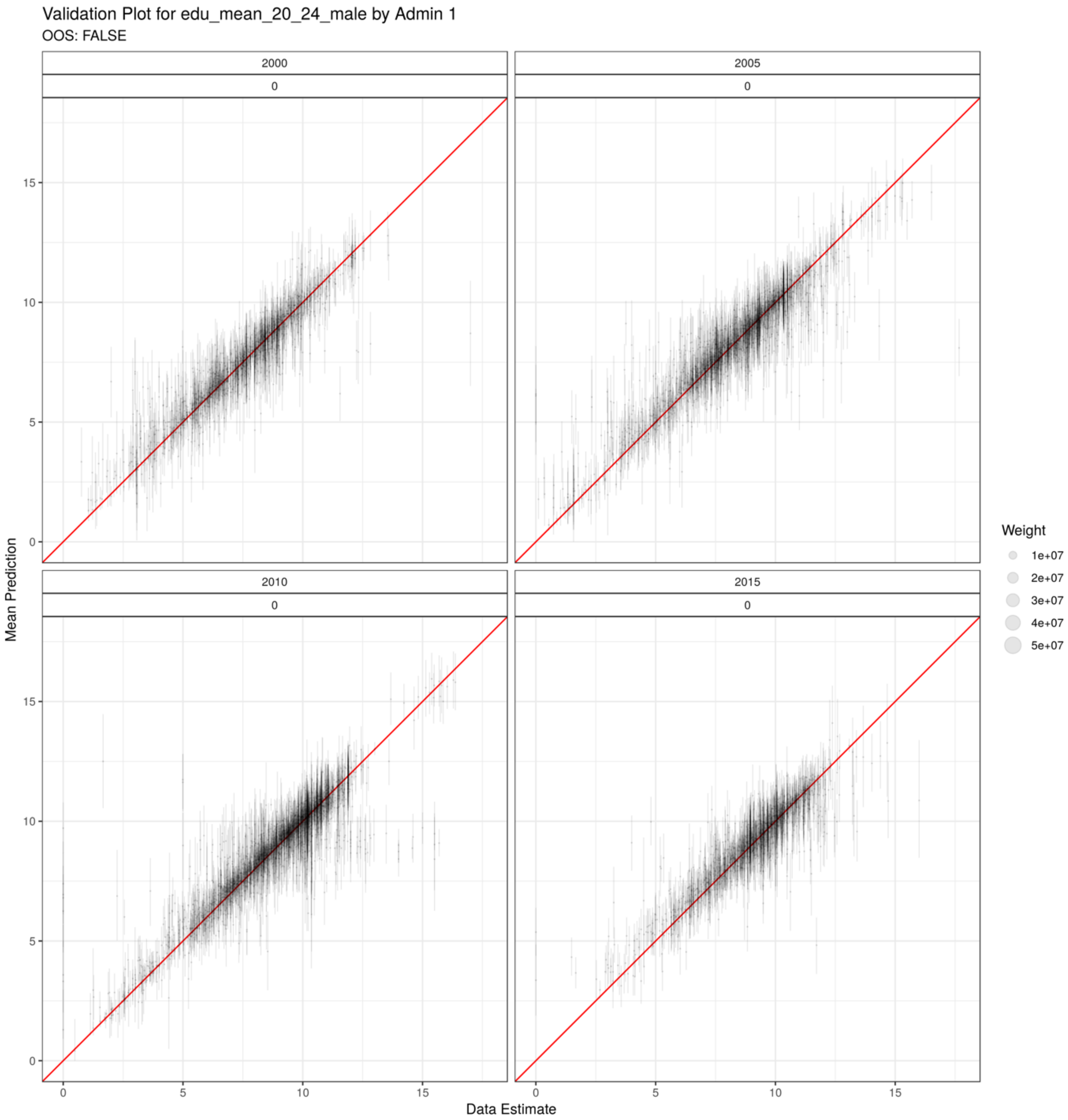

Supplementary Figure 74. Mean years education (men, ages 20–24) admin 1 aggregation

Comparison of in-sample education predictions aggregated to admin 1 with 95% uncertainty intervals plotted against admin 1 aggregated data observations.

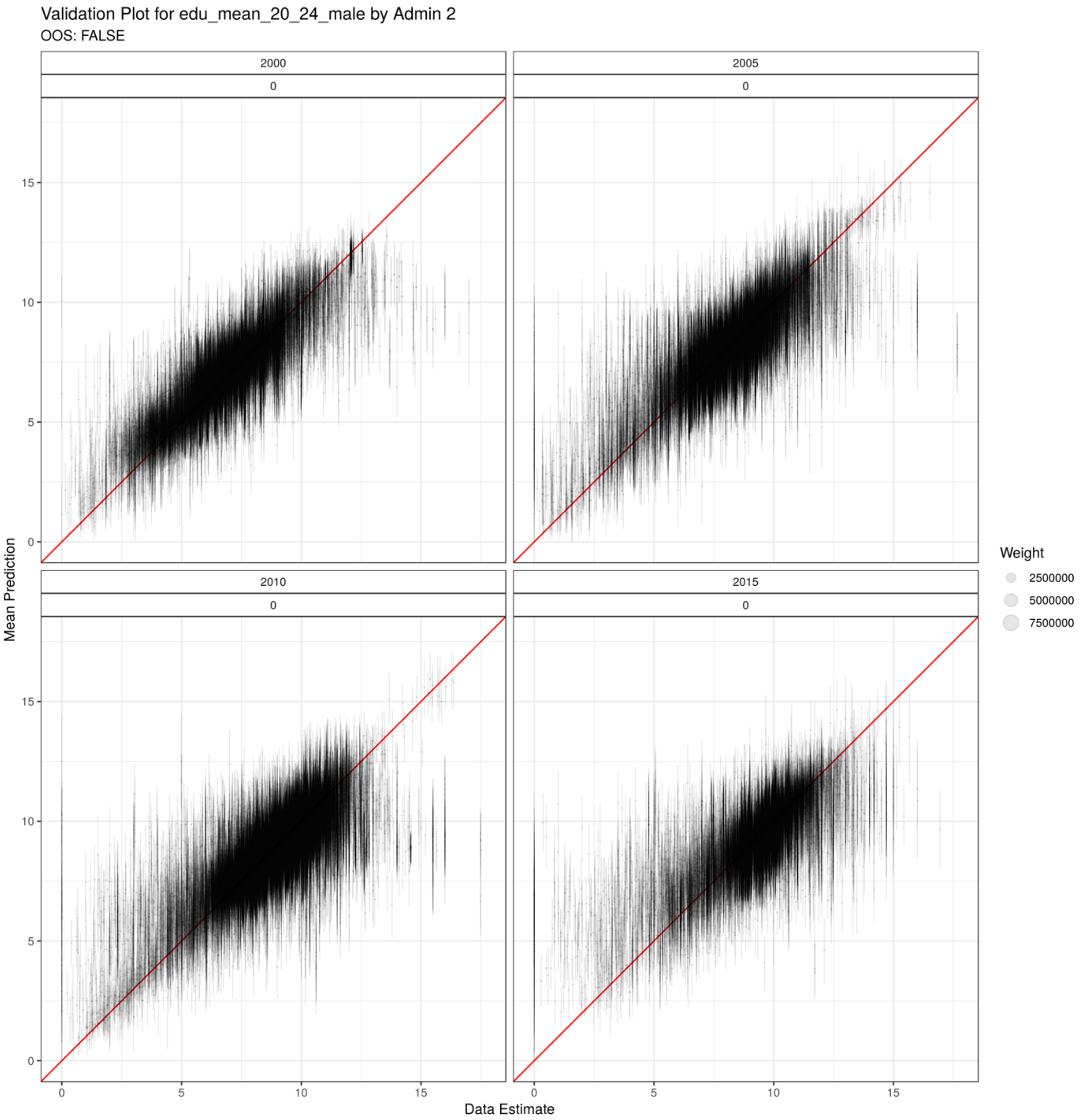

Supplementary Figure 75. Mean years education (men, ages 20–24) admin 2 aggregation
Comparison of in-sample education predictions aggregated to admin 2 with 95% uncertainty intervals plotted against admin 1 aggregated data observations.

Supplementary Table 40. Out of sample predictive metrics for zero years of education (women, ages 15–49), aggregated by fold to admin 0, admin 1, and admin 2

Aggregation	Year	Median SS	Mean Err	RMSE	Corr.	95% Cov.
Admin 0	2000	13964	0.021816	0.073398	0.886468	0.733808
Admin 0	2005	12507	0.043129	0.113146	0.874571	0.784065
Admin 0	2010	16512	-0.03541	0.07606	0.399688	0.600605
Admin 0	2015	13780	-0.00459	0.047058	0.951426	0.97231
Admin 1	2000	1317.03	0.021816	0.084249	0.873736	0.733808
Admin 1	2005	710	0.043129	0.123481	0.875769	0.784065
Admin 1	2010	1059	-0.03541	0.100874	0.372317	0.600605
Admin 1	2015	802	-0.00459	0.059673	0.930041	0.97231
Admin 2	2000	149.0875	0.021816	0.098344	0.843907	0.733808
Admin 2	2005	33.0365	0.043129	0.138161	0.852629	0.784065
Admin 2	2010	53.91765	-0.03541	0.10243	0.371169	0.600605
Admin 2	2015	36.1915	-0.00459	0.077226	0.889754	0.97231

Supplementary Table 41. Out of sample predictive metrics for zero years of education (women, ages 20–24), aggregated by fold to admin 0, admin 1, and admin 2

Aggregation	Year	Median SS	Mean Err	RMSE	Corr.	95% Cov.
Admin 0	2000	2582	0.028997	0.06382	0.903345	0.58698
Admin 0	2005	2409	-0.00012	0.090847	0.878222	0.712959
Admin 0	2010	2751	-0.09621	0.104118	0.73513	0.449646
Admin 0	2015	2554	-0.00825	0.045658	0.939149	0.928603
Admin 1	2000	228.5	0.028997	0.081401	0.856628	0.58698
Admin 1	2005	112.7412	-0.00012	0.113385	0.850773	0.712959
Admin 1	2010	185.178	-0.09621	0.169225	0.187396	0.449646
Admin 1	2015	141	-0.00825	0.069204	0.867662	0.928603
Admin 2	2000	24	0.028997	0.099093	0.798014	0.58698
Admin 2	2005	5.5618	-0.00012	0.134105	0.809085	0.712959
Admin 2	2010	8.32155	-0.09621	0.184387	0.162311	0.449646
Admin 2	2015	6	-0.00825	0.089893	0.793993	0.928603

Supplementary Table 42. Out of sample predictive metrics for mean years of education (women, ages 15–49), aggregated by fold to admin 0, admin 1, and admin 2

Aggregation	Year	Median SS	Mean Err	RMSE	Corr.	95% Cov.
Admin 0	2000	13966	-0.21718	0.454815	0.978911	0.998792
Admin 0	2005	13247	-0.49357	0.807787	0.955519	0.99595
Admin 0	2010	16821	-0.45568	0.864634	0.942702	0.998226
Admin 0	2015	13894	0.129772	1.100053	0.825949	0.995274
Admin 1	2000	1136.5	-0.21718	0.540983	0.97335	0.998792
Admin 1	2005	666.0305	-0.49357	0.907992	0.954133	0.99595
Admin 1	2010	1008	-0.45568	0.990256	0.941877	0.998226
Admin 1	2015	779	0.129772	1.181919	0.849278	0.995274
Admin 2	2000	147	-0.21718	0.855615	0.932814	0.998792
Admin 2	2005	30.9308	-0.49357	1.220395	0.917461	0.99595
Admin 2	2010	45.9816	-0.45568	1.265249	0.904898	0.998226
Admin 2	2015	39	0.129772	1.382121	0.828785	0.995274

Supplementary Table 43. Out of sample predictive metrics for mean years of education (women, ages 20–24), aggregated by fold to admin 0, admin 1, and admin 2

Aggregation	Year	Median SS	Mean Err	RMSE	Corr.	95% Cov.
Admin 0	2000	2582	-0.61007	1.506197	0.814281	0.995982
Admin 0	2005	2472.404	0.738274	1.490048	0.821111	0.946285
Admin 0	2010	2940	0.007172	1.755524	0.709886	0.97415
Admin 0	2015	2554	0.842747	1.107054	0.887816	0.987735
Admin 1	2000	224	-0.61007	1.589455	0.813813	0.995982
Admin 1	2005	119	0.738274	1.930784	0.75304	0.946285
Admin 1	2010	193	0.007172	2.209842	0.651887	0.97415
Admin 1	2015	138.11	0.842747	1.441946	0.785729	0.987735
Admin 2	2000	24.1623	-0.61007	1.780478	0.778246	0.995982
Admin 2	2005	5.5624	0.738274	2.293885	0.686054	0.946285
Admin 2	2010	8.897	0.007172	2.423214	0.627152	0.97415
Admin 2	2015	5.7339	0.842747	1.873254	0.652194	0.987735

5.3.3 Post estimation calibration to national estimates

In order to leverage national-level data included in GBD 2017[41], but outside the scope of our current geospatial modelling framework, and to ensure perfect calibration between our estimates and GBD 2017 national-level estimates, we performed a post hoc calibration to each of our 1,000 candidate maps. For each posterior draw we calculated population-weighted pixel aggregations to a national level and compared these country-year estimates to the GBD 2017country-years. We defined the raking factor to be the ratio between the GBD 2017 estimate and our current estimates. Finally, we multiplied each of our pixels in a country-year by its associated raking factor. This ensured a perfect calibration between our geospatial estimates and GBD 2017 national-level estimates, while preserving our estimated within-country geospatial and temporal variation.

To allow comparison between our modelled estimates and the GBD 2017 national-level estimates to which they were calibrated, Supplementary Figs 76–91 plot mean uncalibrated estimates from the model-based geostatistics (MBG) process aggregated to the national-level ("MBG mean") as compared to the GBD national estimates ("GBD mean") for all modelled years.

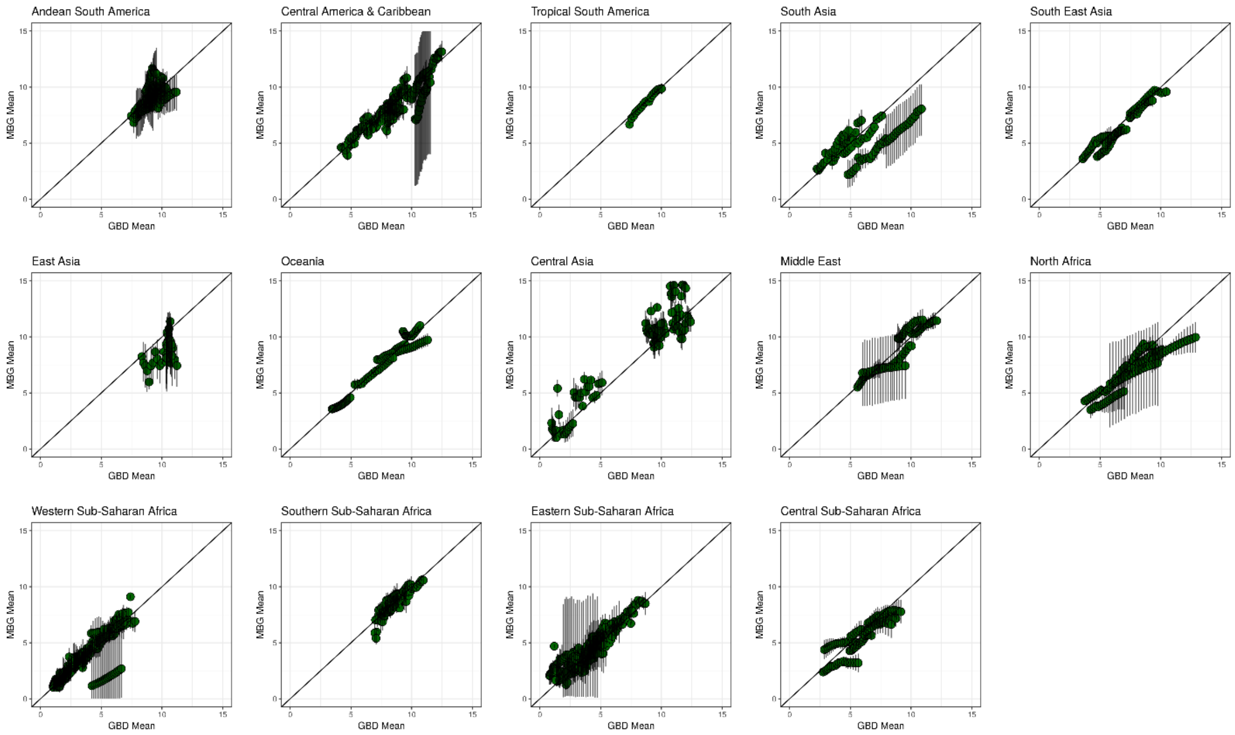

Supplementary Figure 76. Mean years education (women, ages 15–49) comparison to GBD national estimates.

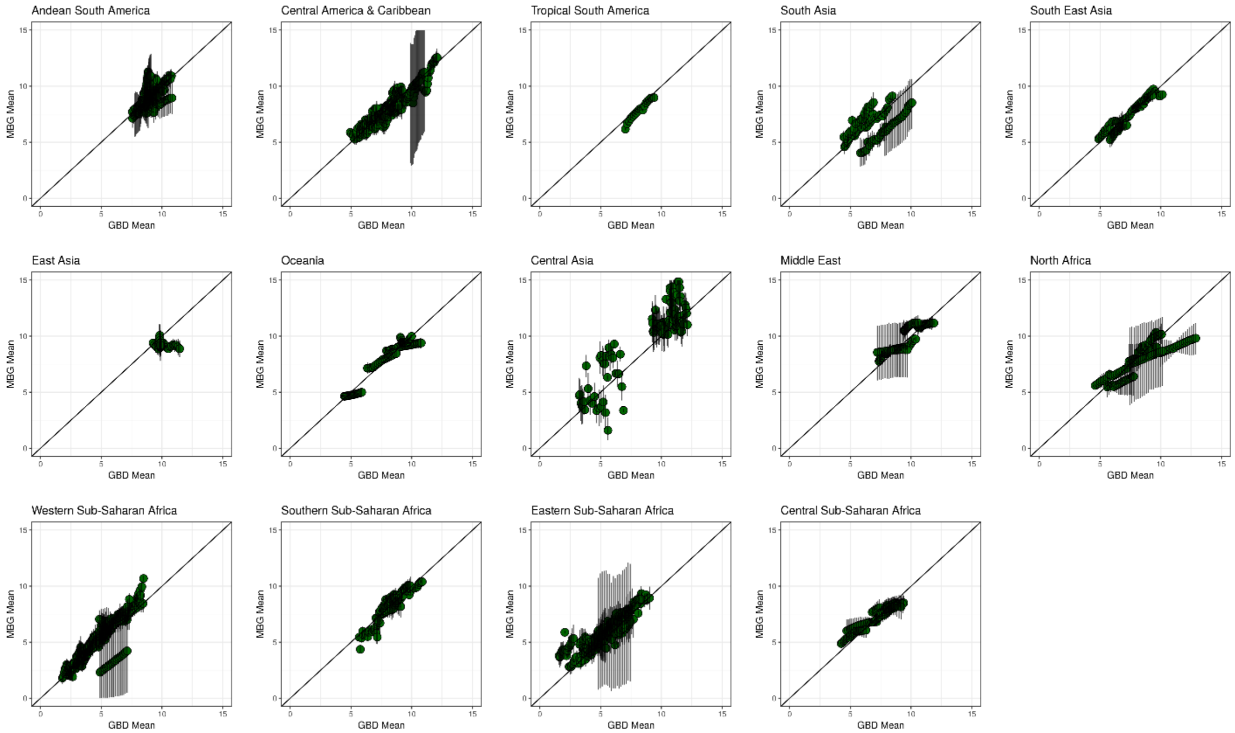

Supplementary Figure 77. Mean years education (men, ages 15–49) comparison to GBD national estimates.

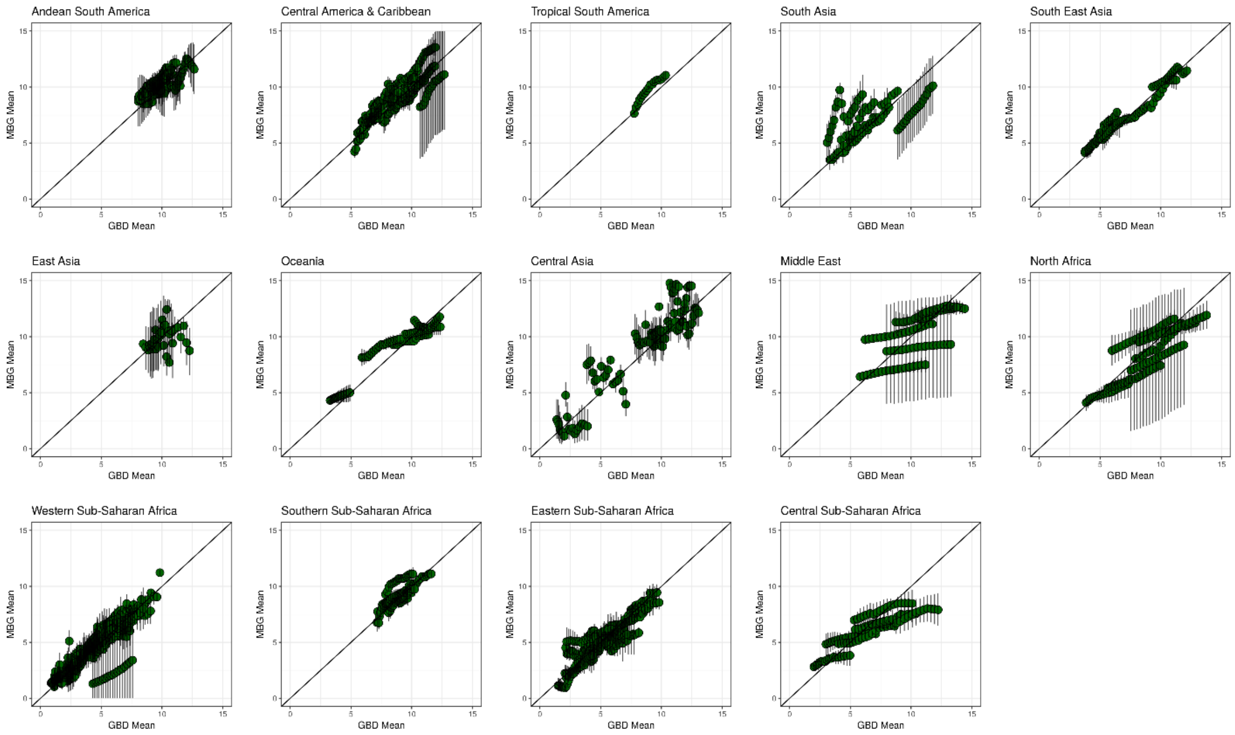

Supplementary Figure 78. Mean years education (women, ages 20–24) comparison to GBD national estimates.

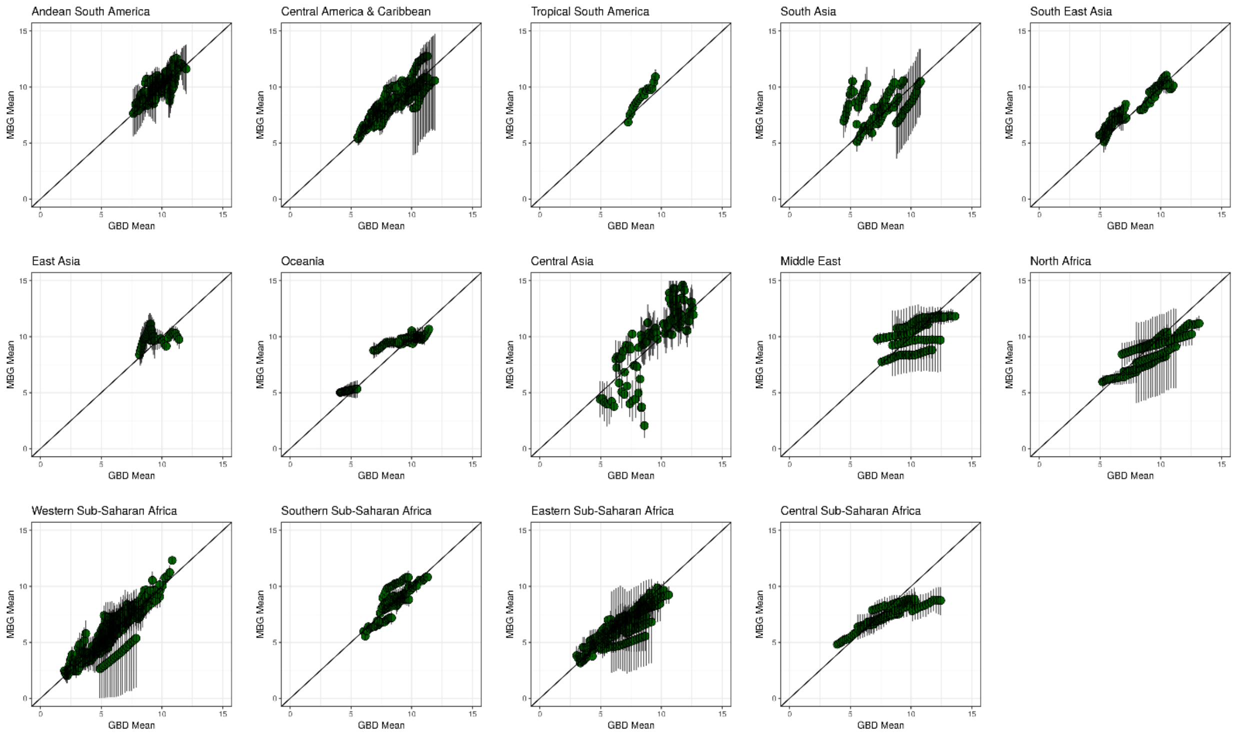

Supplementary Figure 79. Mean years education (men, ages 20–24) comparison to GBD national estimates.

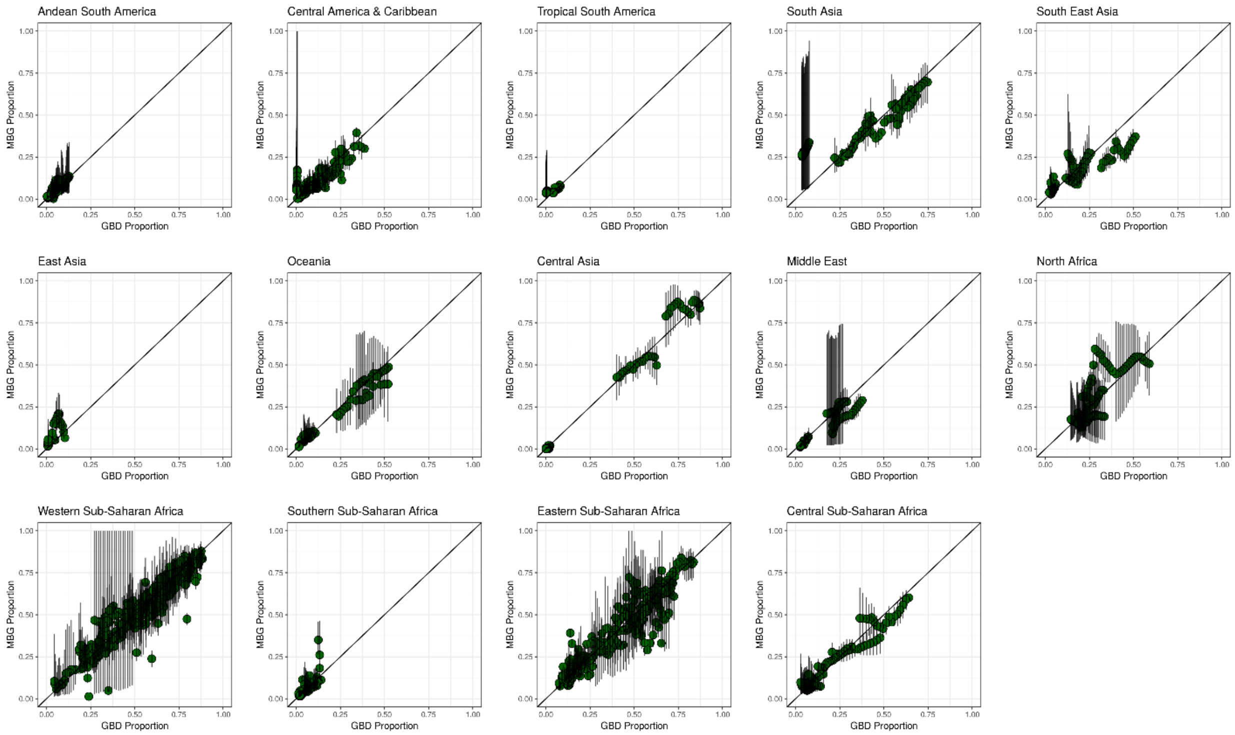

Supplementary Figure 80. Zero years of education (women, ages 15–49) comparison to GBD national estimates.

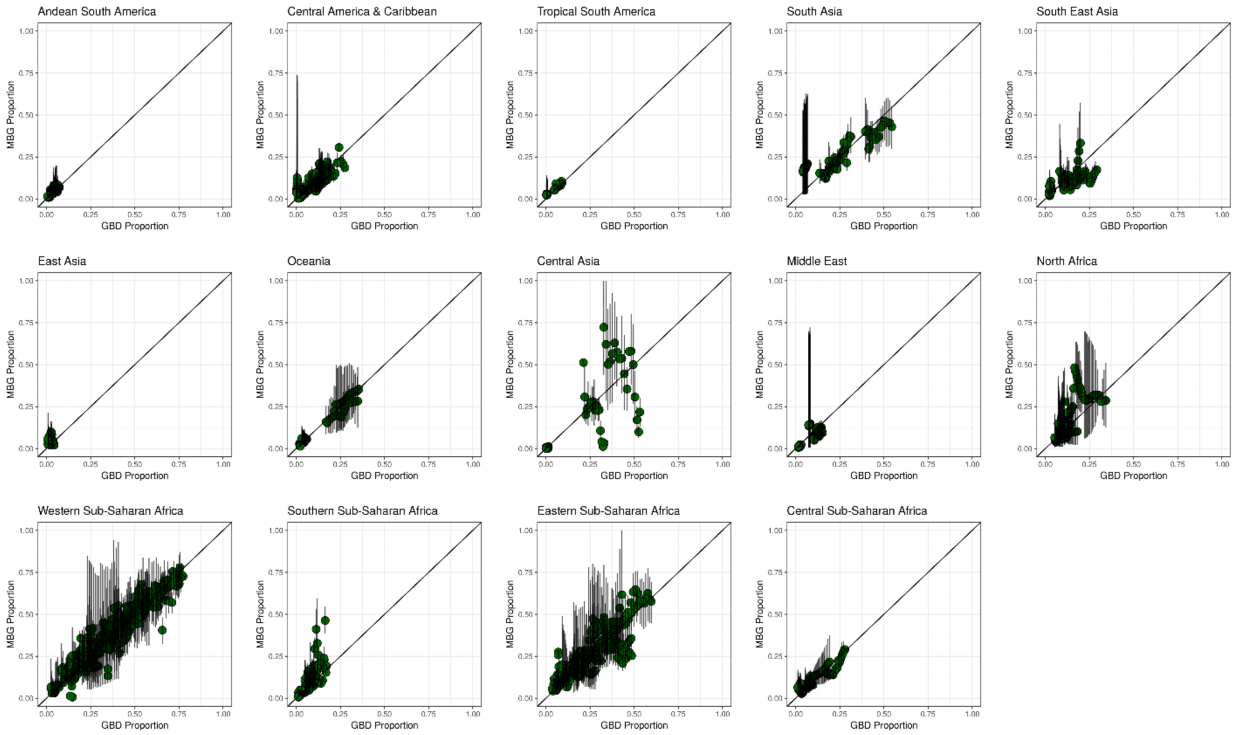

Supplementary Figure 81. Zero years of education (men, ages 15–49) comparison to GBD national estimates.

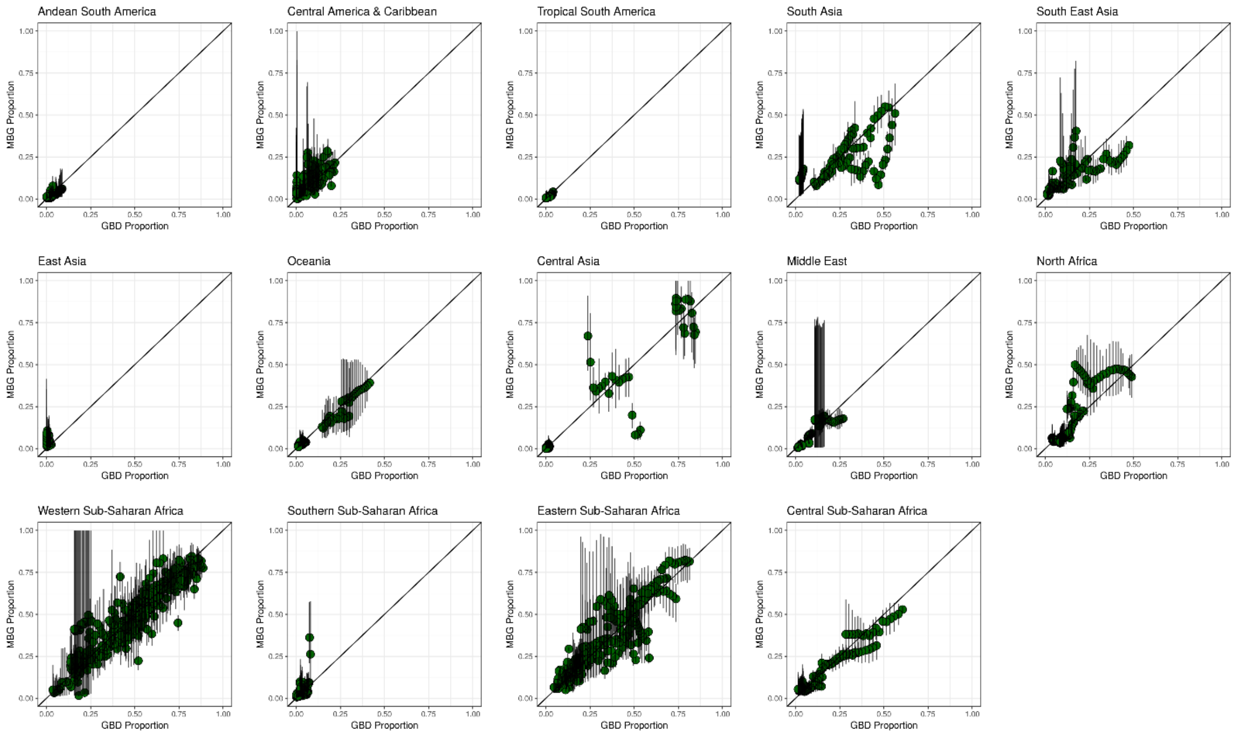

Supplementary Figure 82. Zero years of education (women, ages 20–24) comparison to GBD national estimates.

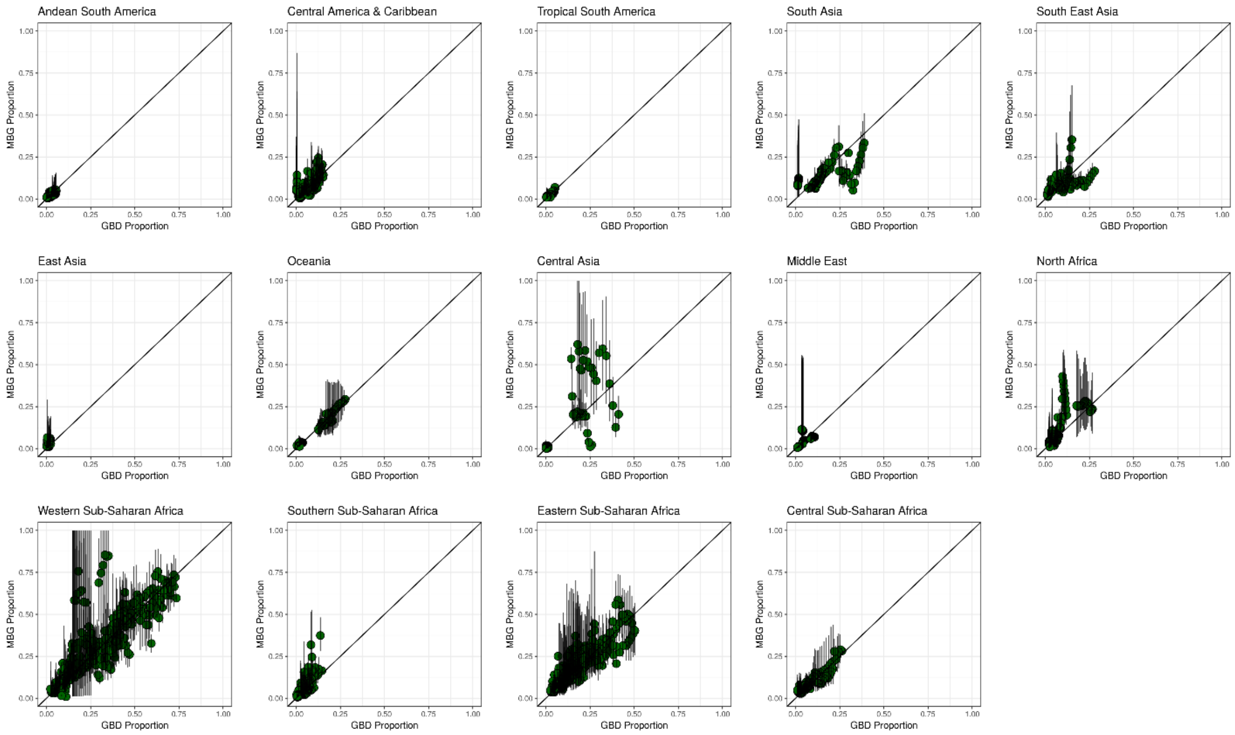

Supplementary Figure 83. Zero years of education (men, ages 20–24) comparison to GBD national estimates.

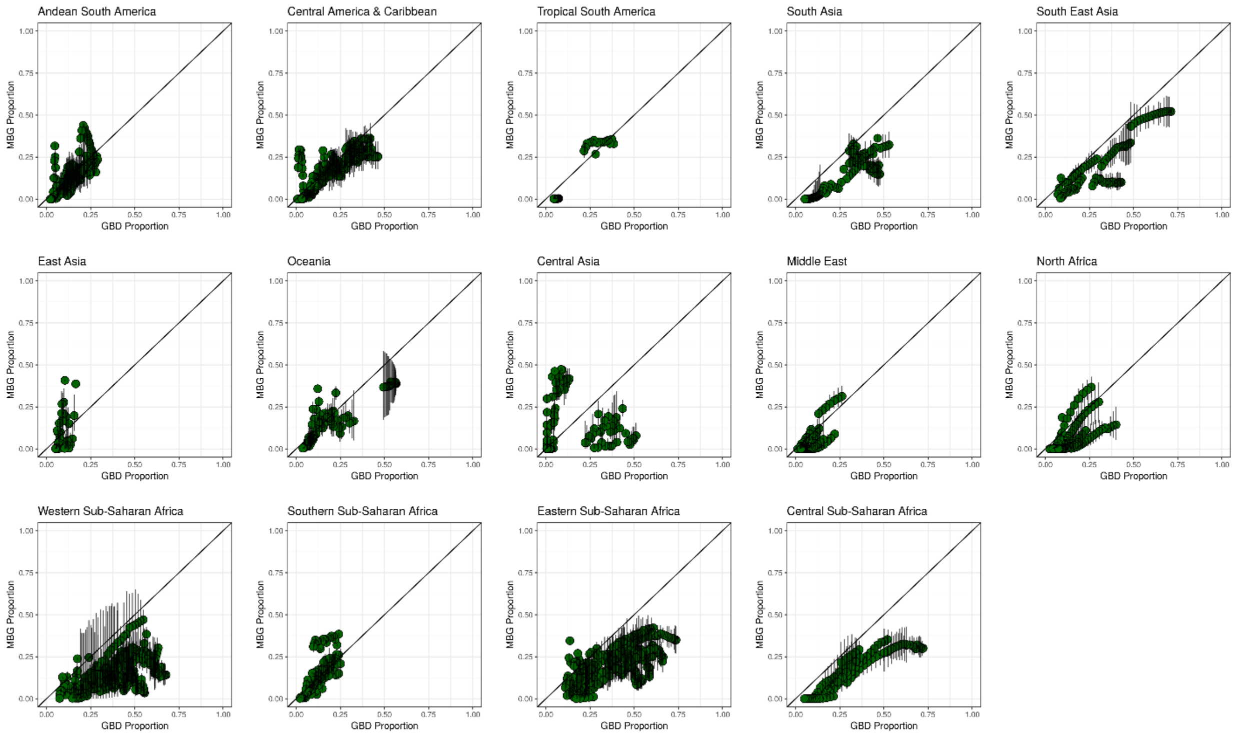

Supplementary Figure 84. 1–6 years of education (women, ages 15–49) comparison to GBD national estimates.

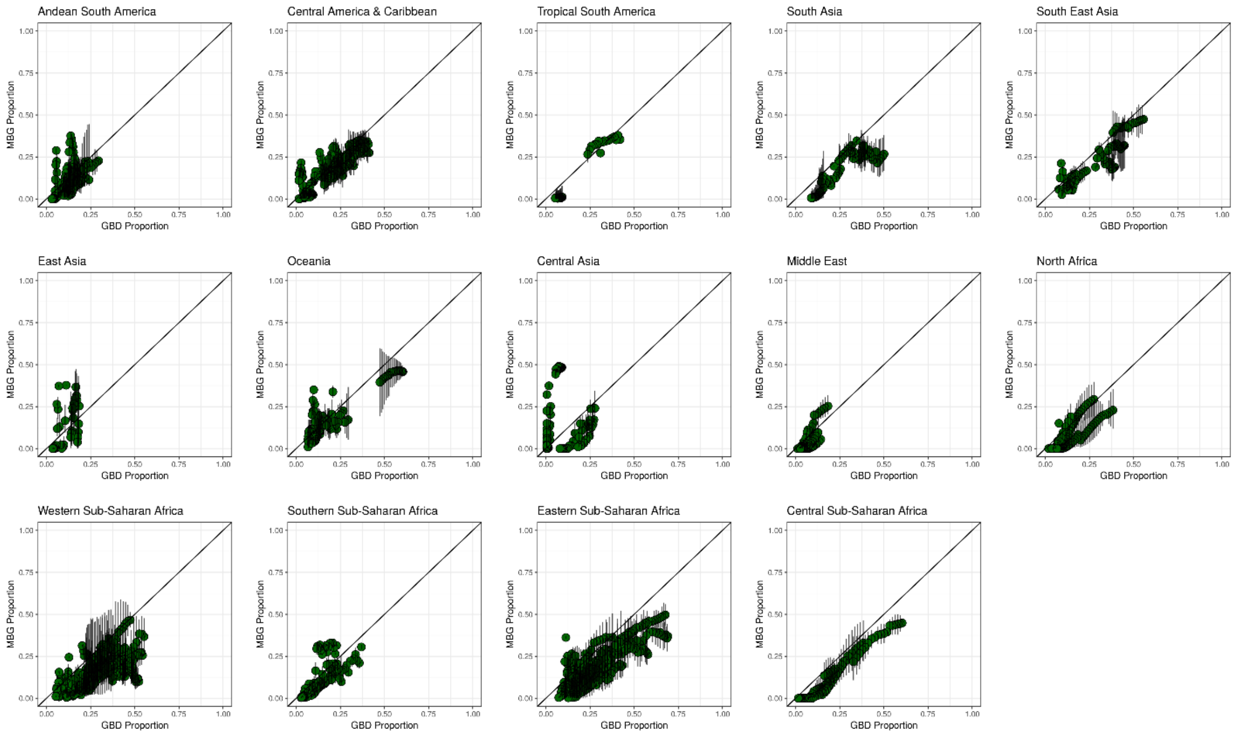

Supplementary Figure 85. 1–6 years of education (men, ages 15–49) comparison to GBD national estimates.

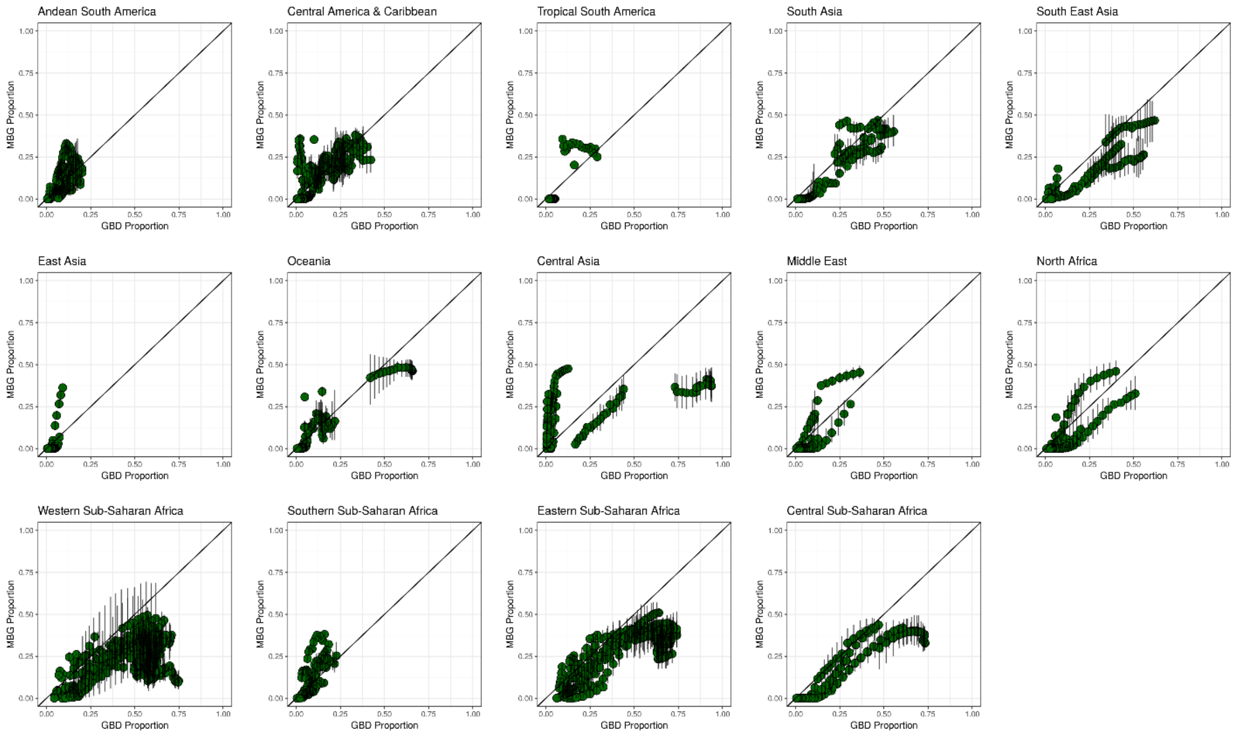

Supplementary Figure 86. 1–6 years of education (women, ages 20–24) comparison to GBD national estimates.

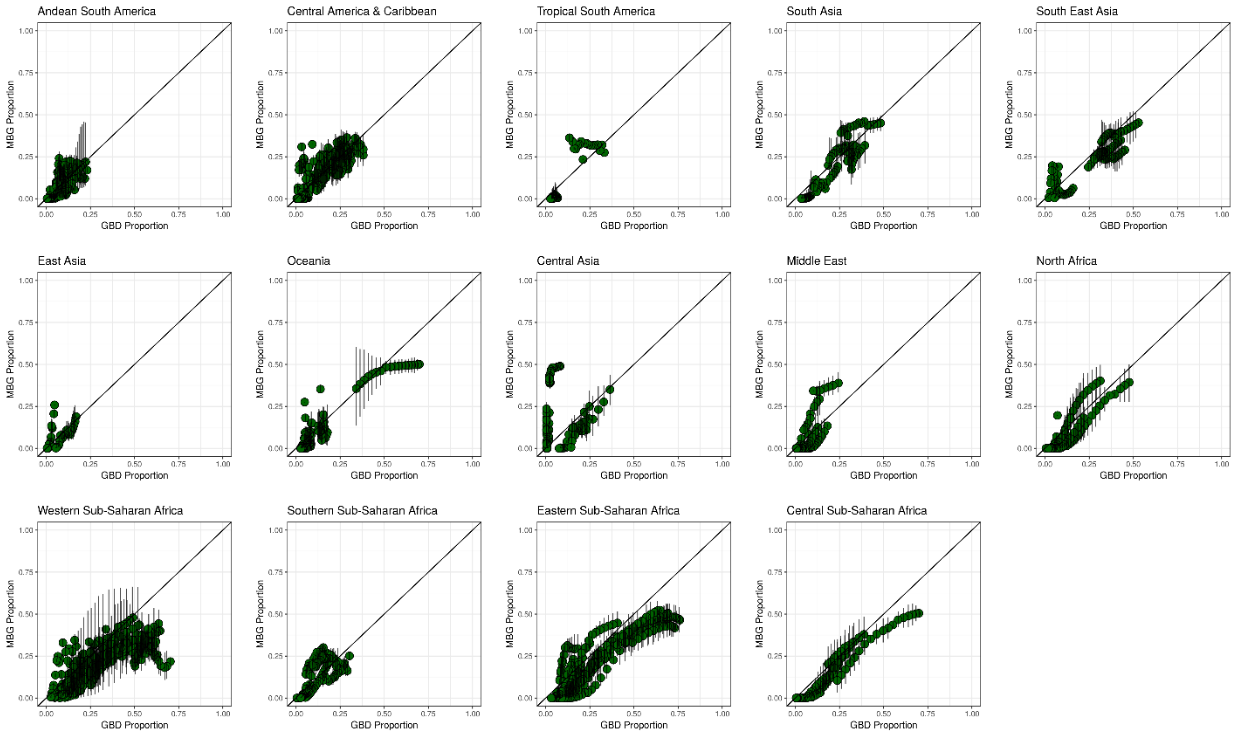

Supplementary Figure 87. 1–6 years of education (men, ages 20–24) comparison to GBD national estimates.

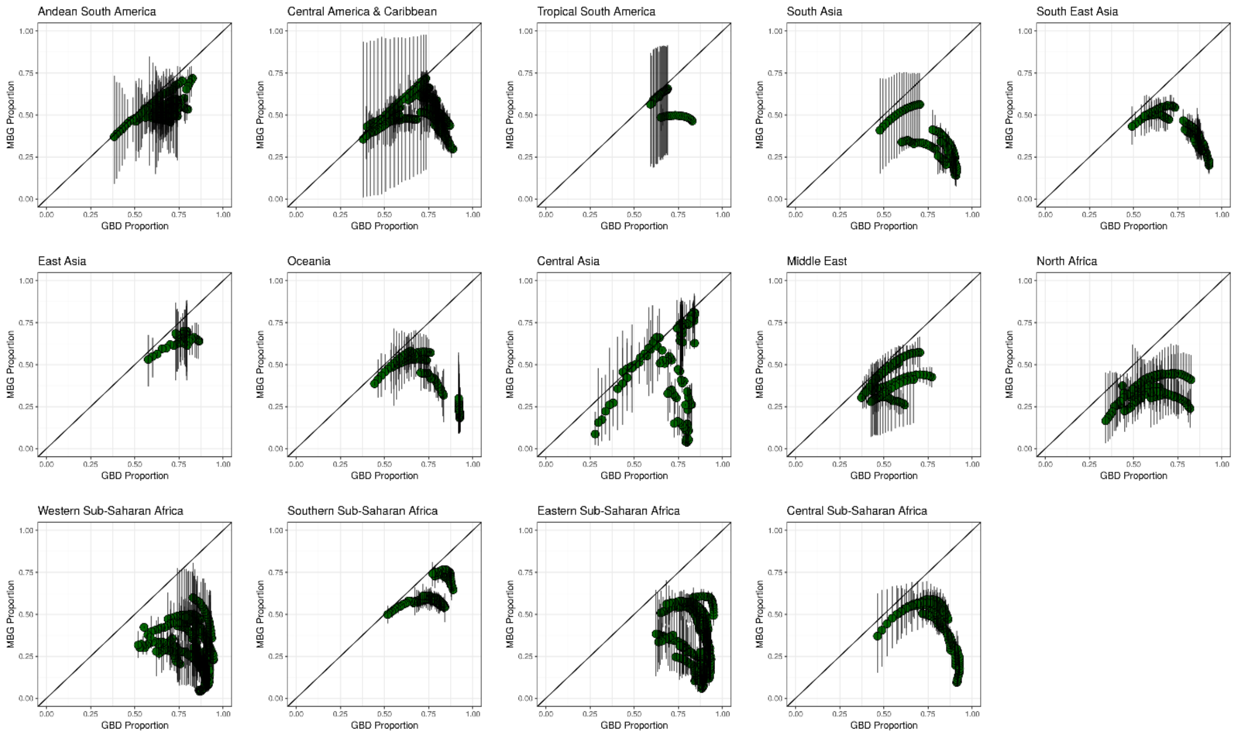

Supplementary Figure 88. 6–11 years of education (women, ages 15–49) comparison to GBD national estimates.

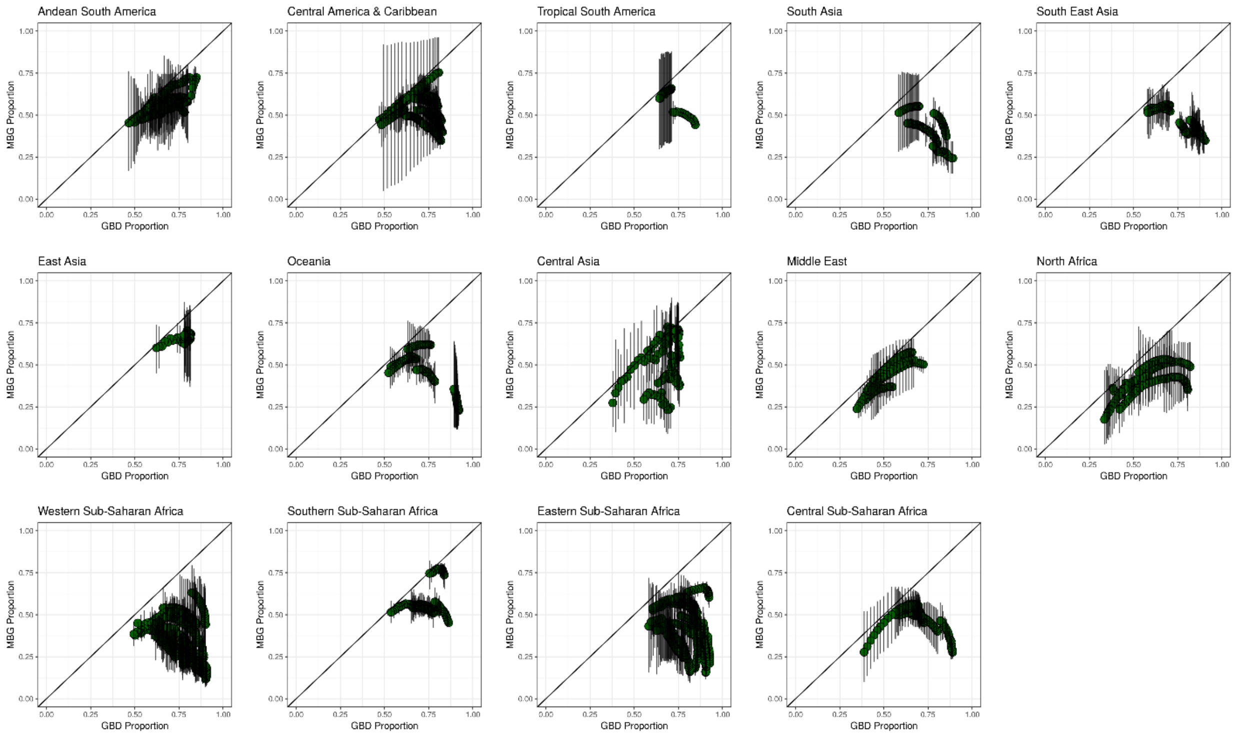

Supplementary Figure 89. 6–11 years of education (men, ages 15–49) comparison to GBD national estimates.

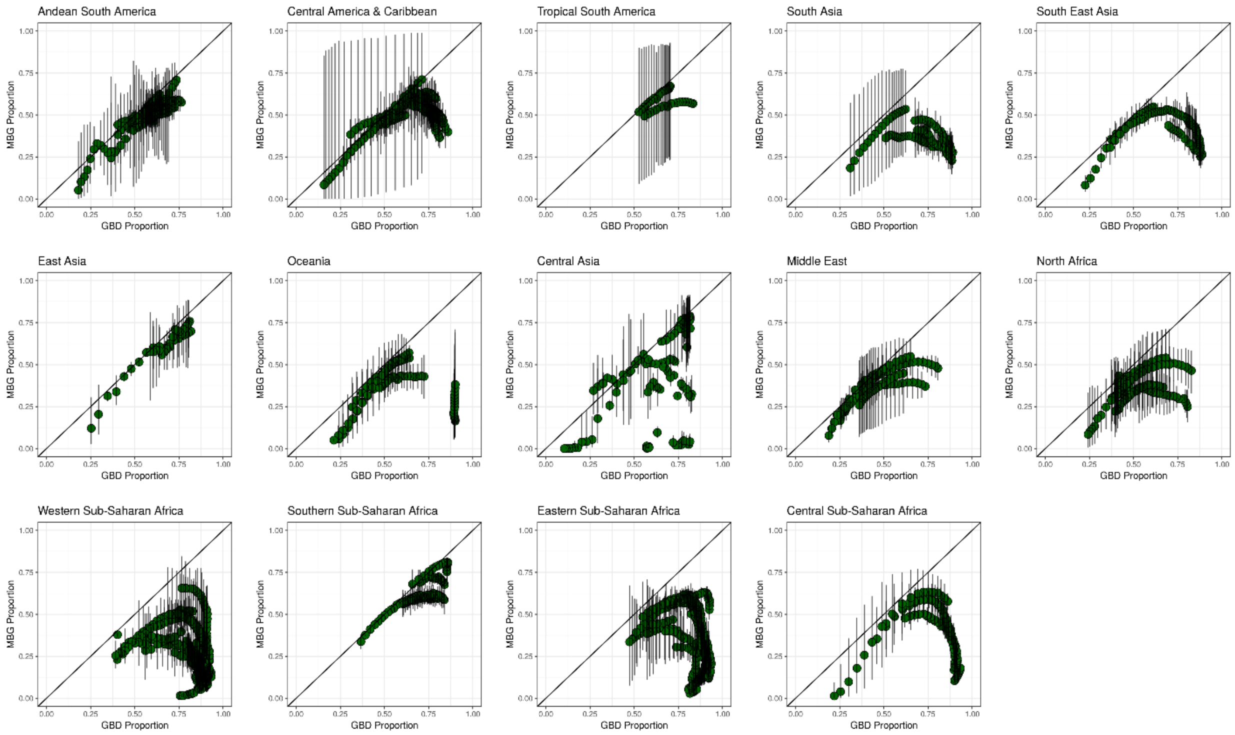

Supplementary Figure 90. 6–11 years of education (women, ages 20–24) comparison to GBD national estimates.

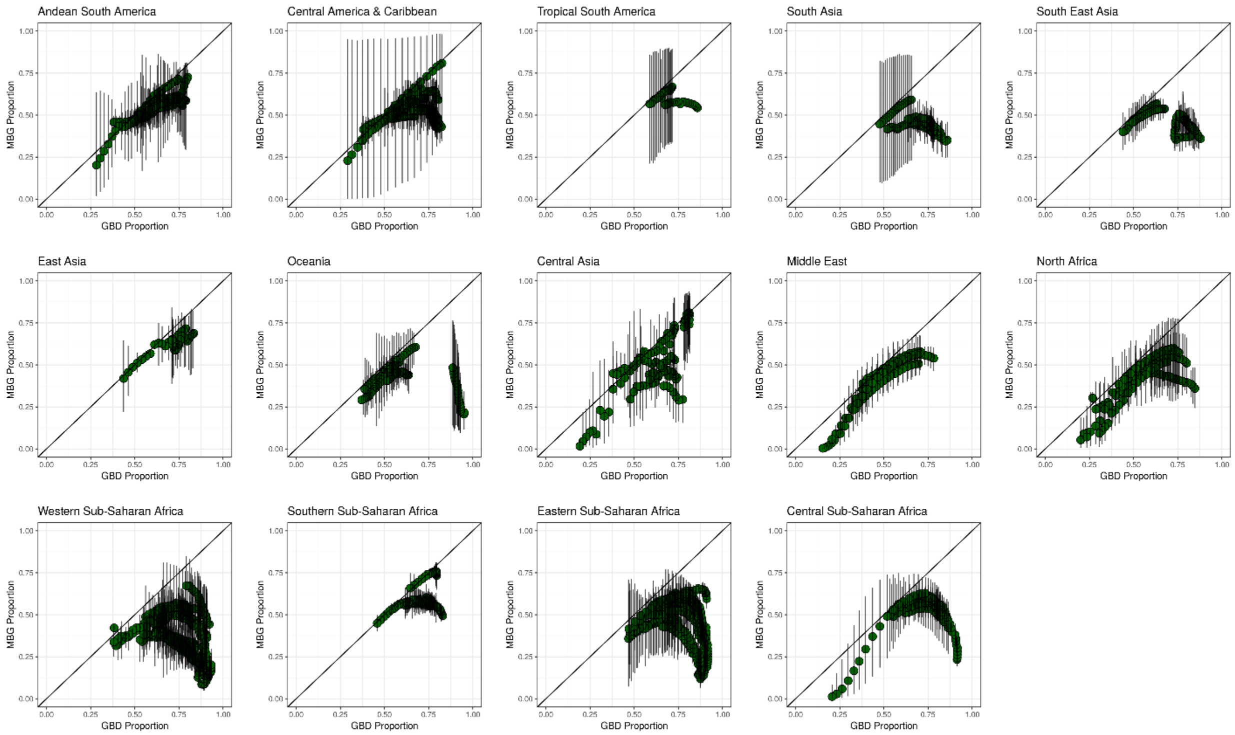

Supplementary Figure 91. 6–11 years of education (men, ages 20–24) comparison to GBD national estimates.

Supplementary Table 44. Quartiles for raking factors (RF) from MBG to GBD estimates for all indicators

Indicator	1st Quartile RF	Median RF	3rd Quartile RF
**Mean years, 15–49 male**	0.917	0.998	1.084
**Mean years, 15–49 male**	0.888	0.977	1.047
**Mean years, 20–24 female**	0.961	1.030	1.100
**Mean years, 20–24 male**	0.953	1.026	1.113
**Zero years, 15–49 female**	0.751	1.120	1.144
**Zero years, 15–49 male**	0.701	0.932	1.154
**Zero years, 20–24 female**	0.681	0.913	1.189
**Zero years, 20–24 male**	0.786	1.001	1.257
**1–5 years, 15–49 female**	0.755	0.930	1.123
**1–5 years, 15–49 male**	0.731	1.055	1.115
**1–5 years, 20–24 female**	0.694	0.981	1.278
**1–5 years, 20–24 male**	0.672	0.904	1.162
**6–11 years, 15–49 female**	0.9312	0.996	1.072
**6–11 years, 15–49 male**	0.941	0.983	1.078
**6–11 years, 20–24 female**	0.890	1.086	1.107
**6–11 years, 20–24 male**	0.879	1.091	1.066

5.3.4 Aggregation to first- and second-level administrative subdivisions

In addition to estimates of educational attainment on a grid, we also constructed estimates of attainment for first- and second-level administrative subdivisions. These estimates were derived by calculating population-weighted averages of each indicator for each grid cell or fractional grid cell within a given first- or second-level administrative subdivision. Grid cell fractions were assigned through intersection as described above and this process utilized the calibrated population estimates and prevalence draws also described above. This was carried out for each of the 1,000 posterior draws at the grid cell level, generating 1,000 posterior draws for each administrative subdivision. Final point estimates and uncertainty intervals for each subdivision at each level of the administrative hierarchy were derived from the mean, 2.5th percentile, and 97.5th percentile of these draws, respectively.

5.3.5 Calculating people with zero years of education

We estimated the number of people with zero education in each grid cell and year by combining estimated population and proportion of population with zero education after calibration to GBD as described above. Specifically, for each cell and fractional cell, we multiplied the estimated population by each of the 1,000 prevalence draws to generate 1,000 draws of people with zero education. Final point estimates and uncertainty intervals for people with zero education were calculated as the mean, 2.5th percentile, and 97.5th percentile of these draws, respectively.

6.0 Supplementary references

1. UNESCO. Meeting our commitments to gender equality in education. *Glob. Educ. Monit. Rep.* (2018).
2. Nilsson, M., Griggs, D. & Visbeck, M. Policy: Map the interactions between Sustainable Development Goals. *Nature* **534**, 320 (2016).
3. The World Bank. *Abolishing School Fees in Africa*. https://www.unicef.org/publications/files/Abolishing_School_Fees_in_Africa.pdf (2009).
4. UNESCO. *Is real progress being made in the equitable provision of education? #PISAresults*. (2017).
5. DiGirolamo, A. M., Stansbery, P. & Lung'aho, M. Advantages and challenges of integration: opportunities for integrating early childhood development and nutrition programming. *Ann. N. Y. Acad. Sci.* **1308**, 46–53 (2014).
6. Caldwell, J. C. How is greater maternal education translated into lower child mortality? *Source Health Transit. Rev.* **4**, 224–229 (1994).
7. Caldwell, J. C. Education as a Factor in Mortality Decline An Examination of Nigerian Data. *Popul. Stud.* **33**, 395 (1979).
8. Gakidou, E., Cowling, K., Lozano, R. & Murray, C. J. L. Increased educational attainment and its effect on child mortality in 175 countries between 1970 and 2009: a systematic analysis. *The Lancet* **376**, 959–974 (2010).
9. Jejeebhoy, S. J. *Women's education, autonomy, and reproductive behaviour : experience from developing countries*. (Clarendon Press, 1995).
10. Desai, S. & Alva, S. Maternal Education and Child Health: Is There a Strong Causal Relationship? *Demography* **35**, 71 (1998).
11. Basu, A. M. & Stephenson, R. Low levels of maternal education and the proximate determinants of childhood mortality: a little learning is not a dangerous thing. *Soc. Sci. Med.* **60**, 2011–2023 (2005).
12. LeVine, R. A., LeVine, S., Schnell-Anzola, B., Rowe, M. L. & Dexter, E. *Literacy and MotheringHow Women's Schooling Changes the Lives of the World's Children*. (Oxford University Press, 2012). doi:10.1093/acprof:oso/9780195309829.001.0001.
13. Ruel, M. T. & Alderman, H. Nutrition-sensitive interventions and programmes: how can they help to accelerate progress in improving maternal and child nutrition? *The Lancet* **382**, 536–551 (2013).
14. Pamuk, E., Fuchs, R. & Lutz, W. Cmparing Relative Effects of Education and Economic Resources on Infant Mortality in Developing Countries. *Popul. Dev. Rev.* **37**, 637–664 (2011).
15. Klasen, S. Does Gender Inequality Reduce Growth and Development? Evidence from Cross-Country Regressions. (2000).
16. Klasen, S. Low Schooling for Girls, Slower Growth for All? Cross-Country Evidence on the Effect of Gender Inequality in Education on Economic Development. *World Bank Econ. Rev.* **16**, 345–373 (2002).

17. Klasen, S. & Lamanna, F. The Impact of Gender Inequality in Education and Employment on Economic Growth: New Evidence for a Panel of Countries. *Fem. Econ.* **15**, 91–132 (2009).

18. Nations, U. Transforming our world: The 2030 agenda for sustainable development. *Resolut. Adopt. Gen. Assem.* (2015).

19. Horton, R. & Lo, S. Nutrition: a quintessential sustainable development goal. *The Lancet* **382**, 371–372 (2013).

20. Osgood-Zimmerman, A. *et al.* Mapping child growth failure in Africa between 2000 and 2015. *Nature* **555**, 41–47 (2018).

21. Golding, N. *et al.* Mapping under-5 and neonatal mortality in Africa, 2000–15: a baseline analysis for the Sustainable Development Goals. *The Lancet* **390**, 2171–2182 (2017).

22. Annan, K. Data can help to end malnutrition across Africa. *Nature* **555**, 7 (2018).

23. Horton, R. Offline: In defence of precision public health. *The Lancet* **392**, 1504 (2018).

24. Dowell, S. F., Blazes, D. & Desmond-Hellmann, S. Four steps to precision public health. *Nature* **540**, 181–191 (2016).

25. Barro, R. J. & Lee, J. W. A new data set of educational attainment in the world, 1950–2010. *J. Dev. Econ.* **104**, 184–198 (2013).

26. Friedman, J., Graetz, N. & Gakidou, E. Improving the estimation of educational attainment: New methods for assessing average years of schooling from binned data. *PLOS ONE* **13**, e0208019 (2018).

27. UNESCO. ISCED Mappings. http://uis.unesco.org/en/isced-mappings (2016).

28. Thomas Lumley. Survey analysis in R. http://r-survey.r-forge.r-project.org/survey/ (2014).

29. Lumley, T. Simple and Stratified Sampling. in *Complex Surveys* 17–37 (John Wiley & Sons, Inc., 2010).

30. Tatem, A. J. WorldPop, open data for spatial demography. *Sci. Data* **4**, 170004 (2017).

31. Bhatt, S. *et al.* Improved prediction accuracy for disease risk mapping using Gaussian Process stacked generalisation. *J. R. Soc. Interface* **14**, (2017).

32. Murray, C. J. *et al.* GBD 2010: design, definitions, and metrics. *The Lancet* **380**, 2063–2066 (2012).

33. Hosmer DW, Lemeshow S, S. R. Ordinal Logistic Regression Models. in *Applied logistic regression* 289–305 (Wiley, 2013).

34. Stein, M. L. *Interpolation of Spatial Data*. (Springer New York, 1999).

35. Diggle, P. & Ribeiro, P. J. *Model-based Geostatistics*. (Springer-Verlag New York, 2007).

36. Rue, H. *et al.* INLA: Functions Which Allow to Perform Full Bayesian Analysis of Latent Gaussian Models Using Integrated Nested Laplace Approximaxion. (2014).

37. Blangiardo, M., Cameletti, M., Baio, G. & Rue, H. avard. Spatial and spatio-temporal models with R-INLA. *Spat. Spatio-Temporal Epidemiol.* **7**, 39–55 (2013).

38. Cameletti, M., Lindgren, F., Simpson, D. & Rue, H. avard. Spatio-temporal modeling of particulate matter concentration through the SPDE approach. *AStA Adv. Stat. Anal.* **97**, 109–131 (2013).

39. Alegana, V. A. *et al.* Fine resolution mapping of population age-structures for health and development applications. *J. R. Soc. Interface* **12**, 20150073–20150073 (2015).

40. Patil, A. P., Gething, P. W., Piel, F. B. & Hay, S. I. Bayesian geostatistics in health cartography: the perspective of malaria. *Trends Parasitol.* **27**, 246–253 (2011).

41. Gakidou, E. *et al.* Global, regional, and national comparative risk assessment of 84 behavioural, environmental and occupational, and metabolic risks or clusters of risks, 1990–2016: a systematic analysis for the Global Burden of Disease Study 2016. *The Lancet* **390**, 1345–1422 (2017).

7.0 Authors' contributions

Managing the estimation or publications process

Amber Sligar and Aubrey J Levine.

Writing the first draft of the manuscript

Nicholas Graetz, Lauren Woyczynski, and Simon I Hay.

Primary responsibility for this manuscript focused on: applying analytical methods to produce estimates

Lauren Woyczynski, Nicholas Graetz, Michael Collison, Nathaniel J Henry, Roy Burstein, Laura Dwyer-Lindgren, Ani Deshpande, Damaris Kinyoki, and Jon Mosser.

Primary responsibility for this manuscript focused on: seeking, cataloguing, extracting, or cleaning data; designing or coding figures and tables

Kate F Wilson, Jason B Hall, John C Wilkinson, and Lucas Earl.

Managing the overall research enterprise

Farah Daoud, Aubrey J Levine, George Mensah, Ali H Mokdad, Christopher J L Murray, Benn Sartorius, Megan F Schipp, Amber Sligar, Emmanuela Gakidou, and Simon I Hay.

Providing data or critical feedback on data sources

Jason B Hall, Kalkidan Hassen Abate, Foad Abd-Allah, Oladimeji Adebayo, Fares Alahdab, Mehran Alijanzadeh, Khalid Altirkawi, Catalina Liliana Andrei, Mina Anjomshoa, Jalal Arabloo, Olatunde Aremu, Bahram Armoon, Marcel Ausloos, Ashish Awasthi, Beatriz Paulina Ayala Quintanilla, Samad Azari, Neeraj Bedi, Somayeh Bohlouli, Gabrielle Britton, Roy Burstein, Zahid Butt, Luis Cámera, Carlos Castañeda-Orjuela, Franz Castro, Cyrus Cooper, Aso Darwesh, Rajat Das Gupta, Jan-Walter De Neve, Kebede Deribe, Manisha Dubey, Lucas Earl, Ziad El-Khatib, Anwar Faraj, Mohammad Fareed, Andre Faro, Irina Filip, Takeshi Fukumoto, Sameer Gopalani, Ayman Grada, Hamid Y Hassen, Claudiu Herteliu, Long Hoang Nguyen, Michael Hole, Mehdi Hosseinzadeh, Guoqing Hu, Sheikh Mohammed Shariful Islam, Nader Jafaribalalami , Mihajlo Jakovljevic, John Ji, Jost B Jonas, Jacek Jozwiak, Tanuj Kanchan, Amir Kasaeian, Amir Kasaeian, Peter Keiyoro, Yousef Khader, Morteza Abdullatif Khafaie, Ali Kiadaliri, Yun Jin Kim, Adnan Kisa, Kewal Krishan, Barthelemy Kuate Defo, Pushpendra Kumar, Paul Lee, Yu Liao, Lee-Ling Lim, Jaifred Christian Lopez, Francisco Rogerlândio Martins-Melo, Anthony Masaka, Benjamin Massenburg, Benjamin Mayala, Walter Mendoza, Tuomo Meretoja, Ted R Miller, Erkin Mirrakhimov, Babak Moazen, Dara Mohammad, Naser Mohammad Gholi Mezerji, Shafiu Mohammed, Ali Mokdad, Lorenzo Monasta, Maziar Moradi-Lakeh, Seyyed Meysam Mousavi, Christopher J L Murray, Ghulam Mustafa, Seyed Sina Naghibi Irvani, Ionut Negoi, Josephine Ngunjiri, Malihe Nourollahpour Shiadeh , Felix Ogbo, Andrew T Olagunju, Bolajoko Olusanya, Jacob Olusanya, Obinna Onwujekwe , Eduardo Ortiz-Panozo, Mahesh P A, Adrian Pana, Songhomitra Panda-Jonas, Sanghamitra Pati, Maarten Postma, Mostafa Qorbani, Amir Radfar, Fakher Rahim, Fatemeh Rajati, Chhabi Lal Ranabhat, David Rawaf, Salman Rawaf, Carlos Rios-González, Leonardo Roever, Gholamreza Roshandel, Ali Rostami, Enrico Rubagotti, Ehsan Sadeghi, Rajesh Sagar, Nasir Salam, Payman Salamati, Hamideh Salimzadeh, Abdallah M Samy, Juan Sanabria , David C Schwebel, Masood Ali Shaikh, Mehran Shams-Beyranvand, Morteza Shamsizadeh, Mehdi Sharif, Soraya Siabani, Dhirendra Narain Sinha, Ireneous Soyiri, Chandrashekhar T Sreeramareddy, Agus Sudaryanto, Cuong Tat Nguyen, Mohamad-Hani Temsah, Abdullah Terkawi, Zemenu Tessema, Giang Thu

Vu, Marcos Roberto Tovani-Palone, Bach Tran, Irfan Ullah, Pascual Valdez, Yousef Veisani, Vasiliy Vlassov, Yasir Waheed, John Wilkinson, Charles Wolfe, Tomohide Yamada, Alex Yeshaneh, Naohiro Yonemoto, Mustafa Younis, Chuanhua Yu, Yunquan Zhang, Sanjay Zodpey, Emmanuela Gakidou, and Simon I Hay.

Development of methods or computational machinery

Katherine F Wilson, Jason B Hall, Samad Azari, Roy Burstein, Michael Collison, Michael A Cork, Aso Darwesh, Aniruddha Deshpande, Lucas Earl, Anwar Faraj, Nathaniel Henry, Mehdi Hosseinzadeh, Nader Jafaribalalami, Adnan Kisa, Ali Mokdad, Jonathan Mosser, Christopher J L Murray, Chhabi Lal Ranabhat, Robert Reiner, Carlos Rios-González, Abdallah M Samy, Mehdi Sharif, Emmanuela Gakidou, and Simon I Hay.

Providing critical feedback on methods or results

Jason B Hall, Kalkidan Hassen Abate, Foad Abd-Allah, Oladimeji Adebayo, Victor Adekanmbi, Mahdi Afshari, Fares Alahdab, Ziyad Al-Aly, Jacqueline Alcalde Rabanal, Vahid Alipour, Khalid Altirkawi, Nahla Anber, Catalina Liliana Andrei, Mina Anjomshoa, Carl Abelardo T Antonio, Jalal Arabloo, Olatunde Aremu, Suleman Atique, Marcel Ausloos, Ashish Awasthi, Beatriz Paulina Ayala Quintanilla, Samad Azari, Alaa Badawi, Joseph Banoub, Suzanne Barker-Collo, Derrick Bennett, Natalia Bhattacharjee, Krittika Bhattacharyya, Suraj Bhattarai, Ali Bijani, Boris Bikbov, Somayeh Bohlouli, Gabrielle Britton, Roy Burstein, Zahid Butt, Luis Cámera, Rosario Cárdenas, Félix Carvalho, Franz Castro, Ester Cerin, Jung-Chen Chang, Cyrus Cooper, Michael A Cork, Farah Daoud, Aso Darwesh, Rajat Das Gupta, Jan-Walter De Neve, Kebede Deribe, Beruk Desalegn, Aniruddha Deshpande, Melaku Desta, Daniel Diaz, Shirin Djalalinia, Manisha Dubey, Andre Duraes, Mohammad Ebrahimi Kalan, Ziad El-Khatib, Anwar Faraj, Mahbobeh Faramarzi, Mohammad Fareed, Andre Faro, Seyed-Mohammad Fereshtehnejad, Eduarda Fernandes, Irina Filip, Florian Fischer, Takeshi Fukumoto, Paramjit Gill, Philimon Gona, Sameer Gopalani, Ayman Grada, Yuming Guo, Rajeev Gupta, Vipin Gupta, Arvin Haj-Mirzaian, Arya Haj-Mirzaian, Samer Hamidi, Mehedi Hasan, Hamid Y Hassen, Delia Hendrie, Nathaniel Henry, Bernardo Hernandez, Claudiu Herteliu, Long Hoang Nguyen, Michael Hole, Naznin Hossain, Mehdi Hosseinzadeh, Guoqing Hu, Olayinka Ilesanmi, Sheikh Mohammed Shariful Islam, Neda Izadi, Nader Jafaribalalami , Mihajlo Jakovljevic, Ravi Prakash Jha, John Ji, Jost B Jonas, Zahra Jorjoran Shushtari, Jacek Jozwiak, Tanuj Kanchan, Amir Kasaeian, Amir Kasaeian, Peter Keiyoro, Yousef Khader, Morteza Abdullatif Khafaie, Ejaz Khan, Mona Khater, Ali Kiadaliri, Yun Jin Kim, Ruth Kimokoti, Damaris Kinyoki, Adnan Kisa, Hamidreza Komaki, Ai Koyanagi, Kewal Krishan, Barthelemy Kuate Defo, Manasi Kumar, Pushpendra Kumar, Faris Lami, Shanshan Li, Yu Liao, Lee-Ling Lim, Stefan Listl, Jaifred Christian Lopez, Reza Malekzadeh, Mohammad Ali Mansournia , Francisco Rogerlândio Martins-Melo, Anthony Masaka, Benjamin Massenburg, Benjamin Mayala, Kala M Mehta, Walter Mendoza, George Mensah, Tuomo Meretoja, Ted R Miller, Gk Mini, Erkin Mirrakhimov, Babak Moazen, Dara Mohammad, Naser Mohammad Gholi Mezerji, Shafiu Mohammed, Farnam Mohebi, Ali Mokdad, Lorenzo Monasta, Yoshan Moodley, Mahmood Moosazadeh, Ghobad Moradi, Maziar Moradi-Lakeh, Paula Moraga, Lidia Morawska, Shane Morrison, Jonathan Mosser, Seyyed Meysam Mousavi, Christopher J L Murray, Ghulam Mustafa, Seyed Sina Naghibi Irvani, Farid Najafi, Duduzile Ndwandwe, Ionut Negoi, Ruxandra Irina Negoi, Josephine Ngunjiri, Dina Nur Anggraini Ningrum, Malihe Nourollahpour Shiadeh , Felix Ogbo, Andrew T Olagunju, Bolajoko Olusanya, Jacob Olusanya, Obinna Onwujekwe , Doris V Ortega-Altamirano, Eduardo Ortiz-Panozo, Simon Øverland, Mahesh P A, Adrian Pana, Songhomitra Panda-Jonas, Sanghamitra Pati, George Patton, Norberto Perico, David Pigott, Meghdad Pirsaheb, Maarten Postma, Akram Pourshams, Mostafa Qorbani, Amir Radfar,

Fakher Rahim, Vafa Rahimi-Movaghar, Chhabi Lal Ranabhat, David Rawaf, Salman Rawaf, Robert Reiner, Giuseppe Remuzzi, Andre Renzaho, Aziz Rezapour, Carlos Rios-González, Leonardo Roever, Gholamreza Roshandel, Ali Rostami, Enrico Rubagotti, Ehsan Sadeghi, Yahya Safari, Roya Safari-Faramani, Rajesh Sagar, Payman Salamati, Yahya Salimi, Hamideh Salimzadeh, Abdallah M Samy, Juan Sanabria , Milena Santric-Milicevic, Benn Sartorius, Brijesh Sathian, Lauren Schaeffer, David C Schwebel, Sadaf Sepanlou, Masood Ali Shaikh, Mehran Shams-Beyranvand, Morteza Shamsizadeh, Kiomars Sharafi, Mehdi Sharif, Rajesh Sharma, Aziz Sheikh, Mika Shigematsu, Soraya Siabani, Jasvinder Singh, Dhirendra Narain Sinha, Joan B Soriano, Ireneous Soyiri, Chandrashekhar T Sreeramareddy, Agus Sudaryanto, Muawiyyah Babale Sufiyan, Ipsita Sutradhar , Pn Sylaja, Rafael Tabarés-Seisdedos, Birkneh Tilahun Tadesse, Cuong Tat Nguyen, Zemenu Tessema, Giang Thu Vu, Marcos Roberto Tovani-Palone, Bach Tran, Lorainne Tudor Car, Irfan Ullah, Pascual Valdez, Vasiliy Vlassov, Sebastian Vollmer, Yasir Waheed, Yuan-Pang Wang, Andrea Winkler, Tomohide Yamada, Alex Yeshaneh, Paul Yip, Engida Yisma, Naohiro Yonemoto, Mustafa Younis, Chuanhua Yu, Sojib Bin Zaman, Yunquan Zhang, Sanjay Zodpey, Emmanuela Gakidou, and Simon I Hay.

Drafting the manuscript or revising is critically for important intellectual content

Foad Abd-Allah, Oladimeji Adebayo, Victor Adekanmbi, Fares Alahdab, Jacqueline Alcalde, Mehran Alijanzadeh, Nahla Anber, Catalina Liliana Andrei, Carl Abelardo T Antonio, Jalal Arabloo, Olatunde Aremu, Mehran Asadi, Marcel Ausloos, Ashish Awasthi, Suzanne Barker-Collo, Anthony Barnett, Neeraj Bedi, Krittika Bhattacharyya, Zahid Butt, Félix Carvalho, Carlos Castañeda-Orjuela, Franz Castro, Ester Cerin, Cyrus Cooper, Aso Darwesh, Rajat Das Gupta, Jan-Walter De Neve, Kebede Deribe, Beruk Desalegn, melaku desta, Daniel Diaz, Manisha Dubey, Laura Dwyer-Lindgren, Lucas Earl, Anwar Faraj, Andre Faro, Seyed-Mohammad Fereshtehnejad, Eduarda Fernandes, Irina Filip, Florian Fischer, Takeshi Fukumoto, Tiffany K Gill, Philimon Gona, Sameer Gopalani, Ayman Grada, Yuming Guo, Rajeev Gupta, Randah Hamadeh, Mehedi Hasan, Hamid Y. Hassen, Claudiu Herteliu, Long Hoang Nguyen, Michael Hole, Naznin Hossain, Mehdi Hosseinzadeh, Olayinka Ilesanmi, Sheikh Mohammed Shariful Islam, Nader Jafaribalalami , Mihajlo Jakovljevic, Ravi Prakash Jha, Jost B. Jonas, Jacek Jozwiak, Amir Kasaeian, Amir Kasaeian, Peter Keiyoro, Chandrasekharan Nair Kesavachandran, Yousef Khader, morteza abdullatif khafaie, Ejaz Khan, Mona Khater, Ali Kiadaliri, Hamidreza Komaki, Ai Koyanagi, Barthelemy Kuate Defo, Manasi Kumar, Paul Lee, Shanshan Li, Lee-Ling Lim, Stefan Listl, Jaifred Christian Lopez, Azeem Majeed, Reza Malekzadeh, Francisco Rogerlândio Martins-Melo, Anthony Masaka, Benjamin Massenburg, Walter Mendoza, Tuomo Meretoja, Tomislav Mestrovic, Ted R Miller, Babak Moazen, Dara Mohammad, Shafiu Mohammed, Farnam Mohebi, Ali Mokdad, Lorenzo Monasta, Ghobad Moradi, Maziar Moradi-Lakeh, Paula Moraga, Shane Morrison, Jonathan Mosser, Christopher J L Murray, Seyed Sina Naghibi Irvani, Azin Nahvijou, Ionut Negoi, Ruxandra Irina Negoi, Josephine Ngunjiri, Felix Ogbo, Andrew T. Olagunju, Bolajoko Olusanya, Jacob Olusanya, Obinna Onwujekwe , Doris V. Ortega-Altamirano, Eduardo Ortiz-Panozo, Simon Øverland, Mahesh P A, Adrian Pana, Songhomitra Panda-Jonas, Sanghamitra Pati, George Patton, David Pigott, Maarten Postma, Mostafa Qorbani, Amir Radfar, Fakher Rahim, VAFA RAHIMI-MOVAGHAR, Chhabi Lal Ranabhat, Carlos Rios-González, Leonardo Roever, Enrico Rubagotti, Nasir Salam, Hamideh Salimzadeh, Abdallah M. Samy, Juan Sanabria , Milena Santric-Milicevic, David C Schwebel, Sadaf Sepanlou, Masood Ali Shaikh, Mehdi Sharif, Mika Shigematsu, jasvinder singh, Dhirendra Narain Sinha, Joan B Soriano, Ireneous Soyiri, chandrashekhar T sreeramareddy, Muawiyyah Babale Sufiyan, Ipsita Sutradhar , Cuong Tat Nguyen, Mohamad-Hani Temsah, Zemenu Tessema, Giang Thu Vu, Marcos Roberto Tovani-Palone, Bach Tran, Irfan Ullah, Francesco S Violante, Vasiliy Vlassov,

Yuan-Pang Wang, Andrea Winkler, Alex Yeshaneh, Sojib Bin Zaman, Jianrong Zhang, Emmanuela Gakidou, and Simon I Hay.
