# Supplementary material for: Mapping disparities in education across low- and middle-income countries
Source: Nature. 2019 Dec 25;577(7789):235–8. doi: 10.1038/s41586-019-1872-1 (PMC7015853; doi:10.1038/s41586-019-1872-1)
Supplement: Supplementary file 2 — Reporting Summary [file 41586_2019_1872_MOESM2_ESM.pdf]

nature research

Corresponding author(s): Simon I.Hay and Nick Graetz

Reporting Summary

Nature Research wishes to improve the reproducibility of the work that we publish. This form provides structure for consistency and transparency in reporting. For further information on Nature Research policies, see Authors & Referees and the Editorial Policy Checklist.

Statistical parameters

When statistical analyses are reported, confirm that the following items are present in the relevant location (e.g. figure legend, table legend, main text, or Methods section).

n/a	Confirmed	
☐	☒	The exact sample size (n) for each experimental group/condition, given as a discrete number and unit of measurement
☐	☒	An indication of whether measurements were taken from distinct samples or whether the same sample was measured repeatedly
☒	☐	The statistical test(s) used AND whether they are one- or two-sided<br>*Only common tests should be described solely by name; describe more complex techniques in the Methods section.*
☐	☒	A description of all covariates tested
☐	☒	A description of any assumptions or corrections, such as tests of normality and adjustment for multiple comparisons
☐	☒	A full description of the statistics including central tendency (e.g. means) or other basic estimates (e.g. regression coefficient) AND variation (e.g. standard deviation) or associated estimates of uncertainty (e.g. confidence intervals)
☒	☐	For null hypothesis testing, the test statistic (e.g. F, t, r) with confidence intervals, effect sizes, degrees of freedom and P value noted<br>*Give P values as exact values whenever suitable.*
☐	☒	For Bayesian analysis, information on the choice of priors and Markov chain Monte Carlo settings
☒	☐	For hierarchical and complex designs, identification of the appropriate level for tests and full reporting of outcomes
☒	☐	Estimates of effect sizes (e.g. Cohen's d, Pearson's r), indicating how they were calculated
☐	☒	Clearly defined error bars<br>*State explicitly what error bars represent (e.g. SD, SE, CI)*

Our web collection on statistics for biologists may be useful.

Software and code

Policy information about availability of computer code

Data collection	No primary data collection was carried out for this analysis.
Data analysis	This analysis was carried out using R version 3.5.0. The main geostatistical models were fit using R-INLA version 18.07.12. All code used for these analyses is publicly available online at http://ghdx.healthdata.org/.

For manuscripts utilizing custom algorithms or software that are central to the research but not yet described in published literature, software must be made available to editors/reviewers upon request. We strongly encourage code deposition in a community repository (e.g. GitHub). See the Nature Research guidelines for submitting code & software for further information.

Data

Policy information about availability of data

All manuscripts must include a data availability statement. This statement should provide the following information, where applicable:

- Accession codes, unique identifiers, or web links for publicly available datasets
- A list of figures that have associated raw data
- A description of any restrictions on data availability

The findings of this study are supported by data that are available in public online repositories, data that are publicly available upon request from the data provider, and data that are not publicly available due to restrictions by the data provider and which were used under license for the current study. A detailed table of data

sources and availability can be found in the Supplemental Information.

Administrative boundaries were retrieved from the Global Administrative Unit Layers (GAUL) dataset, implemented by FAO within the CountrySTAT and Agricultural Market Information System (AMIS) projects. Land cover was retrieved from the online Data Pool, courtesy of the NASA EOSDIS Land Processes Distributed Active Archive Center (LP DAAC), USGS/Earth Resources Observation and Science (EROS) Center, Sioux Falls, South Dakota. Lakes were retrieved from the Global Lakes and Wetlands Database (GLWD), courtesy of the World Wildlife Fund and the Center for Environmental Systems Research, University of Kassel. Populations were retrieved from WorldPop.

Field-specific reporting

Please select the best fit for your research. If you are not sure, read the appropriate sections before making your selection.

☒ Life sciences ☐ Behavioural & social sciences ☐ Ecological, evolutionary & environmental sciences

For a reference copy of the document with all sections, see nature.com/authors/policies/ReportingSummary-flat.pdf

Life sciences study design

All studies must disclose on these points even when the disclosure is negative.

Sample size	Sample size was calculated as the number of unique data source location-pairs with observations of years of educational attainment for males and females. This sample size is reported in the methods section as "We compiled a database of survey and census datasets that contained geocoding of subnational administrative boundaries or GPS coordinates for sampled clusters. These included datasets from 528 sources (see Supplementary Table 2).
Data exclusions	As described n the methods section of the main text (with greater detail in the Supplementary Information) we did not include data from Western Sahara or French Guiana, due to lack of availability. 42 of 105 included countries have only subnational administrative level data. Employing a Bayesian model-based geostatistical framework and synthesizing geolocated data from 517 household and census datasets, this analysis provides subnational estimates of mean years of education and proportion of the population attaining key levels of education for women of reproductive age (15-49), women age 20-24, and equivalent male age-bins between 2000-2017 in low- and middle-income countries. This includes 105 countries across all low- and middle-income countries. Countries were selected for inclusion in this analysis using the Socio-demographic Index (SDI) published in the GBD46. The SDI is a measure of development that combines education, fertility, and poverty. Countries in the Middle, Lower-Middle, or Low SDI quintiles were included, with several exceptions. Albania, Bosnia, and Moldova were excluded despite Middle SDI status due to geographic discontinuity with other included countries and lack of available survey data. Libya, Malaysia, Panama, and Turkmenistan were included despite Higher-Middle SDI status to create better geographic continuity. We do not estimate for American Samoa, Federated States of Micronesia, Fiji, Kiribati, Marshall Islands, Samoa, Solomon Islands, or Tonga, where no available survey data could be sourced.
Replication	This is an observational study using many years of survey and surveillance data and could be replicated.
Randomization	This analysis is an observational mapping study and there were no experimental groups.
Blinding	Blinding was not relevant to this study, as it was an observational study using survey and surveillance data.

Reporting for specific materials, systems and methods

Materials & experimental systems

n/a	Involved in the study	
☒	☐	Unique biological materials
☒	☐	Antibodies
☒	☐	Eukaryotic cell lines
☒	☐	Palaeontology
☒	☐	Animals and other organisms
☒	☐	Human research participants

Methods

n/a	Involved in the study	
☒	☐	ChIP-seq
☒	☐	Flow cytometry
☒	☐	MRI-based neuroimaging
